# Coordination‐Driven Direct C─H Metalation of *N*‐Heterocycles With a Superbasic Co(II) Amide Co(TMP)_2_

**DOI:** 10.1002/anie.7728005

**Published:** 2026-06-15

**Authors:** Na Jin, Manting Mu, Max García‐Melchor, Eva Hevia

**Affiliations:** ^1^ Departement Für Chemie, Biochemie und Pharmazie Universität Bern 3012 Bern Switzerland; ^2^ School of Chemistry Trinity College Dublin College Green Dublin 2 Ireland; ^3^ CIC energiGUNE Parque Tecnológico de Álava Vitoria‐Gasteiz Spain; ^4^ IKERBASQUE Basque Foundation for Science Bilbao Spain

**Keywords:** cobalt, coordination effects, DFT calculations, diazines, metalation, pyridine

## Abstract

Metalation of *N*‐heterocycles such as pyridines or diazines with conventional alkali metal bases is particularly challenging due to the limited activation of their C─H bonds coupled with the thermal fragility of the resulting organometallic intermediates. Building upon previous work in this area, we demonstrate that the superbasic cobalt(II) amide Co(TMP)_2_ (TMP = 2,2,6,6‐tetramethylpiperidide) enables high‐yielding and highly regioselective C─H metalation of a broad range of *N*‐heterocycles via direct, redox‐neutral Co(II)–H exchange. This reactivity extends beyond single‐site activation, with controlled di‐cobaltation of non‐activated substrates such as pyrimidine and pyrazine. Via a combination of NMR reaction monitoring, isolation and characterisation of key organometallic intermediates, and density functional theory calculations, we establish that regioselectivity is dictated by coordination effects at positions *α* to nitrogen, overriding conventional trends based solely on C─H acidity. Exploiting cobalt's redox flexibility, oxidative treatment of the metalated intermediates with iodine enables selective C─N bond formation, giving sterically demanding TMP‐substituted heterocycles. Collectively, these findings establish Co(TMP)_2_ as a mechanistically distinct and versatile reagent for the selective functionalisation of *N*‐heterocycles.

## Graphical abstract

By enabling regioselectivities that are inaccessible with conventional bases, this study introduces a Co(II) amide platform for the deprotonative C─H metalation of sensitive N‐heterocycles. Subsequent interception of the resulting Co(II) intermediates with external oxidants affords a family of sterically congested, TMP‐substituted heterocycles via C─N bond formation. (TMP = 2,2,6,6‐tetramethylpiperidide).

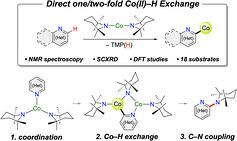

## 1 Introduction

**FIGURE 1:**
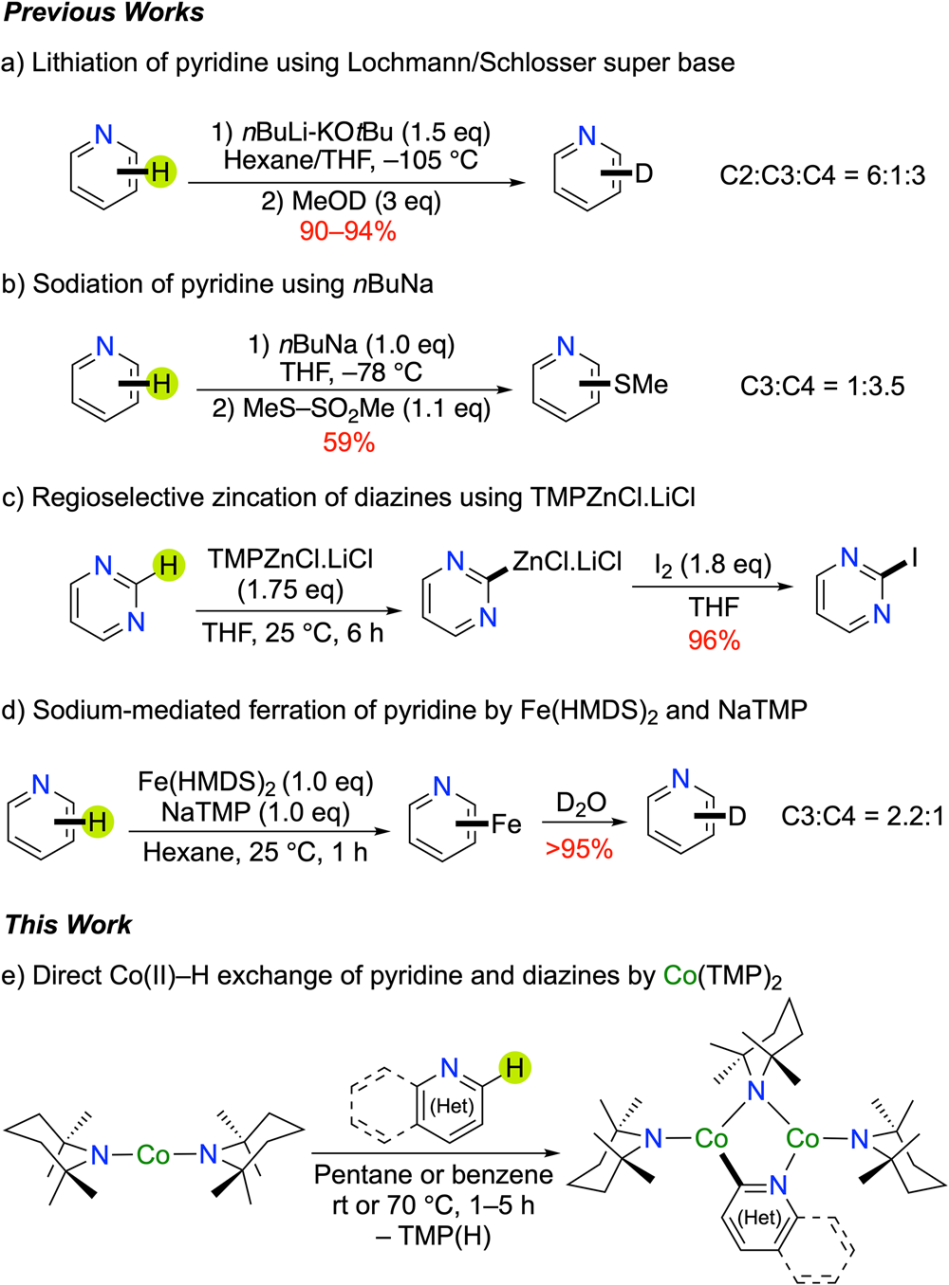
Selected examples of pyridine and pyrimidine metalation.

Typified by pyridine and pyrimidine, azines and diazines constitute an important family of *N*‐heterocyclic motifs within the structures of natural products, pharmaceuticals, agrochemicals, and functional materials [1, 2, 3, 4, 5, 6, 7, 8]. Despite their extensive utility, their metalation with conventional s‐block reagents such as alkyllithiums or lithium amides remains challenging. The low‐lying lowest unoccupied molecular orbitals (LUMOs) of these electron‐deficient heterocycles render them prone to nucleophilic addition and other side reactions upon exposure to strongly nucleophilic alkyl or aryl lithium bases [9]. As a result, extremely low temperatures and large excesses of base are often required, and even under such conditions undesired pathways occur frequently [9, 10, 11, 12, 13, 14]. For example, lithiation of pyridine with the Lochmann–Schlosser superbase (*n*BuLi/KO*t*Bu) gives a mixture of regioisomers (C2:C3:C4 = 6:1:3) even at −105°C (Figure 1a) [13]. Improved, though still cryogenic, the selectivity is achieved with the unimetallic superbase *n*BuLi.LiDMAE (LiDMAE = Me_2_N(CH_2_)_2_OLi), which affords exclusive *α*‐lithiation at −78°C when used in excess [15]. More recently, Thomas and coworkers reported that sodiation of pyridine with *n*BuNa at −78°C furnishes a 1:3.5 ratio of the C3 and C4 isomers (Figure 1b) [16]. Notably, the metalated intermediates generated in these transformations are highly fragile, even under cryogenic conditions, so their detailed structures have remained concealed [9].

To address these limitations, several heterobimetallic bases that combine Li with a less electropositive metal such as Mg, Zn, or Ga have been developed [17, 18, 19, 20, 21, 22, 23]. These reagents often generate less labile organometallic intermediates, which allows milder reaction conditions and improved regioselective control. An exemplar is Knochel's C2‐zincation of pyrimidine using TMPZnCl.LiCl (TMP = 2,2,6,6‐tetramethylpiperidide), which proceeds with high selectivity (Figure 1c) [18]. Our groups have similarly shown that combining NaTMP with the Fe(II) amide [Fe{N(SiMe_3_)_2_}_2_] affords a potent sodium ferrate capable of promoting direct ferration of pyridine via a redox‐neutral Fe(II)–H exchange. However, the selectivity of this method is limited, and quenching studies using D_2_O gave a 2.2:1 mixture of the C3 and C4 metalated isomers (Figure 1d) [24].

Motivated by the search for more basic, earth‐abundant transition metal amides that can function as regioselective bases for aromatic metalation, we recently explored the amides M(TMP)_2_ (M = Fe and Co). These reagents promote direct ferration or cobaltation of multi‐fluorinated arenes such as pentafluorobenzene and 1,2,4,5‐tetrafluorobenzene [25]. Their reactivity exhibits a distinctive superbasic character that resembles the behaviour of alkali‐metal amides, but with the added benefit that they form thermally stable organometallic intermediates.

Encouraged by these findings, we next investigated whether this approach could be extended to the regioselective functionalisation of non‐substituted pyridine and diazines. These substrates are considerably less activated than fluoroarenes in terms of C─H acidity (p*K*_a_ values) and lack pre‐installed directing groups. Here, we systematically evaluate the reactivity of these earth‐abundant transition metal amides toward a broad range of pyridines and diazines and uncover their unique ability to promote direct Co─H exchange with unprecedented control over regioselectivity (Figure 1e). Through NMR spectroscopic studies, isolation and characterisation of key organometallic intermediates, supplemented by density functional theory (DFT) calculations, we delineate the possible mechanism of these transformations and highlight the pivotal role of coordination effects in guiding the Co(II)─H exchange process.

## 2 Results and Discussion

### 2.1 Metalation of Pyridine With M(TMP)_2_

**FIGURE 2:**
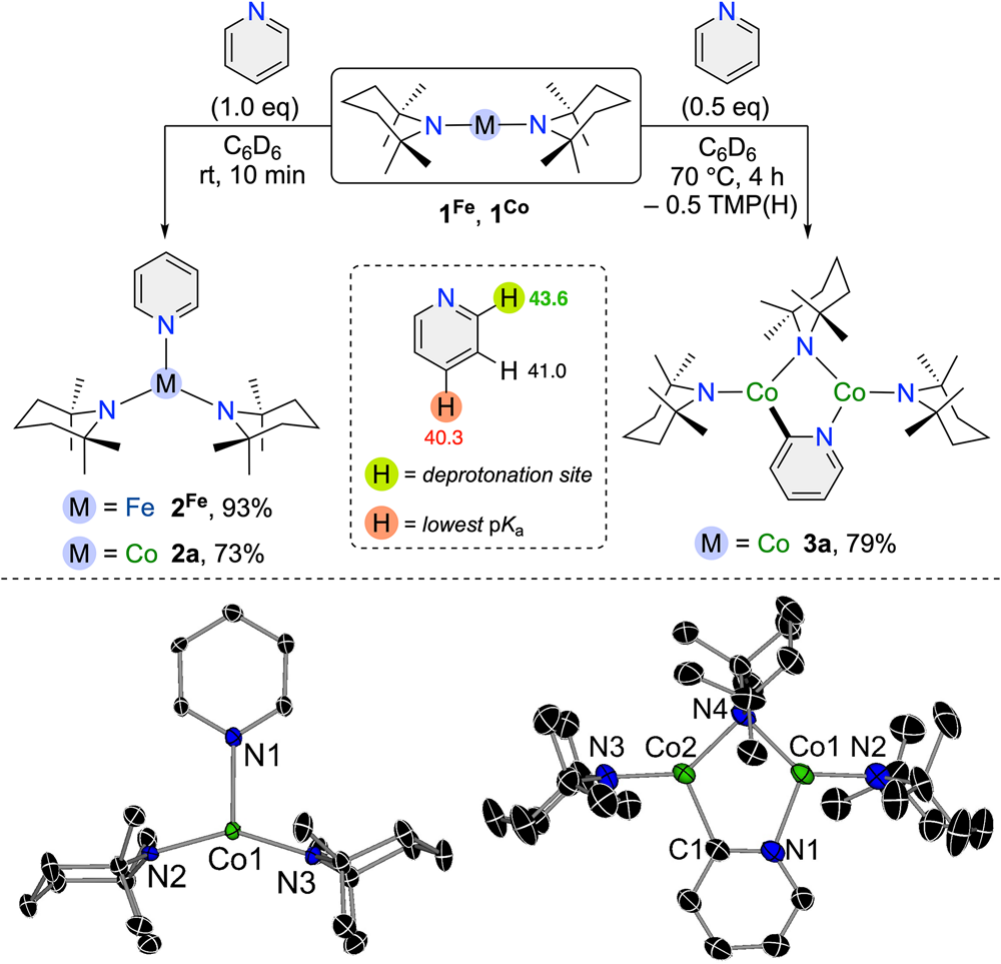
Reaction of **1^M^** (M = Fe, Co) with pyridine. Isolated crystalline yields. Molecular structures of **2a** (left) and **3a** (right). H atoms are omitted for clarity and displacement ellipsoids are displayed at 50% probability level. Selected bond lengths (Å) and angles (°) in **2a**: Co1N1 2.095(4), Co1–N2 1.913(7), Co1–N3 1.890(7); N2–Co1–N3 147.2(2); in **3a**: Co1–N1 2.026(2), Co2–C1 2.033(3), Co1–Co2 2.8830(5); Co1–N4–Co2 88.90(8).

We began our investigation using pyridine (py) as the model substrate for metalation. Relative to polyfluoroarenes, pyridine is poorly activated toward C─H metalation, in line with the higher p*K*_a_ values of its aromatic protons (C2 = 43.6; C3  = 41.0; C4 = 40.3 in DMSO versus 29.0 for C_6_H_5_F) [26]. Consistent with this, ^1^H NMR monitoring of equimolar reactions of M(TMP)_2_ (**1^M^**) (M = Fe, Co) and pyridine in C_6_D_6_ at room temperature showed paramagnetically shifted pyridine resonances (31.54 to −16.74 ppm for Fe and 24.18 to −10.07 ppm for Co), indicating quantitative formation of pyridine coordination adducts [(py)Fe(TMP)_2_] (**2^Fe^**) and [(py)Co(TMP)_2_] (**2a**). The absence of TMP(H) under these conditions supported the conclusion that no C─H metalation takes place at ambient temperature. Rerunning the reactions in pentane, **2^Fe^** and **2a** could be isolated as crystalline solids in respective 93% and 73% yields (Figure 2). X‐ray crystallography confirmed that both complexes are monomeric in the solid state. Although severe twinning prevented a detailed discussion of bond parameters for **2^Fe^**, the presence of an Fe─N_py_ bond could still be unambiguously established (Figure S52).

Structurally, **2a** features a trigonal planar Co(II) centre made up of two TMP ligands and one pyridine. The sum of the N─Co─N angles is 360(4)° ranging from 105.5(4) to 147.2(2)°. The Co─N_py_ distance of 2.095(4) Å lies at the upper limit of reported values for monopyridine adducts of Co(II) bis(amides) [2.055(5) Å in [(py)Co{N(SiMe_3_)_2_}_2_] and 2.091(3) Å in [(py)Co{N(SiMe_3_)(Dipp)}_2_] (Dipp = 2,6‐diisopropylphenyl)], which is consistent with the steric demand of the TMP groups [27, 28]. In C_6_D_6_ solution at 300 K, **2^Fe^** and **2a** exhibit magnetic moments of 5.18 and 4.06 *μ*_B_ (Evans method), respectively, values that agree well with other trigonal planar high‐spin Fe(II) and Co(II) complexes [25, 29, 30, 31].

Heating the reaction mixtures to 70°C for four hours led to divergent outcomes for Fe and Co. For **1^Co^**, the solution colour changed from yellow‐green to dark orange, and ^1^H NMR monitoring showed conversion of **2a** into a new paramagnetic species accompanied by the appearance of TMP(H), suggesting successful C─H metalation (Figure S36). Slow evaporation of the solvent at room temperature afforded dark‐coloured crystals suitable for X‐ray crystallography, which were identified as the C2‐metalated pyridine complex [(2‐pyridinyl)Co_2_(TMP)_3_] (**3a**) (Figure 2).

In **3a**, the two Co(II) centres are bridged by one TMP ligand and one C2‐pyridinyl anion. The pyridinyl fragment binds via carbon to Co2 [2.033(3) Å] and via nitrogen to Co1 [2.026(2) Å], thereby forming a five‐membered {CoNCoNC} ring. Each cobalt centre also binds to a terminal TMP ligand to complete a distorted trigonal environment. From a structural viewpoint, **3a** can be regarded as the metalation product [(2‐pyridinyl)Co(TMP)] coordinated to unreacted **1^Co^** or, equivalently, as a cationic dinuclear assembly that stabilises the anionic pyridinyl fragment.

Evidence that the dinuclear structure persists in solution was obtained by ^1^H NMR spectroscopy (see Supporting Information for details). The spectrum of **3a** displays sharp aromatic singlet resonances at 86.97, 66.56, 54.81, and 54.39 ppm, together with three distinct sets of signals for the TMP ligands. The well‐resolved features, despite the paramagnetic nature of the complex, indicate that the solid‐state structure is retained in solution. Consistent with this picture, the magnetic moment of **3a** measured at 300 K is 3.75 *μ*_B_, which is substantially lower than the spin‐only value of 5.48 *μ*_B_ expected for two uncoupled high‐spin Co(II) centres (*S*  = 3/2) [29, 30, 31]. Although this value is higher than those typically observed for strongly antiferromagnetically coupled Co(II) systems (1.95 *μ*_B_ at room temperature) [25], it nevertheless indicates the presence of partial magnetic coupling between the two cobalt centres. This interpretation is supported by their close spatial proximity in the solid‐state structure [Co1···Co2 = 2.8830(5) Å] (Figure 2).

Remarkably, **3a** is a thermally robust pyridine‐metalated cobalt complex, in marked contrast to the highly fragile metalated pyridine intermediates typically witnessed with s‐block bases. Repeating the metalation of **1^Co^** with 0.5 equivalents of pyridine gave **3a** cleanly and quantitatively according to ^1^H NMR monitoring, and the complex could be isolated in 79% yield (Figure 2, see Supporting Information for details). Notably, this reactivity is unique to cobalt, as **1^Fe^** showed no evidence of pyridine metalation under identical conditions but instead decomposed over sixteen hours at 70°C (Figure S39).

These observations establish that **1^Co^** functions as a base capable of direct Co(II)–H exchange with pyridine, a substrate that is significantly less activated in terms of p*K*_a_ than fluorobenzenes. This reaction proceeds selectively at the least acidic C─H position, in contrast with the behaviour of s‐block reagents such as the Lochman–Schlosser superbase or *n*BuNa [13, 16], since these typically metalate more acidic sites (Figure 1). Although C2‐lithiation of pyridine can be accomplished using *n*BuLi.LiDMAE, that protocol requires strict temperature control and immediate trapping at −78°C to prevent decomposition of the organolithium intermediate [15].

While **3a** forms through a direct Co(II)–H exchange, it is helpful to place this result within the broader landscape of C2‐functionalisation of pyridines. Transition metal‐mediated C─H activation at C2 has been reported in both catalytic and stoichiometric regimes, taking place typically with a change in the oxidation state of the metal [32, 33, 34, 35, 36]. For instance, Chirik and co‐workers reported a Co(I) complex supported by a tridentate PNP pincer ligand that promotes catalytic C2‐borylation via a Co(I)/Co(III) redox manifold [34]. In addition, several stoichiometric C2‐selective activations of pyridines have been reported which rely on bimetallic cooperation [37, 38, 39, 40]. This includes an iron–aluminylene system reported by Crimmin and co‐workers, which engages in reductive deprotonation of pyridine operating as a frustrated Lewis pair [38].

Because **3a** retains three TMP ligands, we next examined whether any of them remained sufficiently basic to metalate a second equivalent of pyridine. However, neither prolonged heating at 70°C for 16 h nor the addition of LiCl or *n*Bu_4_NCl [25], promoted further reactivity, and **3a** was recovered unchanged in all cases. These experiments suggested that cobaltation terminates after formation of the dinuclear complex since its steric surrounds prevent access to the cobalt centres.

**FIGURE 3:**
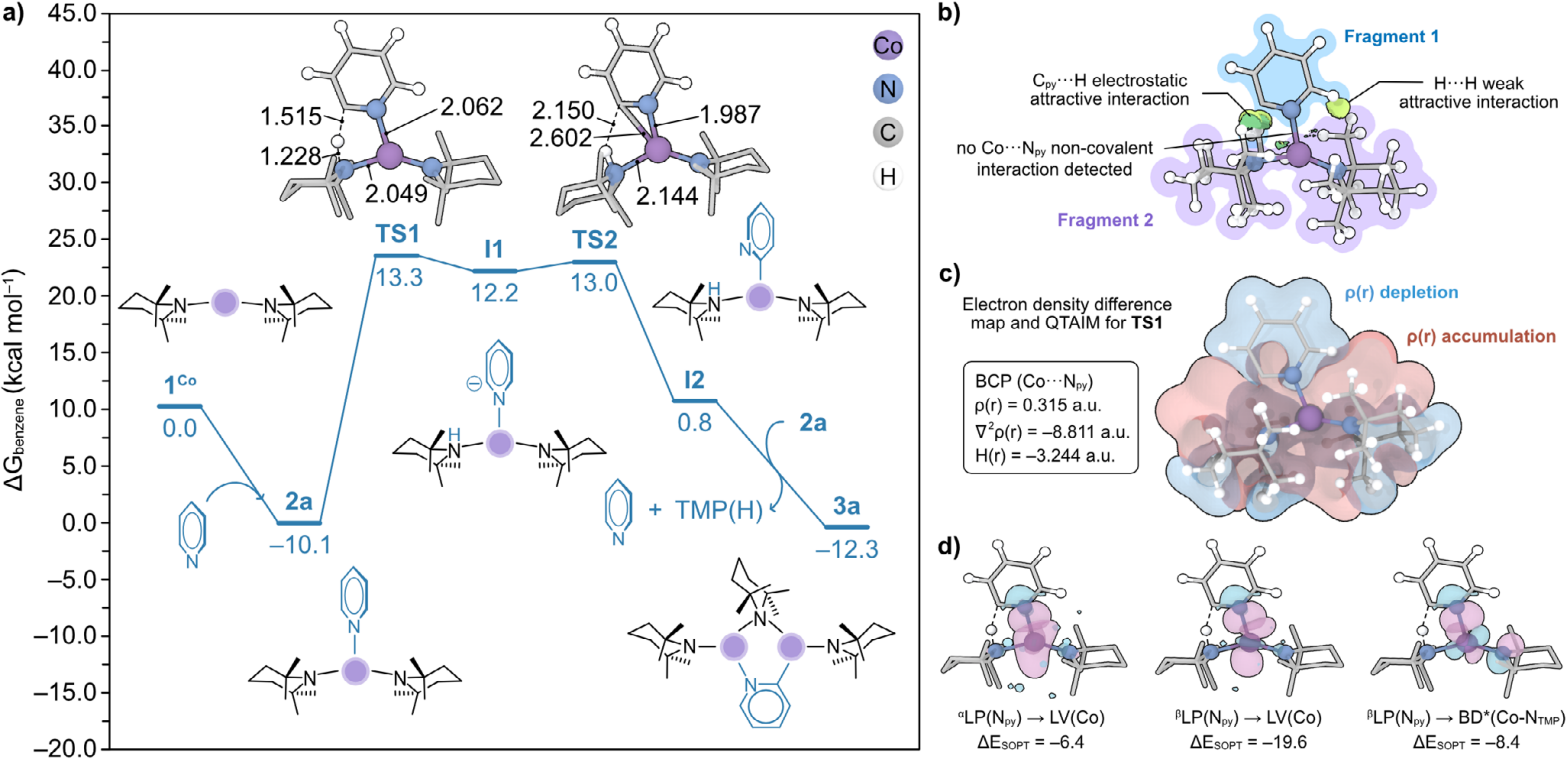
a) Gibbs energy profile for the C─H metalation of pyridine by **1^Co^** at 298 K and 1 atm. Optimised structures of the transition states are shown as insets, with relevant bond lengths (Å); some H atoms are omitted for clarity. b) Non‐covalent interaction (NCI) analysis (isovalue = 0.50 a.u.) of **TS1**, highlighting attractive interactions between the pyridine and Co(TMP)_2_ fragments. c) Quantum theory of atoms in molecules (QTAIM) parameters and 3D electron density difference map (isovalue = 0.80 a.u.) between the pyridine and Co(TMP)_2_ fragments in **TS1**. Electron density accumulation (red) between Co···N_py_ indicates a covalent bond, with electron density flowing from the N_py_ atom to the Co center. d) Natural bond orbital (NBO) analysis of **TS1**, depicting the main orbital interactions between pyridine and Co(TMP)_2_ fragments as isosurfaces (isovalue = 0.05 a.u.). Key interaction energies for the Co···N_py_ bond, calculated using second‐order perturbation theory (ΔE_SOPT_), are reported in kcal mol^−1^. Further details on the software used for the analyses in panels b)–d) can be found in the Supporting Information.

To rationalise the unusual reactivity and the regioselectivity of **1^Co^** toward pyridine, as well as the stability of **3a**, we turned to DFT calculations at the ωB97XD level (see Supporting Information for details). As shown in Figure 3a, pyridine initially coordinates to **1^Co^** to give the stable adduct **2a** (−10.1 kcal mol^−1^), and this association facilitates the subsequent Co(II)–H exchange via transition state **TS1** with a barrier of +23.4 kcal mol^−1^. Alternative pathways involving metalation at the *meta* or *para* positions were also examined and were significantly higher in energy (Figure S76). The intermediate that follows, **I1**, displays a Co─N_py_ bond rather than the Co─C_py_ connectivity characteristic of the metalated product. This feature highlights the strong cobalt–nitrogen interaction that stabilises transition state **TS1** and enables selective deprotonation at the least acidic C─H site. This mechanistic picture is reminiscent of our earlier work on pentafluorobenzene, where substrate pre‐coordination was identified as an important contributor to metalation and the Co–substrate interactions were primarily non‐covalent [25]. In the present system, however, non‐covalent interaction (NCI) analysis of **TS1** shows no sign of non‐covalent Co···N_py_ contacts, which is consistent with the formation of a covalent C─N bond (Figure 3b). Quantum theory of atoms in molecules (QTAIM) analysis further reinforces this interpretation, revealing a bond critical point (BCP) with relatively high electron density [ρ(r) = 0.315 a.u.], a negative Laplacian [∇^2^ρ(r) = −8.811 a.u.], and negative electronic energy density [H(r) = −3.244 a.u.], all characteristic of covalent bonding (Figure 3c). Complementary electron density difference maps show depletion at N_py_ and accumulation around Co upon interaction, along with charge buildup at the formed carbanion.

Natural bond orbital (NBO) analysis provides additional insight into this interaction (Figure 3d). Two principal donor‐acceptor interactions correspond to donation from the N_py_ lone pair into distinct Co valence orbitals and together stabilise the system by −26.0 kcal mol^−1^. A third interaction involves donation from the nitrogen lone pair into Co─N_TMP_ antibonding orbitals (−8.4 kcal mol^−1^), suggesting that weakening of both Co─N_TMP_ bonds precedes **TS1** and helps facilitate deprotonation. These combined observations rationalise how TMP ligands can activate N‐containing arenes despite their comparatively higher *α*‐C─H p*K*_a_ values and explain the regioselectivity observed experimentally in the formation of **3a**.

To probe this mechanistic interpretation experimentally, we evaluated the reactivity of **1^Co^‐IMes** (IMes = 1,3‐bis(2,4,6‐trimethylphenyl)imidazol‐2‐ylidene), which contains a non‐labile NHC ligand coordinated to the Co(II) centre. In this case, no pyridine metalation was observed, even under forcing conditions (70°C, 16 h; see Supporting Information for details), a result consistent with the inability of pyridine to displace IMes and form adduct **2a**.

From **I1**, the reaction proceeds through the low‐barrier transition state **TS2** (+0.8 kcal mol^−1^) to give the C‐bound isomer **I2**, which then undergoes co‐complexation with unreacted **2^Co^** to form the dinuclear product **3a** together with TMP(H) (Figure 3a). This step is highly exergonic (–12.3 kcal mol^−1^) and drives the overall reaction forward. We also evaluated a possible alternative pathway in which **I2** dimerises with elimination of two equivalents of TMP(H) to generate a putative [{(2‐pyridinyl)Co(TMP)}_2_] species analogous to those proposed in fluoroarene metalation. However, this pathway is endergonic by +15.7 kcal mol^−1^ and therefore was not considered further (Figure S77).

Taken together, these findings show that coordination of the Co(II) centre to the pyridine nitrogen has a dual role: it controls regioselectivity at **TS1** and stabilises the metalated intermediate **I2** through co‐complexation with **2^Co^** and elimination of TMP(H), thereby preventing the reversal of the cobaltation.

Finally, to understand why Fe(TMP)_2_ does not metalate pyridine, we performed analogous calculations for the iron system. The sequence of intermediates (**TS1^Fe^**, **I1^Fe^**, **TS2^Fe^** and **I2^Fe^**) resembles that calculated for cobalt (Figures S78 and S79), yet the subsequent co‐complexation step differs markedly. Formation of a putative [(2‐pyridinyl)Fe_2_(TMP)_3_] complex (**3a^Fe^**, Figure S79) does not provide sufficient stabilisation to render the process thermodynamically favourable (+2.1 kcal mol^−1^). This distinction accounts for the lack of pyridine metalation observed experimentally with Fe(TMP)_2_ and highlights the critical influence of the greater Lewis acidity of Co(II) relative to Fe(II) in enabling this low‐polarity metalation.

### 2.2 Extending Co(II)–H Exchanges to Other *N*‐Heterocyclic Substrates

**FIGURE 4:**
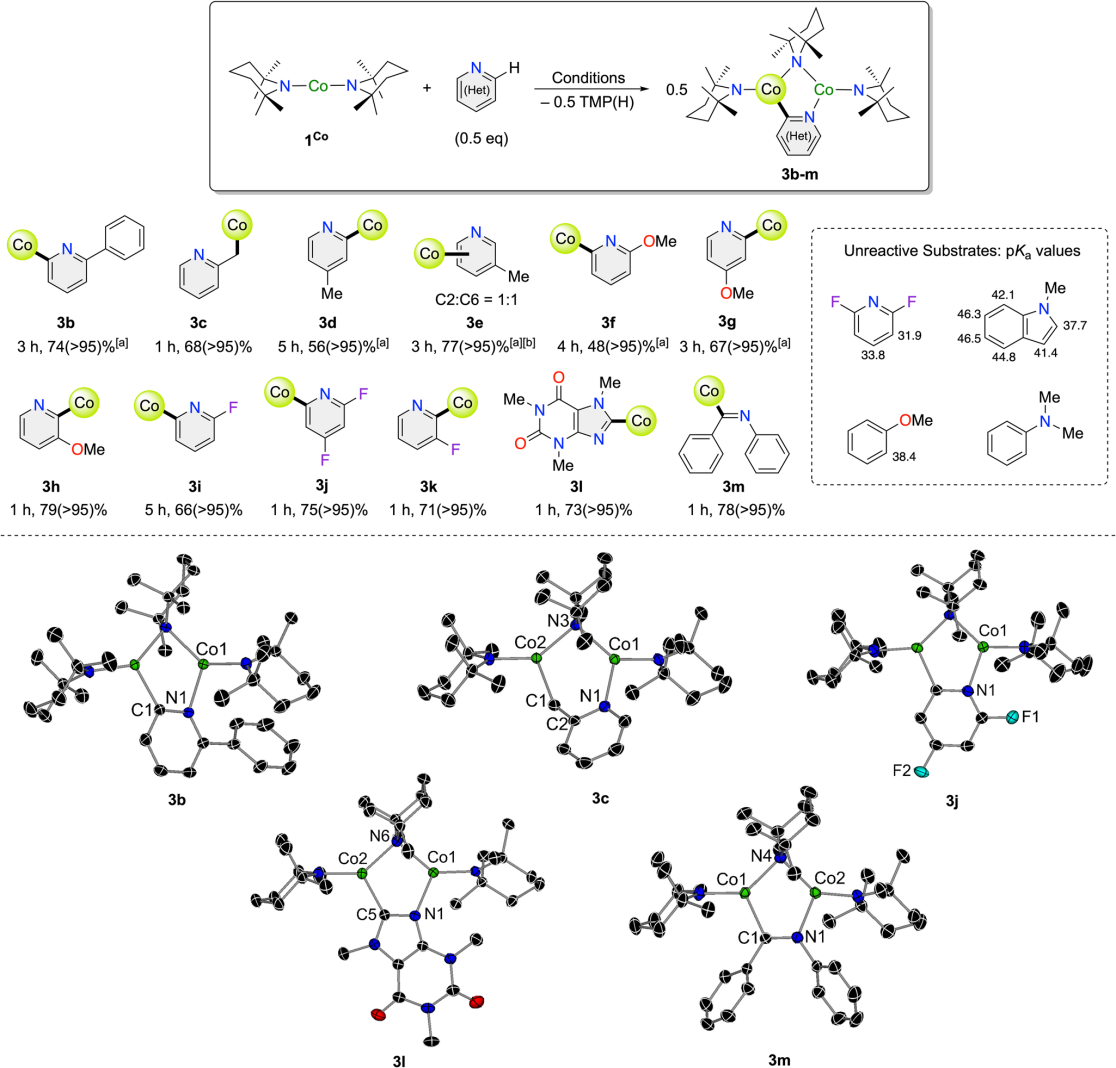
Substrate scope of cobaltation with **1^Co^**. Reaction conditions: **1^Co^** (0.2 mmol), substrate (0.1 mmol), pentane (2 mL), rt. Isolated crystalline yields are shown; conversions by ^1^H NMR spectroscopy are given in parentheses. [a] Benzene as solvent, 70°C. [b] 1:1 mixture of C2‐ and C6‐metalated products determined by ^1^H NMR. Inset: Molecular structures of selected complexes **3b**, **3c**, **3j**, **3l**, and **3m**. H atoms omitted for clarity and displacement ellipsoids displayed at 50% probability level.

We next explored the scope of this cobaltation method, focusing on *N*‐heterocycles (Figure 4). Reactions were carried out in pentane, using **1^Co^** and the relevant substrate in a 2:1 ratio, furnishing the cobaltation products **3b‐m**, which were subsequently isolated and characterised spectroscopically.

Their solid‐state structures were established by X‐ray crystallography (see Supporting Information for details). Isolated crystalline yields are shown in Figure 4, with ^1^H NMR conversions given in parentheses. In all cases, dinuclear complexes with a similar structure to that discussed for **3a** were obtained, and cobaltation occurred at a C─H site located in close proximity to a nitrogen centre. Except for **3c** (*vide supra*), complexes **3b‐m** display the same dinuclear tris‐TMP five‐membered ring motif described for **3a** and exhibit similar structural parameters in most cases (see Supporting Information for details). The Co···Co distances in **3b**, **3d**, **3f‐k**, and **3m** range from 2.7349(2) to 2.9282(3) Å, and their solution magnetic moments lie between 3.43 and 3.98 *μ*_B_ in C_6_D_6_ at 300 K. These values are consistent with two antiferromagnetically coupled high‐spin Co(II) centres (*S* = 3/2). In contrast, longer Co···Co distances are observed in **3c** [3.1177(4) Å] and **3l** [3.0908(7) Å]; this is reflected in their higher solution magnetic moments [both 4.68 *μ*_B_ in C_6_D_6_ at 300 K by Evans method], which point to a reduced degree of antiferromagnetic coupling in these two complexes [29, 30, 31].

The substrate 2‐phenylpyridine was chosen because its metalation has been widely explored with main‐group bases. In particular, using the lithium alkyl/lithium alkoxide combination *n*BuLi.LiDMAE favours regioselective metalation at the C6‐position on the pyridine ring [41], whereas the Turbo Grignard base TMPMgCl.LiCl reported by Knochel and co‐workers promotes C2‐magnesiation on the phenyl ring, although in the latter case an excess of base (2.0 equivalents) and prolonged heating (55°C, 24 h) are required [42]. Under our conditions, treatment of 2‐phenylpyridine with **1^Co^** leads to quantitative Co─H exchange at the C6‐position of the pyridinyl ring after 16 h at room temperature (or after 3 h at 70°C), affording complex **3b** in 74% crystalline yield. X‐ray crystallography confirmed that **3b** adopts the same structural motif as **3a**, in which the pyridine nitrogen acts as a σ‐donor to a Co(TMP)_2_ unit (Figure 4). This suggests that formation of the bimetallic motif is better stabilised when metalation occurs on the pyridinyl ring rather than on the phenyl ring. Although this regioselectivity resembles that observed in organolithium chemistry (*vide supra*), it contrasts with the behaviour reported for many transition‐metal‐catalysed C─H functionalisation protocols of 2‐phenylpyridine [43, 44, 45, 46, 47, 48, 49, 50, 51], which typically activate the C2‐position of the phenyl ring and gain additional stabilisation through coordination with the pyridine nitrogen [52, 53, 54].

We then turned to methyl‐substituted pyridines. For 2‐picoline, cobaltation proceeds cleanly at the benzylic position at room temperature within one hour to give complex **3c** in 68% crystalline yield, and ^1^H NMR monitoring in C_6_D_6_ shows this transformation is quantitative. The molecular structure of **3c** is related to those of **3a** and **3b**; however, metalation at the methyl position generates a six‐membered {CoNCoNCC} ring, which results in a larger Co···Co distance [3.1177(4) Å vs 2.8830(5) Å in **3a**]. In addition, the Co─C bond in **3c** [2.0898(10) Å] is slightly longer than the Co─C bonds in **3a** and **3b** [2.033(3) and 2.0321(13) Å], consistent with its sp^3^ character. The electron density of the monoanionic picolyl fragment is delocalised across the azaallyl moiety, as evidenced by the increased double bond character of C1–C2 [1.4426(15) Å] compared to the corresponding bond in free 2‐picoline [1.507 Å] [55]. The observed regioselectivity, which targets the most acidic site of the molecule (p*K*_a_ = 34.5 in THF) [56], agrees with trends reported for main‐group metal bases [57, 58, 59, 60], while still allowing pyridine nitrogen coordination and formation of the dinuclear assembly via the Co1─N1 bond (Figure 4). Interestingly, for 4‐picoline, full conversion into the C2‐metalated product **3d** was achieved upon heating at 70°C for 5 h, and **3d** could be isolated as a crystalline solid in 56% yield. For 3‐picoline, which contains two non‐equivalent *α‐*C─H positions relative to the nitrogen, cobaltation produces **3e** in 77% yield as a 1:1 statistical mixture of the C2‐ and C6‐metalated products (see Supporting Information). For these substrates, preferential *α*‐metalation of the pyridine rings contrasts with the more commonly observed benzylic metalation when using alkali‐metal alkyls or amides [57].

We next investigated substituted fluoro‐ and methoxypyridines, which contain classical directing *ortho*‐metalation (D*o*M) groups. Remarkably, reaction of **1^Co^** with 2‐, 4‐, and 3‐methoxy pyridine leads to quantitative formation of complexes **3f**, **3g**, and **3h**, respectively, each arising from regioselective *α‐*deprotonation of the pyridine ring. The products were isolated in 48%, 67%, and 79% yields, respectively, with reactions for **3f** and **3g** requiring heating at 70°C. For 3‐methoxypyridine, which has two non‐equivalent *α* positions, cobaltation takes place exclusively at the site that is also *ortho* to the methoxy group.

Further insight into this behaviour was obtained from reaction monitoring. ^1^H NMR studies of **1^Co^** with one equivalent of 2‐methoxypyridine revealed initial formation of the coordination adduct [(2‐methoxy‐py)Co(TMP)_2_] (**2f**), followed by its conversion into the metalated **3f** upon heating. This sequence underscores the importance of preliminary Co─N coordination in these metalation processes (see Supporting Information). Overall, these results indicate that for 2‐methoxypyridine the *α*‐directing effect of the pyridine nitrogen overrides the classical *ortho*‐directing effect of the methoxy group. This outcome contrasts sharply with zincation of similar substrates using bimetallic bases [MZn(TMP)R_2_] (M = Li, K; R = *t*Bu, Et), where selective *ortho‐*zincation at the OMe group is observed [61, 62]. In a related case, Mongin and coworkers examined the metalation of 2‐methoxypyridine with an in situ generated LiFe(TMP)_3_ mixture, which after quenching with iodine afforded 3‐iodo‐2‐methoxypyridine and 2,2′‐dimethoxy‐3,3′‐bipyridine, outcomes that are again consistent with favoured *ortho* metalation [63].

On moving to fluoropyridine substrates, the same *α‐*to‐nitrogen regioselectivity was observed, affording complexes **3i**, **3j**, and **3k** in 66%, 75%, and 71% isolated yields, respectively. These results are particularly striking because fluorine exerts a strong inductive effect and typically acidifies *ortho* C─H positions in D*o*M chemistry. For example, in 2‐fluoropyridine the p*K*_a_ value of the C─H bond *ortho* to the C─F unit is 34.0, whereas the C─H bond *α* to the pyridine nitrogen has a p*K*_a_ of 40.7 [64]. Accordingly, s‐block metal bases such as the Lochmann/Schlosser superbase, alkali‐metal magnesiates and zincates metalate this substrate at C3 [64, 65], in clear contrast to the unique C6‐regioselectivity observed with **1^Co^**. This divergence is especially notable in **3j**, where the C─H position doubly activated by two fluorine substituents remains untouched.

Adding significance, the cobaltation strategy was found to extend beyond simple pyridines. For caffeine, **1^Co^** promotes C8‐metalation to afford **3l** in 73% crystalline yield. Reaction of **1^Co^** with *N*‐benzylideneaniline leads to selective *α*‐metalation at the imine CH═NPh unit, giving **3m** in 78% crystalline yield. This regioselectivity differs from that observed for LiTMP, which metalates ArCH═NCy aldimines (Cy = cyclohexyl) at the *ortho* position of the aryl group [66]. It is also worth noting that benzylideneaniline is known to undergo C─H activation at the same *ortho* position with various transition metal complexes, giving cyclometalated products [67, 68, 69].

Collectively, these results demonstrate the excellent regioselectivity of **1^Co^** toward *α*‐cobaltation of a broad range of pyridines and related N‐containing substrates under mild conditions. In line with the DFT studies on pyridine cobaltation, coordination of the substrate nitrogen to Co(II) appears to fix the regioselectivity of these reactions and, in many cases, overrides the intrinsic preferences implied by the p*K*_a_ values of the available C─H sites (see Supporting Information). This interpretation is further supported by the lack of reactivity of **1^Co^** toward 2,6‐difluoropyridine. In this substrate, the C─H bonds *ortho* to the C─F units are relatively acidic (p*K*_a_ = 31.9 in THF) [64], yet ^1^H NMR monitoring shows that 2,6‐difluoropyridine does not coordinate to **1^Co^** and is inert toward metalation. Similarly, no reaction occurs with 1‐methylindole, which lacks a coordinating nitrogen atom, or dimethylaniline and anisole, even though the *ortho* C─H bonds in anisole are considerably more activated that those in pyridine.

**FIGURE 5:**
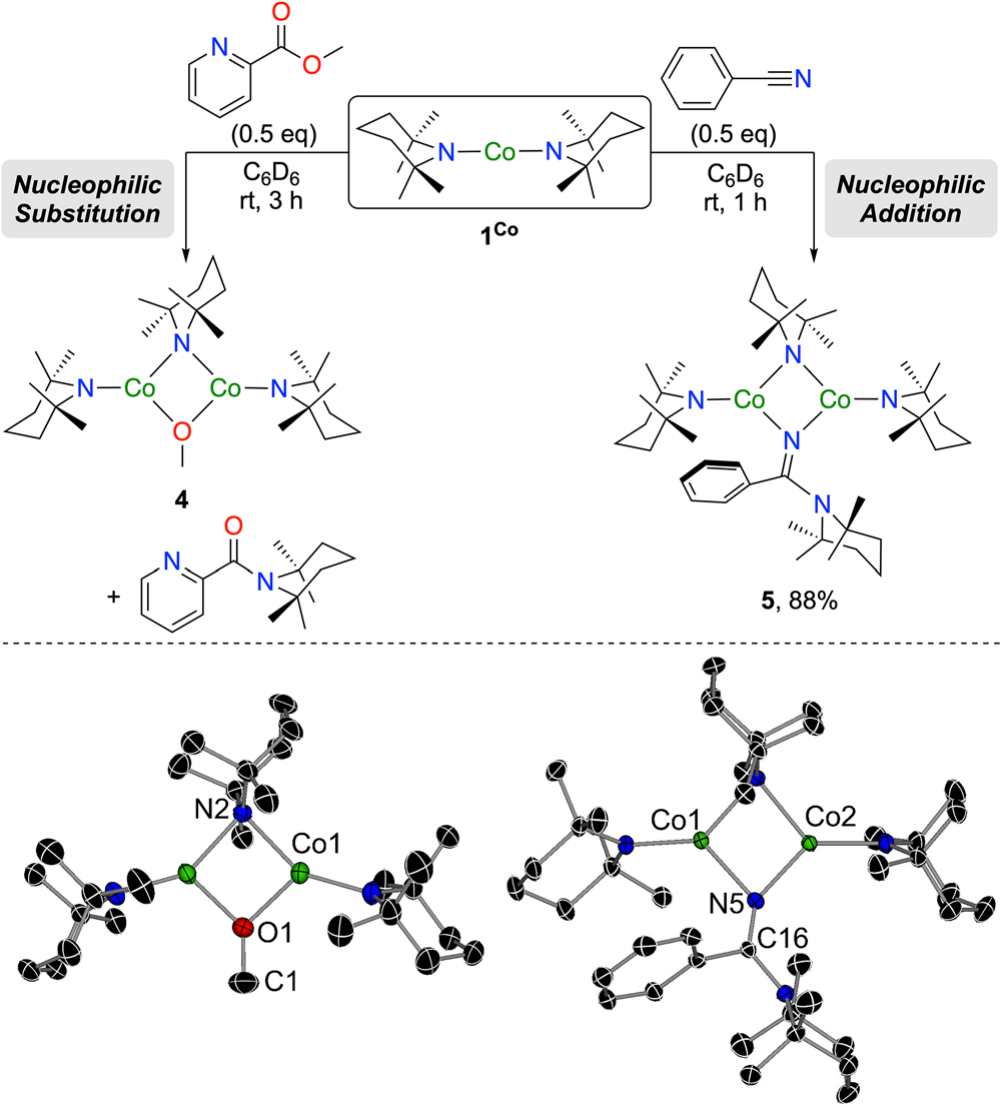
Reaction of **1^Co^** with methyl picolinate and benzonitrile. Isolated crystalline yields are shown. Molecular structures of **4** (left) and **5** (right). H atoms omitted for clarity and displacement ellipsoids displayed at 50% probability level. Selected bond lengths (Å) and angles (°) in **5**: Co1─Co2 2.6630(15); N5–C16 1.2787(10).

Interestingly, when **1^Co^** was treated with methylpicolinate, the complex reacted preferentially as a nucleophile rather than a base. In this case, nucleophilic substitution furnished pyridine‐2‐yl(2,2,6,6‐tetramethylpiperidin‐1‐yl)methanone together with the cobalt complex [(MeO)Co_2_(TMP)_3_] (**4**) (Figure 5). ^1^H NMR reaction monitoring showed that formation of these species is quantitative. In a similar way, reaction of **1^Co^** with benzonitrile affords the ketimide complex [{(Ph)(TMP)C═N}Co_2_(TMP)_3_] (**5**) in 88% crystalline yield as the product of nucleophilic addition across the C─N triple bond. These findings demonstrate that, despite the exceptional basicity of **1^Co^**, the TMP ligands bound to Co(II) retain significant nucleophilic character. In this context, we recently reported the ability of **1^Co^** to undergo double insertion of CO_2_ into its two Co─N_TMP_ bonds to form a bis(carbamate) species [25]. This nucleophilic behaviour contrasts with that of LiTMP, which acts as a base in metalating benzonitrile at −78°C to give a mixture of mono‐ and di‐*ortho*‐substituted products [70].

Isolated as crystalline products, compounds **4** and **5** were characterised by X‐ray crystallography (Figure 5 and Supporting Information). Both complexes display a dinuclear {Co_2_(TMP)_3_} structural core similar to those in **3a‐m**, where the two Co(II) centres connect by a methoxy O bridge in **4** or by the ketimido N═C(Ph)(TMP) N bridge in **5**.

### 2.3 Direct Cobaltation of Diazines and Diazanaphthalenes

We next evaluated the ability of **1^Co^** to promote the metalation of diazines. Although these molecules are important building blocks in pharmaceutical chemistry, their selective deprotonation with s‐block metal bases is particularly challenging [14, 20, 71, 72], especially for non‐substituted substrates that lack a directing group. Typically, strict temperature control is required and, even under cryogenic conditions, unwanted side reactions can occur because of the extreme fragility of the metalated intermediates. These issues can be partially mitigated by using cooperative bimetallic reagents that combine Li with a less electropositive metal such as Zn [18, 20, 61, 72, 73], Ga [19], or Cd [9]. Reaction of pyrazine (C_4_H_4_N_2_, pyz) with one equivalent of **1^Co^** in pentane at room temperature causes an immediate colour change from dark red to dark orange. Slow evaporation of the solvent at −30°C afforded dark orange crystals of [(2,5‐C_4_H_2_N_2_‐pyz)Co_4_(TMP)_6_] (**6a**) in 48% yield.

**FIGURE 6:**
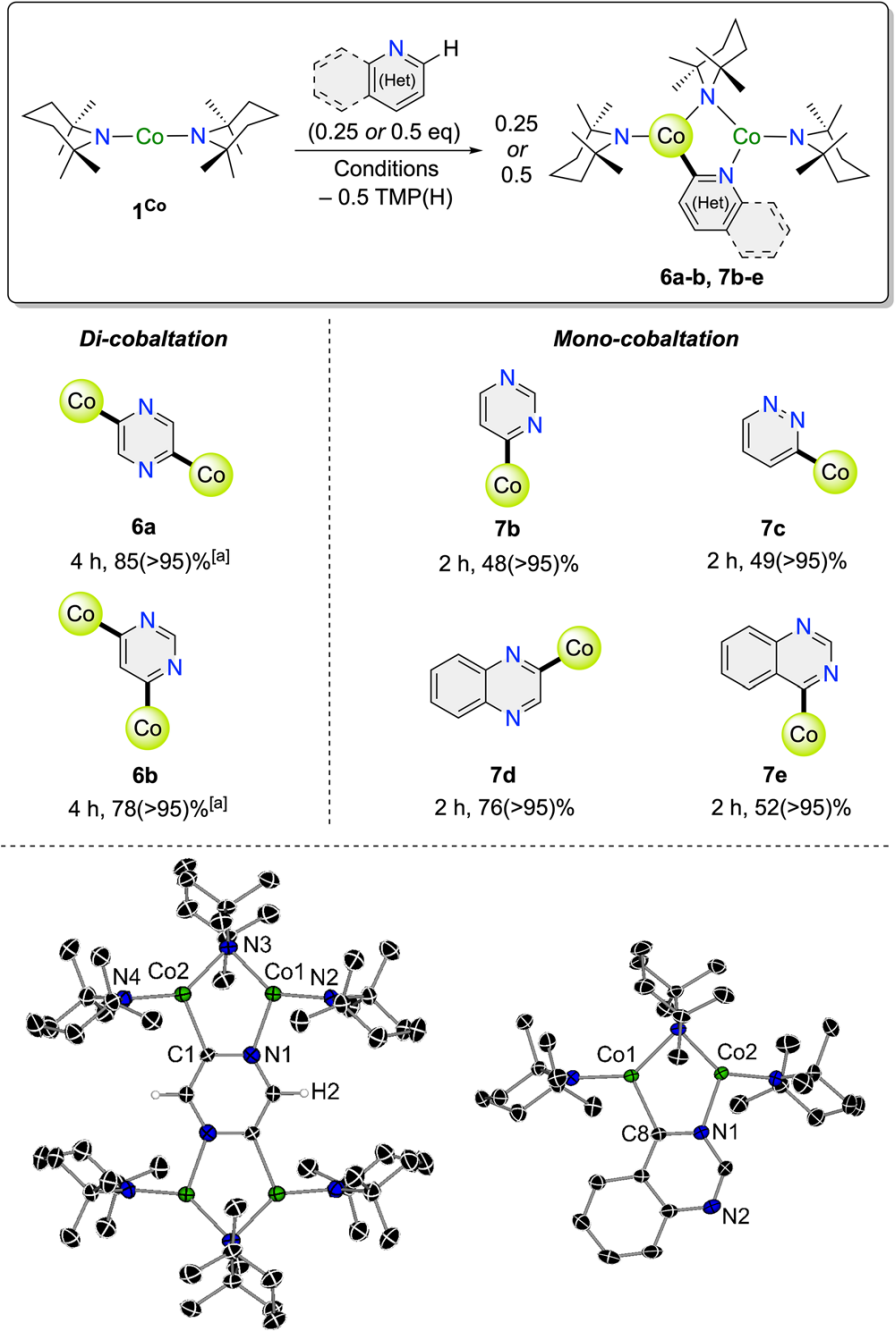
Substrate scope of (di)‐cobaltation with **1^Co^**. Reaction conditions: **1^Co^** (0.4 or 0.2 mmol), substrate (0.1 mmol), pentane (2 mL), rt. Isolated crystalline yields are shown; conversions by ^1^H NMR are given in parentheses. [a] Benzene as solvent, 70°C. Inset: Molecular structures of complexes **6a** and **7e**. H atoms omitted for clarity except those on the aromatic ring; displacement ellipsoids shown at 50% probability level.

X‐ray crystallographic analysis revealed that **6a** contains a two‐fold deprotonated pyrazine ring, substituted at the less sterically hindered 2,5‐positions by Co(II) atoms (Figure 6). The complex adopts a centrosymmetric tetranuclear motif in which each deprotonated carbon binds to one cobalt center [Co2–C1, 2.0145(14) Å], and the remaining two cobalt atoms coordinate to the nitrogen atoms of the heterocycle [Co1–N1, 2.0083(13) Å]. The two types of cobalt centres, Co1 and Co2, are linked by a bridging TMP ligand, and each completes a distorted trigonal planar coordination sphere with a terminal TMP ligand. This coordination arrangement resembles that found in **3a‐m** but here generates a distinctive fused three‐ring (5,6,5) framework. The tricyclic backbone in **6a** is nearly planar, with Co1 and the bridging N3 lying slightly out of the mean plane by 0.171 and 0.435 Å, respectively, which helps relieve steric congestion from the adjacent TMP ligands. Compound **6a** was also characterised by ^1^H NMR spectroscopy in C_6_D_6_, displaying a distinct resonance at 126.26 ppm for the remaining aromatic C─H protons of the dimetallated pyrazine ring, along with paramagnetically shifted signals for the TMP ligands spanning from 32.77 to −25.45 ppm. As far as we can ascertain, **6a** represents the first structurally characterised example of di‐cobaltation of an *N*‐heterocycle.

To improve the efficiency of the process, pyrazine was treated with four equivalents of **1^Co^** at 70°C for four hours, which allowed isolation of **6a** in 85% yield (Figure 6). Monitoring this reaction by ^1^H NMR spectroscopy showed that, at room temperature and using only two equivalents of **1^Co^**, an additional paramagnetic species was present in solution together with **6a**. This species was assigned to a C2‐monometalated intermediate (see Supporting Information for details).

The regioselectivity of this transformation is particularly notable. Although pyrazine can be 2,5‐dimetallated using superbasic bimetallic systems that combine LiTMP with zinc, gallium, or cadmium reagents, direct use of LiTMP alone is far less selective. At −78°C, a four‐fold excess of LiTMP gives mainly C2‐substituted pyrazine together with only small amounts (2%–16%) of C2,C5‐disubstituted products after electrophilic quench [14]. In contrast, cobaltation with **1^Co^** provides **6a** cleanly, indicating that **1^Co^** is not only more selective but also more efficient than classical LiTMP‐based protocols for this sensitive heterocycle.

**FIGURE 7:**
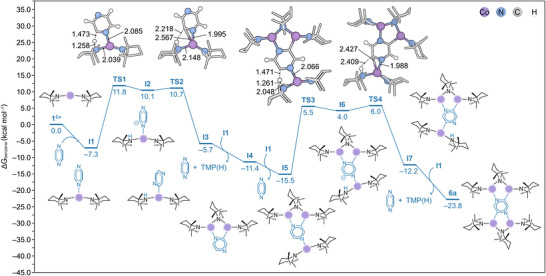
Gibbs energy profile computed for the first and second C─H metalations of pyrazine by **1^Co^** at 298 K and 1 atm. Optimized structures of the transition states are shown as insets, with relevant bond lengths (Å); some H atoms are omitted for clarity.

To try to rationalise this unusual reactivity, we performed additional DFT calculations for pyrazine. Coordination of pyrazine to **1^Co^** gave intermediate **I1** in an exergonic process (−7.3 kcal mol^−1^), identifying **I1** as the most stable species in solution (Figure 7). From this intermediate, C─H cobaltation proceeds via a pathway analogous to that described for pyridine (Figure 3). Co(II)–H exchange through **TS1** (∆G^‡^ = +19.1 kcal mol^−1^) forms **I2**, in which the deprotonated pyrazine remains bound through nitrogen. **I2** then undergoes a change in coordination via **TS2** (∆G^‡^ = +0.6 kcal mol^−1^), affording the mono‐C2‐cobaltated intermediate **I3**, formulated as [(TMPH)Co(C_4_H_3_N_2_‐pyz)(TMP)]. This rearrangement is highly exergonic (−15.8 kcal mol^−1^) and is followed by coordination of one equivalent of **I1** with displacement of TMP(H), which produces the di‐cobalt intermediate [(C_4_H_3_N_2_‐pyz)Co_2_(TMP)_3_], **I4** (−11.4 kcal mol^−1^) and pyrazine. Although **I4** could not be isolated, its formation was observed spectroscopically (*vide supra*).

Intermediate **I4** can then bind an additional equivalent of **I1** through its remaining nitrogen to give **I5** (−15.5 kcal mol^−1^). This coordination facilitates the second Co(II)–H exchange at C5. The second metalation proceeds via **TS3** and **TS4** to afford **I7**, which ultimately yields **6a** upon co‐complexation with another equivalent of **I1** and release of TMP(H) and pyrazin. Overall, formation of **6a** is exergonic by −23.8 kcal mol^−1^ with the rate‐limiting step being the second metalation (∆*G* ^‡^ = +21.5 kcal mol^−1^). Importantly, binding of **1^Co^** to **I4** to generate **I5** is thermodynamically favourable by −4.1 kcal mol^−1^, making **I5** the lowest‐energy intermediate on the reaction profile. This, together with the favourable energetics toward **6a**, explains why **6a** forms even when only two equivalents of **1^Co^** are used experimentally. The fact that the second metalation is rate‐limiting also accounts for the observation of a C2‐monometalated intermediate in solution alongside **6a**.

Having established that **1^Co^** can promote both mono‐ and di‐cobaltation of pyrazine, we next examined whether this reactivity could be extended to other diazines. The synthetic metalation approach also proves effective for pyrimidine (C_4_H_4_N_2_, pym), a substrate that cannot be metalated by LiTMP even under strictly controlled low‐temperature conditions [14]. Reacting pyrimidine with four equivalents of **1^Co^** at 70°C for 16 h furnished [(4,6‐C_4_H_2_N_2_‐pym)Co_4_(TMP)_6_] (**6b**) in 78% crystalline yield. X‐ray diffraction analysis showed that **6b** is isostructural with **6a** and features the same fused three‐ring (5,6,5) motif in which four cobalt atoms coordinate to the doubly deprotonated [C_4_H_2_N_2_]^2–^ dianion (Figure 6). Its paramagnetic ^1^H NMR spectrum in C_6_D_6_ displays two well‐resolved resonances at 132.68 and 126.21 ppm for the remaining C─H hydrogens of the dimetallated ring (see Supporting Information). To our knowledge, **6b** represents the first example of selective dimetallation of pyrimidine.

Notably, decreasing the loading of **1^Co^** to two equivalents and performing the reaction at room temperature led instead to the mono‐C4‐cobaltated product **7b**, which was isolated in 48% crystalline yield (>95% conversion by ^1^H NMR; see Supporting Information). Pyrimidine is another particularly challenging substrate in metalation chemistry. Knochel and co‐workers showed that TMPZnCl.LiCl promotes selective C2‐zincation through coordination‐controlled effects, whereas combinations of LiTMP with equimolar Ga(CH_2_SiMe_3_)_3_ or ZnCl_2_ instead furnish C4‐metalated products [18, 19, 20]. The behaviour of **1^Co^** therefore aligns with this latter trend while enabling direct cobaltation under comparatively mild conditions.

We then assessed the reactivity of **1^Co^** toward pyridazine (C_4_H_4_N_2_, pdz). Reaction at room temperature selectively produced [(3‐C_4_H_3_N_2_‐pdz)Co_2_(TMP)_3_] (**7c**), which was isolated in 49% crystalline yield and forms quantitatively according to ^1^H NMR monitoring (Figure 6). Neither altering the stoichiometry nor raising the temperature resulted in di‐cobaltation. This outcome can be attributed to the steric congestion imposed by the arching {Co_2_(TMP)_3_} fragment in **7c**, which appears to hinder binding of an additional equivalent of **1^Co^** to the remaining nitrogen atom.

Finally, we explored whether cobaltation would extend to more complex *N*‐heterocycles. Treatment of quinoxaline and quinazoline with **1^Co^** at room temperature for two hours gave complexes **7d** and **7e** in respective 76% and 52% crystalline yields, respectively, with metalation occurring selectively at C2 in quinoxaline and C4 in quinazoline (Figure 6). The structures of **7b‐e** were confirmed by X‐ray crystallographic analysis (see Supporting Information).

### 2.4 Applications in Oxidative C─N Coupling Reactions

**FIGURE 8:**
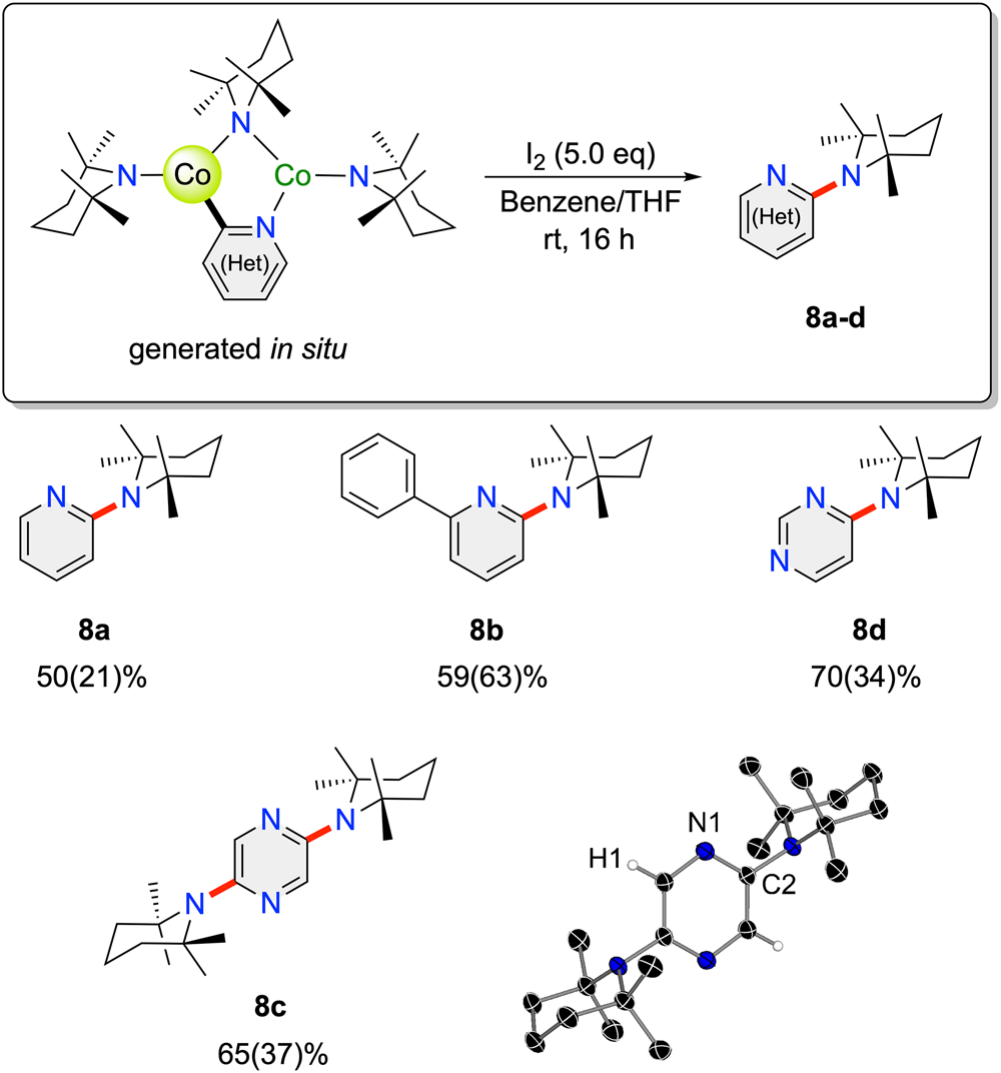
C─N coupling of selected (di)cobaltated species mediated by iodine. Yields were determined by ^1^H NMR using C_6_Me_6_ (10 mol%) as an internal standard; isolated yields are given in parentheses.

To explore how the cobaltated intermediates might be used in downstream transformations, we probed their reactivity under oxidative conditions. Reacting **3a** with I_2_ gave 2‐(2,2,6,6‐tetramethylpiperidin‐1‐yl)pyridine (**8a**) in 50% yield, arising from selective coupling of a TMP group with the C2‐pyridinyl fragment (Figure 8). Comparable results were obtained when *N*‐iodosuccinimide or 5‐(trifluoromethyl)dibenzothiophenium trifluoromethanesulfonate were used as oxidants, each providing **8a** in similar yields (see Supporting Information).

Encouraged by this initial result, we evaluated whether related cobaltated complexes would undergo analogous oxidative transformations. Indeed, complexes **3b**, **6a**, and **7b** delivered the corresponding TMP‐substituted products **8b**, **8c**, and **8d** in respective 59%, 65%, and 70% yields (Figure 8). Notably, **8c**, in which two TMP groups participate in C─N bond formation, could be isolated in crystalline form and structurally characterised by X‐ray diffraction.

These findings demonstrate that the Co(II) system not only activates heterocycles through an initial C─H metalation step but also enables subsequent oxidative C─N bond formation. The resulting amines **8b‐d** are new, and their sterically encumbered TMP substituents make them difficult to access by conventional C─N cross‐coupling methods such as Buchwald–Hartwig reactions. In this regard, the cobaltation–oxidation sequence provides a complementary strategy for assembling highly hindered C─N bonds from simple *N*‐heterocyclic precursors.

## 3 Conclusions

This study has revealed the extraordinary power of the homoleptic cobalt(II) bis‐amide Co(TMP)_2_ to promote the regioselective metalation of a broad range of *N*‐heterocycles via direct Co(II)–H exchange, including pyridine and highly sensitive diazines. These substrates are notoriously difficult to metalate using conventional lithium‐ or magnesium‐based reagents. While cobalt complexes have previously been reported in C─H activation chemistry of heteroarenes, such processes typically proceed through changes in the oxidation state of the metal. In contrast, Co(TMP)_2_ operates in redox‐neutral processes displaying a reactivity profile that closely parallels that of main‐group metal amides, while simultaneously affording access to thermally robust organocobalt intermediates.

By combining detailed experimental and computational studies, we elucidate the mechanistic origins of this reactivity, and particularly the decisive role played by coordination effects. Initial binding of the *N*‐heterocycle to the cobalt centre not only enables Co–H exchange but also dictates the regioselectivity of such metalations, consistently favouring C─H sites *α* to nitrogen. Subsequent stabilisation of the metalated intermediate through co‐complexation with a second equivalent of Co(TMP)_2_ renders the cobaltation thermodynamically favourable and effectively suppresses reversibility. This coordination‐driven activation leads to regioselectivities that override trends predicted solely by C─H acidity (p*K*_a_ values). Notably, even weakly acidic substrates such as pyrazine and pyrimidine undergo controlled two‐fold di‐cobaltation when stoichiometry and temperature are judiciously adjusted.

Finally, the redox flexibility of cobalt can be exploited to transform these organometallic intermediates into valuable organic products. Exposure of the metalated species to external oxidants such as iodine triggers selective C─N bond formation, enabling coupling of the heteroaryl fragment with a sterically demanding TMP group.

Overall, this work firmly establishes Co(TMP)_2_ as a versatile and mechanistically distinct reagent for the selective functionalisation of *N*‐heterocycles. In bridging concepts from main‐group deprotonative metalation and transition metal coordination chemistry, this non‐precious transition metal reagent opens up new avenues for the controlled activation and derivatisation of challenging heteroaromatic substrates.

## Author Contributions

**Na Jin**: investigation, writing – original draft, formal analysis, data curation. **Manting Mu**: investigation, writing – original draft, formal analysis. **Max García‐Melchor**: conceptualisation, writing – review and editing, funding acquisition, project administration, supervision. **Eva Hevia**: conceptualisation, writing – review and editing, funding acquisition, project administration, supervision.

## Funding

HORIZON‐Europe, Research Ireland, and Schweizerischer Nationalfonds zur Förderung der Wissenschaftlichen Forschung.

## Conflicts of Interest

The authors declare no conflict of interest.

## Supporting information




**Supporting File**: anie73155‐sup‐0001‐SuppMat.pdf.

## Data Availability

The data that support the findings of this study are openly available in BORIS at https://boris‐portal.unibe.ch/handle/20.500.12422/230779. All the DFT data underlying this work, including the cartesian coordinates and energies of all the modelled structures, is openly accessible via the following ioChem‐BD online dataset: doi.org/10.19061/iochem‐bd‐6‐618.

## References

[1] P. Singh , Recent Developments in the Synthesis and Applications of Pyridines (Elsevier, 2022), 1–628.

[2] S. De , A. Kumar , S. K. Shah , et al., “Pyridine: The Scaffolds With Significant Clinical Diversity,” RSC Advances 12 (2022): 15385–15406, 10.1039/D2RA01571D. 35693235 PMC9121228

[3] B. Islam , I. Islam , N. Nath , et al., “Recent Advances in Pyridine Scaffold: Focus on Chemistry, Synthesis, and Antibacterial Activities,” BioMed Research International 2023 (2023): 9967591, 10.1155/2023/9967591. 37250749 PMC10212683

[4] E. Vitaku , D. T. Smith , and J. T. Njardarson , “Analysis of the Structural Diversity, Substitution Patterns, and Frequency of Nitrogen Heterocycles Among U.S. FDA Approved Pharmaceuticals,” Journal of Medicinal Chemistry 57 (2014): 10257–10274, 10.1021/jm501100b. 25255204

[5] K. A. Rinderspacher , Progress in Heterocyclic Chemistry (Elsevier, 2014), 395–447.

[6] P. Corona , A. Carta , M. Loriga , G. Vitale , and G. Paglietti , “Synthesis and in Vitro Antitumor Activity of New Quinoxaline Derivatives,” European Journal of Medicinal Chemistry 44 (2009): 1579–1591, 10.1016/j.ejmech.2008.07.025. 18774202

[7] S. Achelle , C. Baudequin , and N. Plé , “Luminescent Materials Incorporating Pyrazine or Quinoxaline Moieties,” Dyes and Pigments 98 (2013): 575–600, 10.1016/j.dyepig.2013.03.030.

[8] E. V. Verbitskiy , G. N. Lipunova , E. V. Nosova , and V. N. Charushin , “Advances in the Design of Functionalized 1,4‐Diazines as Components for Photo‐ or/and Electroactive Materials,” Dyes and Pigments 220 (2023): 111763, 10.1016/j.dyepig.2023.111763.

[9] F. Chevallier and F. Mongin , “Functionalization of Diazines and Benzo Derivatives Through Deprotonated Intermediates,” Chemical Society Reviews 37 (2008): 595–609, 10.1039/B709416G. 18224265

[10] A. Turck , “Advances in the Directed Metallation of Azines and Diazines (Pyridines, Pyrimidines, Pyrazines, Pyridazines, Quinolines, Benzodiazines and Carbolines). Part 2: Metallation of Pyrimidines, Pyrazines, Pyridazines and Benzodiazines,” Tetrahedron 57 (2001): 4489–4505, 10.1016/S0040-4020(01)00225-3.

[11] F. Mongin and G. Quéguiner , “Advances in the Directed Metallation of Azines and Diazines (Pyridines, Pyrimidines, Pyrazines, Pyridazines, Quinolines, Benzodiazines and Carbolines). Part 1: Metallation of Pyridines, Quinolines and Carbolines,” Tetrahedron 57 (2001): 4059–4090, 10.1016/S0040-4020(01)00100-4.

[12] M. Schlosser and F. Mongin , “Pyridine Elaboration Through Organometallic Intermediates: Regiochemical Control and Completeness,” Chemical Society Reviews 36 (2007): 1161–1172, 10.1039/b706241a. 17576483

[13] J. Verbeek , A. V. E. George , R. L. P. De Jong , and L. Brandsma , “Functionalization of Pyridine via Direct Metallation,” Journal of the Chemical Society, Chemical Communications (1984): 257–258, 10.1039/c39840000257.

[14] N. Plé , A. Turck , K. Couture , and G. Quéguiner , “Diazines 13: Metalation Without Ortho‐Directing Group—Functionalization of Diazines via Direct Metalation,” Journal of Organic Chemistry 60 (1995): 3781–3786, 10.1021/jo00117a033.

[15] P. Gros , Y. Fort , and P. Caubère , “Aggregative Activation in Heterocyclic Chemistry. Part 5.† Lithiation of Pyridine and Quinoline With the Complex Base BuLi·Me_2_N(CH_2_)_2_OLi (BuLi·LiDMAE),” Journal of the Chemical Society, Perkin Transactions 1 24 (1997): 3597–3600, 10.1039/a705027e.

[16] H. H. Hsu , S. Kang , C. C. Chen , R. Sk , and A. A. Thomas , “Functionalization of Pyridines at the C4 Position via Metalation and Capture,” Angewandte Chemie 137 (2025): e202424172, 10.1002/ange.202424172. PMC11933531 39752240

[17] L. Davin , R. McLellan , A. Hernán‐Gómez , et al., “Regioselective Magnesiation of N‐Heterocyclic Molecules: Securing Insecure Cyclic Anions by a β‐Diketiminate‐Magnesium Clamp,” Chemical Communications 53 (2017): 3653–3656, 10.1039/C6CC09675A. 28101539

[18] A. Kremsmair , A. S. Sunagatullina , L. J. Bole , et al., “Exploiting Coordination Effects for the Regioselective Zincation of Diazines Using TMPZnX·LiX (X=Cl, Br),” Angewandte Chemie 134 (2022): e202210491, 10.1002/anie.202210491. PMC9826189 35943036

[19] M. Uzelac , A. R. lan Kennedy , E. Hevia , et al., “Transforming LiTMP Lithiation of Challenging Diazines Through Gallium Alkyl Trans‐Metal‐Trapping,” Angewandte Chemie 128 (2016): 13341–13344, 10.1002/ange.201607284. PMC5113679 27647741

[20] A. Seggio , F. Chevallier , M. Vaultier , and F. Mongin , “Lithium‐Mediated Zincation of Pyrazine, Pyridazine, Pyrimidine, and Quinoxaline,” Journal of Organic Chemistry 72 (2007): 6602–6605, 10.1021/jo0708341. 17655361

[21] Y. Kondo , M. Shilai , M. Uchiyama , and T. Sakamoto , “TMP−Zincate as Highly Chemoselective Base for Directed Ortho Metalation,” Journal of the American Chemical Society 121 (1999): 3539–3540, 10.1021/ja984263t.

[22] R. E. Mulvey , F. Mongin , M. Uchiyama , and Y. Kondo , “Deprotonierende Metallierungen mit 'at‐Verbindungen: Synergie, Synthese und Strukturaufbau,” Angewandte Chemie 119 (2007): 3876–3899, 10.1002/ange.200604369.

[23] Q. Chen , X. M. Du Jourdin , and P. Knochel , “Transition‐Metal‐Free BF_3_‐Mediated Regioselective Direct Alkylation and Arylation of Functionalized Pyridines Using Grignard or Organozinc Reagents,” Journal of the American Chemical Society 135 (2013): 4958–4961, 10.1021/ja401146v. 23506449

[24] L. C. H. Maddock , M. Mu , A. R. Kennedy , M. García‐Melchor , and E. Hevia , “Facilitating the Ferration of Aromatic Substrates Through Intramolecular Sodium Mediation,” Angewandte Chemie 133 (2021): 15424–15429, 10.1002/ange.202104275. PMC8362017 33950575

[25] A. Logallo , L. C. H. Maddock , M. Mu , et al., “Unlocking the Metalation Applications of TMP‐Powered Fe and Co(II) Bis(amides): Synthesis, Structure and Mechanistic Insights,” Angewandte Chemie 136 (2024): e202402907, 10.1002/anie.202402907. 38563772

[26] K. Shen , Y. Fu , J. N. Li , L. Liu , and Q. X. Guo , “What Are the pKa Values of C─H Bonds in Aromatic Heterocyclic Compounds in DMSO?,” Tetrahedron 63 (2007): 1568–1576, 10.1016/j.tet.2006.12.032.

[27] A. Panda , M. Stender , M. M. Olmstead , P. Klavins , and P. P. Power , “Reactions of M{N(SiMe_3_)_2_}_2_ (M=Mn, Fe or Co) With Pyridine and 4,4′‐Bipyridyl: Structural and Magnetic Studies,” Polyhedron 22 (2003): 67–73, 10.1016/S0277-5387(02)01264-0.

[28] C. Y. Lin , J. C. Fettinger , and P. P. Power , “Reversible Complexation of Lewis Bases to Low‐Coordinate Fe(II), Co(II), and Ni(II) Amides: Influence of the Metal, Donor Ligand, and Amide Substituent on Binding Constants,” Inorganic Chemistry 56 (2017): 9892–9902, 10.1021/acs.inorgchem.7b01387. 28758744

[29] D. F. Evans , “400. The Determination of the Paramagnetic Susceptibility of Substances in Solution by Nuclear Magnetic Resonance,” Journal of the Chemical Society 81 (1959): 2003–2005, 10.1039/JR9590002003.

[30] E. M. Schubert , “Utilizing the Evans Method With a Superconducting NMR Spectrometer in the Undergraduate Laboratory,” Journal of Chemical Education 69 (1992): 62, 10.1021/ed069p62.1.

[31] C. Piguet , “Paramagnetic Susceptibility by NMR: The "Solvent Correction" Removed for Large Paramagnetic Molecules,” Journal of Chemical Education 74 (1997): 815–816, 10.1021/ed074p815.

[32] J. Wen , S. Qin , L. F. Ma , et al., “Iron‐Mediated Direct Suzuki−Miyaura Reaction: A New Method for the Ortho ‐Arylation of Pyrrole and Pyridine,” Organic Letters 12 (2010): 2694–2697, 10.1021/ol100838m. 20481607

[33] A. M. Berman , J. C. Lewis , R. G. Bergman , and J. A. Ellman , “Rh(I)‐Catalyzed Direct Arylation of Pyridines and Quinolines,” Journal of the American Chemical Society 130 (2008): 14926–14927, 10.1021/ja8059396. 18855360 PMC2647742

[34] J. V. Obligacion , S. P. Semproni , and P. J. Chirik , “Cobalt‐Catalyzed C─H Borylation,” Journal of the American Chemical Society 136 (2014): 4133–4136, 10.1021/ja500712z. 24588541

[35] Y. Nakao , K. S. Kanyiva , and T. Hiyama , “A Strategy for C−H Activation of Pyridines: Direct C‐2 Selective Alkenylation of Pyridines by Nickel/Lewis Acid Catalysis,” Journal of the American Chemical Society 130 (2008): 2448–2449, 10.1021/ja710766j. 18247621

[36] K. Godula , B. Sezen , and D. Sames , “Site‐Specific Phenylation of Pyridine Catalyzed by Phosphido‐Bridged Ruthenium Dimer Complexes: a Prototype for C−H Arylation of Electron‐Deficient Heteroarenes,” Journal of the American Chemical Society 127 (2005): 3648–3649, 10.1021/ja042510p. 15771470

[37] M. M. Shoshani , “Cooperative Heterometallic Platforms Enabling Selective C─H Bond Activation and Functionalization of Pyridines,” Cell Reports Physical Science 4 (2023): 101213, 10.1016/j.xcrp.2022.101213.

[38] N. Gorgas , A. J. P. White , and M. R. Crimmin , “Cooperative C─H Bond Activation by a Low‐Spin d^6^ Iron–Aluminum Complex,” Journal of the American Chemical Society 144 (2022): 8770–8777, 10.1021/jacs.2c02662. 35512338 PMC9121387

[39] N. H. Hunter , E. M. Lane , K. M. Gramigna , C. E. Moore , and C. M. Thomas , “C─H Bond Activation Facilitated by Bis(phosphinoamide) Heterobimetallic Zr/Co Complexes,” Organometallics 40 (2021): 3689–3696, 10.1021/acs.organomet.1c00511.

[40] T. Muraoka , Y. Ishii , N. Siti , M. Nasu , N. A. Wahida , and K. Ueno , “Syntheses and Structures of Gallyliron Complexes With Pyridine Ligands and Their Reactions With Methyl Acrylate,” Organometallics 40 (2021): 3066–3075, 10.1021/acs.organomet.1c00419.

[41] P. Gros and Y. Fort , “Aminoalkoxide‐Mediated Formation and Stabilization of Phenylpyridyllithium: Straightforward Access to Phenylpyridine Derivatives,” The Journal of Organic Chemistry 68 (2003): 2028–2029, 10.1021/jo026706o. 12608830

[42] A. Krasovskiy , V. Krasovskaya , and P. Knochel , “Gemischte Mg/Li‐Amide des Typs R_2_NMgCl·LiCl Als Hoch Effiziente Basen Zur Regioselektiven Synthese Funktionalisierter Aryl‐ und Heteroarylmagnesium‐Verbindungen,” Angewandte Chemie 118 (2006): 3024–3027, 10.1002/ange.200504024.

[43] K. Gao , P.‐S. Lee , T. Fujita , and N. Yoshikai , “Cobalt‐Catalyzed Hydroarylation of Alkynes Through Chelation‐Assisted C−H Bond Activation,” Journal of the American Chemical Society 132 (2010): 12249–12251, 10.1021/ja106814p. 20712366

[44] W. G. Whitehurst , J. Kim , S. G. Koenig , and P. J. Chirik , “C─H Activation by Isolable Cationic Bis(phosphine) Cobalt(III) Metallacycles,” Journal of the American Chemical Society 144 (2022): 19186–19195, 10.1021/jacs.2c08865. 36194198 PMC9585578

[45] B. Punji , W. Song , G. A. Shevchenko , and L. Ackermann , “Cobalt‐Catalyzed C─H Bond Functionalizations With Aryl and Alkyl Chlorides,” Chemistry – A European Journal 19 (2013): 10605–10610, 10.1002/chem.201301409. 23821432

[46] P. Gandeepan , T. Müller , D. Zell , G. Cera , S. Warratz , and L. Ackermann , “3d Transition Metals for C─H Activation,” Chemical Reviews 119 (2019): 2192–2452, 10.1021/acs.chemrev.8b00507. 30480438

[47] R. Shang , L. Ilies , and E. Nakamura , “Iron‐Catalyzed C─H Bond Activation,” Chemical Reviews 117 (2017): 9086–9139, 10.1021/acs.chemrev.6b00772. 28378590

[48] J. Norinder , A. Matsumoto , N. Yoshikai , and E. Nakamura , “Iron‐Catalyzed Direct Arylation Through Directed C−H Bond Activation,” Journal of the American Chemical Society 130 (2008): 5858–5859, 10.1021/ja800818b. 18410099

[49] N. Yoshikai , S. Asako , T. Yamakawa , L. Ilies , and E. Nakamura , “Iron‐Catalyzed C─H Bond Activation for the Ortho‐Arylation of Aryl Pyridines and Imines With Grignard Reagents,” Chemistry—An Asian Journal 6 (2011): 3059–3065, 10.1002/asia.201100470. 21898839

[50] L. Ackermann , “Cobalt‐Catalyzed C─H Arylations, Benzylations, and Alkylations With Organic Electrophiles and Beyond,” Journal of Organic Chemistry 79 (2014): 8948–8954, 10.1021/jo501361k. 25102352

[51] Z. Dong , Z. Ren , S. J. Thompson , Y. Xu , and G. Dong , “Transition‐Metal‐Catalyzed C─H Alkylation Using Alkenes,” Chemical Reviews 117 (2017): 9333–9403, 10.1021/acs.chemrev.6b00574. 28125210

[52] R. Azpíroz , A. Di Giuseppe , A. Urriolabeitia , et al., “Hydride‐Rhodium(III)‐N‐Heterocyclic Carbene Catalyst for Tandem Alkylation/Alkenylation via C‐H Activation,” ACS Catalysis 9 (2019): 9372–9386, 10.1021/acscatal.9b01233.

[53] M. S. Eum , S. C. Chong , Y. K. Song , et al., “Ligand Effects on Luminescence of New Type Blue Light‐Emitting Mono(2‐Phenylpyridinato)Iridium(III) Complexes,” Inorganic Chemistry 47 (2008): 6289–6295, 10.1021/ic800376e. 18537242

[54] L. Rubio‐Pérez , M. Iglesias , J. Munárriz , et al., “A Well‐Defined NHC–Ir( iii ) Catalyst for the Silylation of Aromatic C─H Bonds: Substrate Survey and Mechanistic Insights,” Chemical Science 8 (2017): 4811–4822, 10.1039/C6SC04899D. 28959403 PMC5602144

[55] G. Fauvet , M. Massaux , and R. Chevalier , “Etude De la Forme Cristalline Du Benzonitrile à 198 K,” Acta Crystallographica 34 (1978): 1376–1378, 10.1107/S0567740878005658.

[56] R. R. Fraser , M. Bresse , and T. S. Mansour , “pK a Measurements in Tetrahydrofuran,” Journal of the Chemical Society, Chemical Communications 11 (1983): 620–621, 10.1039/c39830000620.

[57] M. Hasyeoui , P. M. Chapple , F. Lassagne , et al., “Lateral Deprotometallation‐Trapping Reactions on Methylated Pyridines, Quinolines and Quinoxalines Using Lithium Diethylamide,” European Journal of Organic Chemistry 26 (2023): e202300555, 10.1002/ejoc.202300555.

[58] A. R. Kennedy , R. E. Mulvey , R. I. Urquhart , and S. D. Robertson , “Lithium, Sodium and Potassium Picolyl Complexes: Syntheses, Structures and Bonding,” Dalton Transactions 43 (2014): 14265–14274, 10.1039/C4DT00808A. 24770550

[59] D. E. Anderson , A. H. N. Truong , and E. Hevia , “Dual Basicity and Nucleophilicity of Organosodium Reagents in Benzylic C H Additions of Toluenes to Diarylethenes and Ketones,” Chemistry – A European Journal 30 (2024): e202400492, 10.1002/chem.202400492. 38651778

[60] D. E. Anderson , A. Tortajada , and E. Hevia , “Highly Reactive Hydrocarbon Soluble Alkylsodium Reagents for Benzylic Aroylation of Toluenes Using Weinreb Amides,” Angewandte Chemie 135 (2023): e202218498, 10.1002/ange.202218498. 36636916

[61] V. L. Blair , D. C. Blakemore , D. Hay , E. Hevia , and D. C. Pryde , “Alkali‐Metal Mediated Zincation of N‐Heterocyclic Substrates Using the Lithium Zincate Complex, (THF)Li(TMP)Zn(*t*Bu)_2_ and Applications in in Situ Cross Coupling Reactions,” Tetrahedron Letters 52 (2011): 4590–4594, 10.1016/j.tetlet.2011.06.090.

[62] B. Conway , D. V. Graham , E. Hevia , A. R. Kennedy , J. Klett , and R. E. Mulvey , “Structurally‐Defined Potassium‐Mediated Regioselective Zincation of Amino‐ and Alkoxy‐Substituted Pyridines,” Chemical Communications 23 (2008): 2638–2640, 10.1039/b805606d. 18535692

[63] E. Nagaradja , F. Chevallier , T. Roisnel , V. Jouikov , and F. Mongin , “Deprotonative Metalation of Aromatic Compounds Using Mixed Lithium–iron Combinations,” Tetrahedron 68 (2012): 3063–3073, 10.1016/j.tet.2012.02.019.

[64] M. Hedidi , G. Bentabed‐Ababsa , A. Derdour , et al., “Deprotometalation of Substituted Pyridines and Regioselectivity‐Computed CH Acidity Relationships,” Tetrahedron 72 (2016): 2196–2205, 10.1016/j.tet.2016.03.022.

[65] G. Shi , S. Takagishi , and M. Schlosser , “Metalated Fluoropyridines and Fluoroquinolines as Reactive Intermediates: New Ways for Their Regioselective Generation,” Tetrahedron 50 (1994): 1129–1134, 10.1016/S0040-4020(01)80824-3.

[66] S. A. Orr , P. C. Andrews , and V. L. Blair , “Main Group Metal‐Mediated Transformations of Imines,” Chemistry – A European Journal 27 (2021): 2569–2588, 10.1002/chem.202003108. 32761667

[67] S. Camadanli , R. Beck , U. Flörke , and H. Klein , “C−H Activation of Imines by Trimethylphosphine‐Supported Iron Complexes and Their Reactivities,” Organometallics 28 (2008): 2300–2310, 10.1021/om800828j.

[68] R. G. Little and R. J. Doedens , “Crystal and Molecular Structure of an o‐Metalated Benzylidineaniline‐Manganese Carbonyl Complex, Tetracarbonyl‐2‐(N‐Phenylformimidoyl)phenylmanganese, C_6_H_5_NCHC_6_H_4_Mn(CO)_4_ ,” Inorganic Chemistry 12 (1973): 840–844, 10.1021/ic50122a027.

[69] Z. S. Lincoln , M. R. Hoffbauer , and V. M. Iluc , “Directed C─H Activation With Iron Carbene Complexes,” Inorganic Chemistry Frontiers 11 (2024): 5579–5586, 10.1039/D4QI01088D.

[70] T. D. Krizan and J. C. Martin , “In Situ Trapping of Ortho Lithiated Benzenes Containing Electrophilic Directing Groups,” Journal of the American Chemical Society 105 (1983): 6155–6157, 10.1021/ja00357a034.

[71] K. Groll , S. M. Manolikakes , X. Mollat du Jourdin , et al., “Regioselektive Metallierungen von Pyrimidinen und Pyrazinen mit Frustrierten Lewis‐Paaren aus BF_3_·OEt_2_ und Sterisch Gehinderten Mg‐ und Zn‐Amidbasen,” Angewandte Chemie 125 (2013): 6909–6913, 10.1002/ange.201301694.

[72] Z. Dong , G. C. Clososki , S. H. Wunderlich , A. Unsinn , J. Li , and P. Knochel , “Direct Zincation of Functionalized Aromatics and Heterocycles by Using a Magnesium Base in the Presence of ZnCl_2_ ,” Chemistry – A European Journal 15 (2009): 457–468, 10.1002/chem.200801558. 19035380

[73] S. E. Baillie , V. L. Blair , D. C. Blakemore , et al., “New Lithium‐Zincate Approaches for the Selective Functionalisation of Pyrazine: Direct Dideprotozincation vs. Nucleophilic Alkylation,” Chemical Communications 48 (2012): 1985–1987, 10.1039/c2cc16959b. 22237409

[74] Deposition numbers 2517148–2517171 (for **2^Fe^ **, **2a**, **2f**, **3a‐d**, **3f‐m**, **4**, **5**, **6a‐b**, **7b‐e**, **8c** respectively), contain the supplementary crystallographic data for this paper These data are provided free of charge by the joint Cambridge Crystallographic Data Centre and Fachinformationszentrum Karlsruhe Access Structures service.

