# Supplementary material for: Coordination‐Driven Direct C─H Metalation of N‐Heterocycles With a Superbasic Co(II) Amide Co(TMP)2
Source: Angew Chem Int Ed Engl. 2026 Jun 15;65(34):e7728005. doi: 10.1002/anie.7728005 (PMC13480490; doi:10.1002/anie.7728005)
Supplement: Supplementary file 1 [file ANIE-65-e7728005-s001.pdf]

Supplementary Information for:

Coordination-Driven Direct C–H Metalation of *N*-heterocycles with a Super Basic Co(II) Amide $Co(TMP)_2$

Na Jin,[a] Manting Mu,[b] Max García-Melchor[b],[c],[d]* and Eva Hevia[a]*

[a] Departement für Chemie, Biochemie und Pharmazie, Universität Bern, 3012 Bern, Switzerland

[b] School of Chemistry, Trinity College Dublin, College Green, Dublin 2, Ireland

[c] CIC energiGUNE, Parque Tecnológico de Álava, Albert Einstein 48, 01510 Vitoria-Gasteiz, Spain

[d] IKERBASQUE, Basque Foundation for Science, Plaza de Euskadi 5, 48009 Bilbao, Spain

Table of Contents

***Experimental Procedures* 4**

General Considerations 4

Syntheses of Numbered Compounds 5

[(py)Fe(TMP)$_2$] (2^{Fe}) 5
[(py)Co(TMP)$_2$] (2a)....5
[(2-Methoxy-py)Co(TMP)$_2$] (2f)....5
[(2-Pyridinyl)Co$_2$(TMP)$_3$] (3a) 5
[(6-Phenyl-2-pyridinyl)Co$_2$(TMP)$_3$] (3b)....6
[(2-CH$_2$C$_5$H$_4$N)Co$_2$(TMP)$_3$] (3c)....6
[(4-Methyl-2-pyridinyl)Co$_2$(TMP)$_3$] (3d) 6
[(3-Methyl-2-pyridinyl)Co$_2$(TMP)$_3$] + [(3-Methyl-6-pyridinyl)Co$_2$(TMP)$_3$] (3e) 6
[(6-Methoxy-2-pyridinyl)Co$_2$(TMP)$_3$] (3f) 7
[(4-Methoxy-2-pyridinyl)Co$_2$(TMP)$_3$] (3g) 7
[(3-Methoxy-2-pyridinyl)Co$_2$(TMP)$_3$] (3h) 7
[(6-Fluoro-2-pyridinyl)Co$_2$(TMP)$_3$] (3i) 8
[(4,6-Difluoro-2-pyridinyl)Co$_2$(TMP)$_3$] (3j) 8
[(3-Fluoro-2-pyridinyl)Co$_2$(TMP)$_3$] (3k) 8
[(8-Caffeinyl)Co$_2$(TMP)$_3$] (3l) 9
[{PhN=(Ph)C}Co$_2$(TMP)$_3$] (3m)....9
[(MeO)Co$_2$(TMP)$_3$] (4) 9
[{(Ph)(TMP)C=N}Co$_2$(TMP)$_3$] (5) 10
[(2,5-C$_4$H$_2$N$_2$-pyz)Co$_4$(TMP)$_6$] (6a) 10
[(4,6-C$_4$H$_2$N$_2$-pym)Co$_4$(TMP)$_6$] (6b)....10
[(4-C$_4$H$_3$N$_2$-pym)Co$_2$(TMP)$_3$] (7b)....10
[(3-C$_4$H$_3$N$_2$-pdz)Co$_2$(TMP)$_3$] (7c) 11
[(2-Quinoxalinyl)Co$_2$(TMP)$_3$] (7d)....11
[(4-Quinazolinyl)Co$_2$(TMP)$_3$] (7e) 11
2-(2,2,6,6-Tetramethylpiperidin-1-yl)pyridine (8a) 12
2-Phenyl-6-(2,2,6,6-tetramethylpiperidin-1-yl)pyridine (8b) 12
2,5-bis(2,2,6,6-tetramethylpiperidin-1-yl)pyrazine (8c) 12
4-(2,2,6,6-Tetramethylpiperidin-1-yl)pyrimidine (8d) 13

***NMR Spectra of Numbered Compounds* 14**

[(py)Fe(TMP)$_2$] (2^{Fe}) 14
[(py)Co(TMP)$_2$] (2a)....15
[(2-Methoxy-py)Co(TMP)$_2$] (2f)....16
[(2-Pyridinyl)Co$_2$(TMP)$_3$] (3a) 17
[(6-Phenyl-2-pyridinyl)Co$_2$(TMP)$_3$] (3b)....18
[(2-CH$_2$C$_5$H$_4$N)Co$_2$(TMP)$_3$] (3c)....19
[(4-Methyl-2-pyridinyl)Co$_2$(TMP)$_3$] (3d) 20
[(6-Methoxy-2-pyridinyl)Co$_2$(TMP)$_3$] (3f) 21
[(4-Methoxy-2-pyridinyl)Co$_2$(TMP)$_3$] (3g) 22
[(3-Methoxy-2-pyridinyl)Co$_2$(TMP)$_3$] (3h) 23
[(6-Fluoro-2-pyridinyl)Co$_2$(TMP)$_3$] (3i) 24
[(4,6-Difluoro-2-pyridinyl)Co$_2$(TMP)$_3$] (3j) 26
[(3-Fluoro-2-pyridinyl)Co$_2$(TMP)$_3$] (3k) 28
[(8-Caffeinyl)Co$_2$(TMP)$_3$] (3l) 30
[{PhN=(Ph)C}Co$_2$(TMP)$_3$] (3m)....31
[(MeO)Co$_2$(TMP)$_3$] (4) 32
[{(Ph)(TMP)C=N}Co$_2$(TMP)$_3$] (5) 33
[(2,5-C$_4$H$_2$N$_2$-pyz)Co$_4$(TMP)$_6$] (6a) 34
[(4,6-C$_4$H$_2$N$_2$-pym)Co$_4$(TMP)$_6$] (6b)....36
[(4-C$_4$H$_3$N$_2$-pym)Co$_2$(TMP)$_3$] (7b)....37
[(3-C$_4$H$_3$N$_2$-pdz)Co$_2$(TMP)$_3$] (7c) 38
[(2-Quinoxalinyl)Co$_2$(TMP)$_3$] (7d)....39

[(4-Quinazolinyl)$Co_2(TMP)_3$] (7e) 40
2-(2,2,6,6-Tetramethylpiperidin-1-yl)pyridine (8a) 41
2-Phenyl-6-(2,2,6,6-tetramethylpiperidin-1-yl)pyridine (8b) 43
2,5-Bis(2,2,6,6-tetramethylpiperidin-1-yl)pyrazine (8c) 45
4-(2,2,6,6-Tetramethylpiperidin-1-yl)pyrimidine (8d) 47

NMR Studies and Supplementary Experiments 49
$Co(TMP)_2$ (1^{Co}) + pyridine (1.0 eq) in C_6D_6 49
$Co(TMP)_2$ (1^{Co}) + pyridine (0.5 eq) in C_6D_6 50
$Co(TMP)_2$ (1^{Co}) + IMes (1.0 eq) + pyridine (1.0 eq) in C_6D_6 51
$Fe(TMP)_2$ (1^{Fe}) + pyridine (1.0 eq) in C_6D_6 52
$Fe(TMP)_2$ (1^{Fe}) + pyridine (1.0 eq) in d_8-THF 53
$Co(TMP)_2$ (1^{Co}) + 3-picoline (1.0 eq) in C_6D_6 54
$Co(TMP)_2$ (1^{Co}) + 2-methoxypyridine (1.0 eq) in C_6D_6 55
$Co(TMP)_2$ (1^{Co}) + methyl picolinate (0.5eq) in C_6D_6 56
$Co(TMP)_2$ (1^{Co}) + pyrazine (0.25 eq) in C_6D_6 57
$Co(TMP)_2$ (1^{Co}) + pyrazine (0.5 eq) in C_6D_6 58
[(py)$Co(TMP)_2$] (2a) + $Fe(TMP)_2$ (1^{Fe}) (1.0 eq) in C_6D_6 59
[(py)$Fe(TMP)_2$] (2^{Fe}) + $Co(TMP)_2$ (1^{Co}) (1.0 eq) in C_6D_6 60
[(2-Pyridinyl)$Co_2(TMP)_3$] (3a) + CyBr (5.0 eq) in benzene 61
Pyridine + CyBr (3.0 eq) + $Co(TMP)_2$ (1^{Co}) (20 mol%) in benzene 62
[(2-Pyridinyl)$Co_2(TMP)_3$] (3a) + styrene (5.0 eq) in C_6D_6 63

Solution Effective Magnetic Moment Calculations 65

X-Ray Crystallographic Figures and Data 67

X-Ray Figures 67

Table of Bond Parameters in 3a-d, 3f-m, 6a-b, 7b-e 74

X-Ray Tables 75

Computational Methods 84
Transition-state prediction of experimentally observed selectivity 85
Speciation study of di-nuclear Co and Fe complexes 86
C–H metalation of pyridine with $Fe(TMP)_2$ 87
Calculated C–H acidity of free and $Co(TMP)_2$-coordinated pyridine 87

References 88

Experimental Procedures

General Considerations

All reactions were carried out under an inert dry argon atmosphere utilising standard Schlenk line and glove-box techniques (MBraun, MB10 Compact or UniLab Pro, <0.5 ppm H_2O, O_2). Co(TMP)$_2$ (**1^{Co}**) and Fe(TMP)$_2$ (**1^{Fe}**) were prepared according to literature procedures from Hevia *et al*.[1]

Pentane and benzene were dried prior to synthesis by Grubbs column[2] (MBraun SPS 5) to remove any traces of moisture and dissolved oxygen and then further dried by stirring over $NaK_{2.8}$ overnight before vacuum transferring and subsequently storing over 4 Å molecular sieves under argon atmosphere. THF was dried by heating to reflux over Na wire/benzophenone ketyl radical and stored over 4 Å molecular sieves under argon atmosphere. Pyridine was distilled from CaH_2 and stored over 4 Å molecular sieves under argon atmosphere. All other liquid reagents were commercially available and cycled through three rounds of degassing by employing a freeze-pump-thaw method before subsequently storing over 4 Å molecular sieves under argon atmosphere. All solid reagents are commercially available and were used as received.

Deuterated solvents C_6D_6 and d_8-THF for NMR spectroscopy were dried over $NaK_{2.8}$, vacuum transferred and stored over 4 Å molecular sieves in the glove-box prior to use. All NMR spectroscopy samples were prepared inside the inert argon atmosphere of the glove-box. ^{1}H (300.1 MHz), ^{13}C (75.5 MHz) and ^{19}F{^{1}H} (282.4 MHz) NMR spectra were recorded on a Bruker Avance III HD 300 spectrometer at 300 K and analysed using MestReNova (v16.0.0-39276). NMR spectra were referenced internally to the corresponding residual protio solvent peaks. Solution magnetic susceptibilities were determined by the Evans method at 300 K.[3–5] The presented values are the average of determinations in triplicate to ensure consistency.

CHN elemental microanalyses were performed by the Analytical Research and Services (Schürch Group) of the University of Bern on a Flash 2000 Organic Elemental Analyser (Thermo Scientific). The presented values are the average of determinations in triplicate to ensure consistency. HRMS measurements were performed by the Analytical Research and Services (Schürch Group) of the University of Bern using a ThermoScientific LTQ Orbitrap XL, equipped with a Nanoelectrospray Ion Source (NSI) or a ThermoScientific Q Exactive GC-MS.

Syntheses of Numbered Compounds

[(py)Fe(TMP)$_2$] (2^{Fe})

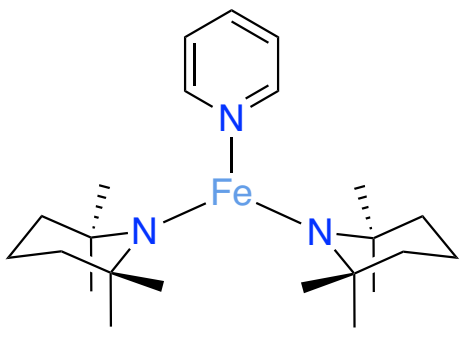

$Fe(TMP)_2$ (67.3 mg, 0.2 mmol) was dissolved in pentane to give a bright orange solution. Upon addition of pyridine (16.1 µL, 0.2 mmol) the solution turned blood orange. The solution was left to stir at room temperature for 10 min and then left at −30 °C overnight. Bright orange crystals were isolated, washed with cold pentane and dried *in vacuo* (77.5 mg, 93%).

^{1}H NMR (C_6D_6, 300 K) δ (ppm) = 154.57, 108.90, 31.54, 12.12, –16.74
Anal. Calcd for $C_{23}H_{41}FeN_3$: C 66.50, H 9.95, N 10.11 Found: C 66.34, H 9.92, N 10.18
Solution Magnetic Moment (C_6D_6, 300 K) = 5.18 μ_B

[(py)Co(TMP)$_2$] (2a)

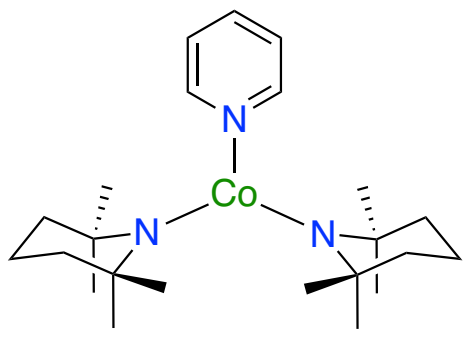

$Co(TMP)_2$ (33.9 mg, 0.1 mmol) was dissolved in pentane to give a dark red solution. Upon addition of pyridine (8.1 µL, 0.1 mmol) the solution turned yellow green. The solution was left to stir at room temperature for 10 min and then left at −30 °C overnight. Green crystals were isolated, washed with cold pentane and dried *in vacuo* (30.7 mg, 73%).

^{1}H NMR (C_6D_6, 300 K) δ (ppm) = 230.25, 86.94, 24.18, 1.53, –10.07, –59.63
Anal. Calcd for $C_{23}H_{41}CoN_3$: C 66.00, H 9.87, N 10.04 Found: C 65.96, H 9.93, N 10.12
Solution Magnetic Moment (C_6D_6, 300 K) = 4.06 μ_B

[(2-Methoxy-py)Co(TMP)$_2$] (2f)

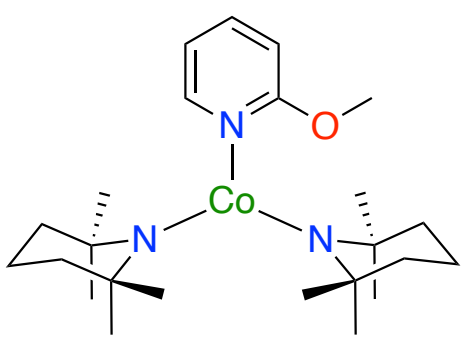

$Co(TMP)_2$ (67.9 mg, 0.2 mmol) was dissolved in pentane to give a dark red solution. Upon addition of 2-methoxypyridine (21.0 µL, 0.2 mmol) the solution turned yellow green. The solution was left to stir at room temperature for 10 min and then left at −30 °C overnight. Green crystals were isolated, washed with cold pentane and dried *in vacuo* (59.2 mg, 66%).

^{1}H NMR (C_6D_6, 300 K) δ (ppm) = 125.05 [TMP C*H*$_2$, 8H], 116.58 [TMP C*H*$_2$, 4H], 71.19 [Pyridyl C*H*, 1H], 41.60 [Pyridyl C*H*, 1H], 35.20 [Pyridyl C*H*, 1H], 6.59 [Pyridyl C*H*, 1H], 5.51 [Picolyl *Me*, 3H], –6.12 [TMP *Me*, 24H].
Anal. Calcd for $C_{24}H_{43}CoN_3O$: C 64.26, H 9.66, N 9.37 Found: C 63.95, H 9.80, N 9.68
Solution Magnetic Moment (C_6D_6, 300 K) = 4.28 μ_B

[(2-Pyridinyl)Co$_2$(TMP)$_3$] (3a)

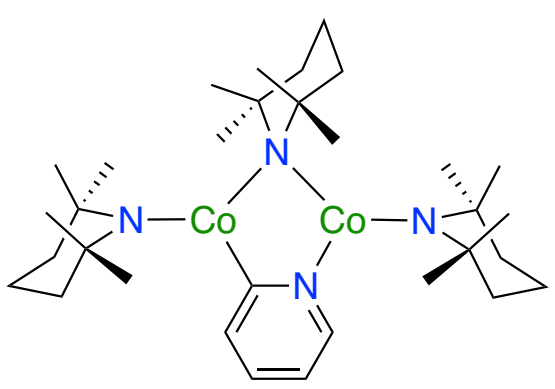

$Co(TMP)_2$ (67.9 mg, 0.2 mmol) was dissolved in benzene to give a dark red solution. Upon addition of pyridine (8.1 µL, 0.1 mmol) the solution turned brownish red. The solution was left to stir at 70 °C for 4 hours to give a dark orange solution. The solvent was removed under vacuum to afford a dark-coloured microcrystalline solid which was then dissolved in a mixture of THF and pentane. The solution was left to slowly evaporate at −30 °C over a few days. Dark orange crystals were isolated, washed with cold pentane and dried *in vacuo* (48.8 mg, 79%).

^{1}H NMR (C_6D_6, 300 K) δ (ppm) = 86.97 [Pyridyl C*H*, 1H], 66.56 [Pyridyl C*H*, 1H], 54.81 [Pyridyl C*H*, 1H], 54.39 [Pyridyl C*H*, 1H], 31.28 [TMP C*H*$_2$, 4H], 30.31 [TMP C*H*$_2$, 2H], 22.42 [TMP C*H*$_2$, 2H], 21.63 [TMP C*H*$_2$, 2H], 15.95 [TMP *Me*, 12H], 15.95 [TMP C*H*$_2$, 4H], 10.17 [TMP C*H*$_2$, 4H], –26.60 [TMP *Me*, 12H]
Anal. Calcd for $C_{32}H_{58}Co_2N_4$: C 62.32, H 9.48, N 9.08 Found: C 62.22, H 9.40, N 9.12

Solution Magnetic Moment (C_6D_6, 300 K) = 3.75 μ_B

[(6-Phenyl-2-pyridinyl)Co2(TMP)3] (3b)

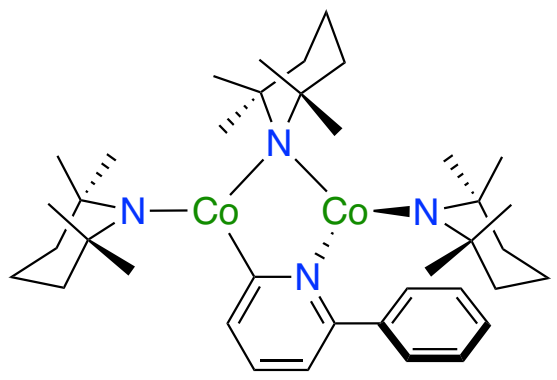

Co(TMP)2 (271.5 mg, 0.8 mmol) was dissolved in benzene to give a dark red solution. Upon addition of 2-phenylpyridine (57.2 µL, 0.4 mmol) the solution turned dark orange. The solution was left to stir at 70 °C for 3 hours. The solvent was removed under vacuum to afford a dark-coloured microcrystalline solid which was isolated, washed with cold pentane and dried *in vacuo* (203.8 mg, 74%).

^{1}H NMR (C_6D_6, 300 K) δ (ppm) = 49.46 [Pyridyl C*H*, 1H], 41.01 [Pyridyl C*H*, 1H], 39.93 [Pyridyl C*H*, 1H], 36.50 [TMP CH_2, 4H], 35.14 [TMP CH_2, 2H], 24.71 [TMP CH_2, 2H], 23.41 [TMP CH_2, 2H], 21.71 [TMP CH_2, 2H], 21.14 [TMP CH_2, 2H], 14.16 [TMP CH_2, 4H], 10.11 [TMP *Me*, 12H], 8.60 [Phenyl C*H*, 2H], 8.09 [Phenyl C*H*, 1H], –1.48 [TMP *Me*, 12H], –38.75 [TMP *Me*, 12H]
Anal. Calcd for $C_{38}H_{62}Co_2N_4$: C 65.88, H 9.02, N 8.09 Found: C 65.64, H 9.03, N 8.35
Solution Magnetic Moment (C_6D_6, 300 K) = 3.56 μ_B

[(2-CH2C5H4N)Co2(TMP)3] (3c)

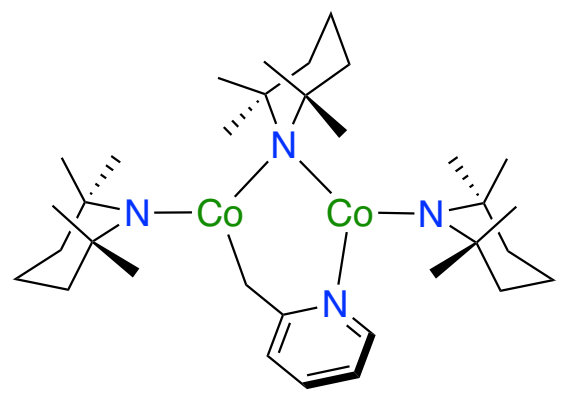

Co(TMP)2 (67.9 mg, 0.2 mmol) was dissolved in pentane to give a dark red solution. 2-Picoline (9.9 µL, 0.1 mmol) was added to form a dark orange solution which was left to stir at room temperature for 1 hour. The solution was left to slowly evaporate at −30 °C over a few days. Dark orange crystals were isolated, washed with cold pentane and dried *in vacuo* (42.8 mg, 68%).

^{1}H NMR (C_6D_6, 300 K) δ (ppm) = 115.56, 60.79, 28.15, 22.54, 8.64, –1.63, –5.92, –22.01, –38.02, –58.10
Anal. Calcd for $C_{33}H_{60}Co_2N_4$: C 62.84, H 9.59, N 8.88 Found: C 62.89, H 9.59, N 9.00
Solution Magnetic Moment (C_6D_6, 300 K) = 4.68 μ_B

[(4-Methyl-2-pyridinyl)Co2(TMP)3] (3d)

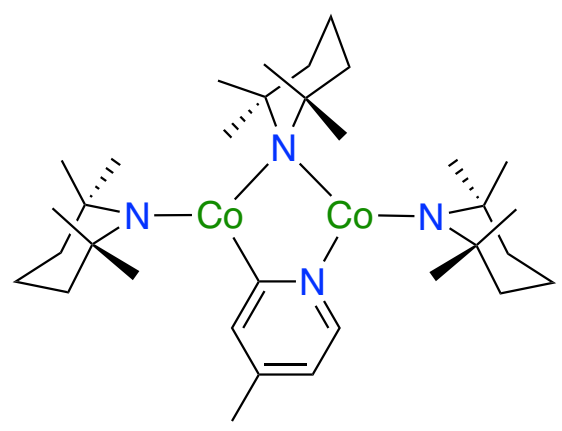

Co(TMP)2 (67.9 mg, 0.2 mmol) was dissolved in benzene to give a dark red solution. Upon addition of 4-picoline (9.7 µL, 0.1 mmol) the solution turned brownish red. The solution was left to stir at 70 °C for 5 hours to give a dark orange solution. The solvent was removed under vacuum to afford a dark-coloured microcrystalline solid which was then dissolved in a mixture of THF and pentane. The solution was left to slowly evaporate at −30 °C over a few days. Dark orange crystals were isolated, washed with cold pentane and dried *in vacuo* (35.4 mg, 56%).

^{1}H NMR (C_6D_6, 300 K) δ (ppm) = 88.31 [Pyridyl C*H*, 1H], 69.60 [Pyridyl C*H*, 1H], 53.81 [Pyridyl C*H*, 1H], 32.27 [TMP CH_2, 4H], 31.16 [TMP CH_2, 2H], 22.97 [TMP CH_2, 2H], 22.49 [TMP CH_2, 2H], 16.44 [TMP *Me*, 12H], 16.44 [TMP CH_2, 4H], 11.00 [TMP CH_2, 4H], 4.09 [Picolyl *Me*, 3H], –27.44 [TMP *Me*, 12H]
Anal. Calcd for $C_{33}H_{60}Co_2N_4$: C 62.84, H 9.59, N 8.88 Found: C 63.03, H 9.83, N 8.49
Solution Magnetic Moment (C_6D_6, 300 K) = 3.43 μ_B

[(3-Methyl-2-pyridinyl)Co2(TMP)3] + [(3-Methyl-6-pyridinyl)Co2(TMP)3] (3e)

Co(TMP)2 (67.9 mg, 0.2 mmol) was dissolved in benzene to give a dark red solution. Upon addition of 3-picoline (9.7 µL, 0.1 mmol) the solution turned brownish red. The solution was left to stir at 70 °C for 3 hours to give a dark orange solution. The solvent was removed under vacuum to afford a dark-coloured microcrystalline solid which was then dissolved in a mixture of

THF and pentane. The solution was left to slowly evaporate at −30 °C over a few days. Dark orange crystals were isolated, washed with cold pentane and dried *in vacuo* (48.6 mg, 77%).

N.B. The isolated product was determined by ^{1}H NMR to be a 1:1 statistical mixture of the C2- and C6- cobaltated 3-picoline. Therefore, no further characterisation was carried out for **3e**.

[(6-Methoxy-2-pyridinyl)Co_2(TMP)$_3$] (3f)

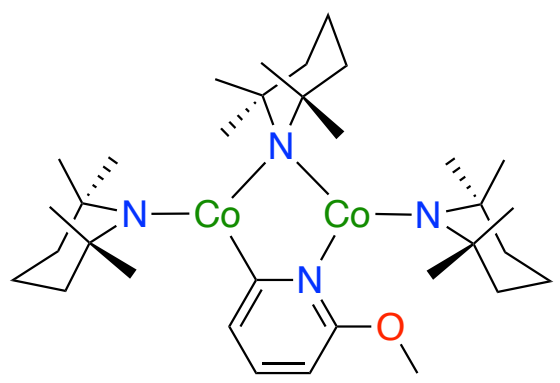

$Co(TMP)_2$ (67.9 mg, 0.2 mmol) was dissolved in benzene to give a dark red solution. Upon addition of 2-methoxypyridine (10.5 µL, 0.1 mmol) the solution turned brownish red. The solution was left to stir at 70 °C for 4 hours to give a dark orange solution. The solvent was removed under vacuum to afford a dark-coloured microcrystalline solid which was then dissolved in a mixture of THF and pentane. The solution was left to slowly evaporate at −30 °C over a few days. Dark orange crystals were isolated, washed with cold pentane and dried *in vacuo* (31.0 mg, 48%).

^{1}H NMR (C_6D_6, 300 K) δ (ppm) = 56.59 [Pyridyl C*H*, 1H], 52.97 [Pyridyl C*H*, 1H], 51.56 [Pyridyl C*H*, 1H], 35.23 [TMP C*H*$_2$, 4H], 34.97 [TMP C*H*$_2$, 2H], 25.02 [TMP C*H*$_2$, 2H], 21.55 [TMP C*H*$_2$, 2H], 16.14 [TMP *Me*, 12H], 15.28 [TMP C*H*$_2$, 4H], 13.12 [Methoxy O*Me*, 3H], 12.25 [TMP C*H*$_2$, 4H], –35.95 [TMP *Me*, 12H]
Anal. Calcd for $C_{33}H_{60}Co_2N_4O$: C 61.29, H 9.35, N 8.66 Found: C 61.15, H 9.52, N 8.35
Solution Magnetic Moment (C_6D_6, 300 K) = 3.73 μ_B

[(4-Methoxy-2-pyridinyl)Co_2(TMP)$_3$] (3g)

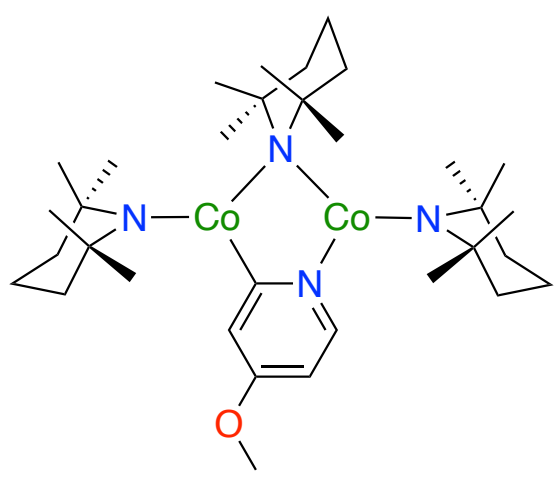

$Co(TMP)_2$ (67.9 mg, 0.2 mmol) was dissolved in benzene to give a dark red solution. Upon addition of 4-methoxypyridine (10.2 µL, 0.1 mmol) the solution turned brownish red. The solution was left to stir at 70 °C for 3 hours to give a dark orange solution. The solvent was removed under vacuum to afford a dark-coloured microcrystalline solid which was then dissolved in a mixture of THF and pentane. The solution was left to slowly evaporate at −30 °C over a few days. Dark orange crystals were isolated, washed with cold pentane and dried *in vacuo* (43.5 mg, 67%).

^{1}H NMR (C_6D_6, 300 K) δ (ppm) = 90.05 [Pyridyl C*H*, 1H], 84.02 [Pyridyl C*H*, 1H], 50.34 [Pyridyl C*H*, 1H], 33.14 [TMP C*H*$_2$, 6H], 23.30 [TMP C*H*$_2$, 4H], 17.95 [TMP C*H*$_2$, 8H], 15.32 [TMP *Me*, 12H], 7.53 [Methoxy O*Me*, 3H], –27.91 [TMP *Me*, 12H]
Anal. Calcd for $C_{33}H_{60}Co_2N_4O$: C 61.29, H 9.35, N 8.66 Found: C 61.09, H 9.40, N 8.83
Solution Magnetic Moment (C_6D_6, 300 K) = 3.89 μ_B

[(3-Methoxy-2-pyridinyl)Co_2(TMP)$_3$] (3h)

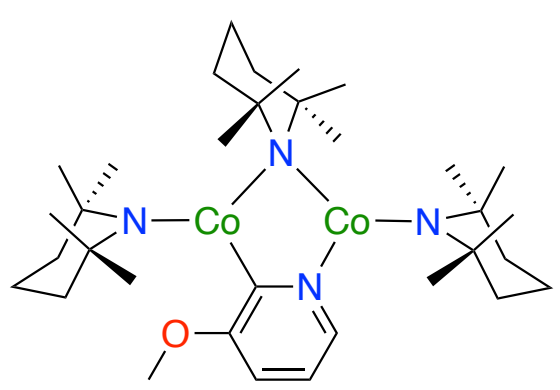

$Co(TMP)_2$ (67.9 mg, 0.2 mmol) was dissolved in pentane to give a dark red solution. Upon addition of 3-methoxypyridine (10.1 µL, 0.1 mmol) the solution turned brownish red. The solution was left to stir at room temperature for 1 hour to give a dark orange solution. The solvent was removed under vacuum to afford a dark-coloured microcrystalline solid which was then dissolved in a mixture of THF and pentane. The solution was left to slowly evaporate at −30 °C over a few days. Dark orange crystals were isolated, washed with cold pentane and dried *in vacuo* (51.1 mg, 79%).

^{1}H NMR (C_6D_6, 300 K) δ (ppm) = 95.41 [Pyridyl C*H*, 1H], 63.94 [Pyridyl C*H*, 1H], 57.66 [Pyridyl C*H*, 1H], 37.81 [TMP C*H*$_2$, 6H], 27.95 [TMP C*H*$_2$, 2H], 24.10 [TMP C*H*$_2$, 2H], 20.04 [TMP *Me*, 12H], 16.22 [TMP C*H*$_2$, 4H], 12.86 [TMP C*H*$_2$, 4H], 8.74 [Methoxy O*Me*, 3H], –32.78 [TMP *Me*, 12H]

Anal. Calcd for $C_{33}H_{60}Co_2N_4O$: C 61.29, H 9.35, N 8.66 Found: C 62.06, H 9.66, N 9.17
Solution Magnetic Moment (C_6D_6, 300 K) = 3.82 μ_B

[(6-Fluoro-2-pyridinyl)Co2(TMP)3] (3i)

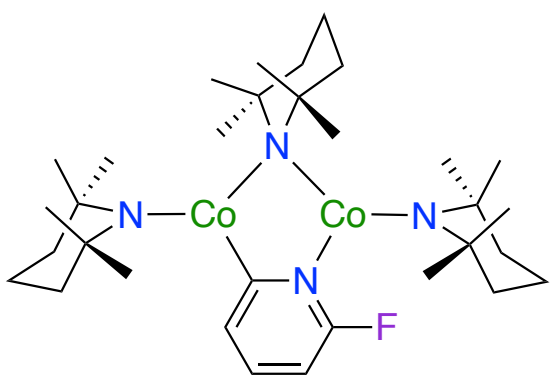

Co(TMP)$_2$ (67.9 mg, 0.2 mmol) was dissolved in pentane to give a dark red solution. Upon addition of 2-fluoropyridine (8.6 µL, 0.1 mmol) the solution turned brownish red. The solution was left to stir at room temperature for 5 hours to give a dark orange solution. The solvent was removed under vacuum to afford a dark-coloured microcrystalline solid which was then dissolved in a mixture of THF and pentane. The solution was left to slowly evaporate at −30 °C over a few days. Dark orange crystals were isolated, washed with cold pentane and dried *in vacuo* (42.2 mg, 66%).

^{1}H NMR (C_6D_6, 300 K) δ (ppm) = 61.50 [Pyridyl C*H*, 1H], 53.25 [Pyridyl C*H*, 1H], 49.91 [Pyridyl C*H*, 1H], 35.00 [TMP C*H*$_2$, 6H], 24.46 [TMP C*H*$_2$, 2H], 21.69 [TMP C*H*$_2$, 2H], 16.33 [TMP *Me*, 12H], 14.17 [TMP C*H*$_2$, 4H], 11.40 [TMP C*H*$_2$, 4H], –32.17 [TMP *Me*, 12H]
^{19}F{^{1}H} NMR (C_6D_6, 300 K) δ (ppm) = –58.72 [*ortho F*]
Anal. Calcd for $C_{32}H_{57}Co_2FN_4$: C 60.56, H 9.05, N 8.83 Found: C 60.88, H 9.27, N 8.95
Solution Magnetic Moment (C_6D_6, 300 K) = 3.69 μ_B

[(4,6-Difluoro-2-pyridinyl)Co2(TMP)3] (3j)

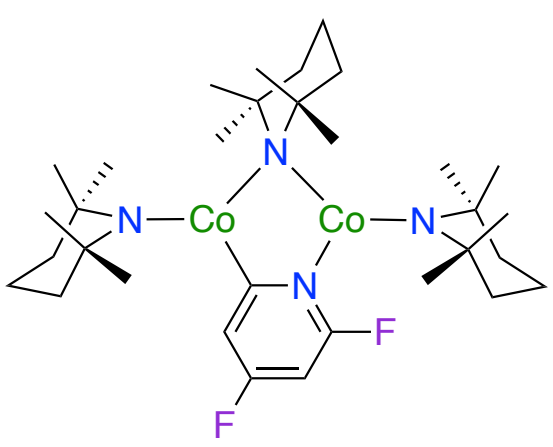

Co(TMP)$_2$ (67.9 mg, 0.2 mmol) was dissolved in pentane to give a dark red solution. Upon addition of 2,4-difluoropyridine (9.1 µL, 0.1 mmol) the solution turned brownish red. The solution was left to stir at room temperature for 1 hour to give a dark orange solution. The solvent was removed under vacuum to afford a dark-coloured microcrystalline solid which was then dissolved in a mixture of THF and pentane. The solution was left to slowly evaporate at −30 °C over a few days. Dark orange crystals were isolated, washed with cold pentane and dried *in vacuo* (49.2 mg, 75%).

^{1}H NMR (C_6D_6, 300 K) δ (ppm) = 79.01 [Pyridyl C*H*, 1H], 45.53 [Pyridyl C*H*, 1H], 37.11 [TMP C*H*$_2$, 4H], 36.64 [TMP C*H*$_2$, 2H], 25.63 [TMP C*H*$_2$, 2H], 22.28 [TMP C*H*$_2$, 2H], 17.24 [TMP *Me*, 12H], 13.39 [TMP C*H*$_2$, 4H], 11.54 [TMP C*H*$_2$, 4H], –32.72 [TMP *Me*, 12H]
^{19}F{^{1}H} NMR (C_6D_6, 300 K) δ (ppm) = 99.31 [*para F*], –51.17 [*ortho F*]
Anal. Calcd for $C_{32}H_{56}Co_2F_2N_4$: C 58.89, H 8.65, N 8.58 Found: C 58.42, H 8.73, N 8.68
Solution Magnetic Moment (C_6D_6, 300 K) = 3.72 μ_B

[(3-Fluoro-2-pyridinyl)Co2(TMP)3] (3k)

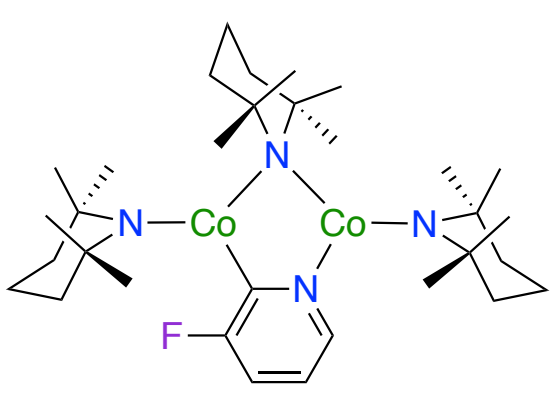

Co(TMP)$_2$ (67.9 mg, 0.2 mmol) was dissolved in pentane to give a dark red solution. Upon addition of 3-fluoropyridine (8.6 µL, 0.1 mmol) the solution turned brownish red. The solution was left to stir at room temperature for 1 hour to give a dark orange solution. The solvent was removed under vacuum to afford a dark-coloured microcrystalline solid which was then dissolved in a mixture of THF and pentane. The solution was left to slowly evaporate at −30 °C over a few days. Dark orange crystals were isolated, washed with cold pentane and dried *in vacuo* (45.0 mg, 71%).

^{1}H NMR (C_6D_6, 300 K) δ (ppm) = 96.98 [Pyridyl C*H*, 1H], 64.92 [Pyridyl C*H*, 1H], 56.82 [Pyridyl C*H*, 1H], 38.57 [TMP C*H*$_2$, 6H], 27.82 [TMP C*H*$_2$, 2H], 25.03 [TMP C*H*$_2$, 2H], 20.04 [TMP *Me*, 12H], 16.67 [TMP C*H*$_2$, 4H], 11.94 [TMP C*H*$_2$, 4H], –31.13 [TMP *Me*, 12H]
^{19}F{^{1}H} NMR (C_6D_6, 300 K) δ (ppm) = –107.80 [*meta F*]

Anal. Calcd for $C_{32}H_{57}Co_2FN_4$: C 60.56, H 9.05, N 8.83 Found: C 60.22, H 8.71, N 9.63
Solution Magnetic Moment (C_6D_6, 300 K) = 3.98 μ_B

[(8-Caffeinyl)Co$_2$(TMP)$_3$] (3l)

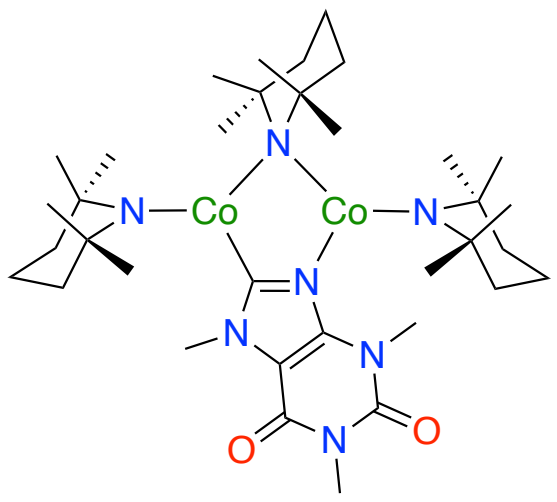

$Co(TMP)_2$ (135.8 mg, 0.4 mmol) was dissolved in pentane to give a dark red solution. Caffeine (38.8 mg, 0.2 mmol) was added to form a dark orange suspension which was left to stir at room temperature for 1 hour to form a dark orange solution. The solvent was removed under vacuum to afford a dark-coloured microcrystalline solid which was then dissolved in a mixture of THF and pentane. The solution was left to slowly evaporate at −30 °C over a few days. Dark orange crystals were isolated, washed with cold pentane and dried *in vacuo* (106.6 mg, 73%).

^{1}H NMR (C_6D_6, 300 K) δ (ppm) = 63.96 [TMP CH_2, 4H], 60.06 [TMP CH_2, 2H], 43.71 [TMP CH_2, 2H], 35.50 [NCH_3, 3H], 25.75 [TMP CH_2, 2H], 25.75 [TMP *Me*, 12H], 20.34 [NCH_3, 3H], 5.67 [NCH_3, 3H], –50.78 [TMP *Me*, 12H]
Anal. Calcd for $C_{35}H_{63}Co_2N_7O_2$: C 57.45, H 8.68, N 13.40 Found: C 57.32, H 8.76, N 13.60
Solution Magnetic Moment (C_6D_6, 300 K) = 4.68 μ_B

[{PhN=(Ph)C}Co$_2$(TMP)$_3$] (3m)

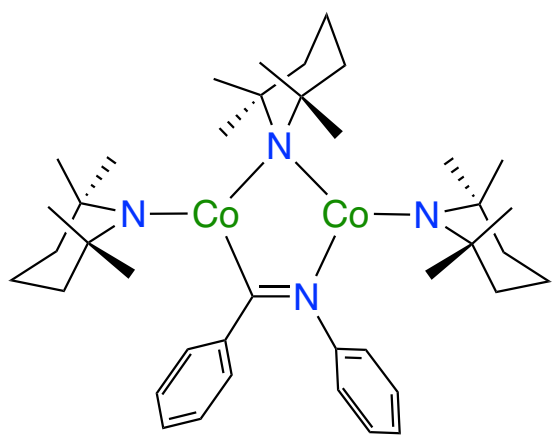

$Co(TMP)_2$ (67.9 mg, 0.2 mmol) was dissolved in pentane to give a dark red solution. *N*-Benzylideneaniline (18.1 mg, 0.1 mmol) was added to form a dark orange solution which was left to stir at room temperature for 1 hour. The solution was left to slowly evaporate at −30 °C over a few days. Dark orange crystals were isolated, washed with cold pentane and dried *in vacuo* (56.0 mg, 78%).

^{1}H NMR (C_6D_6, 300 K) δ (ppm) = 40.59, 34.00, 27.45, 19.80, 16.17, 16.11, 15.21, 10.54, 6.23, 5.58, 1.76, –2.31, –16.44, –35.67, –40.14
Anal. Calcd for $C_{40}H_{64}Co_2N_4$: C 66.83, H 8.97, N 7.79 Found: C 66.95, H 9.11, N 8.05
Solution Magnetic Moment (C_6D_6, 300 K) = 3.81 μ_B

[(MeO)Co$_2$(TMP)$_3$] (4)

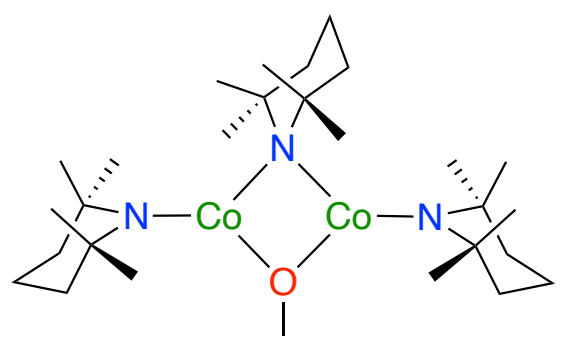

$Co(TMP)_2$ (67.9 mg, 0.2 mmol) was dissolved in pentane to give a dark red solution to which methyl picolinate (12.1 μL, 0.1 mmol) was subsequently added. The solution was left to stir at room temperature for 3 hour to give a dark orange solution. The solution was left to slowly evaporate at −30 °C over a few days. Dark orange crystals were isolated, washed with cold pentane and dried *in vacuo* (34.4 mg, 60%).

^{1}H NMR (C_6D_6, 300 K) δ (ppm) = 107.39, 71.46, 60.70, 48.23, 43.99, 42.94, 32.24, 26.47, 19.82, 12.22, 10.45, 7.42, –39.56.
Anal. Calcd for $C_{28}H_{57}Co_2N_3O$: C 59.04, H 10.09, N 7.38 Found: C 59.00, H 9.81, N 7.66
Solution Magnetic Moment (C_6D_6, 300 K) = 3.94 μ_B

[{(Ph)(TMP)C=N}Co$_2$(TMP)$_3$] (5)

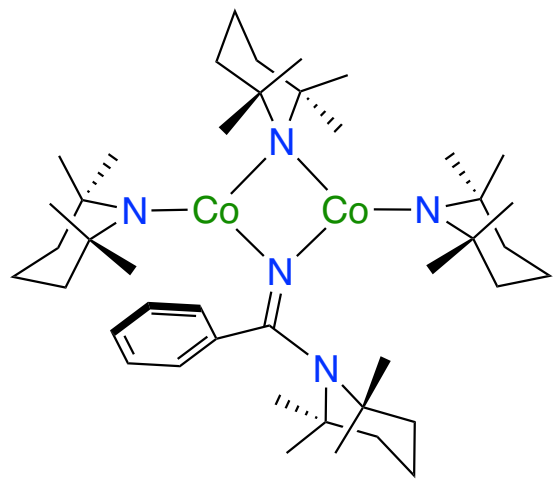

Co(TMP)$_2$ (67.9 mg, 0.2 mmol) was dissolved in pentane to give a dark red solution to which benzonitrile (10.3 μL, 0.1 mmol) was subsequently added. The solution was left to stir at room temperature for 1 hour to give a dark orange solution. The solution was left to slowly evaporate at −30 °C over a few days. Dark green crystals were isolated, washed with cold pentane and dried *in vacuo* (69.0 mg, 88%).

^{1}H NMR (C_6D_6, 300 K) δ (ppm) = 23.07, 15.97, 8.86, 8.51, 3.70, –2.86, –18.90
Anal. Calcd for $C_{31}H_{57}Co_2N_5$: C 66.05, H 9.93, N 8.96 Found: C 65.92, H 10.02, N 9.10
Solution Magnetic Moment (C_6D_6, 300 K) = 4.07 μ_B

[(2,5-C$_4$H$_2$N$_2$-pyz)Co$_4$(TMP)$_6$] (6a)

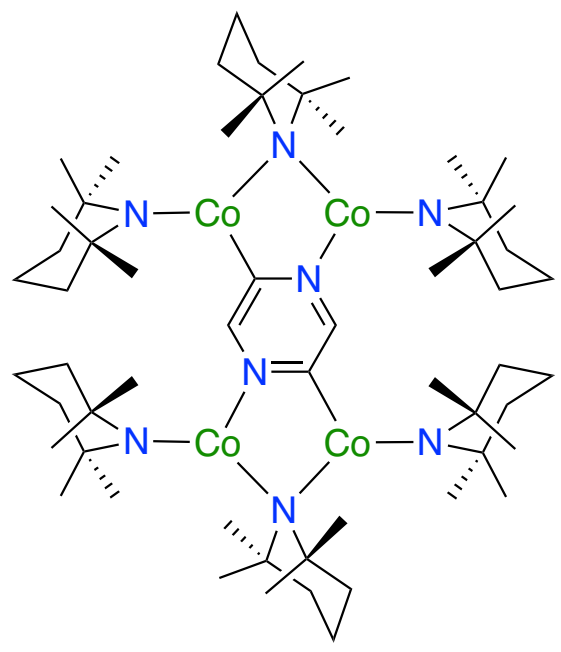

Co(TMP)$_2$ (135.8 mg, 0.4 mmol) was dissolved in benzene to give a dark red solution to which pyrazine (8.0 mg, 0.1 mmol) was subsequently added. The solution was left to stir at 70 °C for 4 hours to give a dark orange solution. The solution was left to slowly evaporate at −30 °C over a few days. Dark orange crystals were isolated, washed with cold pentane and dried *in vacuo* (98.0 mg, 85%).

^{1}H NMR (C_6D_6, 300 K) δ (ppm) = 126.26 [Pyrazinyl C*H*, 2H], 32.55 [TMP CH_2, 12H], 22.39 [TMP CH_2, 8H], 18.43 [TMP *Me*, 24H], 17.10 [TMP CH_2, 8H], 12.72 [TMP CH_2, 8H], –25.45 [TMP *Me*, 24H]
^{1}H NMR (d_8-THF, 300 K) δ (ppm) = 127.24 [Pyrazinyl C*H*, 2H], 32.77 [TMP CH_2, 12H], 22.76 [TMP CH_2, 8H], 18.50 [TMP *Me*, 24H], 17.28 [TMP CH_2, 8H], 12.67 [TMP CH_2, 8H], –26.38 [TMP *Me*, 24H]
Anal. Calcd for $C_{58}H_{110}Co_4N_8$: C 60.30, H 9.60, N 9.70 Found: C 60.11, H 9.62, N 9.31
Solution Magnetic Moment (d_8-THF, 300 K) = 4.29 μ_B

[(4,6-C$_4$H$_2$N$_2$-pym)Co$_4$(TMP)$_6$] (6b)

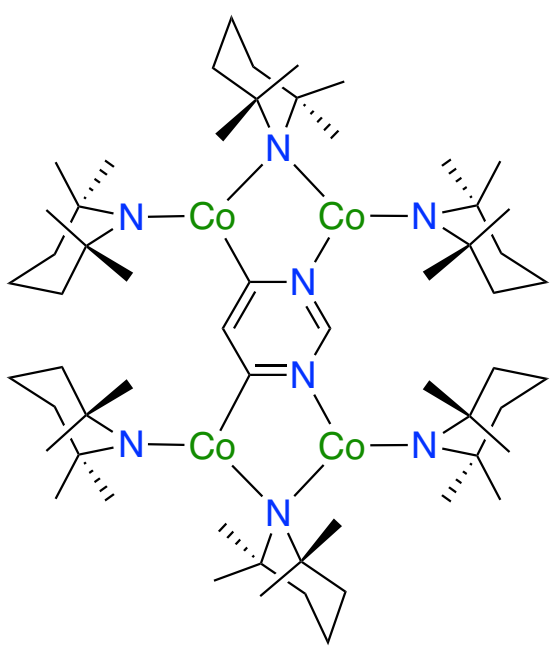

Co(TMP)$_2$ (135.8 mg, 0.4 mmol) was dissolved in benzene to give a dark red solution to which pyrimidine (7.9 μL, 0.1 mmol) was subsequently added. The solution was left to stir at 70 °C for 4 hours to give a dark orange solution. The solution was left to slowly evaporate at −30 °C over a few days. Dark orange crystals were isolated, washed with cold pentane and dried *in vacuo* (89.9 mg, 78%).

^{1}H NMR (C_6D_6, 300 K) δ (ppm) = 132.68 [Pyrimidinyl C*H*, 1H], 126.21 [Pyrimidinyl C*H*, 1H], 33.42, 24.11, 22.53, 18.96, –25.90
Anal. Calcd for $C_{58}H_{110}Co_4N_8$: C 60.30, H 9.60, N 9.70 Found: C 60.01, H 9.55, N 9.42
Solution Magnetic Moment could not be determined due to low solubility in C_6D_6 or d_8-THF.

[(4-C$_4$H$_3$N$_2$-pym)Co$_2$(TMP)$_3$] (7b)

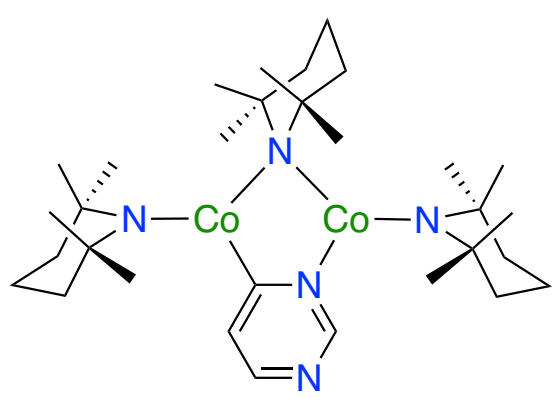

Co(TMP)$_2$ (67.9 mg, 0.2 mmol) was dissolved in pentane to give a dark red solution to which pyrimidine (7.9 μL, 0.1 mmol) was subsequently added at −30 °C. The solution was left to stir at room temperature for 2 hours to give a dark orange solution. The solution was left to slowly evaporate at −30 °C over a few days. Dark orange crystals were isolated, washed with cold pentane and dried *in vacuo* (29.8 mg, 48%).

^{1}H NMR (C_6D_6, 300 K) δ (ppm) = 79.95 [Pyrimidinyl C*H*, 1H], 76.20 [Pyrimidinyl C*H*, 1H], 72.68 [Pyrimidinyl C*H*, 1H], 35.39, 24.38, 19.02, –27.54

Anal. Calcd for $C_{31}H_{57}Co_2N_5$: C 60.28, H 9.30, N 11.34 Found: C 60.94, H 9.03, N 11.77

Solution Magnetic Moment (C_6D_6, 300 K) = 3.74 μ_B

[(3-$C_4H_3N_2$-pdz)Co_2(TMP)$_3$] (7c)

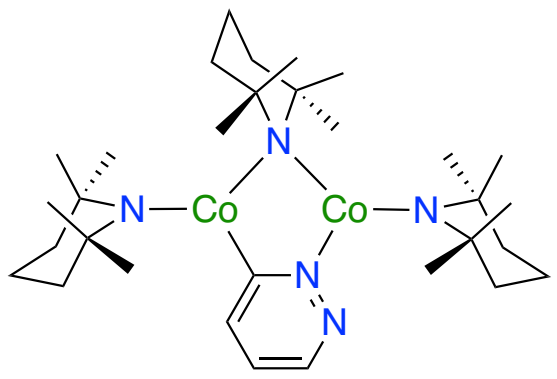

Co(TMP)$_2$ (67.9 mg, 0.2 mmol) was dissolved in pentane to give a dark red solution to which pyridazine (7.3 µL, 0.1 mmol) was subsequently added at −30 °C. The solution was left to stir at room temperature for 2 hours to give a dark orange solution. The solution was left to slowly evaporate at −30 °C over a few days. Dark orange crystals were isolated, washed with cold pentane and dried *in vacuo* (30.2 mg, 49%).

^{1}H NMR (C_6D_6, 300 K) δ (ppm) = 76.24 [Pyridazinyl C*H*, 1H], 60.26 [Pyridazinyl C*H*, 1H], 52.93 [Pyridazinyl C*H*, 1H], 33.80 [TMP C*H*$_2$, 6H], 23.89 [TMP C*H*$_2$, 2H], 22.43 [TMP C*H*$_2$, 2H], 17.78 [TMP *Me*, 12H], 9.58 [TMP C*H*$_2$, 4H], –28.21 [TMP *Me*, 12H]

Anal. Calcd for $C_{31}H_{57}Co_2N_5$: C 60.28, H 9.30, N 11.34 Found: C 59.65, H 9.13, N 11.60

Solution Magnetic Moment (C_6D_6, 300 K) = 3.61 μ_B

[(2-Quinoxalinyl)Co_2(TMP)$_3$] (7d)

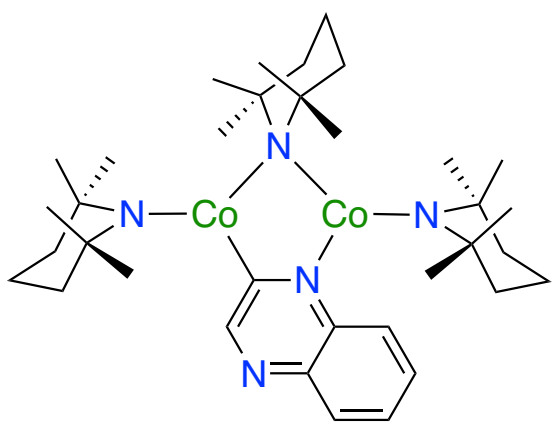

Co(TMP)$_2$ (67.9 mg, 0.2 mmol) was dissolved in pentane to give a dark red solution to which quinoxaline (13.0 mg, 0.1 mmol) was subsequently added at −30 °C. The solution was left to stir at room temperature for 2 hours to give a dark red solution. The solution was left to slowly evaporate at −30 °C over a few days. Dark red crystals were isolated, washed with cold pentane and dried *in vacuo* (50.5 mg, 76%).

^{1}H NMR (C_6D_6, 300 K) δ (ppm) = 53.74 [Quinoxalinyl C*H*, 1H], 45.02 [Quinoxalinyl C*H*, 1H], 28.12 [TMP C*H*$_2$, 2H], 27.73 [TMP C*H*$_2$, 4H], 21.82 [Quinoxalinyl C*H*, 1H], 19.54 [TMP C*H*$_2$, 2H], 17.91 [Quinoxalinyl C*H*, 1H], 17.67 [TMP C*H*$_2$, 2H], 14.09 [Quinoxalinyl C*H*, 1H], 12.52 [TMP *Me*, 12H], 12.52 [TMP C*H*$_2$, 4H], 6.41 [TMP C*H*$_2$, 4H], –23.25 [TMP *Me*, 12H]

Anal. Calcd for $C_{35}H_{59}Co_2N_5$: C 62.95, H 8.91, N 10.49 Found: C 63.56, H 9.19, N 10.43

Solution Magnetic Moment (C_6D_6, 300 K) = 3.66 μ_B

[(4-Quinazolinyl)Co_2(TMP)$_3$] (7e)

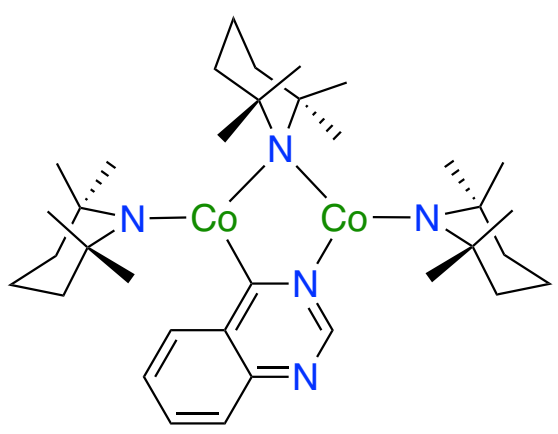

Co(TMP)$_2$ (67.9 mg, 0.2 mmol) was dissolved in pentane to give a dark red solution to which quinazoline (13.0 mg, 0.1 mmol) was subsequently added at −30 °C. The solution was left to stir at room temperature for 2 hours to give a dark orange solution. The solution was left to slowly evaporate at −30 °C over a few days. Dark orange crystals were isolated, washed with cold pentane and dried *in vacuo* (35.0 mg, 52%).

^{1}H NMR (C_6D_6, 300 K) δ (ppm) = 73.61 [Quinazolinyl C*H*, 1H], 51.34 [Quinazolinyl C*H*, 1H], 43.65 [TMP C*H*$_2$, 2H], 41.64 [Quinazolinyl C*H*, 1H], 34.40 [TMP C*H*$_2$, 3H], 30.39 [TMP C*H*$_2$, 3H], 26.95 [Quinazolinyl C*H*, 1H], 22.41 [TMP *Me*, 6H], 18.67 [TMP *Me*, 6H], 15.85 [TMP C*H*$_2$, 2H], 13.67 [Quinazolinyl C*H*, 1H], 4.88 [TMP C*H*$_2$, 2H], –32.18 [TMP *Me*, 12H]

Anal. Calcd for $C_{35}H_{59}Co_2N_5$: C 62.95, H 8.91, N 10.49 Found: C 63.04, H 8.97, N 10.35

Solution Magnetic Moment (C_6D_6, 300 K) = 3.74 μ_B

2-(2,2,6,6-Tetramethylpiperidin-1-yl)pyridine (8a)

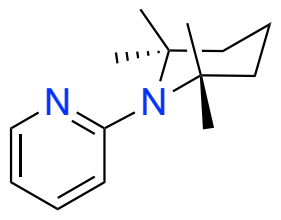

$Co(TMP)_2$ (135.8 mg, 0.4 mmol) was dissolved in benzene to give a dark red solution to which pyridine (16.1 µL, 0.2 mmol) was subsequently added. The solution was left to stir at 70 °C for 4 hours to give a dark orange solution. A solution of I_2 (253.8 mg, 1 mmol) in THF was added and the mixture was left to stir at room temperature for 16 h. The reaction was quenched with saturated $Na_2S_2O_3$ and NH_4Cl aqueous solutions. Saturated $NaHCO_3$ aqueous solution was added until the mixture turned basic. The mixture was washed with brine and extracted with Et_2O. The combined organic layers were dried over anhydrous $MgSO_4$ and concentrated *in vacuo*. Pure compound was isolated by column chromatography on silica gel (0% to 10% EtOAc/hexane) to afford **8a** as a yellow oil (9.3 mg, 21%). ^{1}H and ^{13}C NMR spectroscopic data are in good agreement with literature values.[6]

^{1}H NMR (300 MHz, $CDCl_3$) δ (ppm) = 8.48 (dd, *J* = 4.8, 0.8 Hz, 1H), 7.54 (td, *J* = 7.6, 2.1 Hz, 1H), 7.09 – 7.04 (m, 1H), 7.02 (d, *J* = 7.9 Hz, 1H), 1.82 – 1.68 (m, 2H), 1.59 – 1.51 (m, 4H), 1.09 (s, 12H).
^{13}C NMR (75 MHz, $CDCl_3$) δ (ppm) = 161.13, 148.04, 136.30, 128.88, 120.75, 54.50, 41.84, 29.43, 18.40.

N.B. Instead of I_2 (2,5 eq), *N*-iodosuccinimide (2.5 eq) or 5-(trifluoromethyl)dibenzothiophenium trifluoromethanesulfonate (0.5 eq) could be used as oxidant to generate compound **8a** in 63 and 46% NMR yields respectively against 10 mol% internal standard of C_6Me_6.

2-Phenyl-6-(2,2,6,6-tetramethylpiperidin-1-yl)pyridine (8b)

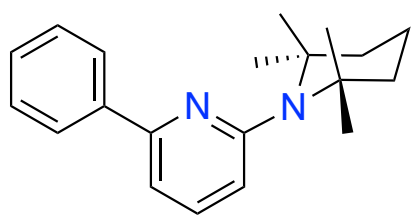

$Co(TMP)_2$ (135.8 mg, 0.4 mmol) was dissolved in benzene to give a dark red solution to which 2-phenylpyridine (28.6 µL, 0.2 mmol) was subsequently added. The solution was left to stir at 70 °C for 3 hours to give a dark orange solution. A solution of I_2 (253.8 mg, 1 mmol) in THF was added and the mixture was left to stir at room temperature for 16 h. The reaction was quenched with saturated $Na_2S_2O_3$ and NH_4Cl aqueous solutions. Saturated $NaHCO_3$ aqueous solution was added until the mixture turned basic. The mixture was washed with brine and extracted with Et_2O. The combined organic layers were dried over anhydrous $MgSO_4$ and concentrated *in vacuo*. Pure compound was isolated by column chromatography on silica gel (0% to 10% EtOAc/hexane) to afford **8b** as a yellow oil (37.1 mg, 63%).

^{1}H NMR (300 MHz, $CDCl_3$) δ (ppm) = 8.14 – 8.04 (m, 2H), 7.67 – 7.53 (m, 2H), 7.51 – 7.43 (m, 2H), 7.42 – 7.33 (m, 1H), 6.92 (dd, *J* = 6.4, 2.2 Hz, 1H), 1.86 – 1.72 (m, 2H), 1.63 – 1.52 (m, 4H), 1.17 (s, 12H).
^{13}C NMR (75 MHz, $CDCl_3$) δ (ppm) = 160.89, 154.99, 139.80, 136.93, 128.76, 128.66, 127.25, 126.75, 116.69, 54.52, 41.74, 29.42, 18.42.
HRMS: ESI $[M+H]^+$ $C_{20}H_{27}N_2^+$ Calc. 295.2169 Found 295.2167.

2,5-bis(2,2,6,6-tetramethylpiperidin-1-yl)pyrazine (8c)

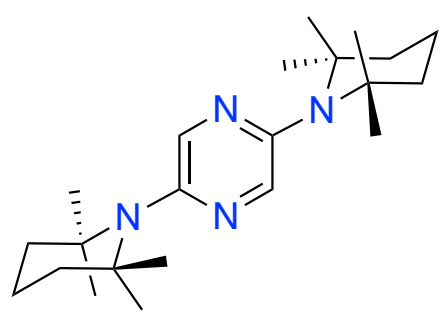

$Co(TMP)_2$ (271.5 mg, 0.8 mmol) was dissolved in benzene to give a dark red solution to which pyrazine (16.0 mg, 0.2 mmol) was subsequently added. The solution was left to stir at 70 °C for 4 hours to give a dark orange solution. A solution of I_2 (253.8 mg, 1 mmol) in THF was added and the mixture was left to stir at room temperature for 16 h. The reaction was quenched with saturated $Na_2S_2O_3$ and NH_4Cl aqueous solutions. Saturated $NaHCO_3$ aqueous solution was added until the mixture turned basic. The mixture was washed with brine and extracted with Et_2O. The combined organic layers were dried over anhydrous $MgSO_4$ and concentrated *in vacuo*. The crude was washed with cold acetone and filtered to afford **8c** as a white solid (26.6 mg, 37%).

^{1}H NMR (300 MHz, $CDCl_3$) δ (ppm) = 8.12 (s, 2H), 1.83 – 1.69 (m, 4H), 1.61 – 1.51 (m, 8H), 1.08 (s, 24H).
^{13}C NMR (75 MHz, $CDCl_3$) δ (ppm) = 153.64, 146.81, 54.74, 41.44, 29.37, 18.28.
HRMS: ESI $[M+H]^+$ $C_{22}H_{39}N_4^+$ Calc. 359.3169 Found 359.3164.

4-(2,2,6,6-Tetramethylpiperidin-1-yl)pyrimidine (8d)

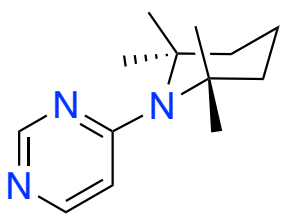

Co(TMP)$_2$ (135.8 mg, 0.4 mmol) was dissolved in benzene to give a dark red solution to which pyrimidine (15.8 μL, 0.2 mmol) was subsequently added. The solution was left to stir at room temperature for 2 hours to give a dark orange solution. A solution of I_2 (253.8 mg, 1 mmol) in THF was added and the mixture was left to stir at room temperature for 16 h. The reaction was quenched with saturated $Na_2S_2O_3$ and NH_4Cl aqueous solutions. Saturated $NaHCO_3$ aqueous solution was added until the mixture turned basic. The mixture was washed with brine and extracted with Et_2O. The combined organic layers were dried over anhydrous $MgSO_4$ and concentrated *in vacuo*. Pure compound was isolated by column chromatography on silica gel (50% EtOAc/hexane) to afford **8d** as a yellow oil (14.7 mg, 34%).

^{1}H NMR (300 MHz, $CDCl_3$) δ (ppm) = 8.81 (s, 1H), 8.29 (dd, *J* = 5.9, 0.7 Hz, 1H), 6.82 (dd, *J* = 5.9, 1.3 Hz, 1H), 1.88 – 1.66 (m, 6H), 1.35 (s, 12H).

^{13}C NMR (75 MHz, $CDCl_3$) δ (ppm) = 165.91, 157.67, 154.60, 118.37, 55.79, 38.59, 29.78, 16.02.

HRMS: ESI $[M+H]^+$ $C_{13}H_{22}N_3^+$ Calc. 220.1808 Found 220.1807.

NMR Spectra of Numbered Compounds

[(py)Fe(TMP)$_2$] (2^{Fe})

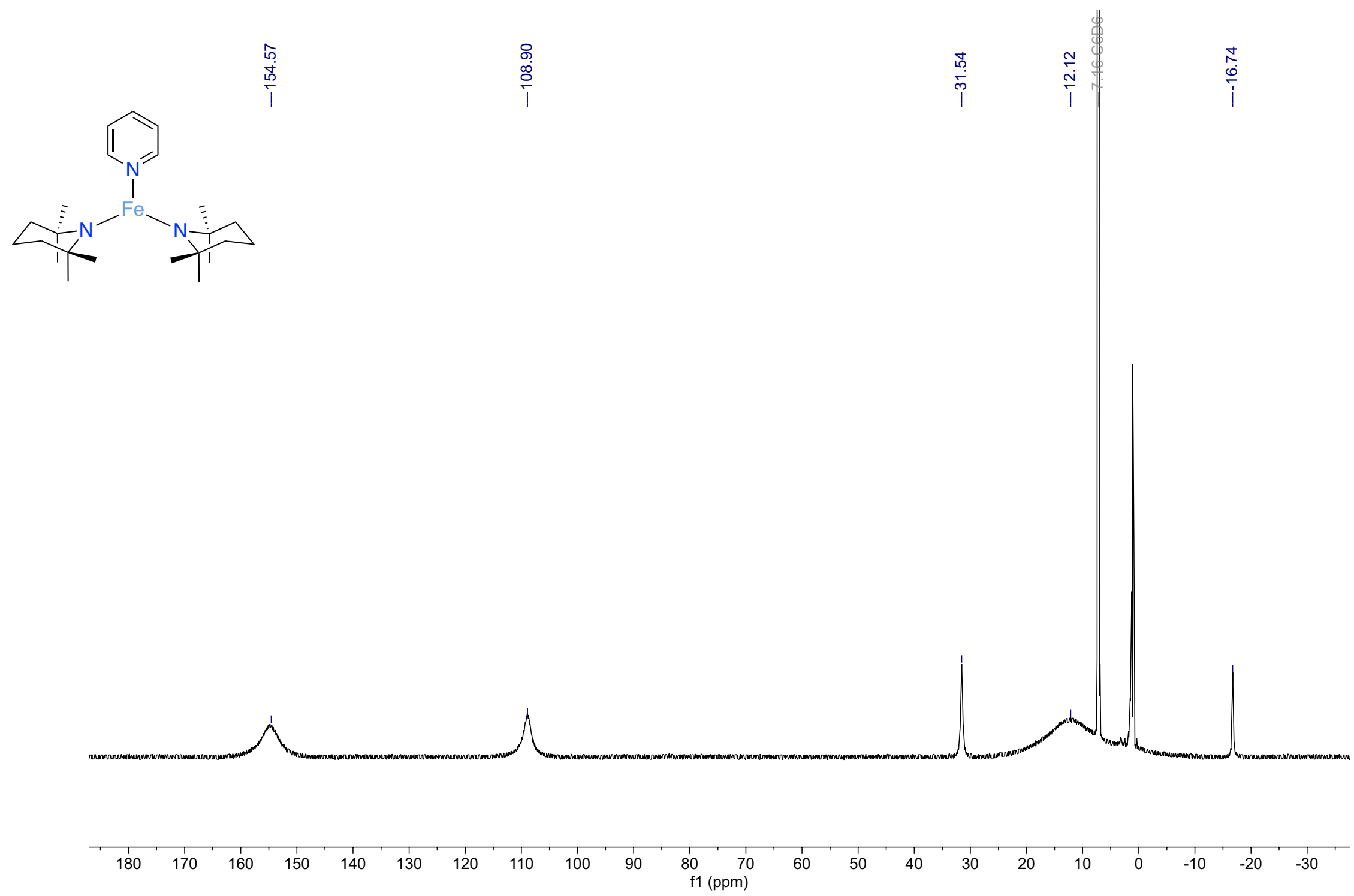

Figure S1. ^{1}H NMR spectrum of $\mathbf{2^{Fe}}$ in C_6D_6.

**δ (^{1}H) / ppm**	**Assignment**
154.57	/
108.90	/
31.54	/
12.12	/
–16.74	/

[(py)Co(TMP)$_2$] (2a)

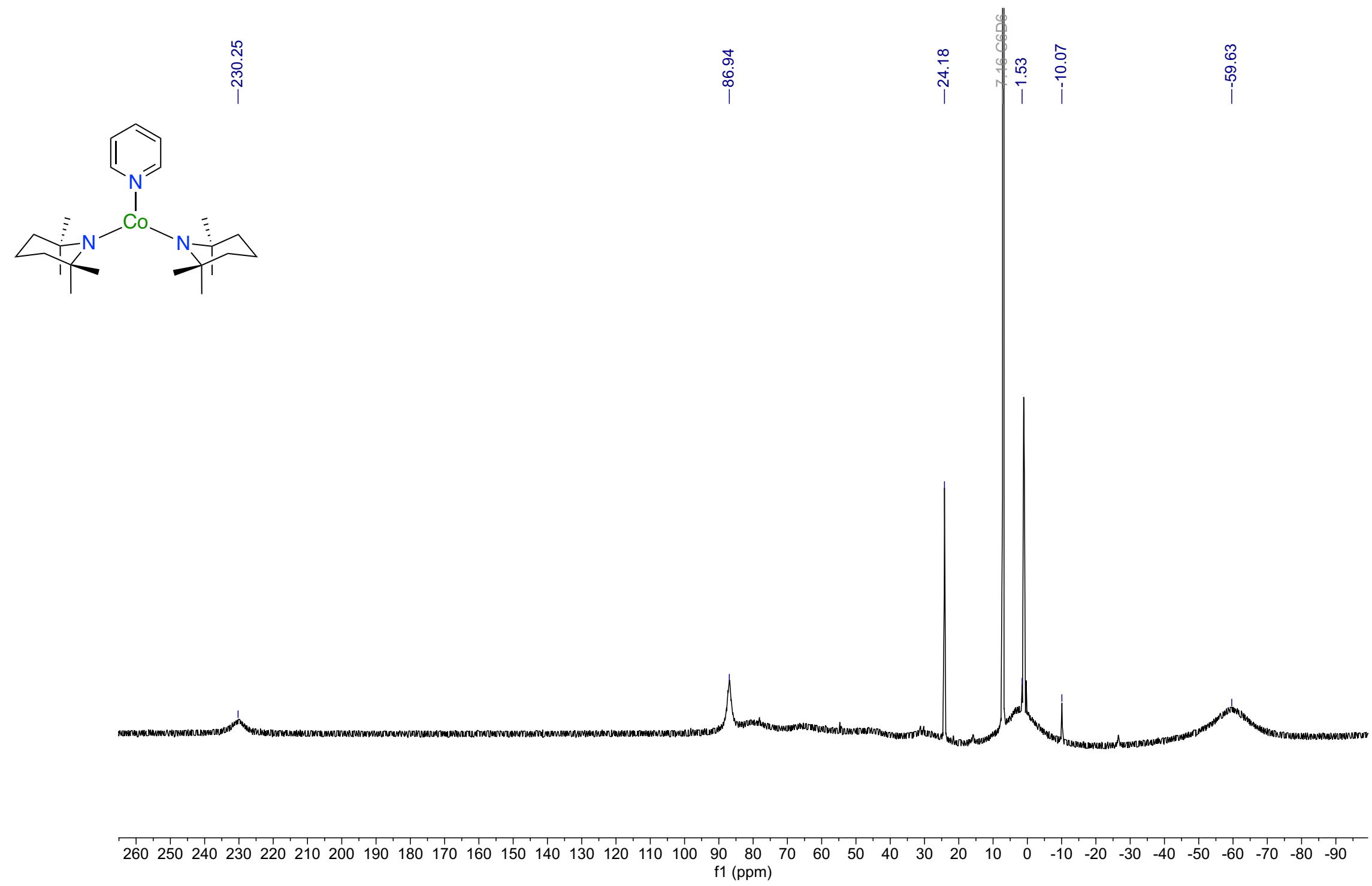

Figure S2. ^{1}H NMR spectrum of **2a** in C_6D_6.

**δ (^{1}H) / ppm**	**Assignment**
230.25	/
86.94	/
24.18	/
1.53	/
–10.07	/
–59.63	/

[(2-Methoxy-py)Co(TMP)$_2$] (2f)

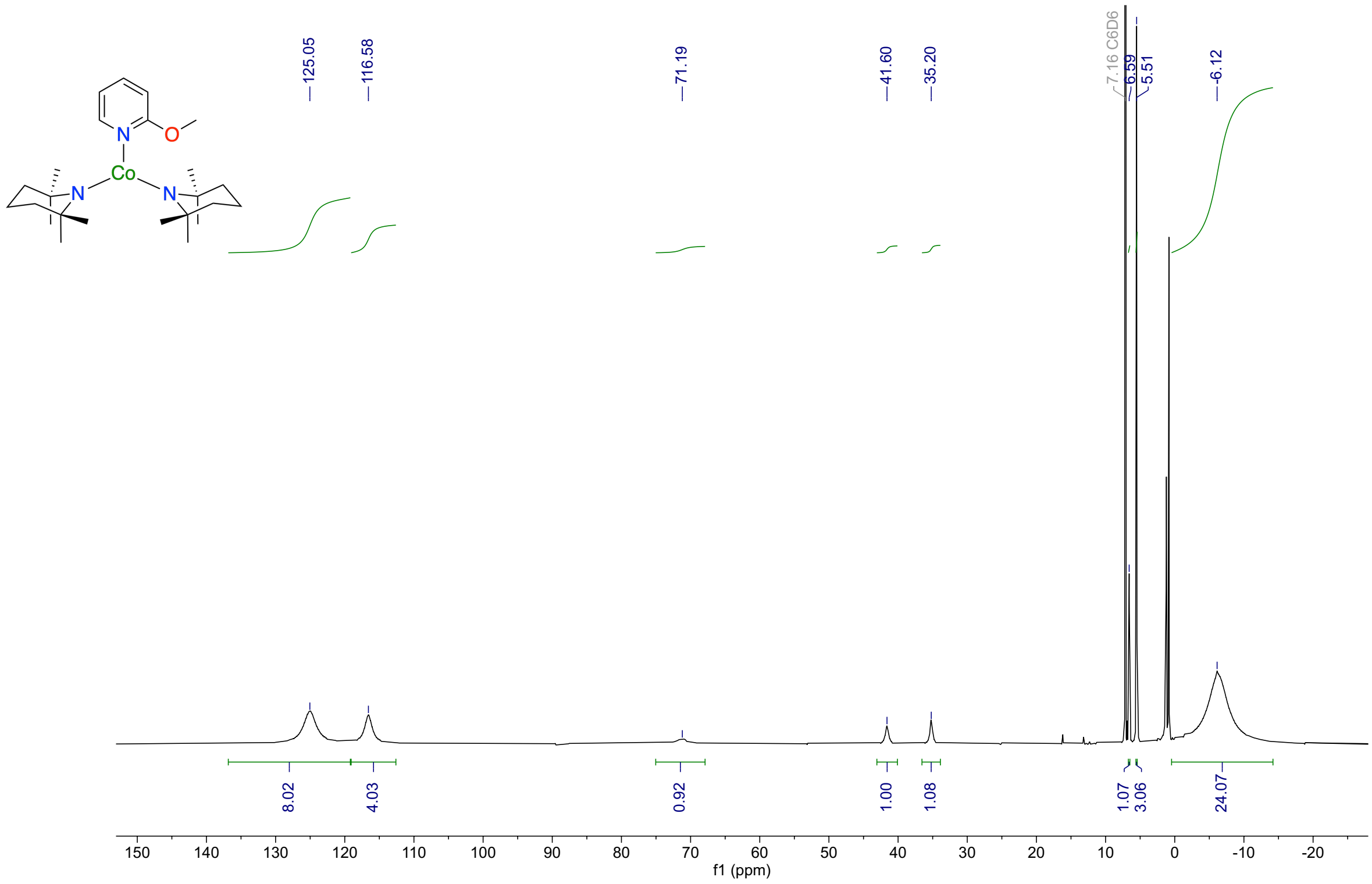

Figure S3. ^{1}H NMR spectrum of **2f** in C_6D_6.

**δ (^{1}H) / ppm**	**Assignment**
125.05	TMP C*H*$_2$ (8H)
116.58	TMP C*H*$_2$ (4H)
71.19	Pyridyl C*H* (1H)
41.60	Pyridyl C*H* (1H)
35.20	Pyridyl C*H* (1H)
6.59	Pyridyl C*H* (1H)
5.51	Methoxy O*Me* (3H)
–6.12	TMP *Me* (12H)

[(2-Pyridinyl)$Co_2(TMP)_3$] (3a)

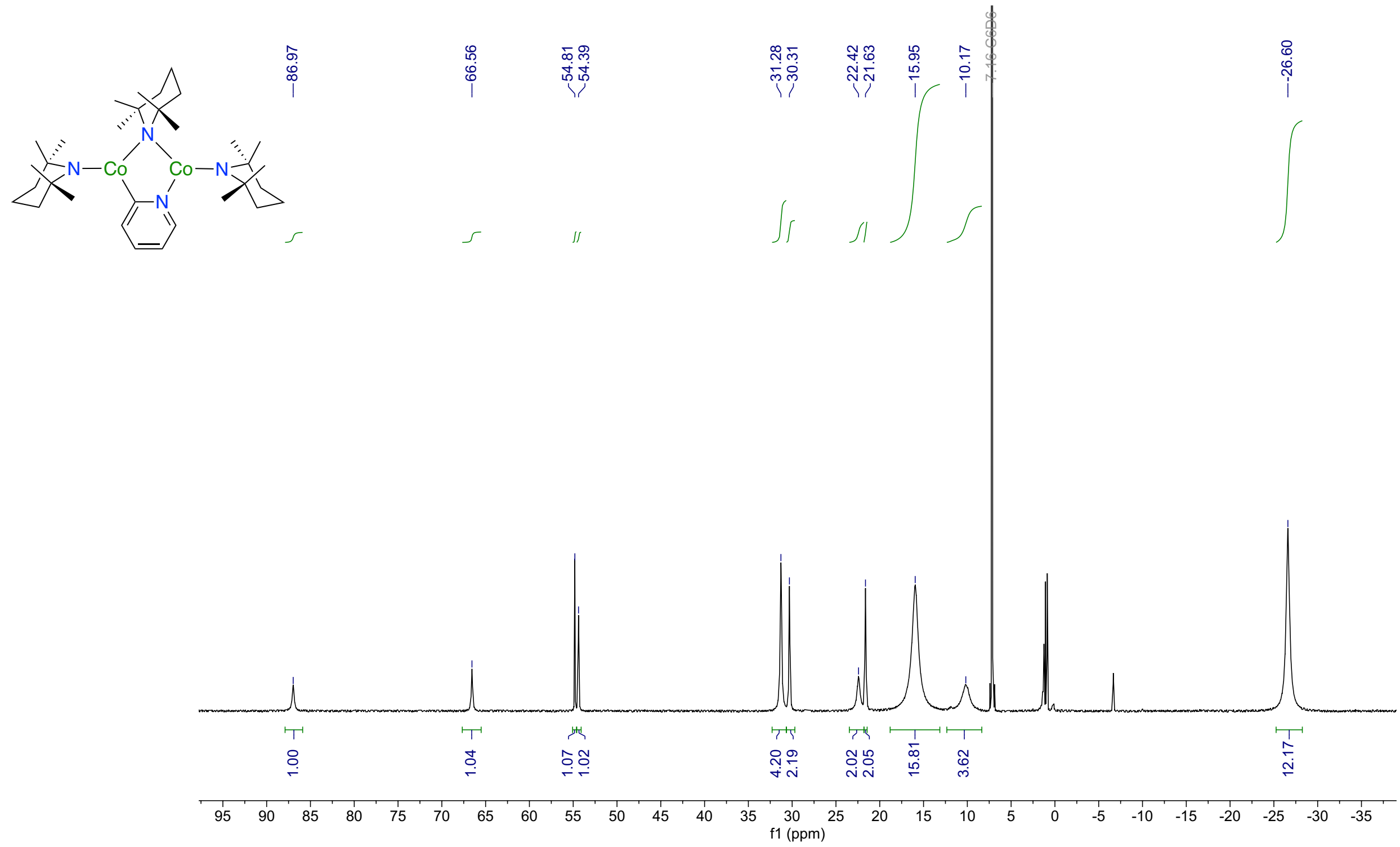

Figure S4. ^{1}H NMR spectrum of **3a** in C_6D_6.

δ (^{1}H) / ppm	Assignment
86.97	Pyridyl C*H* (1H)
66.56	Pyridyl C*H* (1H)
54.81	Pyridyl C*H* (1H)
54.39	Pyridyl C*H* (1H)
31.28	TMP CH_2 (4H)
30.31	TMP CH_2 (2H)
22.42	TMP CH_2 (2H)
21.63	TMP CH_2 (2H)
15.95	TMP *Me* (12H)
15.95	TMP CH_2 (4H)
10.17	TMP CH_2 (4H)
–26.60	TMP *Me* (12H)

[(6-Phenyl-2-pyridinyl)Co_2(TMP)$_3$] (3b)

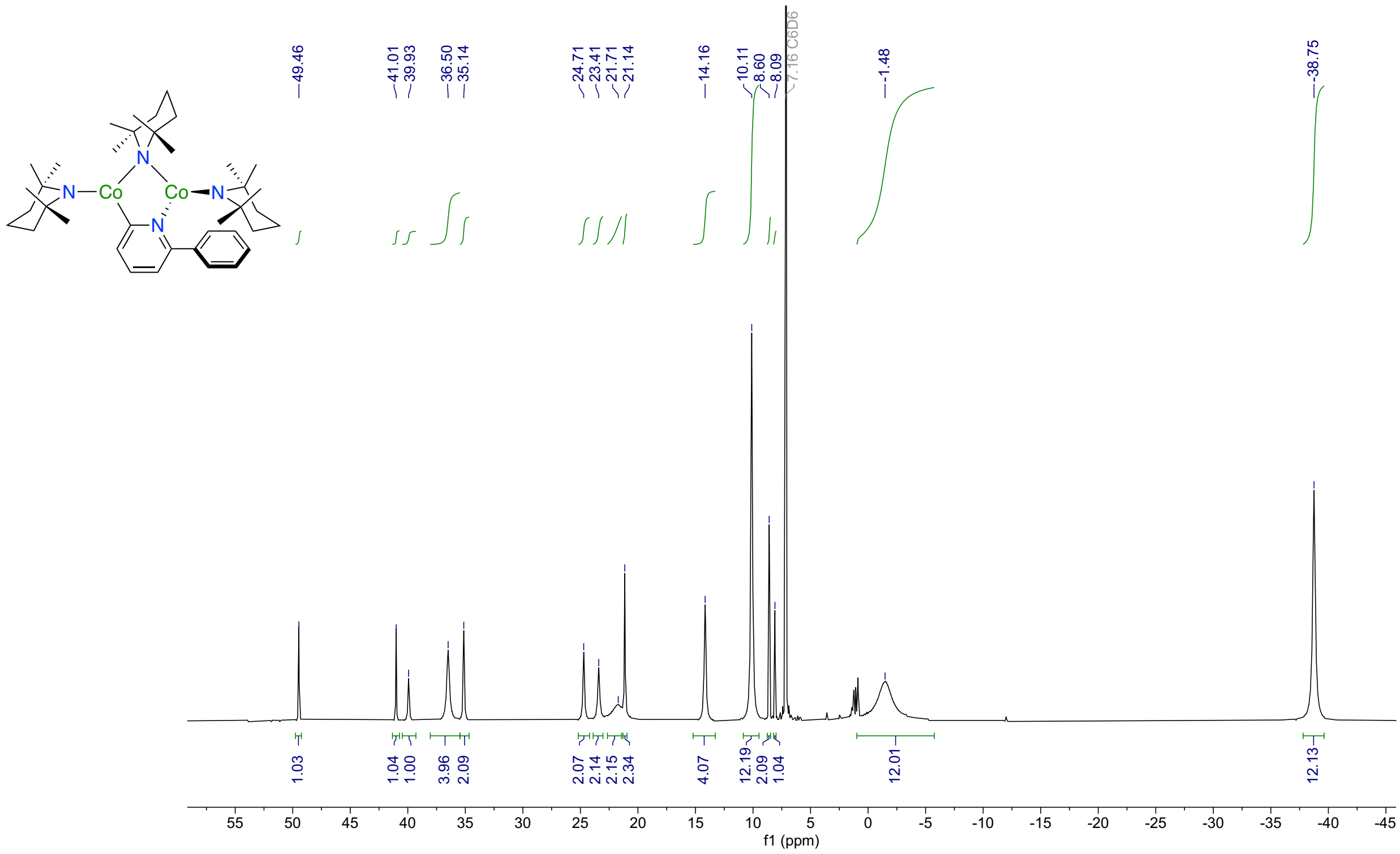

Figure S5. ^{1}H NMR spectrum of **3b** in C_6D_6.

**δ (^{1}H) / ppm**	**Assignment**
49.46	Pyridyl C*H* (1H)
41.01	Pyridyl C*H* (1H)
39.93	Pyridyl C*H* (1H)
36.50	TMP CH_2 (4H)
35.14	TMP CH_2 (2H)
24.71	TMP CH_2 (2H)
23.41	TMP CH_2 (2H)
21.71	TMP CH_2 (2H)
21.14	TMP CH_2 (2H)
14.16	TMP CH_2 (4H)
10.11	TMP *Me* (12H)
8.60	Phenyl C*H* (2H)
8.09	Phenyl C*H* (1H)
–1.48	TMP *Me* (12H)
–38.75	TMP *Me* (12H)

[(2-$CH_2C_5H_4N$)Co_2(TMP)$_3$] (3c)

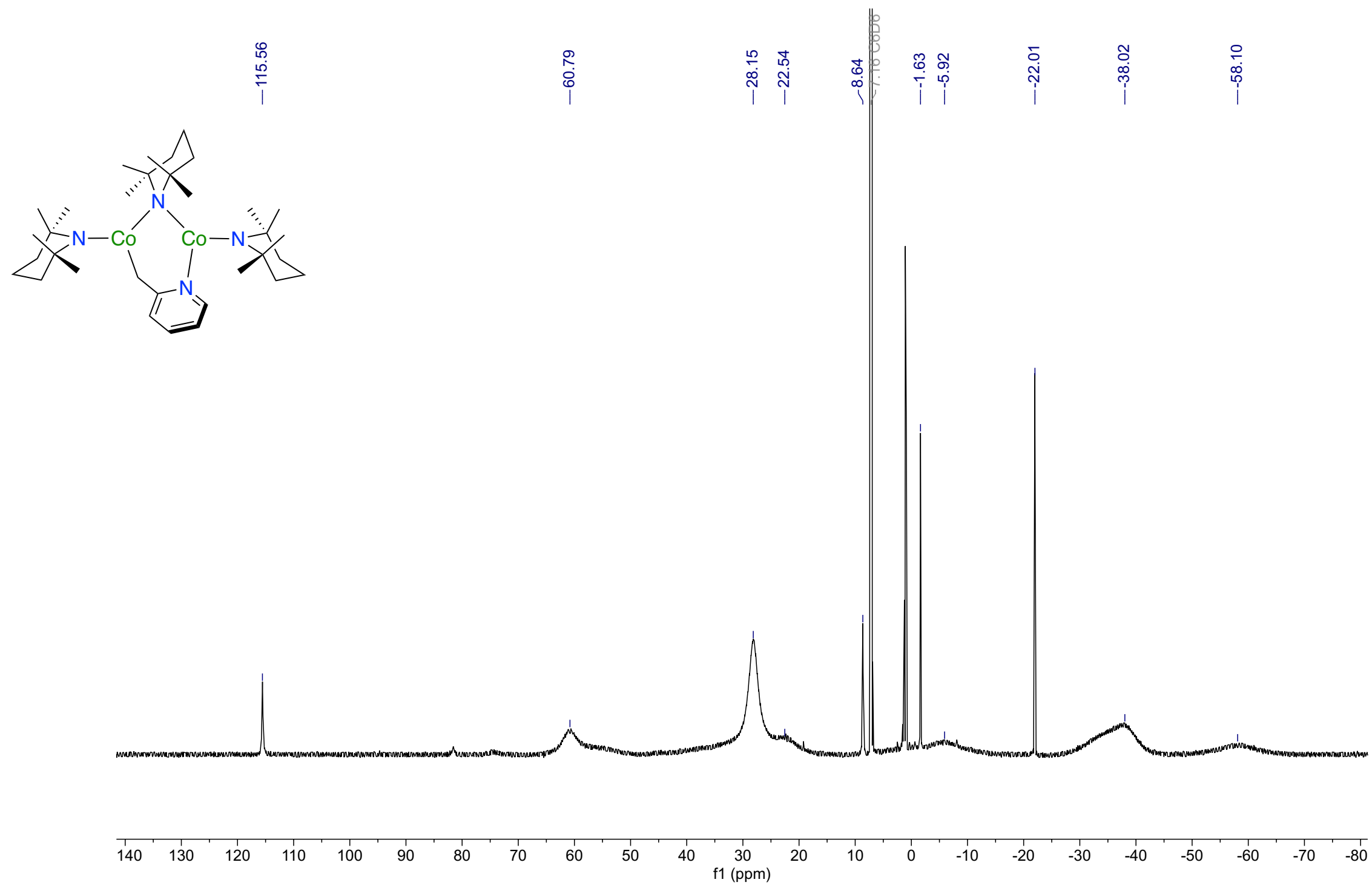

Figure S6. ^{1}H NMR spectrum of **3c** in C_6D_6.

δ (^{1}H) / ppm	Assignment
115.56	/
60.79	/
28.15	/
22.54	/
8.64	/
–1.63	/
–5.92	/
–22.01	/
–38.02	/
–58.10	/

[(4-Methyl-2-pyridinyl)$Co_2(TMP)_3$] (3d)

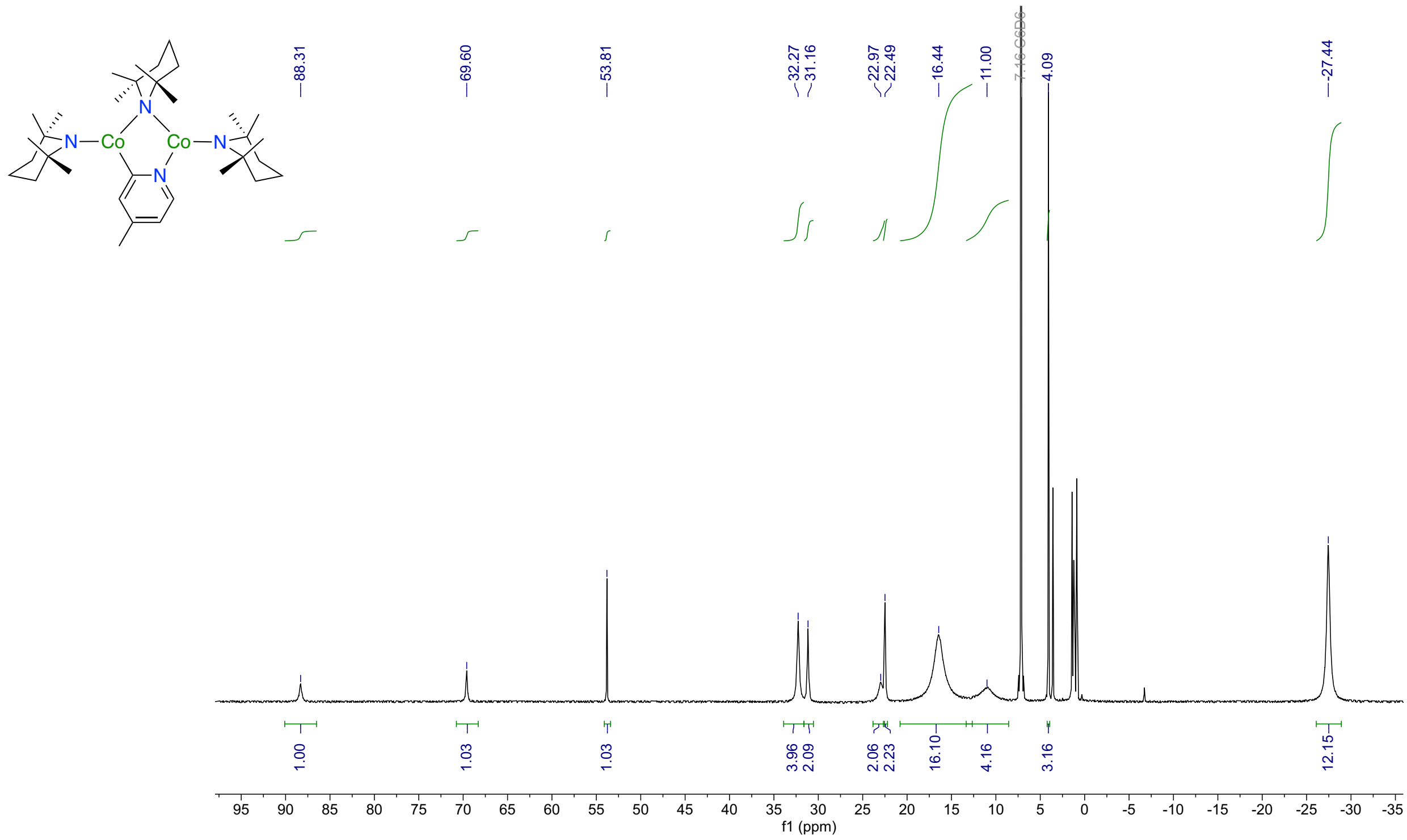

Figure S7. ^1H NMR spectrum of **3d** in C_6D_6.

**δ (^1H) / ppm**	**Assignment**
88.31	Pyridyl C*H* (1H)
69.60	Pyridyl C*H* (1H)
53.81	Pyridyl C*H* (1H)
32.27	TMP C*H*$_2$ (4H)
31.16	TMP C*H*$_2$ (2H)
22.97	TMP C*H*$_2$ (2H)
22.49	TMP C*H*$_2$ (2H)
16.44	TMP *Me* (12H)
16.44	TMP C*H*$_2$ (4H)
11.00	TMP C*H*$_2$ (4H)
4.09	Picolyl *Me* (3H)
–27.44	TMP *Me* (12H)

[(6-Methoxy-2-pyridinyl)Co_2(TMP)$_3$] (3f)

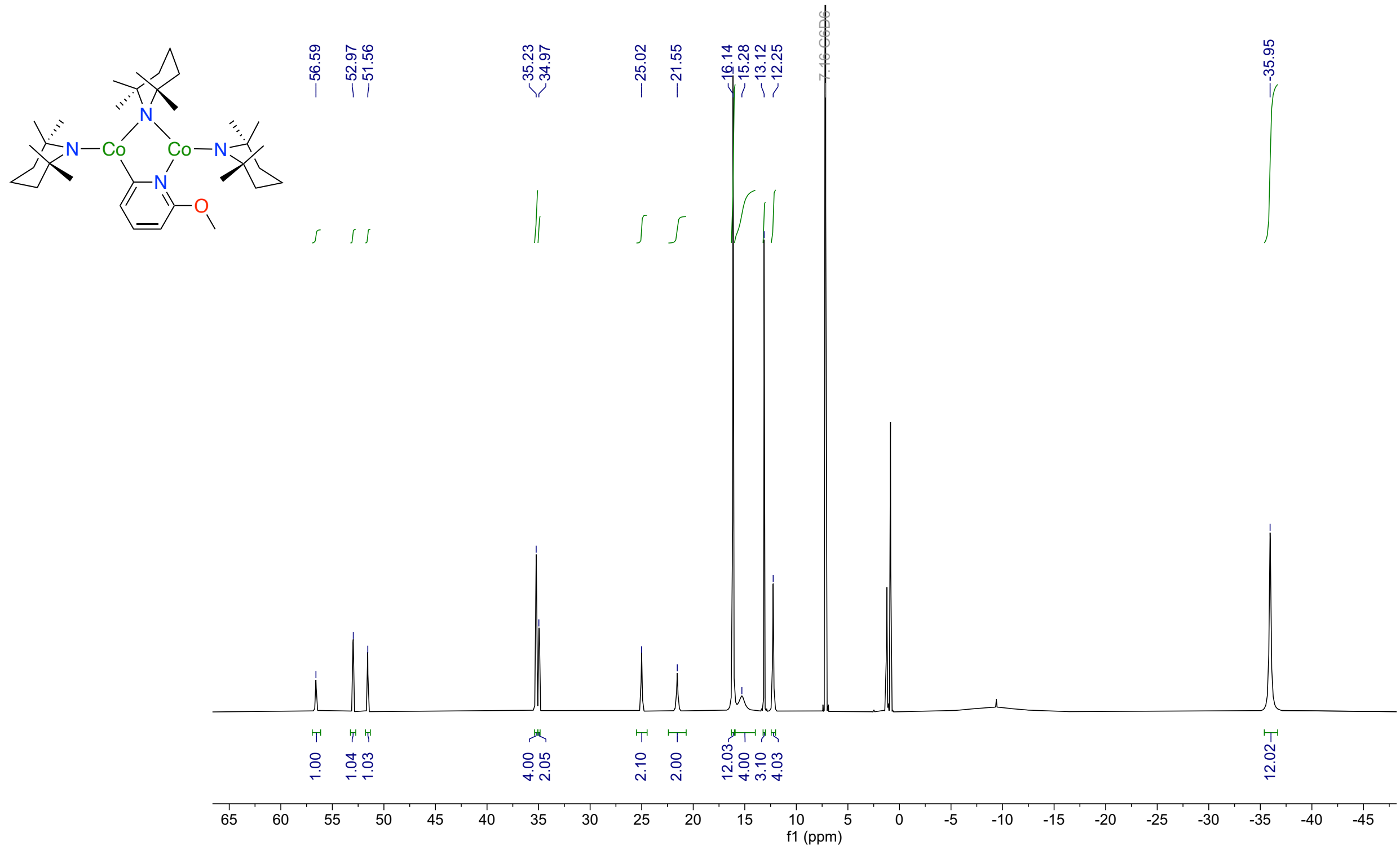

Figure S8. ^{1}H NMR spectrum of **3f** in C_6D_6.

**δ (^{1}H) / ppm**	**Assignment**
56.59	Pyridyl C*H* (1H)
52.97	Pyridyl C*H* (1H)
51.56	Pyridyl C*H* (1H)
35.23	TMP CH_2 (4H)
34.97	TMP CH_2 (2H)
25.02	TMP CH_2 (2H)
21.55	TMP CH_2 (2H)
16.14	TMP *Me* (12H)
15.28	TMP CH_2 (4H)
13.12	Methoxy O*Me* (3H)
12.25	TMP CH_2 (4H)
–35.95	TMP *Me* (12H)

[(4-Methoxy-2-pyridinyl)Co_2(TMP)$_3$] (3g)

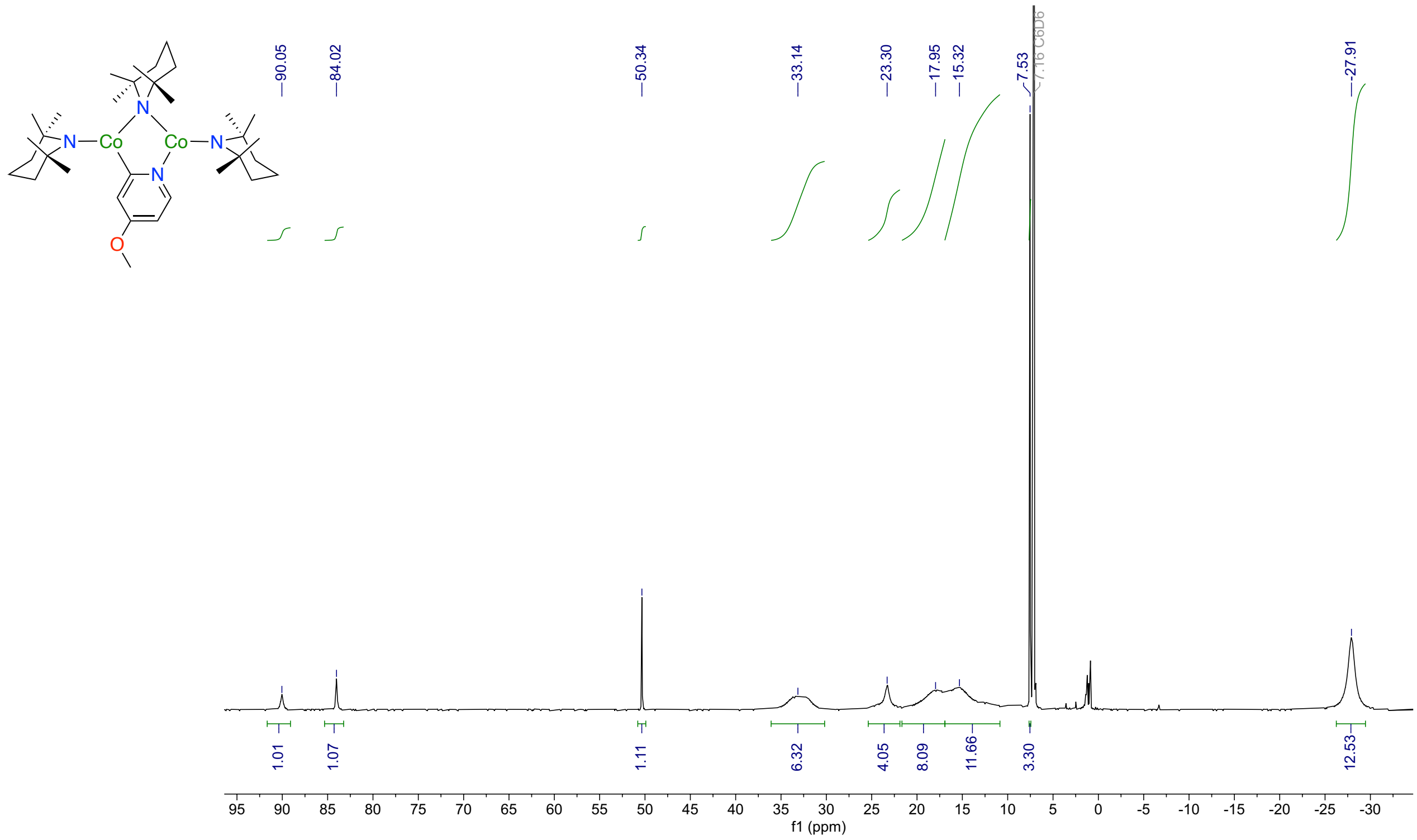

Figure S9. ^{1}H NMR spectrum of **3g** in C_6D_6.

**δ (^{1}H) / ppm**	**Assignment**
90.05	Pyridyl C*H* (1H)
84.02	Pyridyl C*H* (1H)
50.34	Pyridyl C*H* (1H)
33.14	TMP C*H*$_2$ (6H)
23.30	TMP C*H*$_2$ (4H)
17.95	TMP C*H*$_2$ (8H)
15.32	TMP *Me* (12H)
7.53	Methoxy O*Me* (3H)
–27.91	TMP *Me* (12H)

[(3-Methoxy-2-pyridinyl)Co_2(TMP)$_3$] (3h)

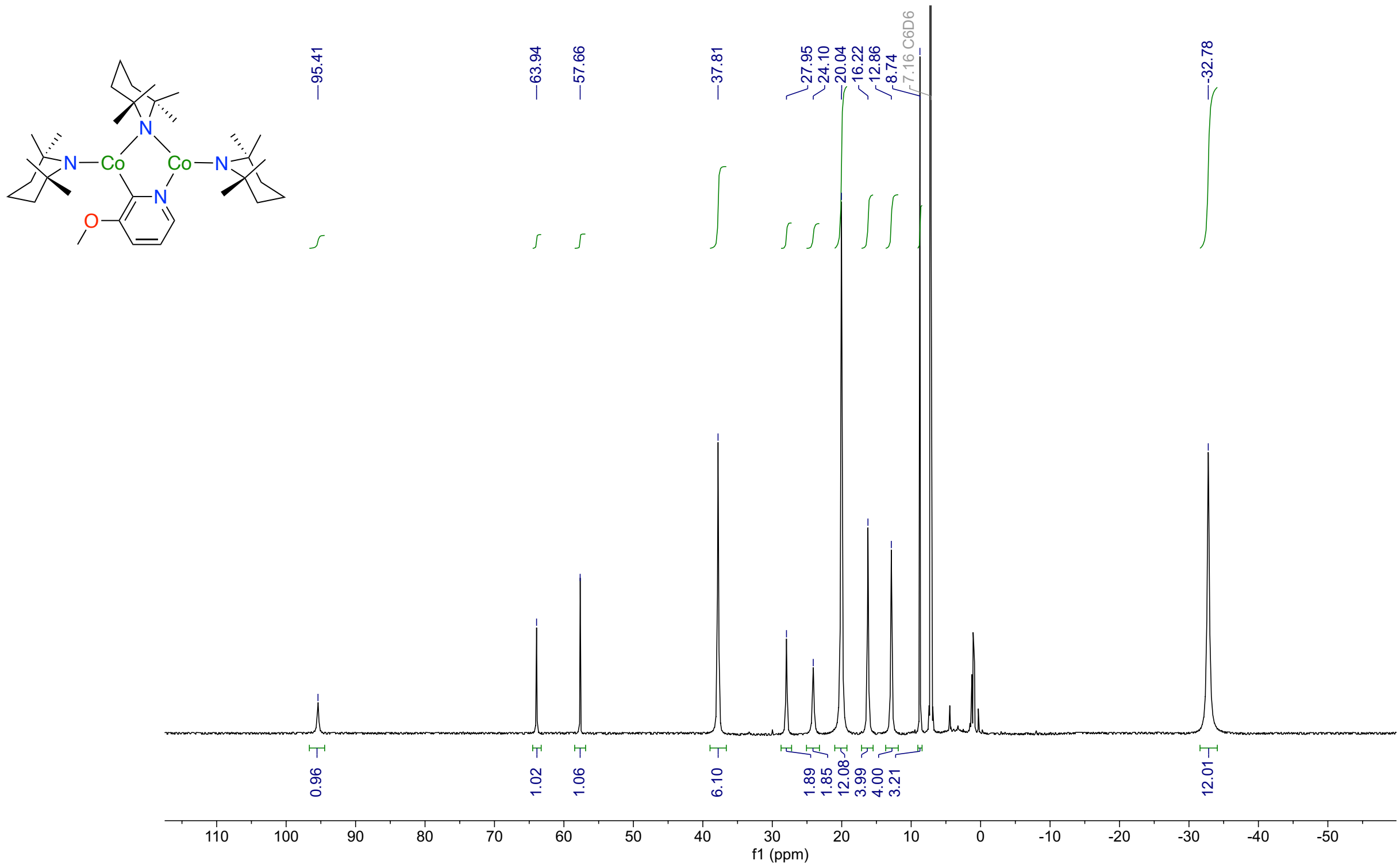

Figure S10. ^{1}H NMR spectrum of **3h** in C_6D_6.

**δ (^{1}H) / ppm**	**Assignment**
95.41	Pyridyl C*H* (1H)
63.94	Pyridyl C*H* (1H)
57.66	Pyridyl C*H* (1H)
37.81	TMP C*H*$_2$ (6H)
27.95	TMP C*H*$_2$ (2H)
24.10	TMP C*H*$_2$ (2H)
20.04	TMP *Me* (12H)
16.22	TMP C*H*$_2$ (4H)
12.86	TMP C*H*$_2$ (4H)
8.74	Methoxy O*Me* (3H)
–32.78	TMP *Me* (12H)

[(6-Fluoro-2-pyridinyl)$Co_2(TMP)_3$] (3i)

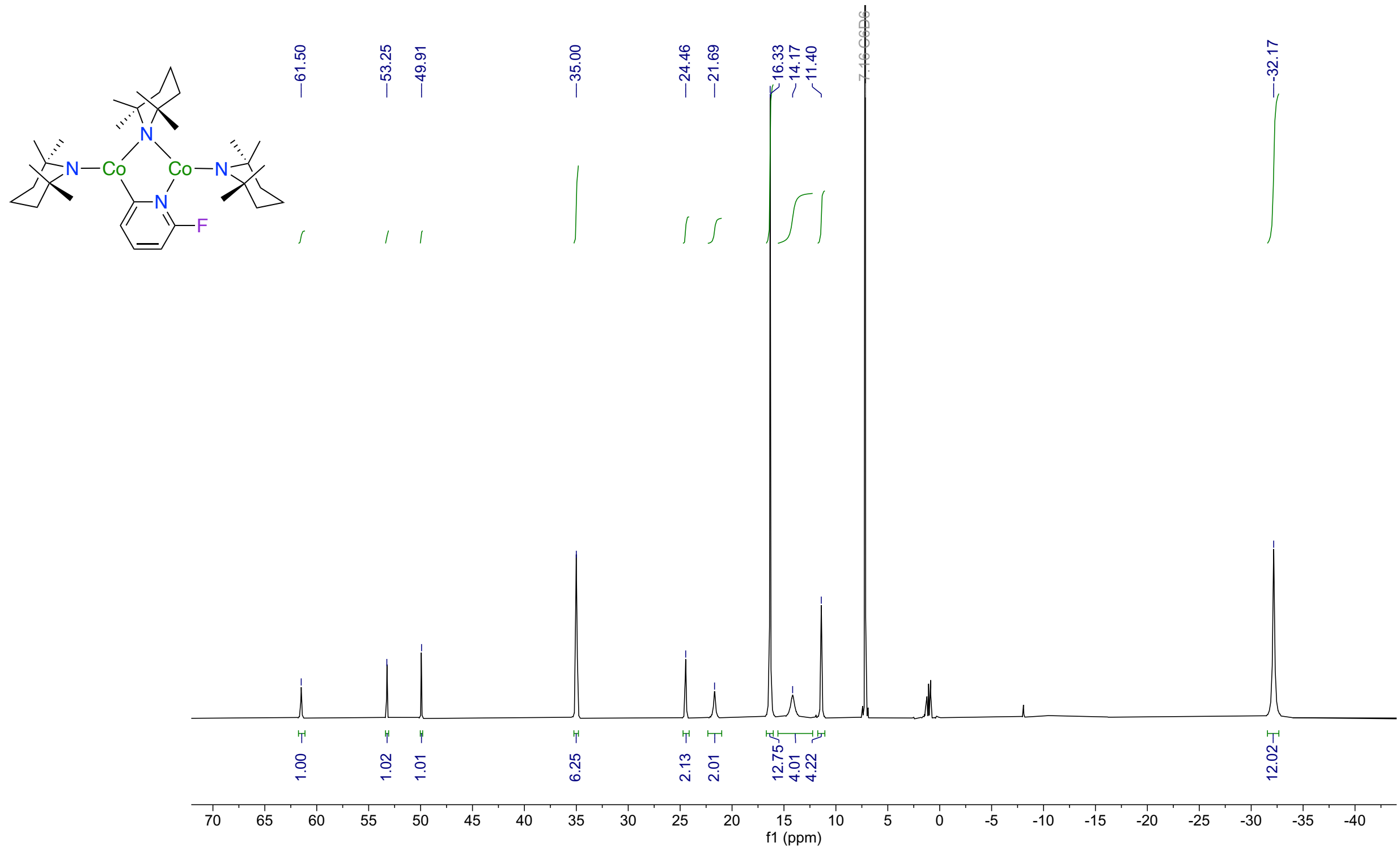

Figure S11. ^{1}H NMR spectrum of **3i** in C_6D_6.

**δ (^{1}H) / ppm**	**Assignment**
61.50	Pyridyl C*H* (1H)
53.25	Pyridyl C*H* (1H)
49.91	Pyridyl C*H* (1H)
35.00	TMP C*H*$_2$ (6H)
24.46	TMP C*H*$_2$ (2H)
21.69	TMP C*H*$_2$ (2H)
16.33	TMP *Me* (12H)
14.17	TMP C*H*$_2$ (4H)
11.40	TMP C*H*$_2$ (4H)
–32.17	TMP *Me* (12H)

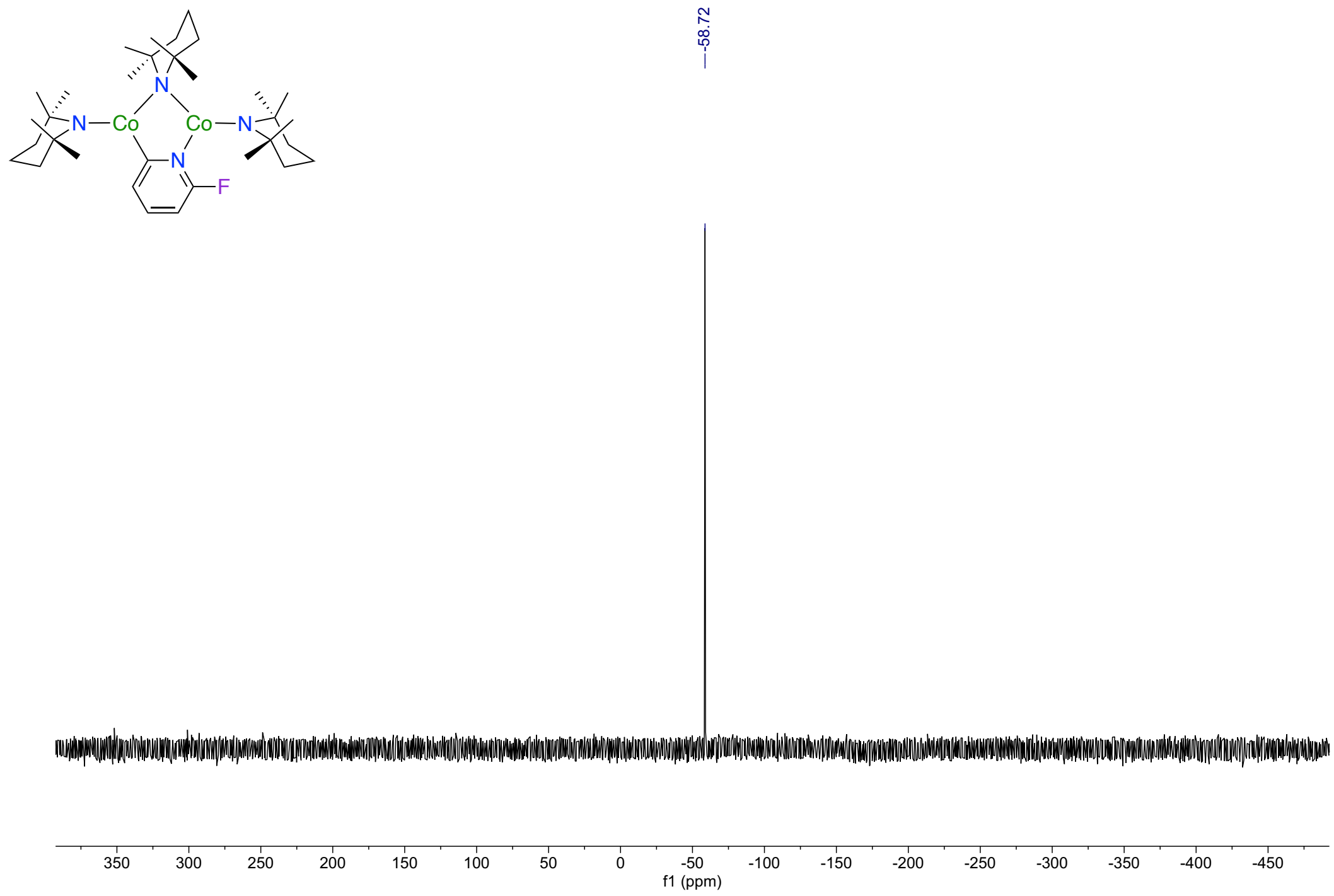

Figure S12. $^{19}F\{^{1}H\}$ NMR spectrum of **3i** in C_6D_6.

**δ (^{19}F) / ppm**	**Assignment**
–58.72	*Ortho F*

[(4,6-Difluoro-2-pyridinyl)$Co_2(TMP)_3$] (3j)

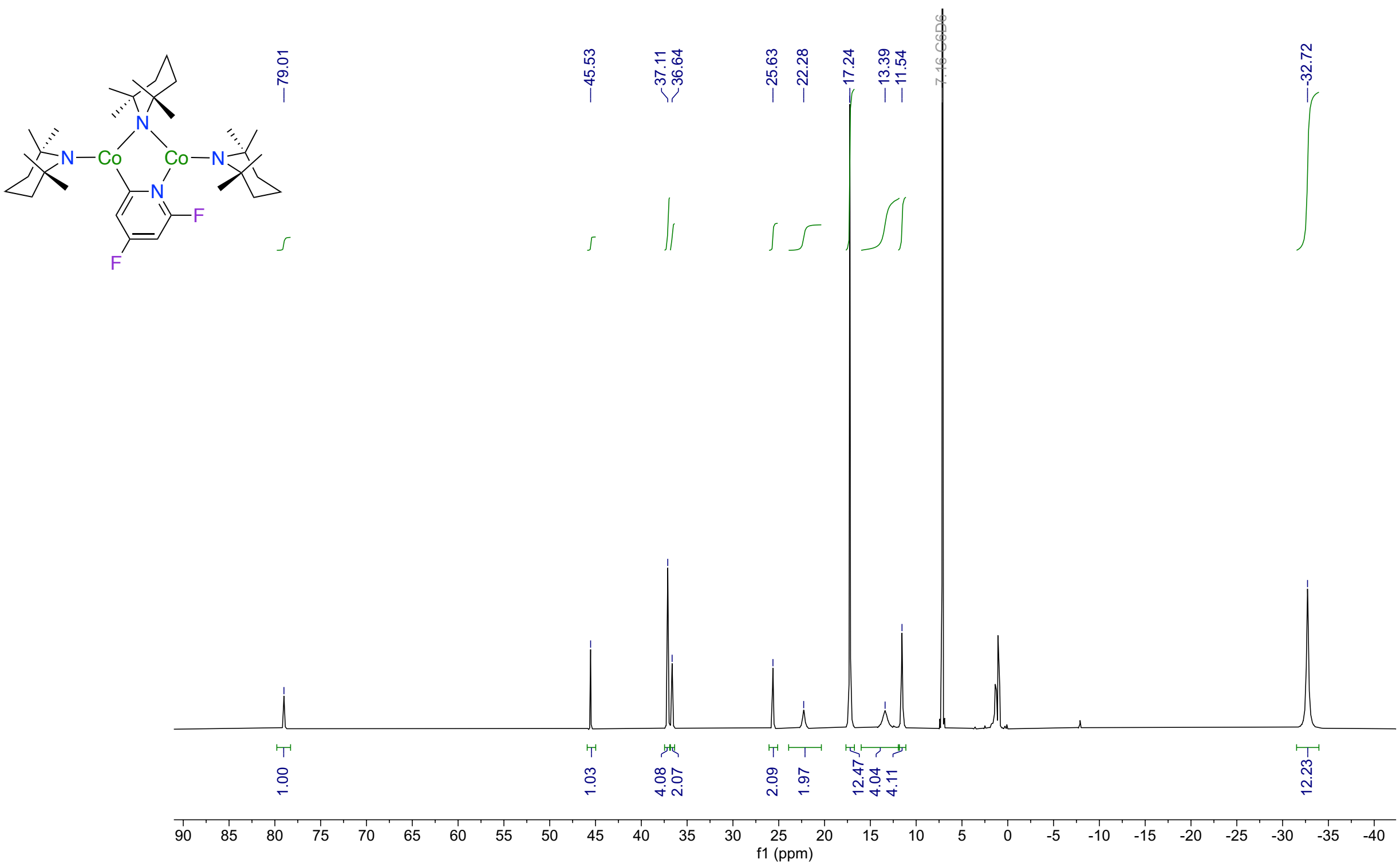

Figure S13. ^{1}H NMR spectrum of **3j** in C_6D_6.

**δ (^{1}H) / ppm**	**Assignment**
79.01	Pyridyl C*H* (1H)
45.53	Pyridyl C*H* (1H)
37.11	TMP CH_2 (4H)
36.64	TMP CH_2 (2H)
25.63	TMP CH_2 (2H)
22.28	TMP CH_2 (2H)
17.24	TMP *Me* (12H)
13.39	TMP CH_2 (4H)
11.54	TMP CH_2 (4H)
–32.72	TMP *Me* (12H)

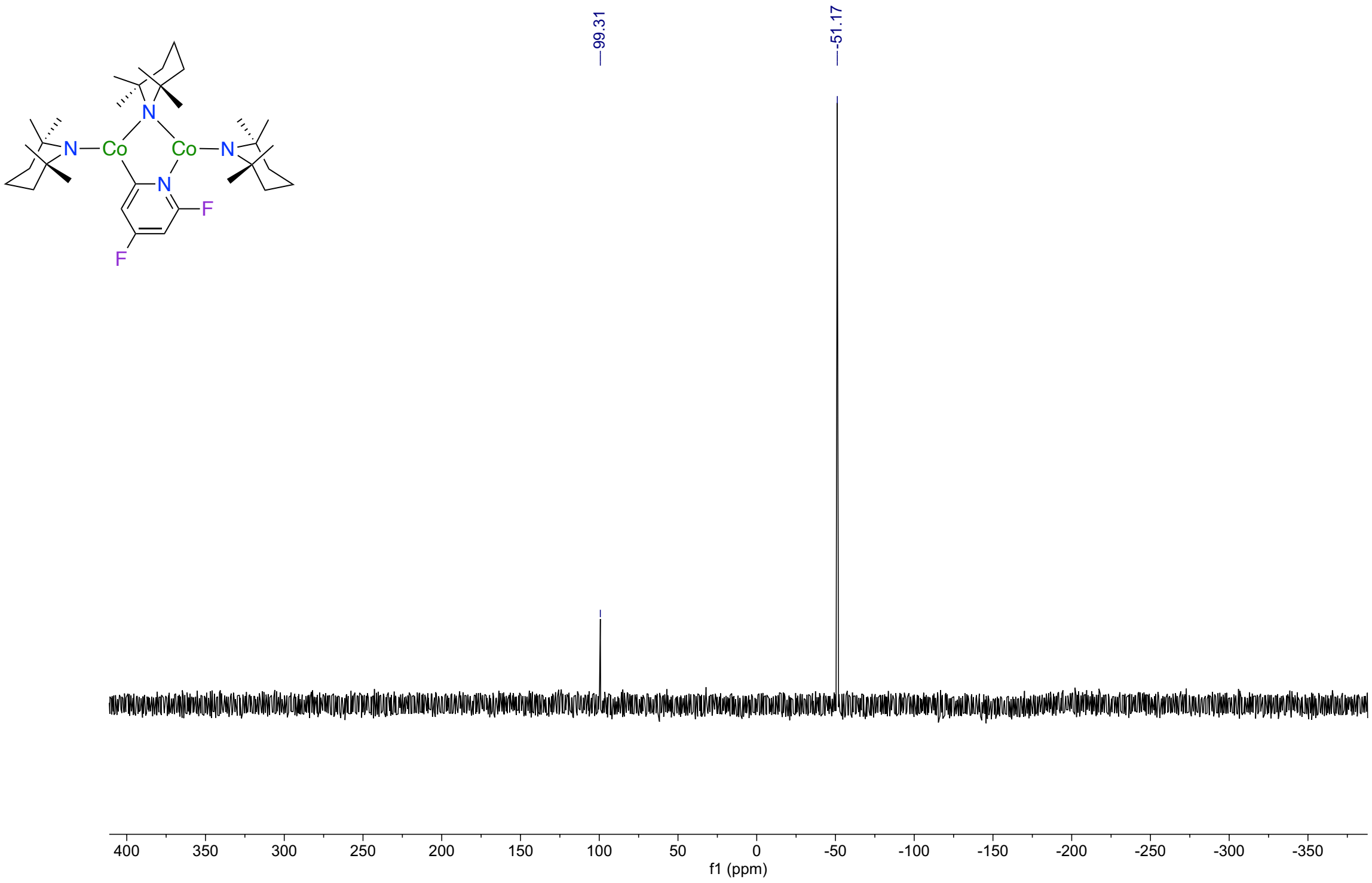

Figure S14. $^{19}F\{^1H\}$ NMR spectrum of **3j** in C_6D_6.

**δ (^{1}H) / ppm**	**Assignment**
99.31	*Para F*
–51.17	*Ortho F*

[(3-Fluoro-2-pyridinyl)$Co_2(TMP)_3$] (3k)

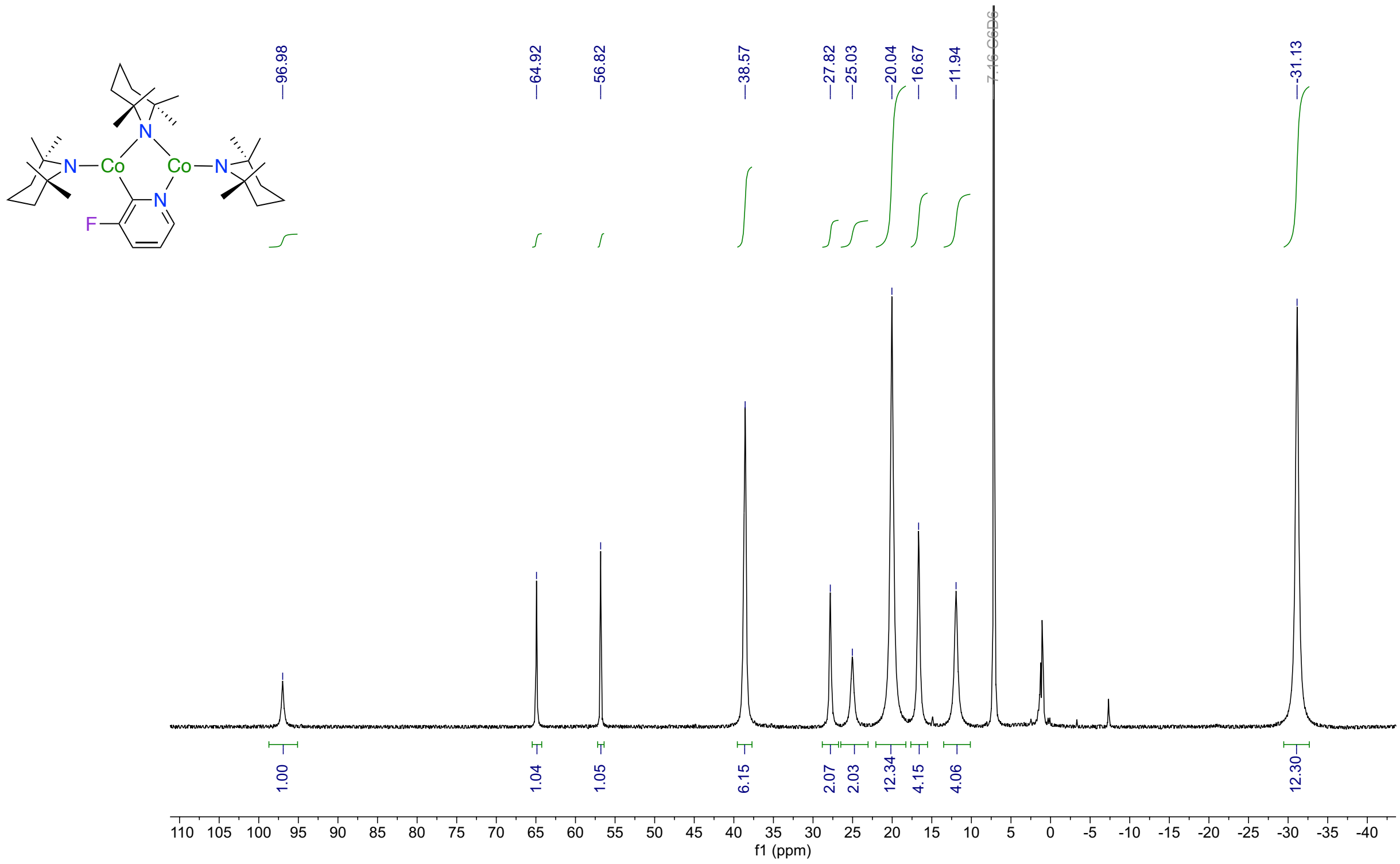

Figure S15. ^1H NMR spectrum of **3k** in C_6D_6.

**δ (^{1}H) / ppm**	**Assignment**
96.98	Pyridyl C*H* (1H)
64.92	Pyridyl C*H* (1H)
56.82	Pyridyl C*H* (1H)
38.57	TMP CH_2 (6H)
27.82	TMP CH_2 (2H)
25.03	TMP CH_2 (2H)
20.04	TMP *Me* (12H)
16.67	TMP CH_2 (4H)
11.94	TMP CH_2 (4H)
–31.13	TMP *Me* (12H)

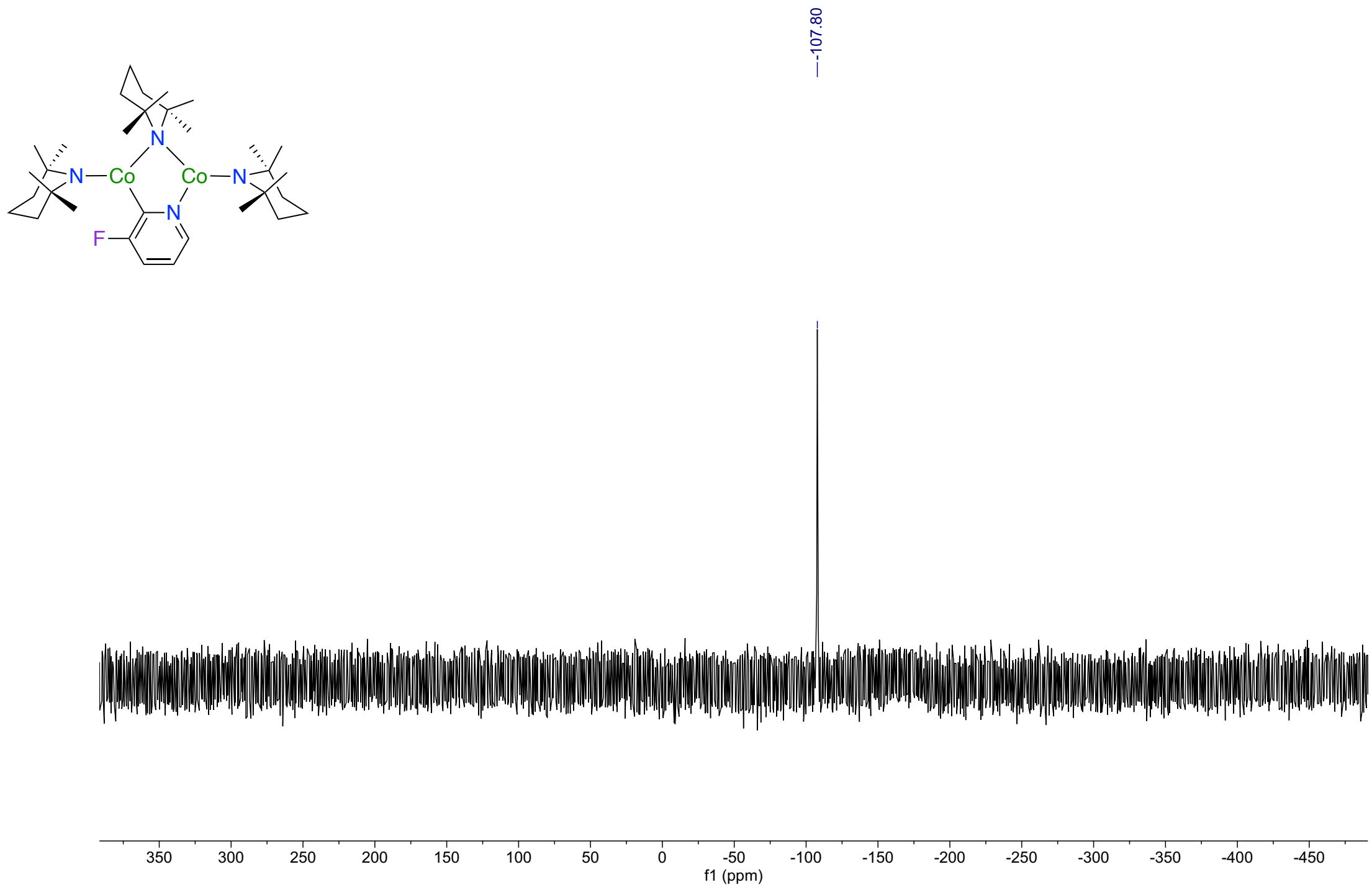

Figure S16. $^{19}F\{^{1}H\}$ NMR spectrum of **3k** in C_6D_6.

**δ (^{19}F) / ppm**	**Assignment**
–107.80	*Meta F*

[(8-Caffeinyl)$Co_2(TMP)_3$] (3l)

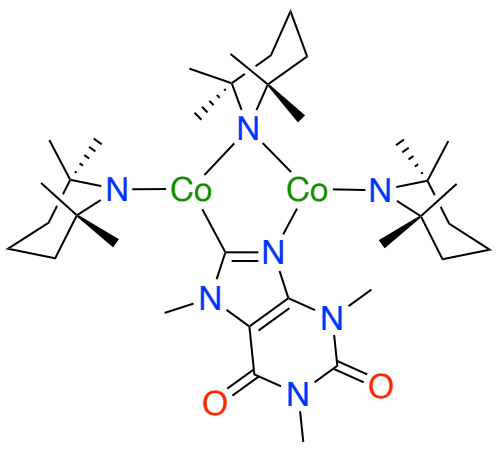

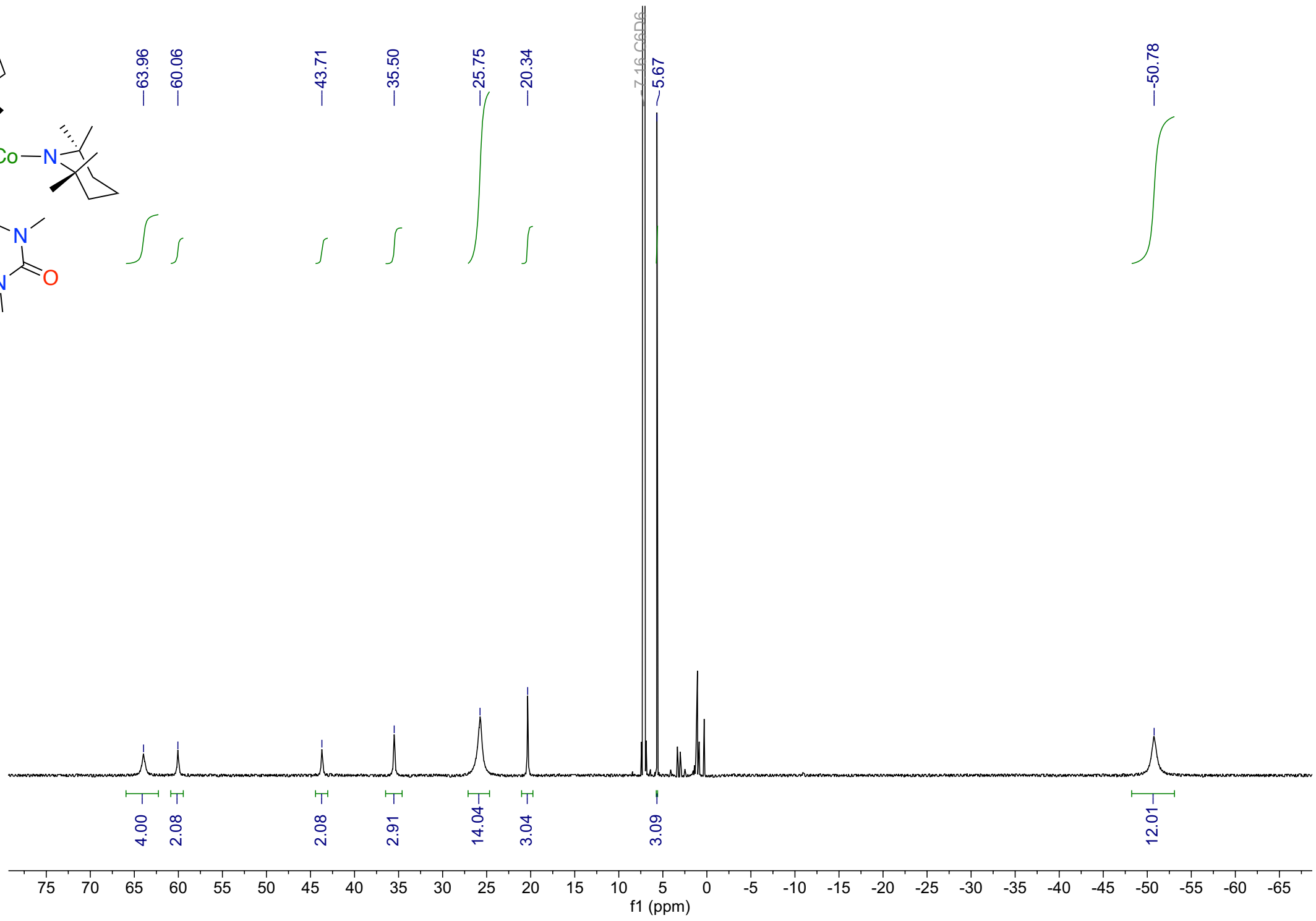

Figure S17. ^{1}H NMR spectrum of **3l** in C_6D_6.

δ (^{1}H) / ppm	Assignment
63.96	TMP C*H*$_2$ (4H)
60.06	TMP C*H*$_2$ (2H)
43.71	TMP C*H*$_2$ (2H)
35.50	NC*H*$_3$ (3H)
25.75	TMP C*H*$_2$ (2H)
25.75	TMP *Me* (12H)
20.34	NC*H*$_3$ (3H)
5.67	NC*H*$_3$ (3H)
–50.78	TMP *Me* (12H)

[{PhN=(Ph)C}Co_2(TMP)$_3$] (3m)

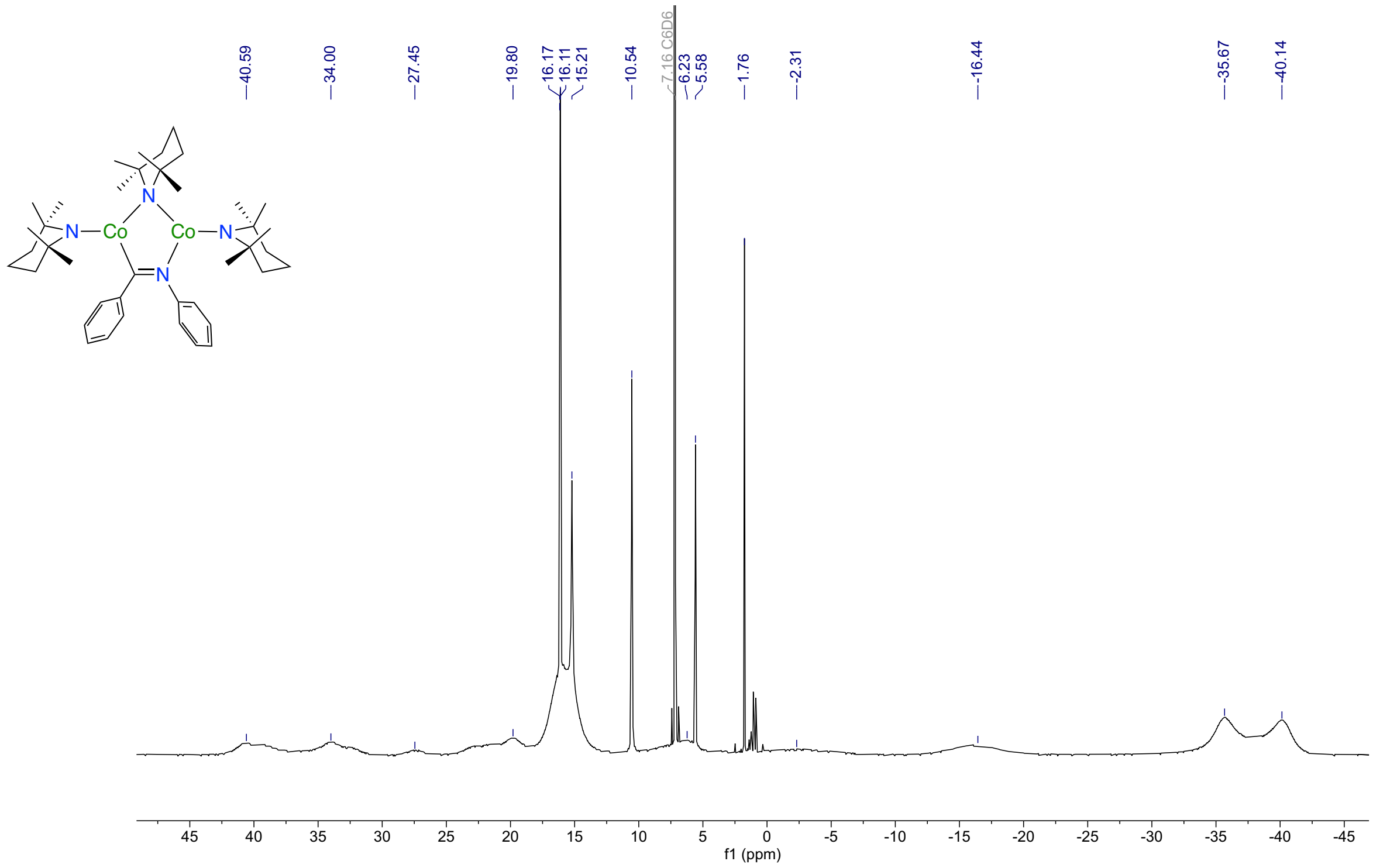

Figure S18. ^{1}H NMR spectrum of **3m** in C_6D_6.

δ (^{1}H) / ppm	Assignment
40.59	/
34.00	/
27.45	/
19.80	/
16.17	/
16.11	/
15.21	/
10.54	/
6.23	/
5.58	/
1.76	/
–2.31	/
–16.44	/
–35.67	/
–40.14	/

[(MeO)Co_2(TMP)$_3$] (4)

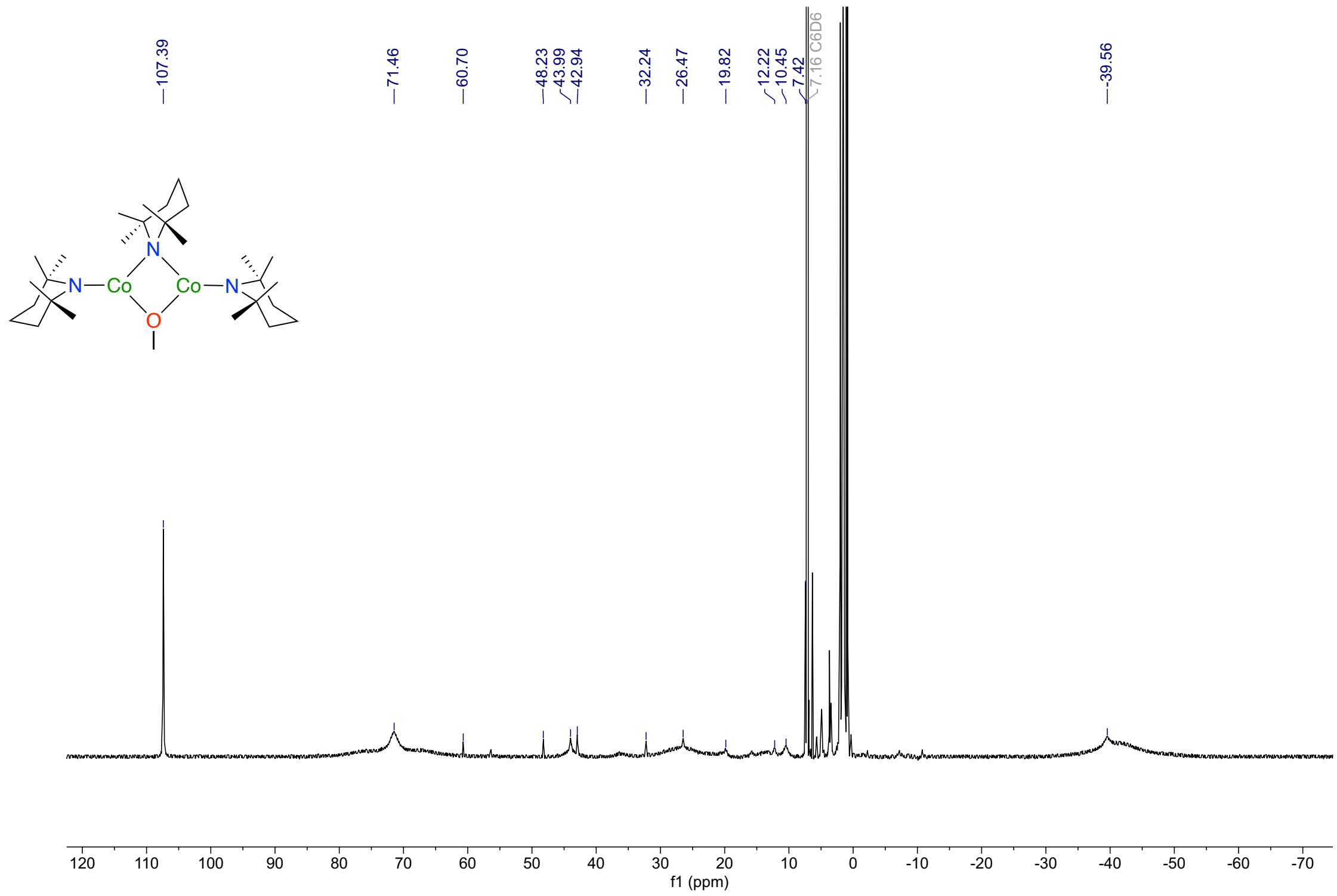

Figure S19. ^{1}H NMR spectrum of **4** in C_6D_6.

δ (^{1}H) / ppm	Assignment
107.39	/
71.46	/
60.70	/
48.23	/
43.99	/
42.94	/
32.24	/
26.47	/
19.82	/
12.22	/
10.45	/
7.42	/
–39.56	/

[{(Ph)(TMP)C=N}Co_2(TMP)$_3$] (5)

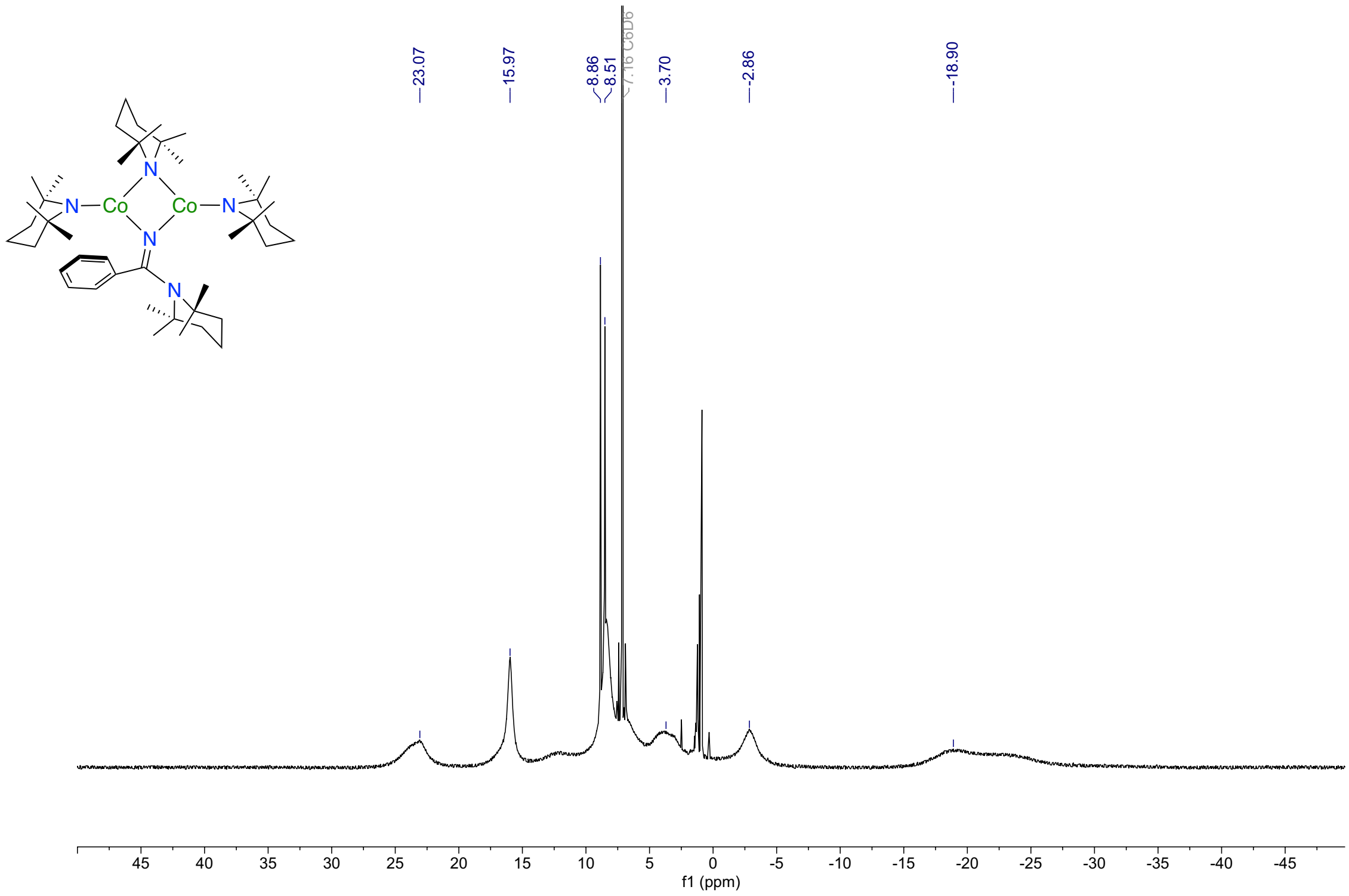

Figure S20. ^{1}H NMR spectrum of **5** in C_6D_6.

**δ (^{1}H) / ppm**	**Assignment**
23.07	/
15.97	/
8.86	/
8.51	/
3.70	/
–2.86	/
–18.90	/

[(2,5-$C_4H_2N_2$-pyz)Co_4(TMP)$_6$] (6a)

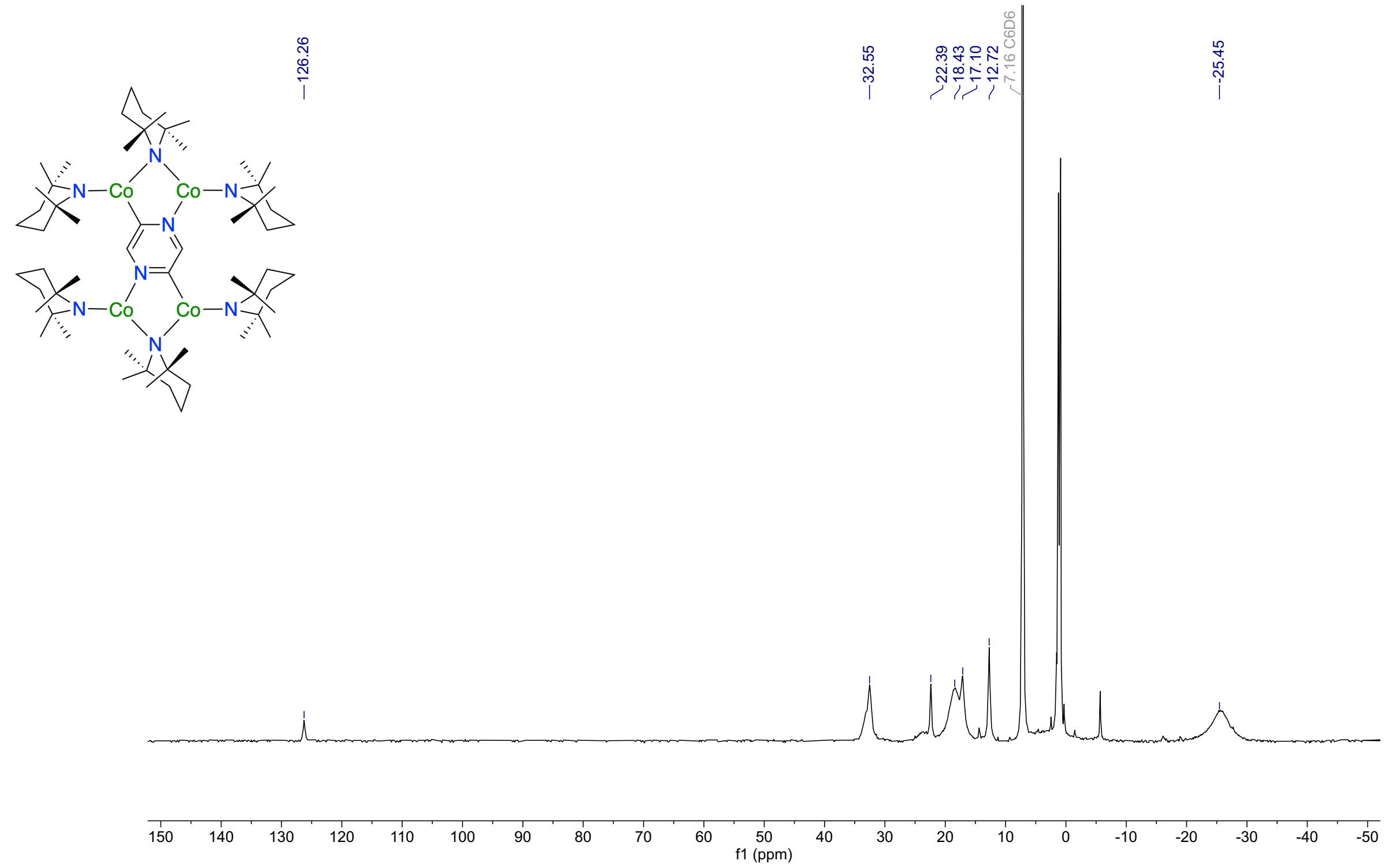

Figure S21. ^{1}H NMR spectrum of **6a** in C_6D_6.

δ (^{1}H) / ppm	Assignment
126.26	Pyrazinyl C*H* (2H)
32.55	/
22.39	/
18.43	/
17.10	/
12.72	/
–25.45	/

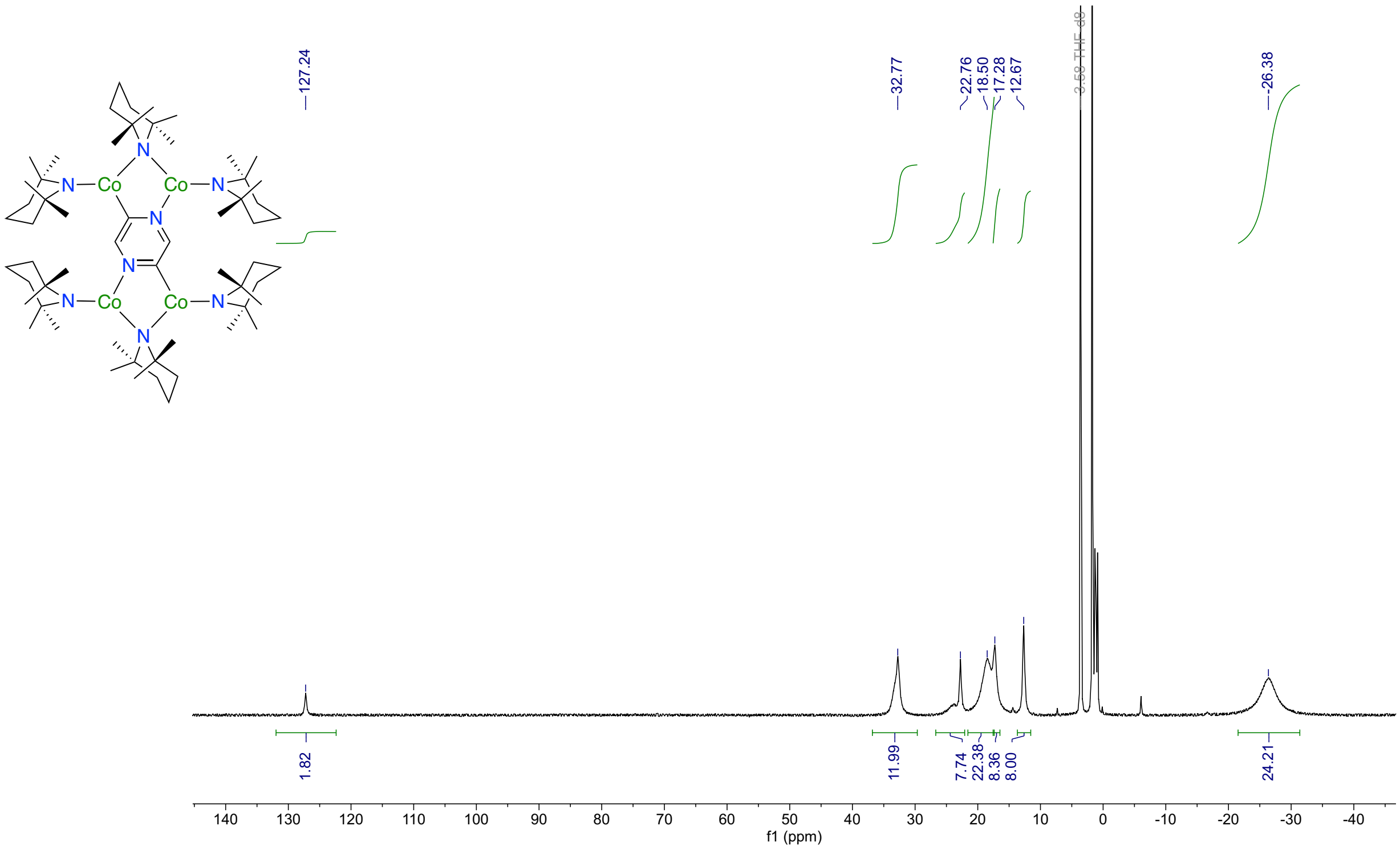

Figure S22. ^{1}H NMR spectrum of **6a** in d_8-THF.

**δ (^{1}H) / ppm**	**Assignment**
127.24	Pyrazinyl C*H* (2H)
32.77	TMP CH_2 (12H)
22.76	TMP CH_2 (8H)
18.50	TMP *Me* (24H)
17.28	TMP CH_2 (8H)
12.67	TMP CH_2 (8H)
–26.38	TMP *Me* (24H)

[(4,6-$C_4H_2N_2$-pym)Co_4(TMP)$_6$] (6b)

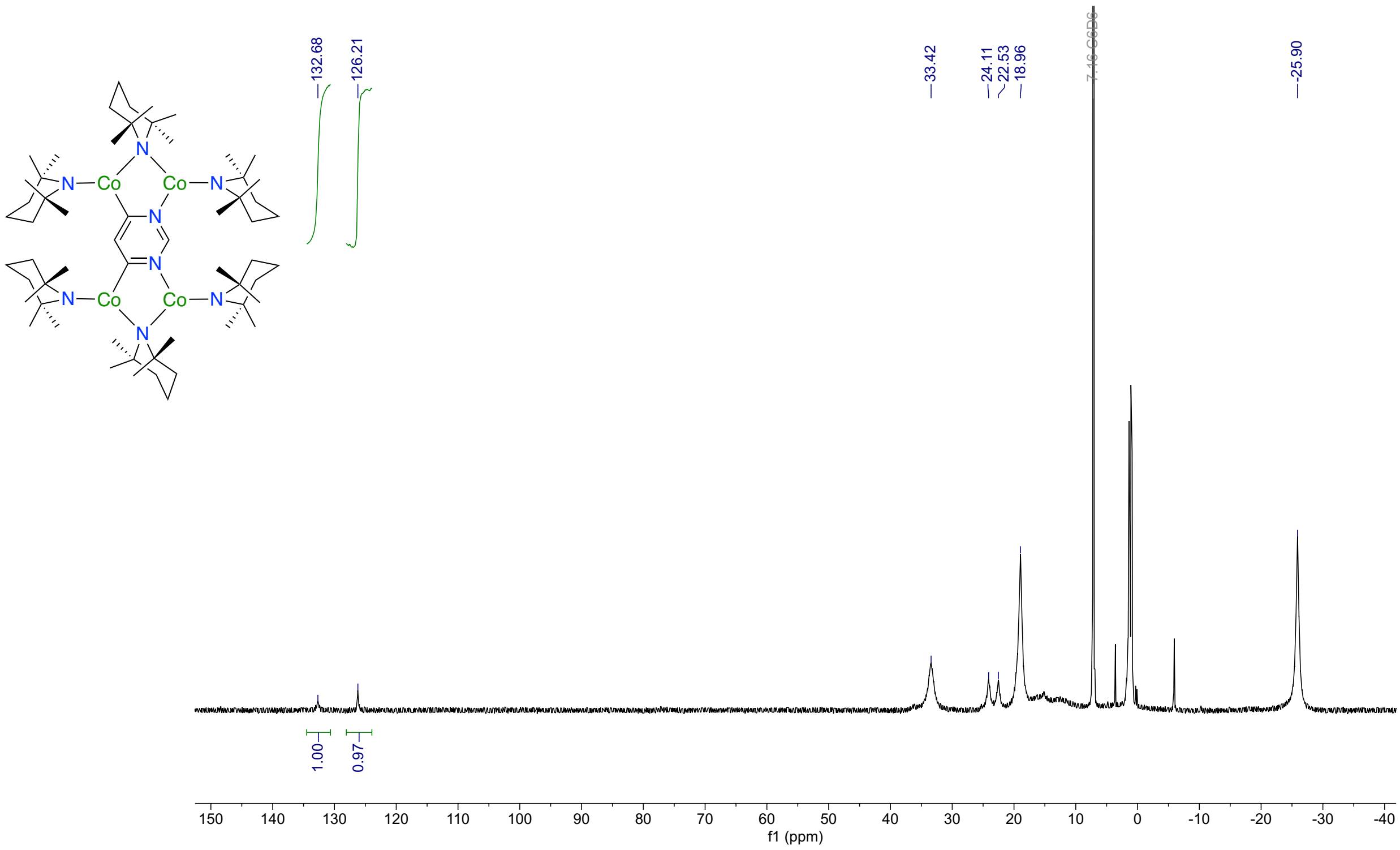

Figure S23. ^{1}H NMR spectrum of **6b** in C_6D_6.

**δ (^{1}H) / ppm**	**Assignment**
132.68	Pyrimidinyl C*H* (1H)
126.21	Pyrimidinyl C*H* (1H)
33.42	/
24.11	/
22.53	/
18.96	/
–25.90	/

[(4-$C_4H_3N_2$-pym)Co_2(TMP)$_3$] (7b)

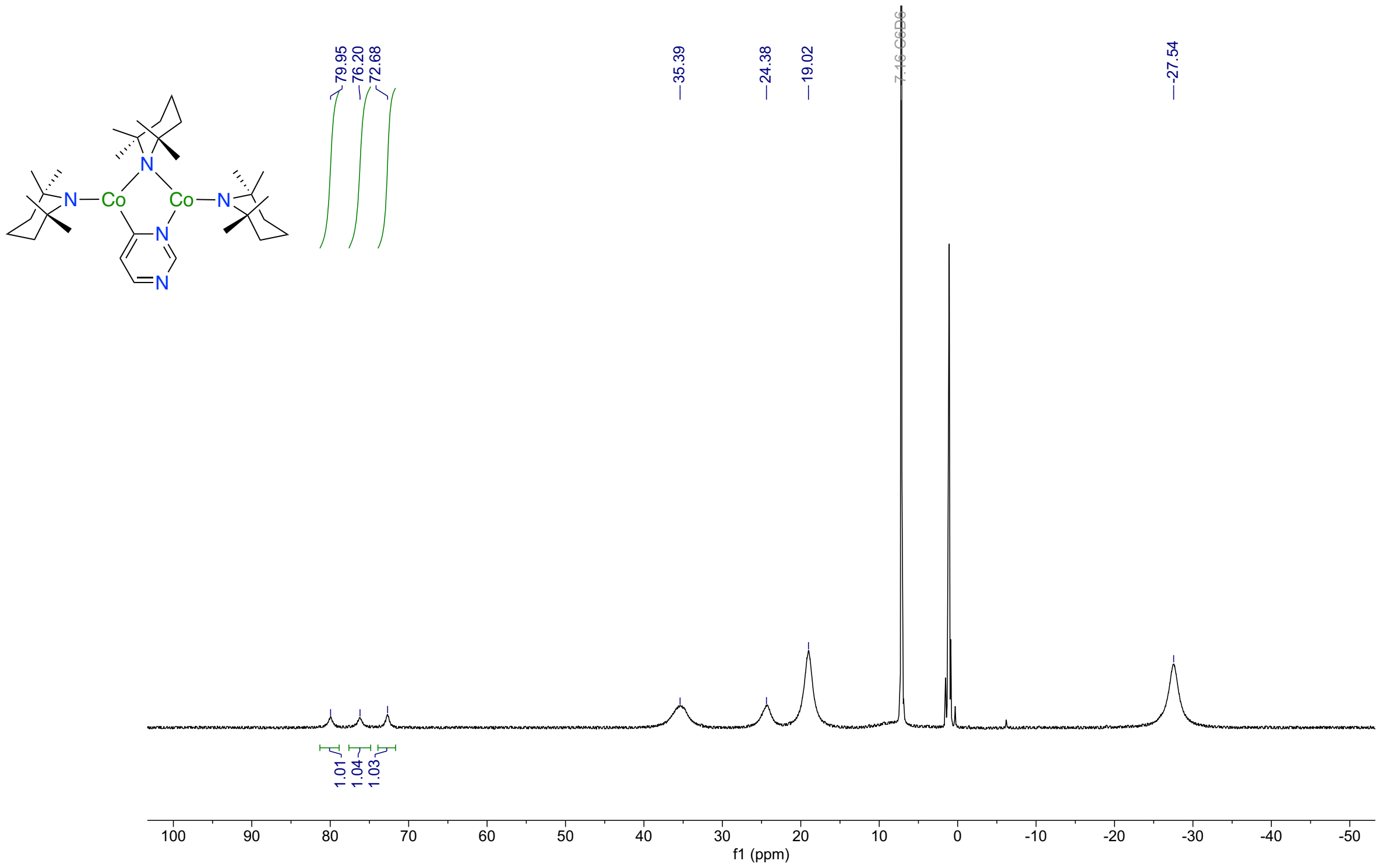

Figure S24. ^{1}H NMR spectrum of **7b** in C_6D_6.

**δ (^{1}H) / ppm**	**Assignment**
79.95	Pyrimidinyl C*H* (1H)
76.20	Pyrimidinyl C*H* (1H)
72.68	Pyrimidinyl C*H* (1H)
35.39	/
24.38	/
19.02	/
–27.54	/

[(3-$C_4H_3N_2$-pdz)Co_2(TMP)$_3$] (7c)

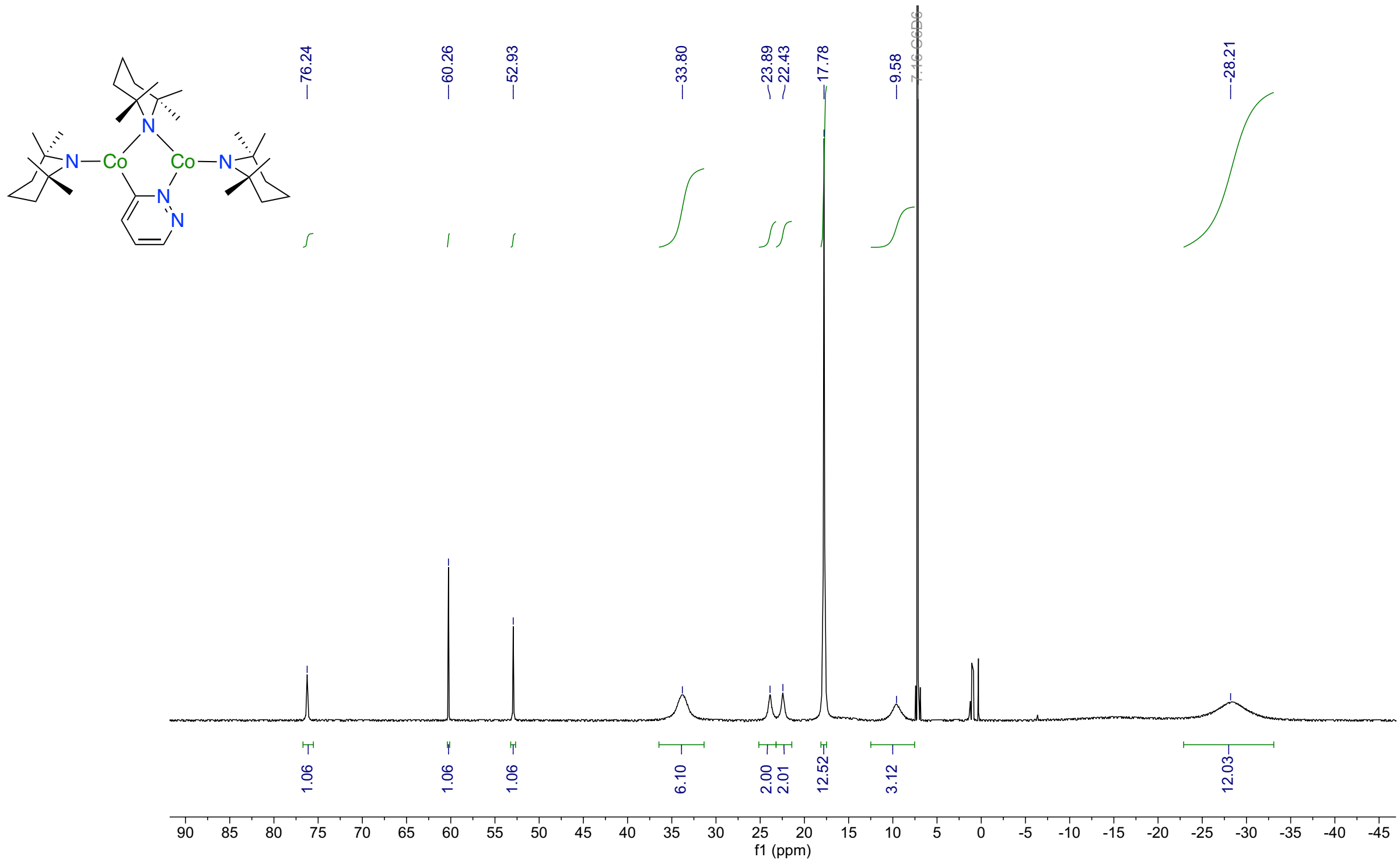

Figure S25. ^{1}H NMR spectrum of **7c** in C_6D_6.

**δ (^{1}H) / ppm**	**Assignment**
76.24	Pyridazinyl C*H* (1H)
60.26	Pyridazinyl C*H* (1H)
52.93	Pyridazinyl C*H* (1H)
33.80	TMP CH_2 (6H)
23.89	TMP CH_2 (2H)
22.43	TMP CH_2 (2H)
17.78	TMP *Me* (12H)
9.58	TMP CH_2 (4H)
–28.21	TMP *Me* (12H)

[(2-Quinoxalinyl)Co_2(TMP)$_3$] (7d)

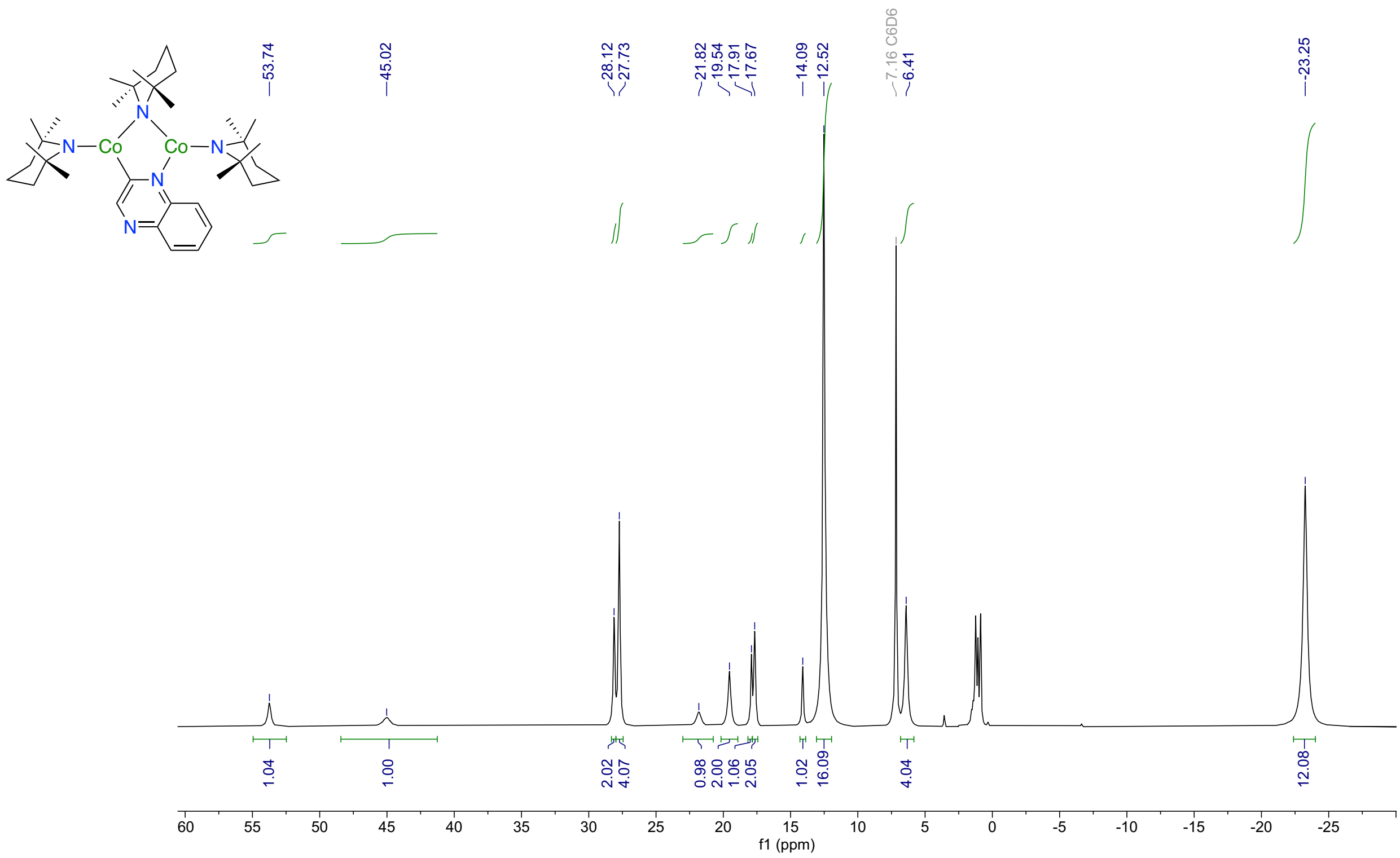

Figure S26. ^{1}H NMR spectrum of **7d** in C_6D_6.

δ (^{1}H) / ppm	Assignment
53.74	Quinoxalinyl C*H* (1H)
45.02	Quinoxalinyl C*H* (1H)
28.12	TMP C*H*$_2$ (2H)
27.73	TMP C*H*$_2$ (4H)
21.82	Quinoxalinyl C*H* (1H)
19.54	TMP C*H*$_2$ (2H)
17.91	Quinoxalinyl C*H* (1H)
17.67	TMP C*H*$_2$ (2H)
14.09	Quinoxalinyl C*H* (1H)
12.52	TMP *Me* (12H)
12.52	TMP C*H*$_2$ (4H)
6.41	TMP C*H*$_2$ (4H)
–23.25	TMP *Me* (12H)

[(4-Quinazolinyl)Co_2(TMP)$_3$] (7e)

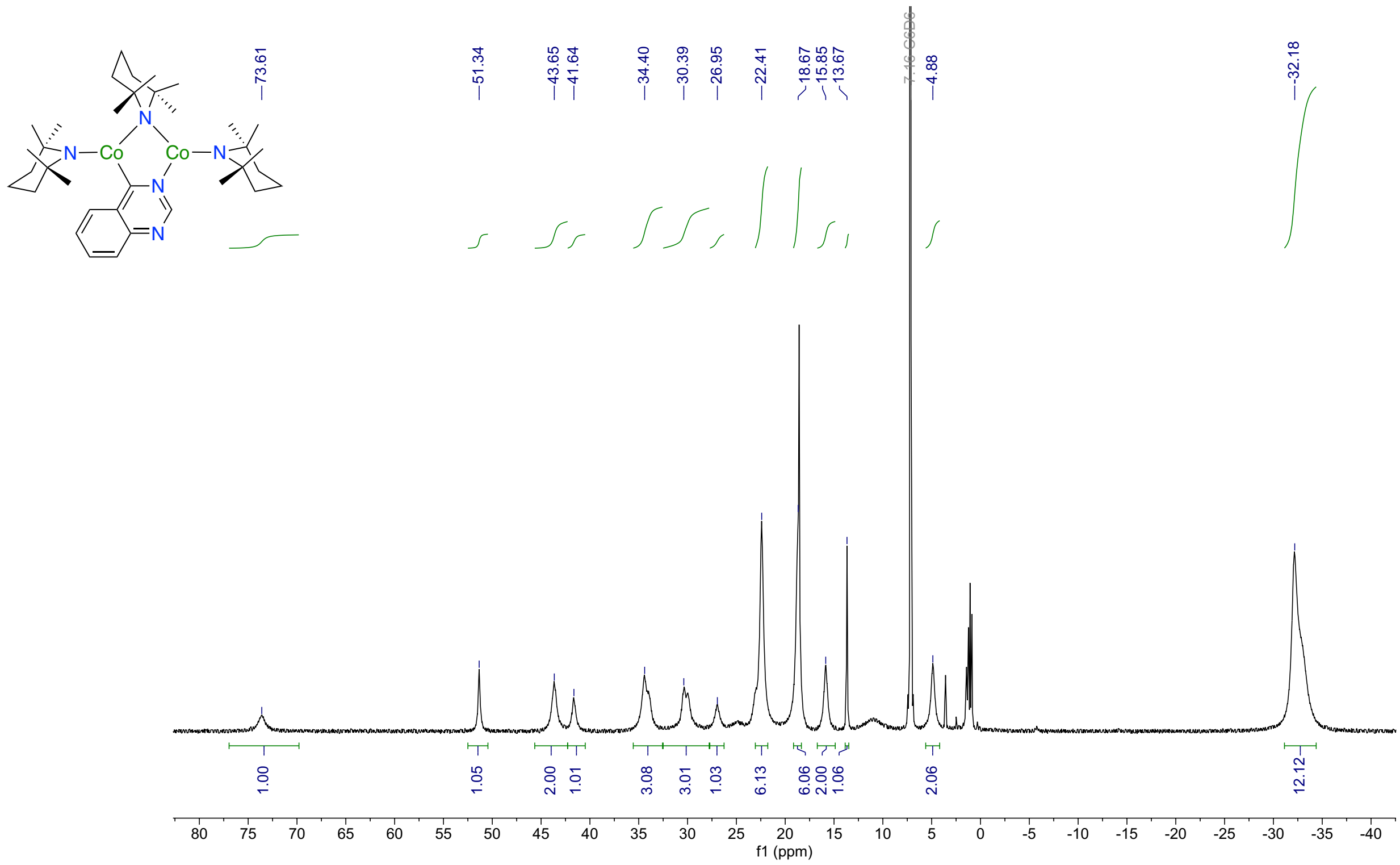

Figure S27. ^1H NMR spectrum of **7e** in C_6D_6.

δ (^1H) / ppm	Assignment
73.61	Quinazolinyl C*H* (1H)
51.34	Quinazolinyl C*H* (1H)
43.65	TMP CH_2 (2H)
41.64	Quinazolinyl C*H* (1H)
34.40	TMP CH_2 (3H)
30.39	TMP CH_2 (3H)
26.95	Quinazolinyl C*H* (1H)
22.41	TMP *Me* (6H)
18.67	TMP *Me* (6H)
15.85	TMP CH_2 (2H)
13.67	Quinazolinyl C*H* (1H)
4.88	TMP CH_2 (2H)
–32.18	TMP *Me* (12H)

2-(2,2,6,6-Tetramethylpiperidin-1-yl)pyridine (8a)

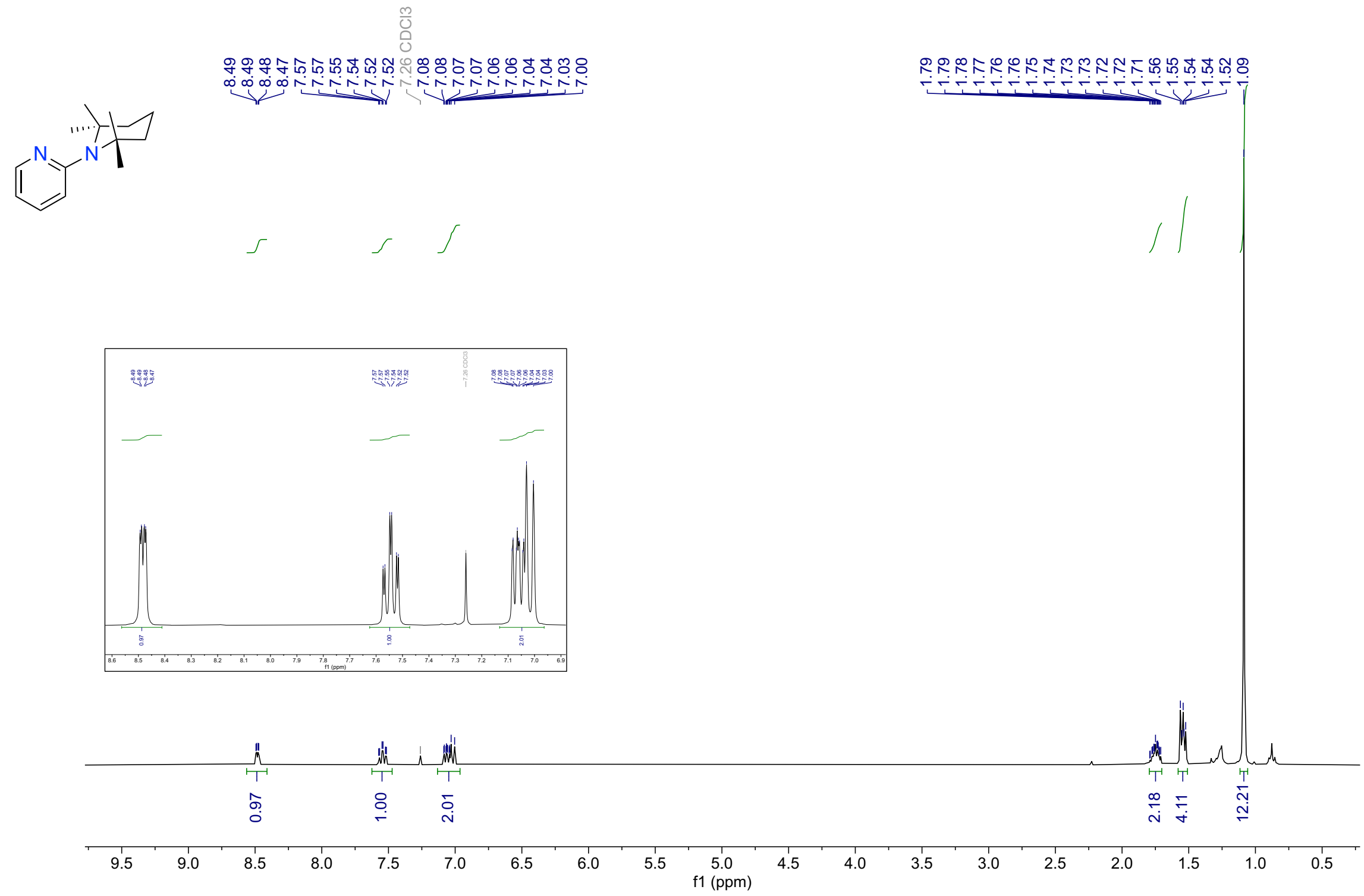

Figure S28. ^{1}H NMR spectrum of **8a** in $CDCl_3$.

δ (^{1}H) / ppm	Assignment
8.48	Pyridinyl C*H* (1H)
7.54	Pyridinyl C*H* (1H)
7.09 – 7.04	Pyridinyl C*H* (1H)
7.02	Pyridinyl C*H* (1H)
1.82 – 1.68	TMP CH_2 (2H)
1.59 – 1.51	TMP CH_2 (4H)
1.09	TMP *Me* (12H)

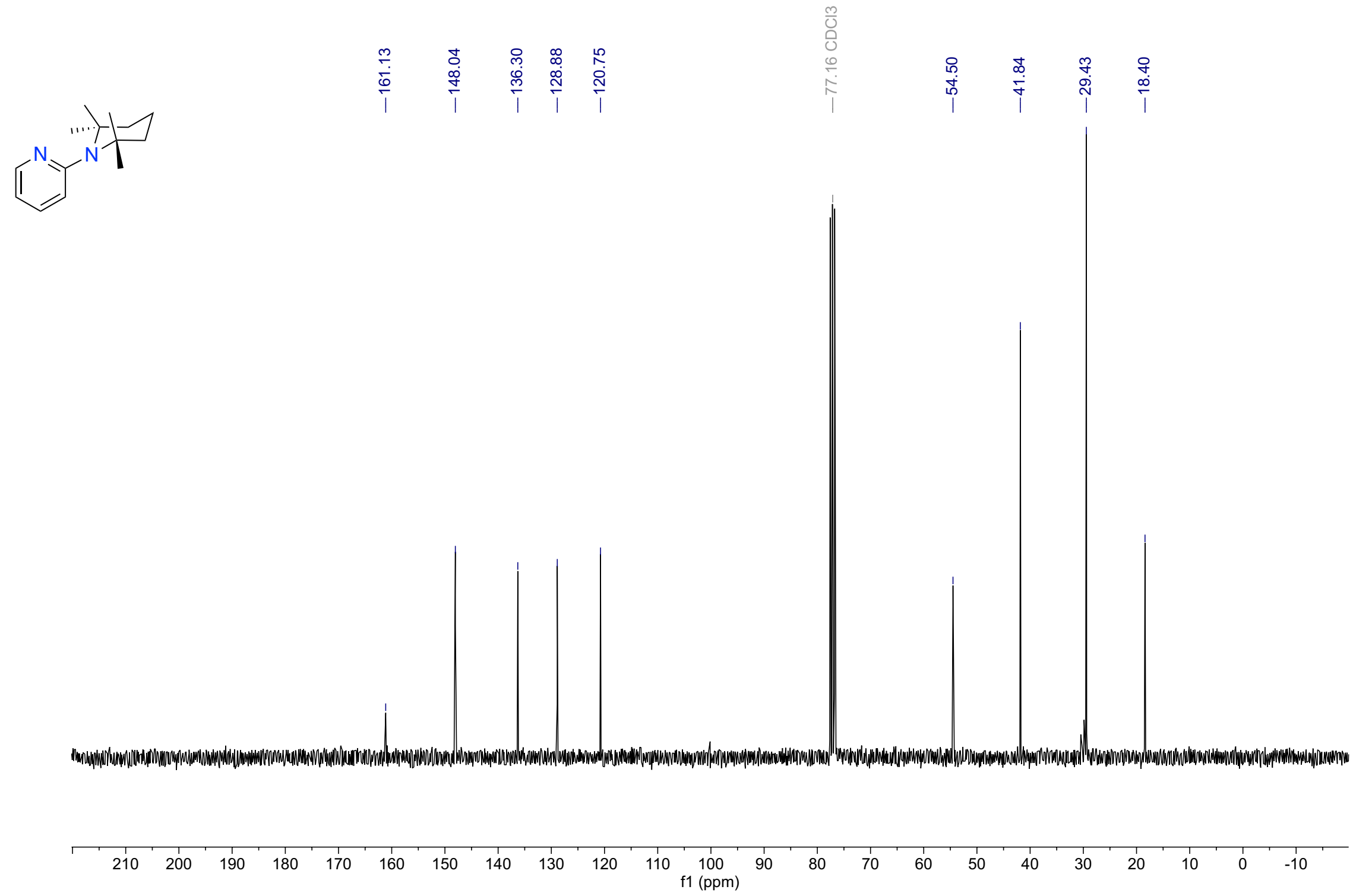

Figure S29. ^{13}C NMR spectrum of **8a** in $CDCl_3$.

**δ (^{1}H) / ppm**	**Assignment**
161.13	Pyridinyl *C*
148.04	Pyridinyl *C*
136.30	Pyridinyl *C*
128.88	Pyridinyl *C*
120.75	Pyridinyl *C*
54.50	TMP *C*
41.84	TMP *C*
29.43	TMP *C*
18.40	TMP *C*

2-Phenyl-6-(2,2,6,6-tetramethylpiperidin-1-yl)pyridine (8b)

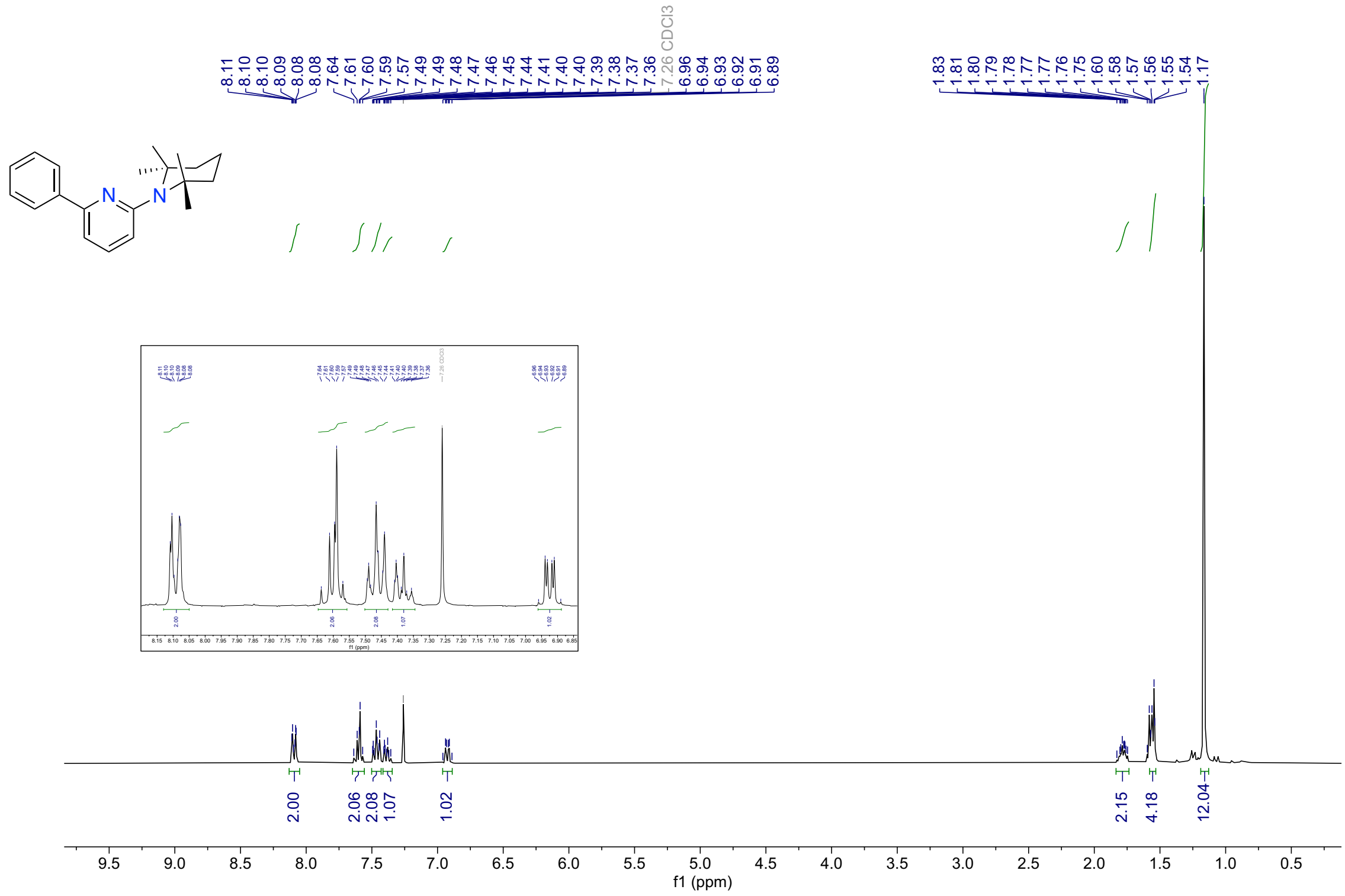

Figure S30. ^{1}H NMR spectrum of **8b** in $CDCl_3$.

**δ (^{1}H) / ppm**	**Assignment**
8.14 – 8.04	Aromatic C*H* (2H)
7.67 – 7.53	Aromatic C*H* (2H)
7.51 – 7.43	Aromatic C*H* (2H)
7.42 – 7.33	Aromatic C*H* (1H)
6.92	Aromatic C*H* (1H)
1.86 – 1.72	TMP CH_2 (2H)
1.63 – 1.52	TMP CH_2 (4H)
1.17	TMP *Me* (12H)

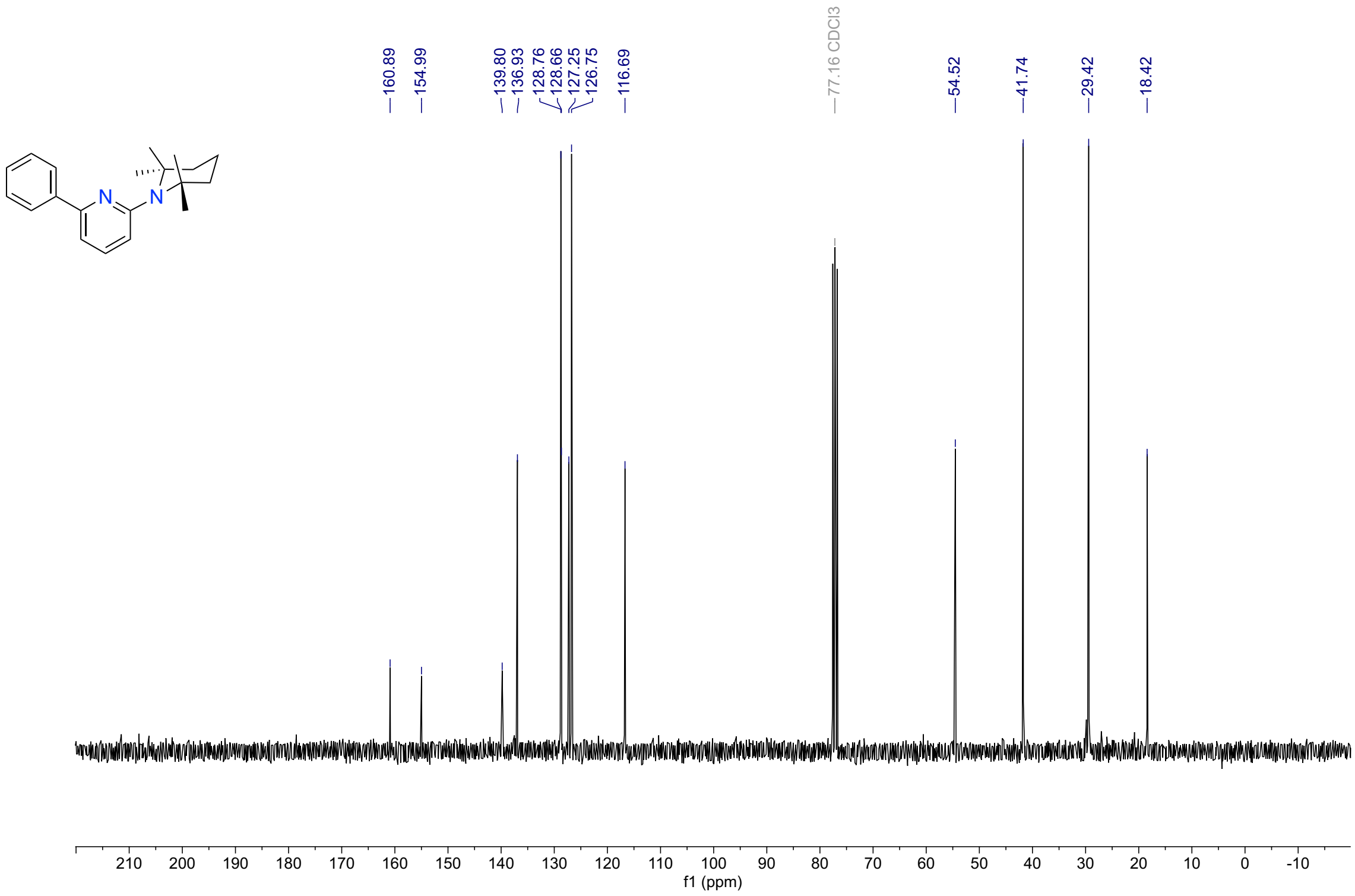

Figure S31. ^{13}C NMR spectrum of **8b** in $CDCl_3$.

**δ (^{1}H) / ppm**	**Assignment**
160.89	Aromatic *C*
154.99	Aromatic *C*
139.80	Aromatic *C*
136.93	Aromatic *C*
128.76	Aromatic *C*
128.66	Aromatic *C*
127.25	Aromatic *C*
126.75	Aromatic *C*
116.69	Aromatic *C*
54.52	TMP *C*
41.74	TMP *C*
29.42	TMP *C*
18.42	TMP *C*

2,5-Bis(2,2,6,6-tetramethylpiperidin-1-yl)pyrazine (8c)

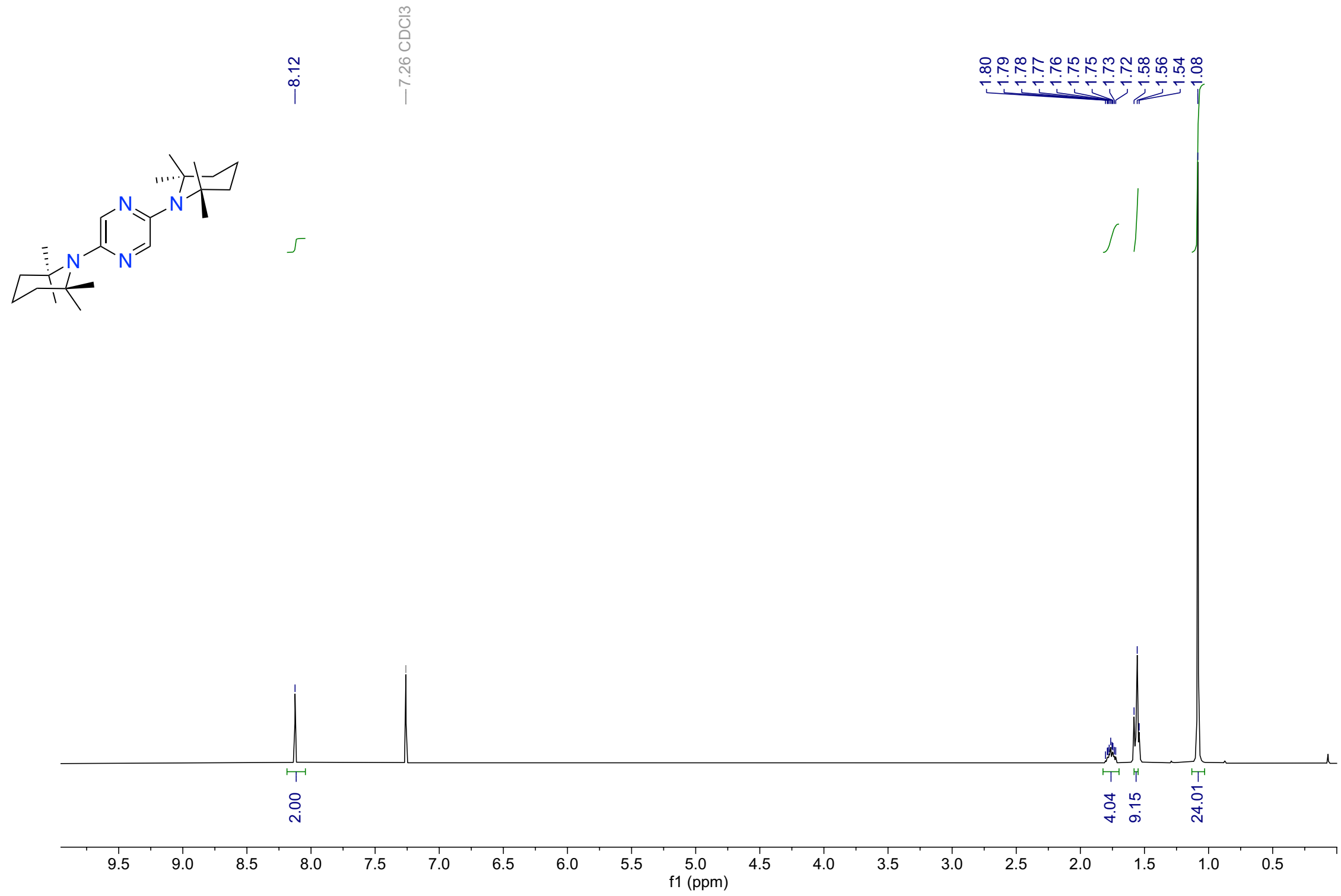

Figure S32. ^1H NMR spectrum of **8c** in $CDCl_3$.

δ (^1H) / ppm	Assignment
8.12	Pyrazinyl C*H* (2H)
1.83 – 1.69	TMP CH_2 (4H)
1.61 – 1.51	TMP CH_2 (8H)
1.08	TMP *Me* (24H)

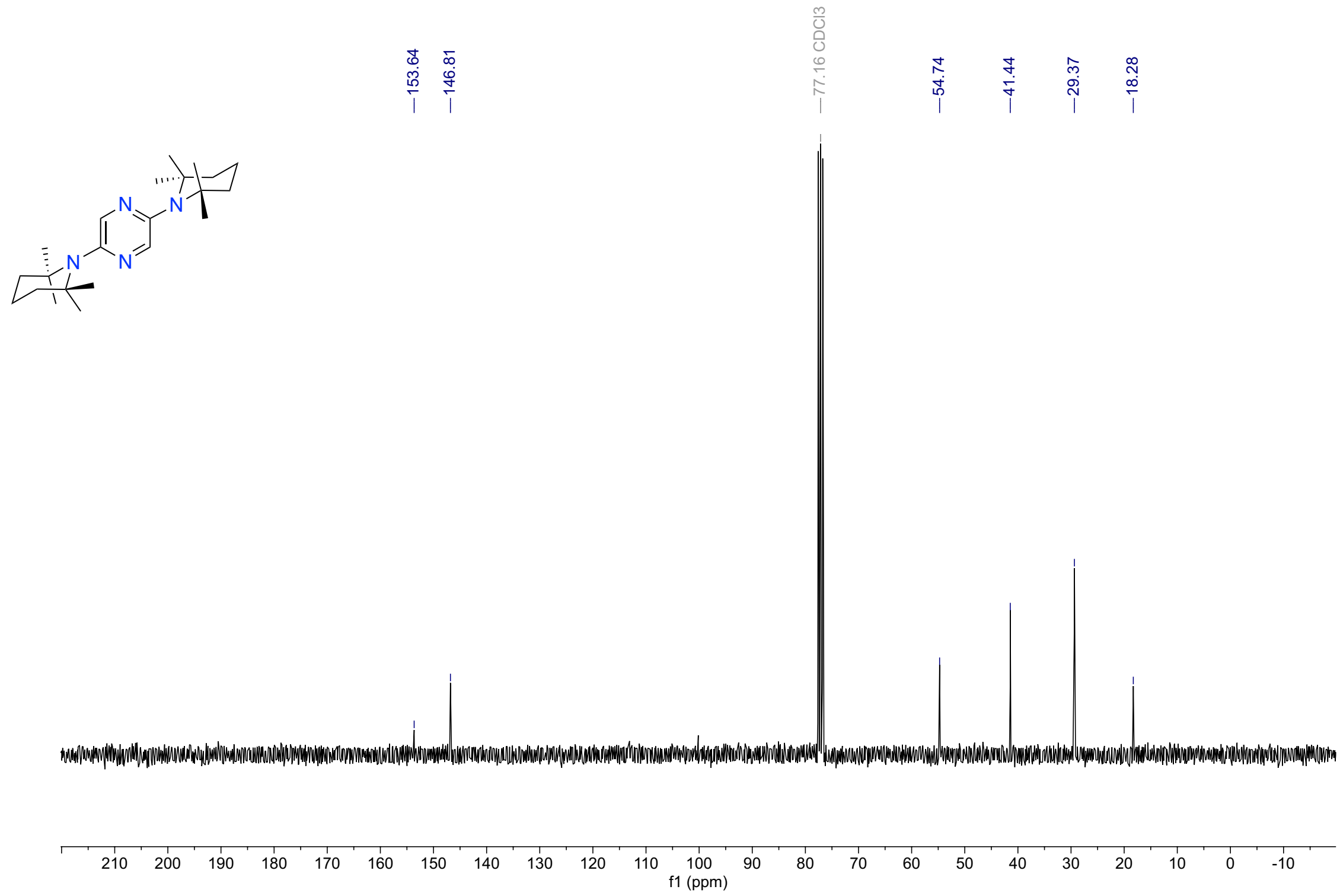

Figure S33. ^{13}C NMR spectrum of **8c** in $CDCl_3$.

**δ (^{1}H) / ppm**	**Assignment**
153.64	Pyrazinyl *C*
146.81	Pyrazinyl *C*
54.74	TMP *C*
41.44	TMP *C*
29.37	TMP *C*
18.28	TMP *C*

4-(2,2,6,6-Tetramethylpiperidin-1-yl)pyrimidine (8d)

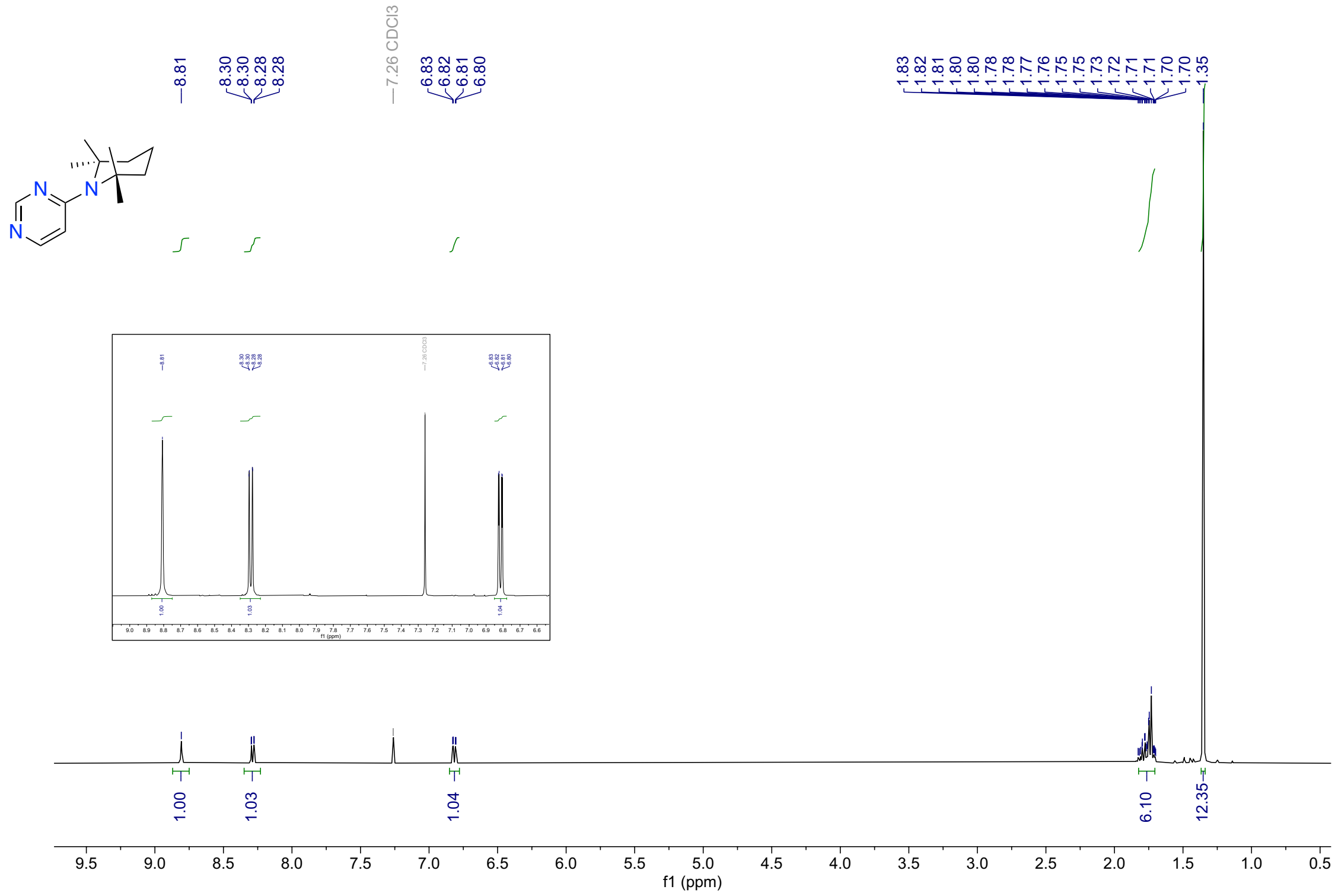

Figure S34. ^{1}H NMR spectrum of **8d** in $CDCl_3$.

**δ (^{1}H) / ppm**	**Assignment**
8.81	Pyrimidinyl C*H* (1H)
8.29	Pyrimidinyl C*H* (1H)
6.82	Pyrimidinyl C*H* (1H)
1.88 – 1.66	TMP CH_2 (6H)
1.35	TMP *Me* (12H)

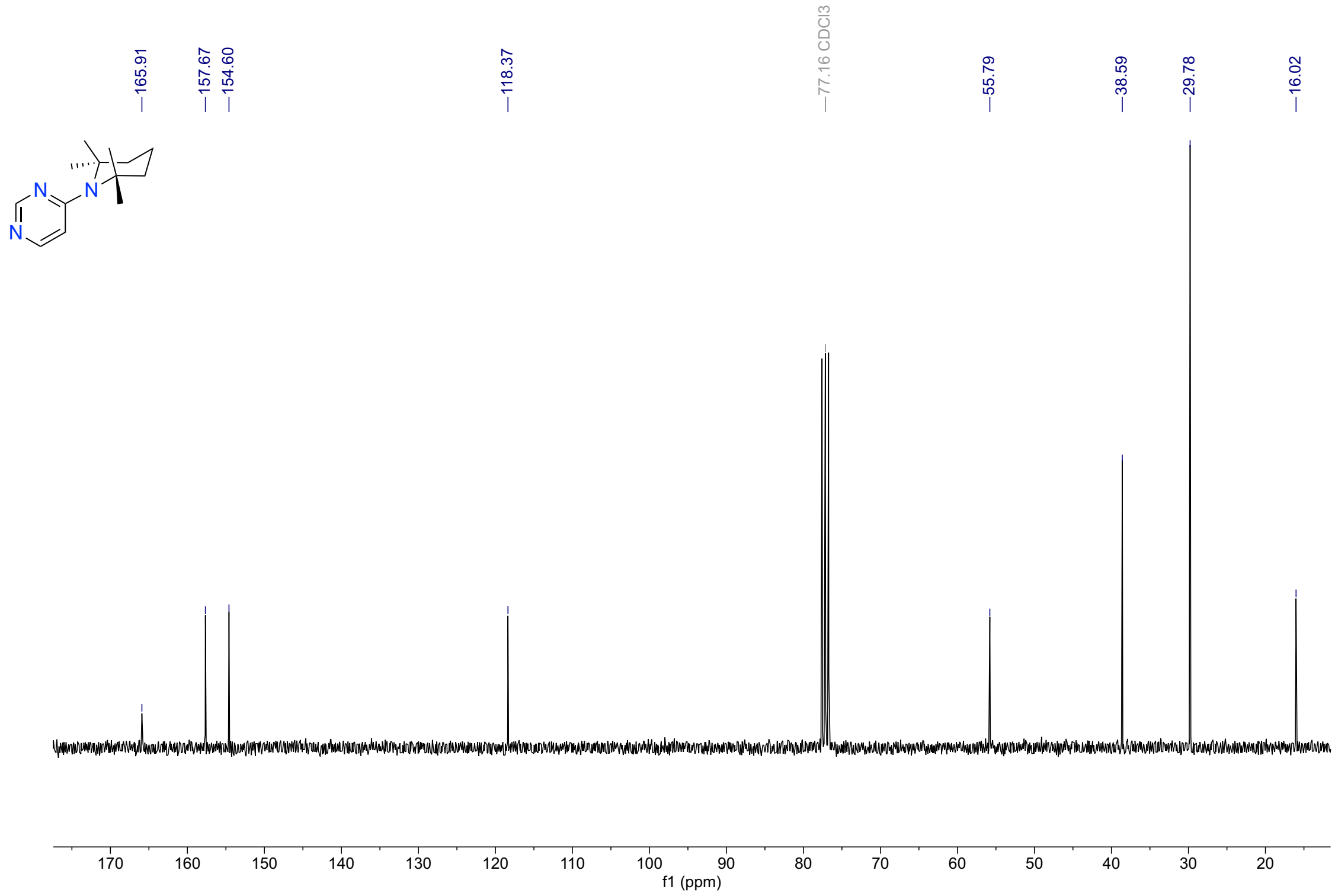

Figure S35. $^{13}C\{^{1}H\}$ NMR spectrum of **8d** in $CDCl_3$.

**δ (^{13}C) / ppm**	**Assignment**
165.91	Pyrimidinyl *C*
157.67	Pyrimidinyl *C*
154.60	Pyrimidinyl *C*
118.37	Pyrimidinyl *C*
55.79	TMP *C*
38.59	TMP *C*
29.78	TMP *C*
16.02	TMP *C*

NMR Studies and Supplementary Experiments

Co(TMP)$_2$ (1^{Co}) + pyridine (1.0 eq) in C_6D_6

Co(TMP)$_2$ (**1**^{Co}) (10.0 mg, 29.5 µmol) was dissolved in C_6D_6 (0.5 mL) in a J. Young NMR tube to which pyridine (2.4 µL, 29.5 µmol) was subsequently added. A ^{1}H NMR spectrum was recorded to show formation of the pyridine adduct **2a**. Subsequently the solution was heated at 70 °C for 4 hours. *In situ* NMR monitoring was carried out where a ^{1}H NMR spectrum was recorded every hour. Full conversion of **2a** to the cobaltated species **3a** along with TMP(H) generation was observed. Resonances of the excess pyridine overlapped with the broad signal at 10.13 ppm and the solvent signal at 7.16 ppm.

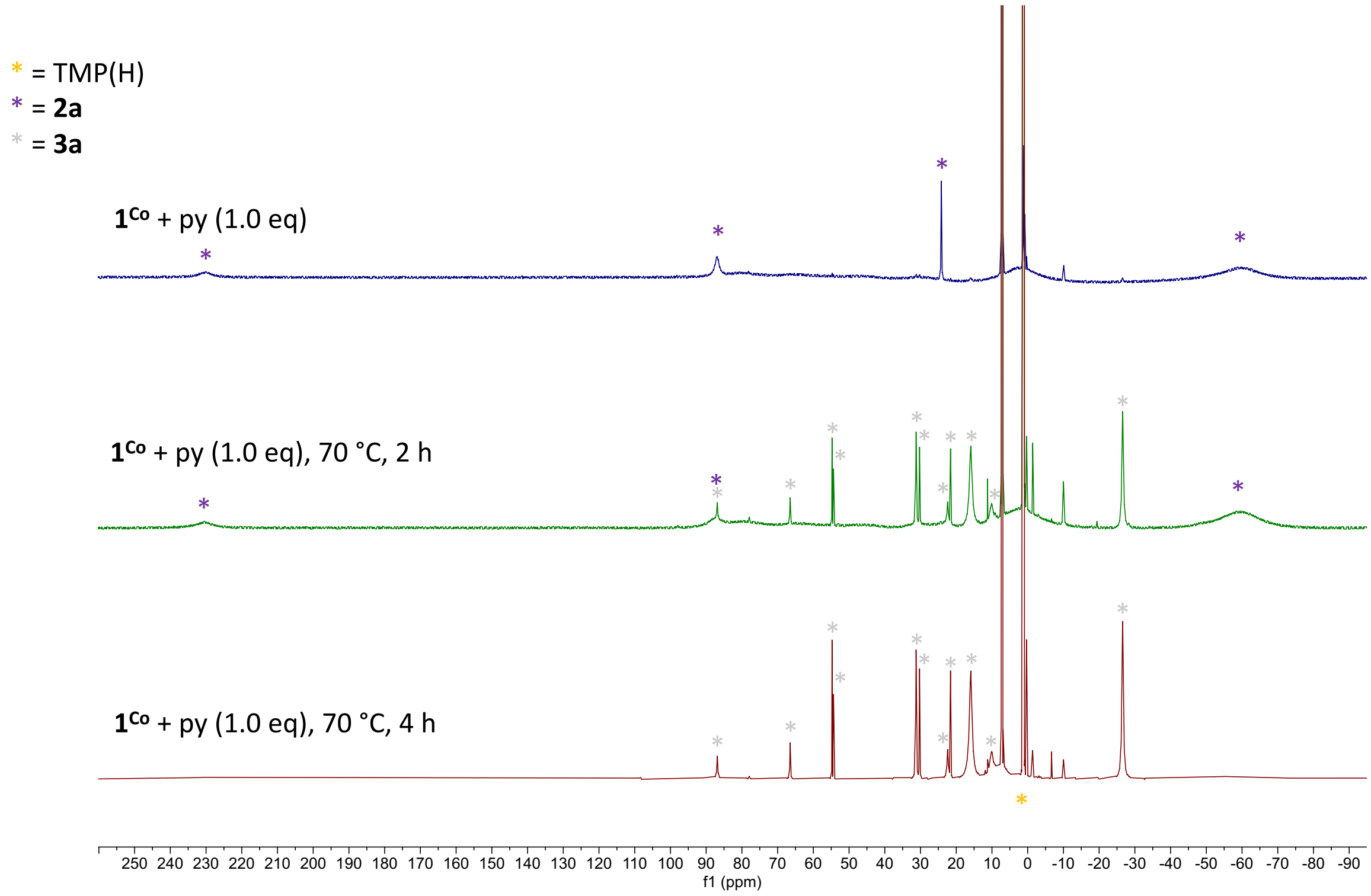

Figure S36. ^{1}H NMR spectra of the reaction of **1**^{Co} and pyridine (1.0 eq) in C_6D_6 in the 250 to –90 ppm region at each stage of the *in situ* reaction.

Co(TMP)$_2$ (1^{Co}) + pyridine (0.5 eq) in C_6D_6

Co(TMP)$_2$ (**1**^{Co}) (15.0 mg, 44.2 µmol) was dissolved in C_6D_6 (0.5 mL) in a J. Young NMR tube to which pyridine (1.8 µL, 22.1 µmol) was subsequently added. After heating at 70 °C for 4 hours, a ^{1}H NMR spectrum was recorded where full conversion into the cobaltated species **3a** along with TMP(H) formation was evident when compared to the ^{1}H NMR spectrum of isolated crystals of **3a**.

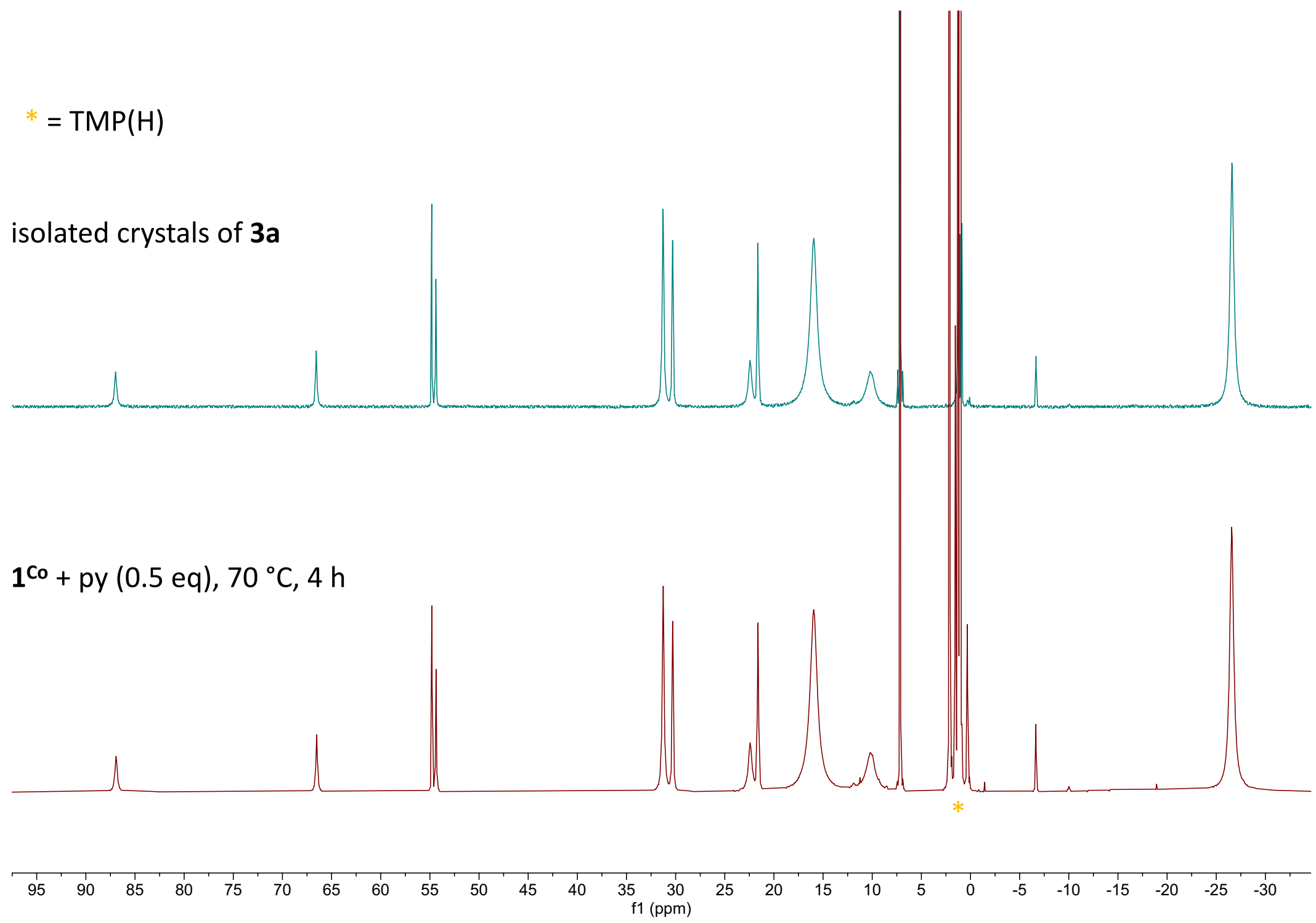

Figure S37. ^{1}H NMR spectra of isolated crystals of **3a** and of the reaction of **1**^{Co} and pyridine (0.5 eq) after heating at 70 °C for 4 h in C_6D_6 in the 95 to –30 ppm region.

Co(TMP)$_2$ (1^{Co}) + IMes (1.0 eq) + pyridine (1.0 eq) in C_6D_6

Co(TMP)$_2$ (**1^{Co}**) (15.0 mg, 44.2 µmol) and IMes (13.5 mg, 44.2 µmol) were dissolved in C_6D_6 (0.5 mL) in a J. Young NMR tube to which pyridine (3.6 µL, 44.2 µmol) was subsequently added. A ^{1}H NMR spectrum was recorded to show formation of the IMes carbene adduct **1^{Co}-IMes**. After heating at 70 °C for 16 hours, another ^{1}H NMR spectrum was recorded where resonances of unreacted pyridine and newly generated TMP(H) due to decomposition of **1^{Co}** were visible. No formation of the cobaltated species **3a** was observed, corroborating the diminished reactivity of **1^{Co}-IMes** towards Co–H exchange with pyridine.

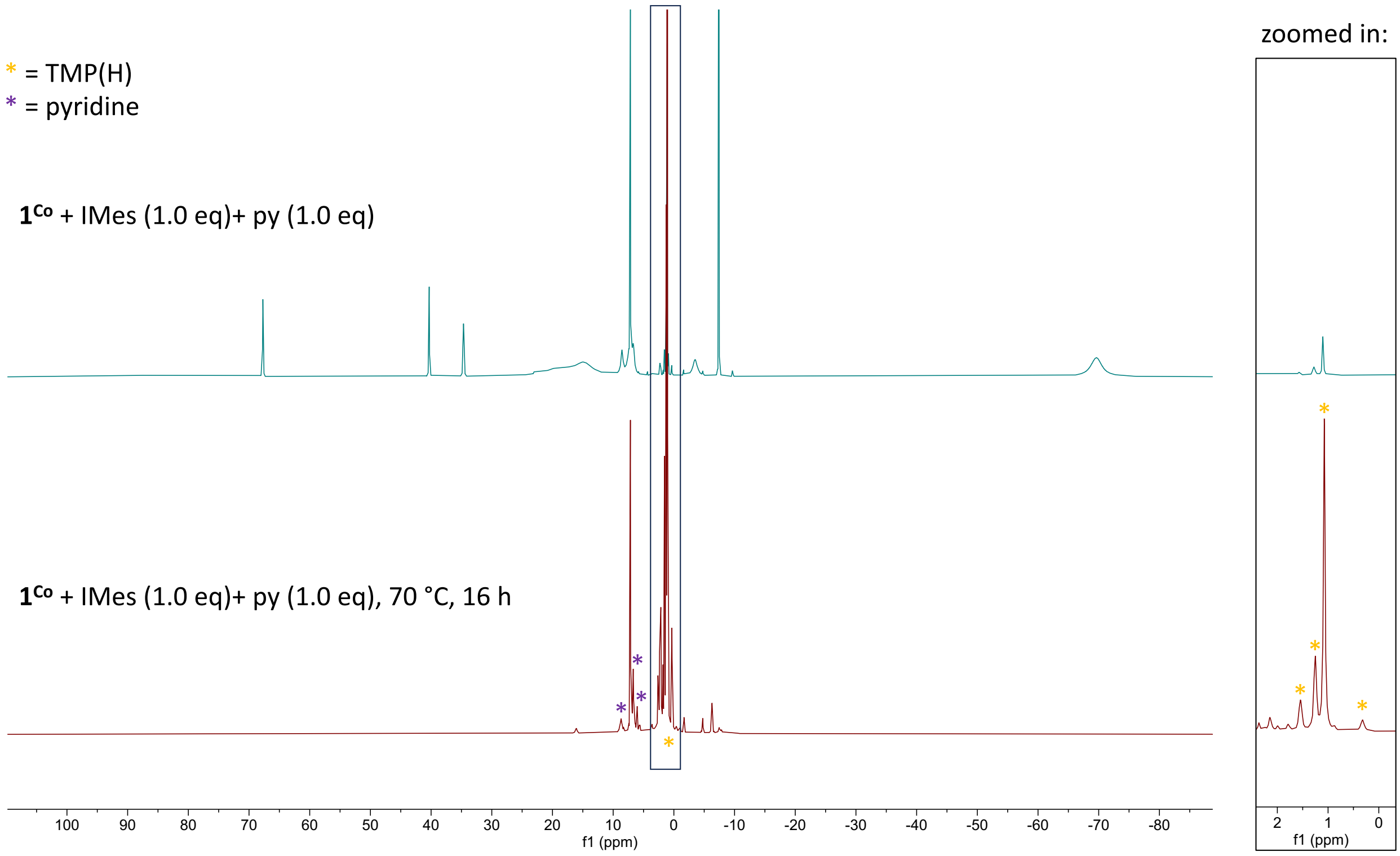

Figure S38. ^{1}H NMR spectra of the reaction of **1^{Co}**, IMes (1.0 eq) and pyridine (1.0 eq) in C_6D_6 in the 100 to –80 ppm region at each stage of the *in situ* reaction.

$Fe(TMP)_2$ (1^{Fe}) + pyridine (1.0 eq) in C_6D_6

$Fe(TMP)_2$ (**1**^{Fe}) (10.0 mg, 29.7 μmol) was dissolved in C_6D_6 (0.5 mL) in a J. Young NMR tube to which pyridine (2.4 μL, 29.7 μmol) was subsequently added. A ^{1}H NMR spectrum was recorded to show formation of the pyridine adduct **2**^{Fe}. After heating at 70 °C for 16 hours, another ^{1}H NMR spectrum was recorded where decomposition of **2**^{Fe} into **1**^{Fe} and mostly TMP(H) was visible without any hint of ferration of pyridine.

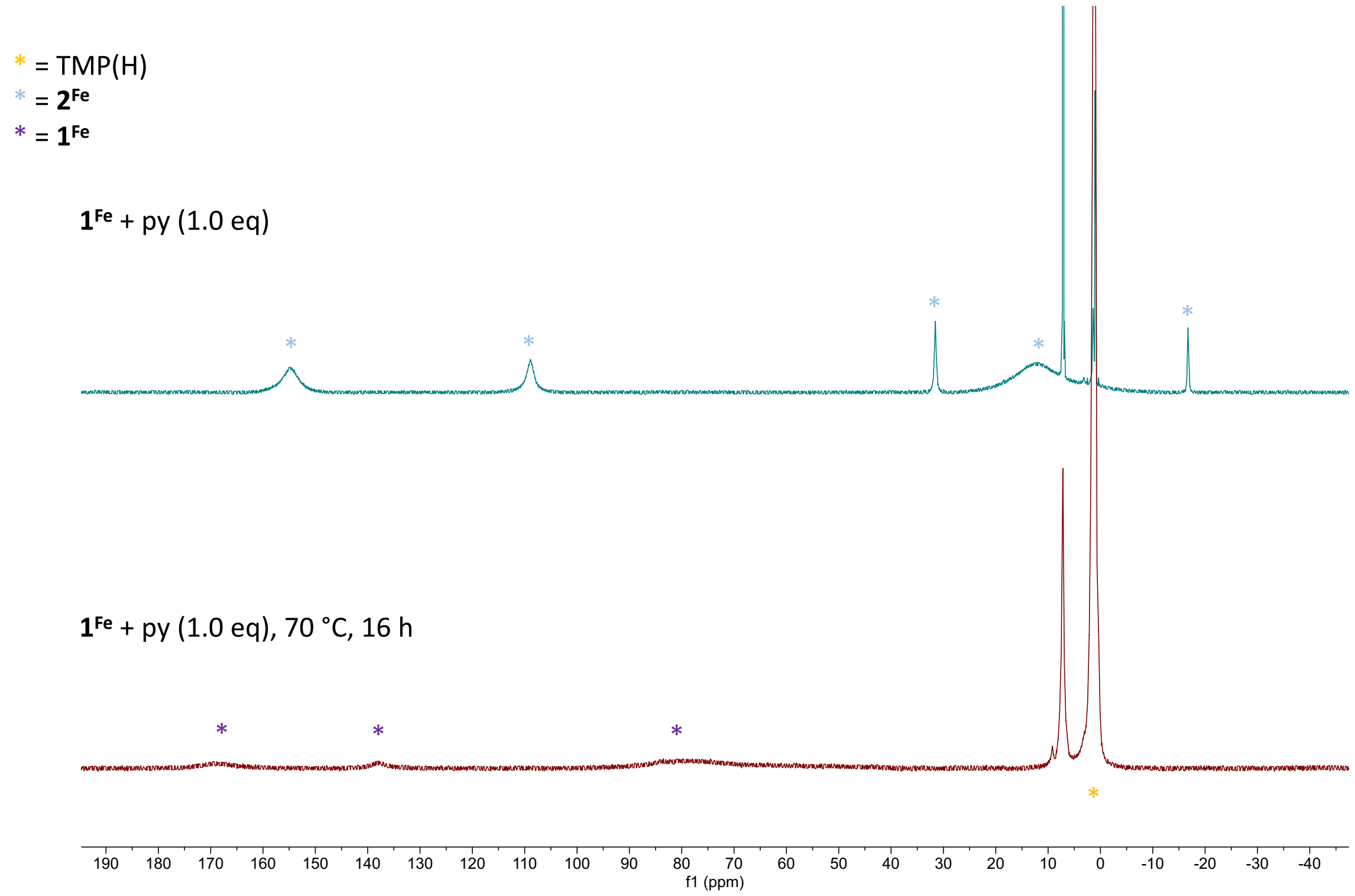

Figure S39. ^{1}H NMR spectra of the reaction of **1**^{Fe} and pyridine (1.0 eq) in C_6D_6 in the 190 to –40 ppm region at each stage of the *in situ* reaction.

Fe(TMP)$_2$ (1^{Fe}) + pyridine (1.0 eq) in d_8-THF

Fe(TMP)$_2$ (**1^{Fe}**) (10.0 mg, 29.7 μmol) was dissolved in d_8-THF (0.5 mL) in a J. Young NMR tube to which pyridine (2.4 μL, 29.7 μmol) was subsequently added. A ^{1}H NMR spectrum was recorded to show formation of the pyridine adduct **2^{Fe}**. After heating at 70 °C for 6 hours, another ^{1}H NMR spectrum was recorded where formation of TMP(H) due to decomposition was visible without any hint of ferration of pyridine.

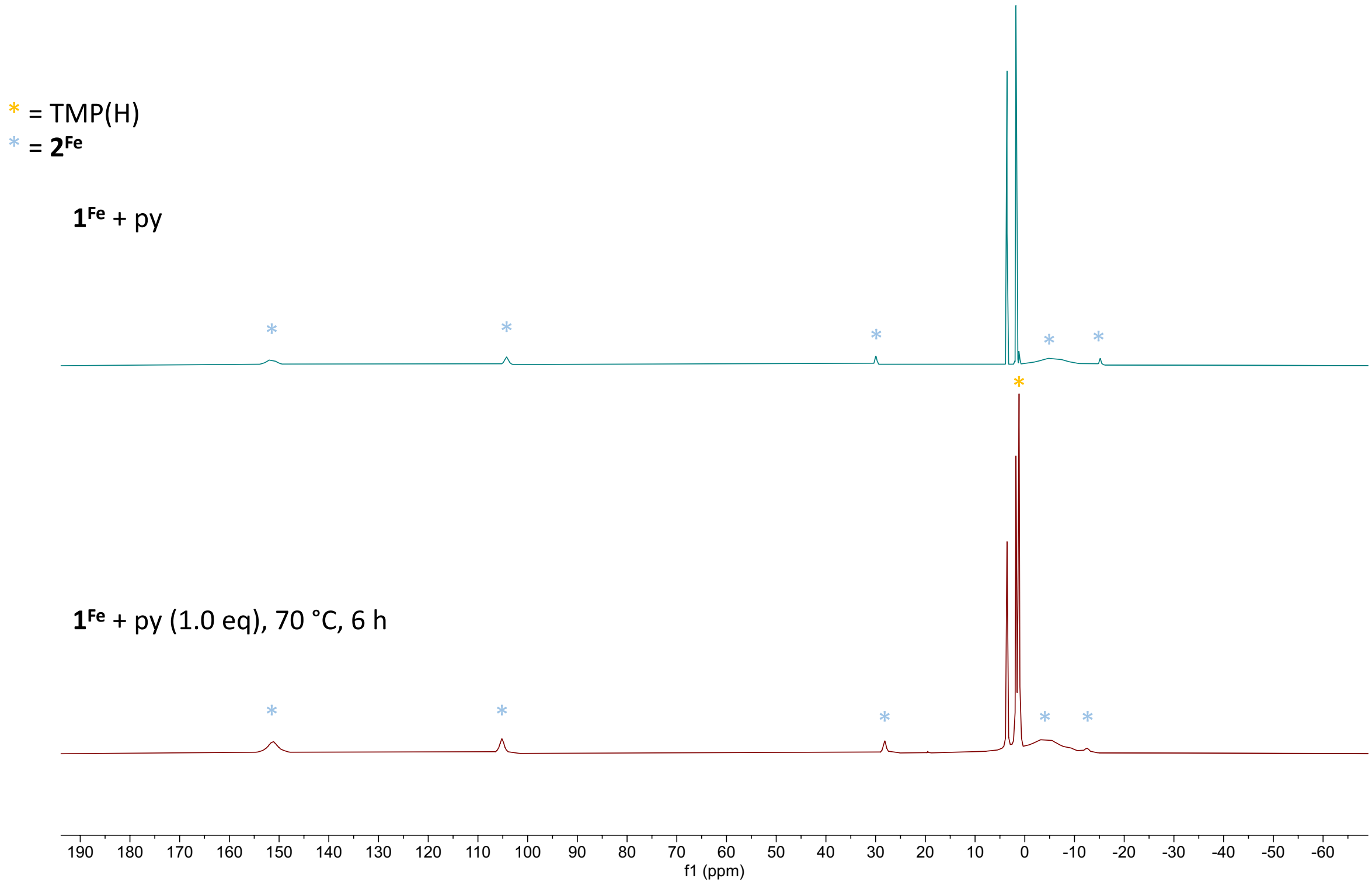

Figure S40. ^{1}H NMR spectra of the reaction of **1^{Fe}** and pyridine (1.0 eq) in d_8-THF in the 190 to –60 ppm region at each stage of the *in situ* reaction.

Co(TMP)$_2$ (1^{Co}) + 3-picoline (1.0 eq) in C_6D_6

Co(TMP)$_2$ ($\mathbf{1}^{\mathbf{Co}}$) (10.0 mg, 29.5 µmol) was dissolved in C_6D_6 (0.5 mL) in a J. Young NMR tube to which 3-picoline (2.9 µL, 29.5 µmol) was subsequently added. After heating at 70 °C for 3 hours, a ^1H NMR spectrum was recorded where full conversion of $\mathbf{1}^{\mathbf{Co}}$ into the cobaltated species **3e** was observed. Based on the integration of the remaining aromatic protons on the pyridinyl ring (96.21, 87.94, 64.68, 64.02, 51.72 and 49.51 ppm), a statistical mixture of both C2- and C6-cobaltated species was found to be present in solution. Further assignment of signals was not possible due to their broad and overlapping nature.

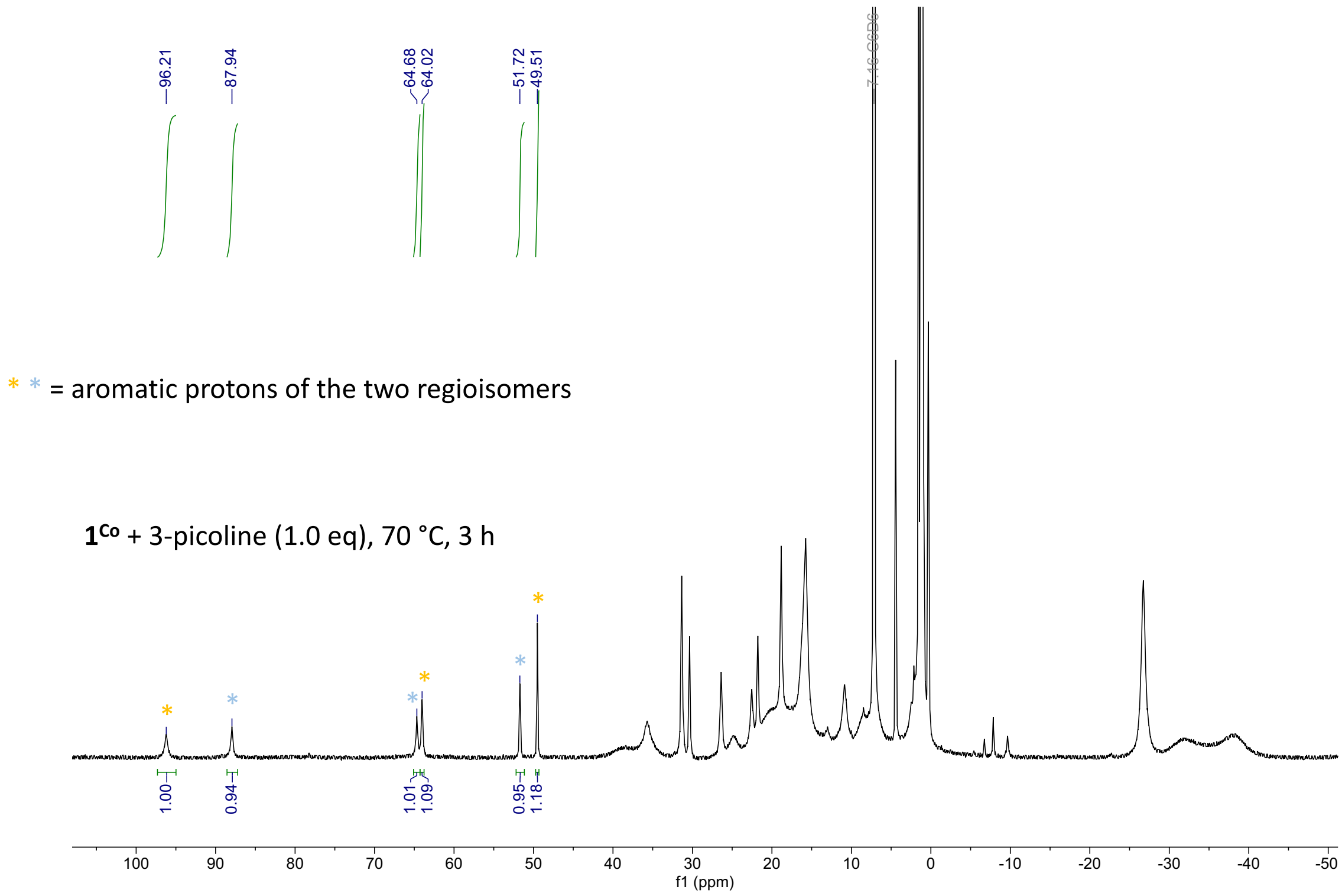

Figure S41. ^1H NMR spectrum of the reaction of $\mathbf{1}^{\mathbf{Co}}$ and 3-picoline (1.0 eq) after heating at 70 °C for 3 h in C_6D_6 in the 100 to –50 ppm region.

Co(TMP)$_2$ (1^{Co}) + 2-methoxypyridine (1.0 eq) in C_6D_6

Co(TMP)$_2$ (**1^{Co}**) (10.0 mg, 29.5 µmol) was dissolved in C_6D_6 (0.5 mL) in a J. Young NMR tube to which 2-methoxypyridine (3.1 µL, 29.5 µmol) was subsequently added. A ^{1}H NMR spectrum was recorded to show formation of the 2-methoxypyridine adduct **2f**. Subsequently the solution was heated at 70 °C for 4 hours. *In situ* NMR monitoring was carried out where a ^{1}H NMR spectrum was recorded every hour. Full conversion of **2f** to the cobaltated species **3f** along with TMP(H) generation was observed.

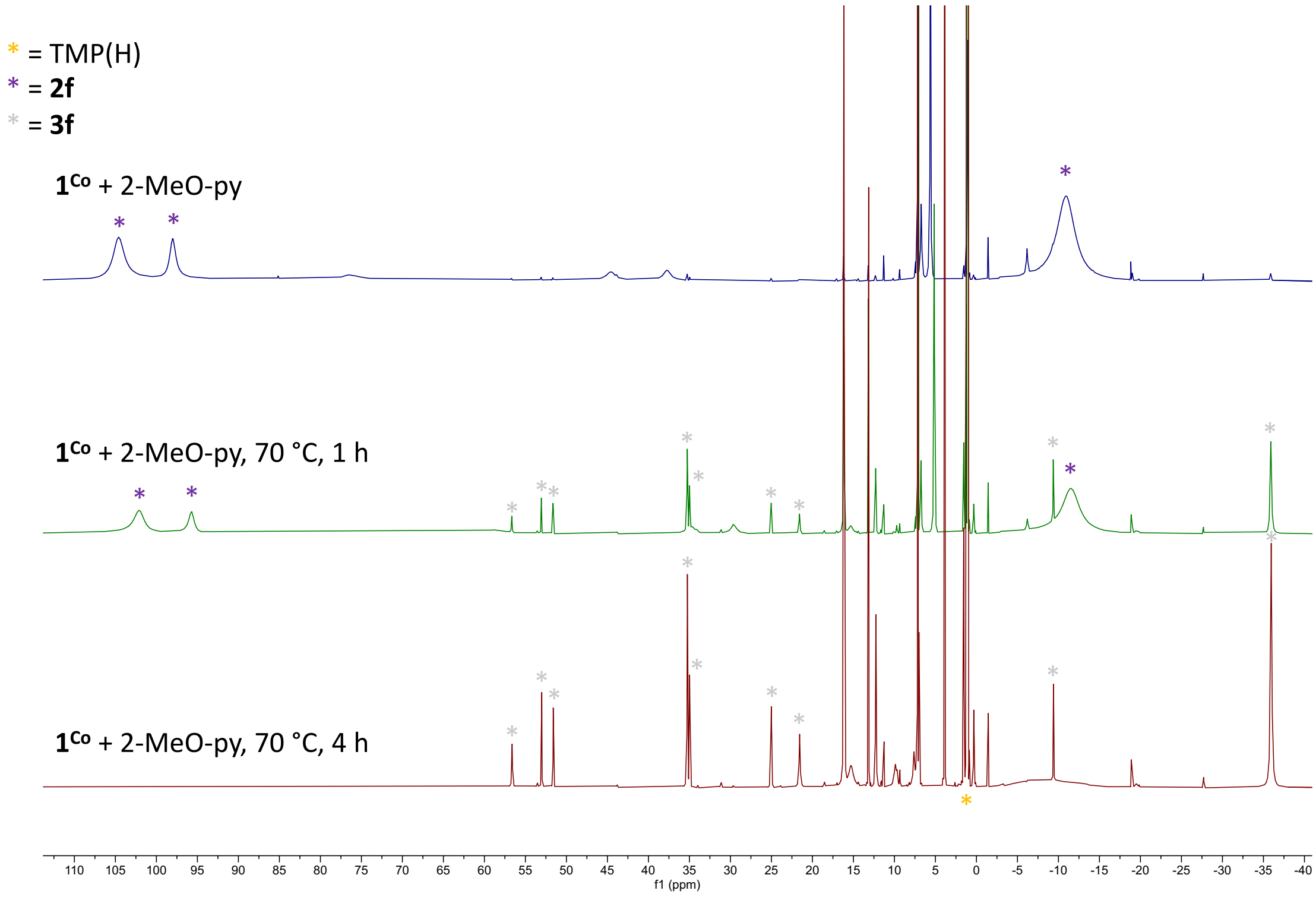

Figure S42. ^{1}H NMR spectra of the reaction of **1^{Co}** and 2-methoxypyridine (1.0 eq) in C_6D_6 in the 110 to –40 ppm region at each stage of the *in situ* reaction.

Co(TMP)$_2$ (1^{Co}) + methyl picolinate (0.5eq) in C_6D_6

Co(TMP)$_2$ (**1**^{Co}) (20.0 mg, 58.9 µmol) was dissolved in C_6D_6 (0.5 mL) in a J. Young NMR tube to which methyl picolinate (3.6 µL, 29.5 µmol) was subsequently added. The solution was left at room temperature for 3 hours. *In situ* NMR monitoring was carried out where a ^{1}H NMR spectrum was recorded every hour. Full conversion of **1**^{Co} to **4** along with TMP(H) generation was observed.

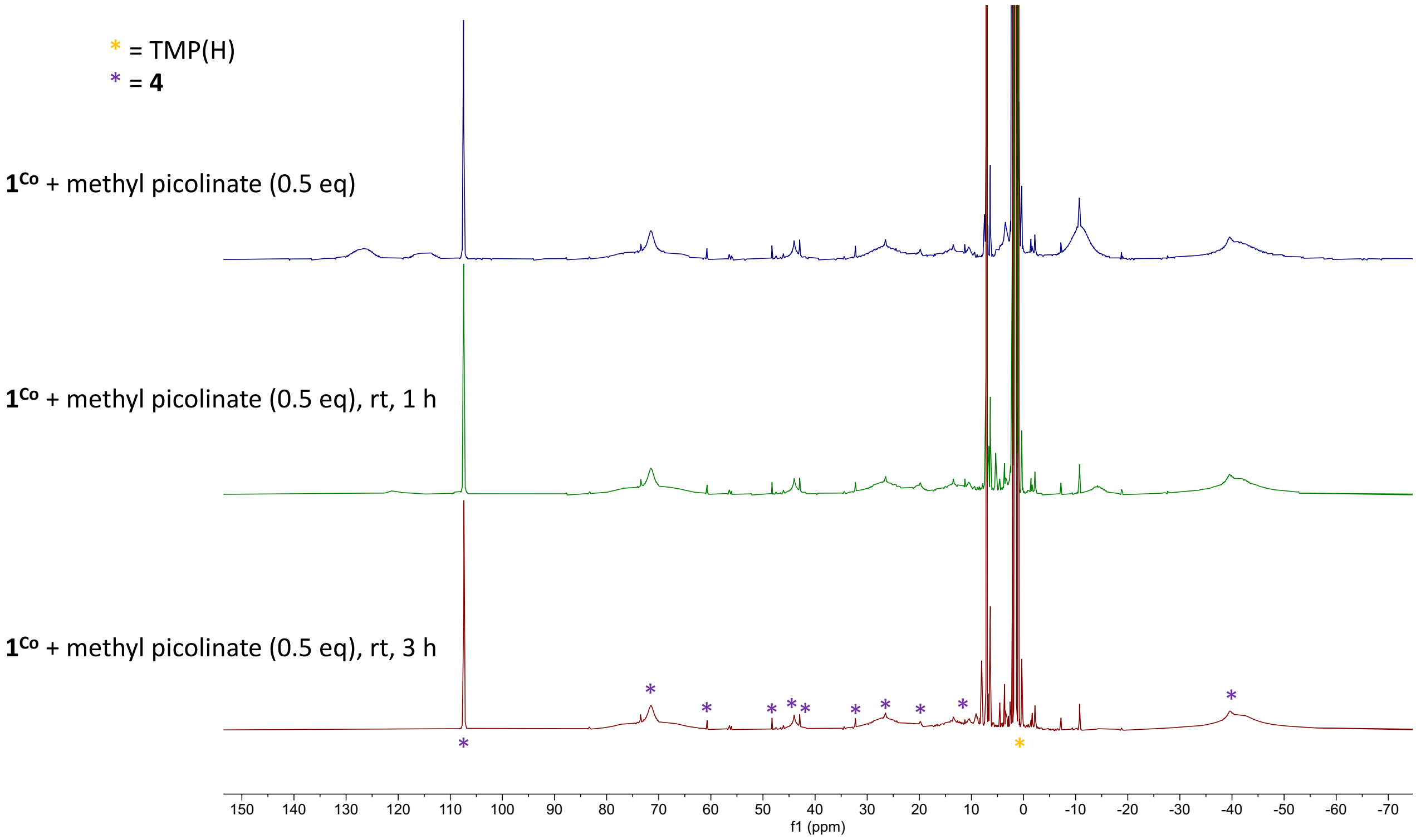

Figure S43. ^{1}H NMR spectra of the reaction of **1**^{Co} and methyl picolinate (0.5 eq) in C_6D_6 in the 150 to –70 ppm region at each stage of the *in situ* reaction.

Co(TMP)$_2$ (1^{Co}) + pyrazine (0.25 eq) in C_6D_6

Co(TMP)$_2$ (**1**^{Co}) (20.0 mg, 58.9 μmol) was dissolved in C_6D_6 (0.5 mL) in a J. Young NMR tube to which pyrazine (1.2 mg, 14.7 μmol) was subsequently added. A ^{1}H NMR spectrum was recorded to reveal the presence of remaining **1**^{Co}, the dimetallated product **6a** along with one or multiple unidentified paramagnetic species which could be intermediates in the metalation process. Subsequently the solution was heated at 70 °C for 4 hours. *In situ* NMR monitoring was carried out where a ^{1}H NMR spectrum was recorded every hour. Full conversion of **1**^{Co} to the di-cobaltated species **6a** along with TMP(H) generation was observed overtime.

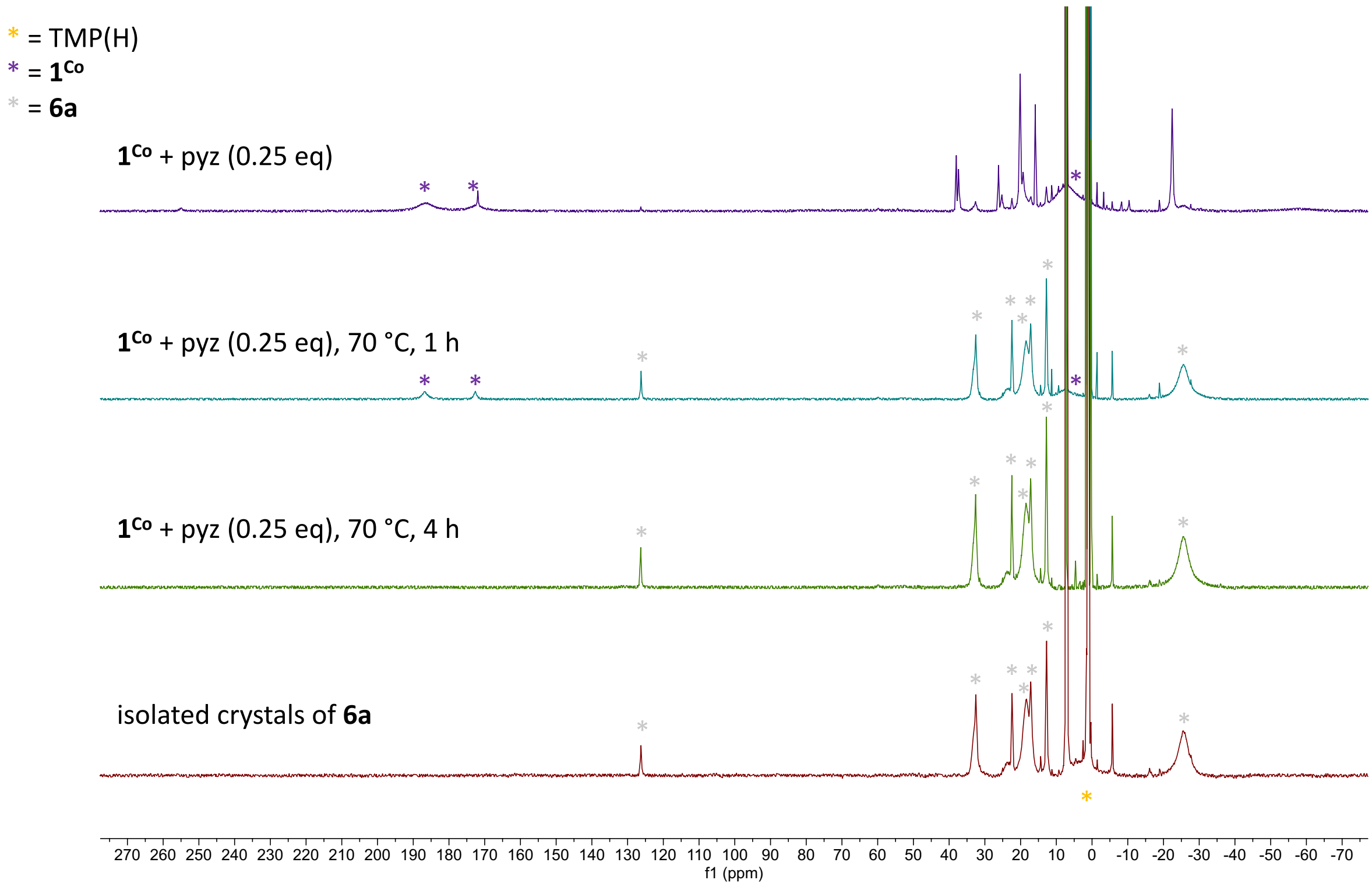

Figure S44. ^{1}H NMR spectra of isolated crystals of **6a** and of the reaction of **1**^{Co} and pyrazine (0.25 eq) in C_6D_6 in the 270 to –70 ppm region at each stage of the *in situ* reaction.

$Co(TMP)_2$ (1^{Co}) + pyrazine (0.5 eq) in C_6D_6

$Co(TMP)_2$ (**1^{Co}**) (8.5 mg, 25.0 µmol) was dissolved in C_6D_6 (0.5 mL) in a J. Young NMR tube to which pyrazine (1.0 mg, 12.5 µmol) was subsequently added. After 3 hours at room temperature, a ^{1}H NMR spectrum was recorded to reveal the presence of the dimetallated product **6a** along with another paramagnetic species postulated to be the C2-monometalated species. The three resonances at 91.23, 68.47 and 52.68 ppm could be tentatively assigned to the three inequivalent aromatic protons on the pyrazinyl ring of the mono-cobaltated species.

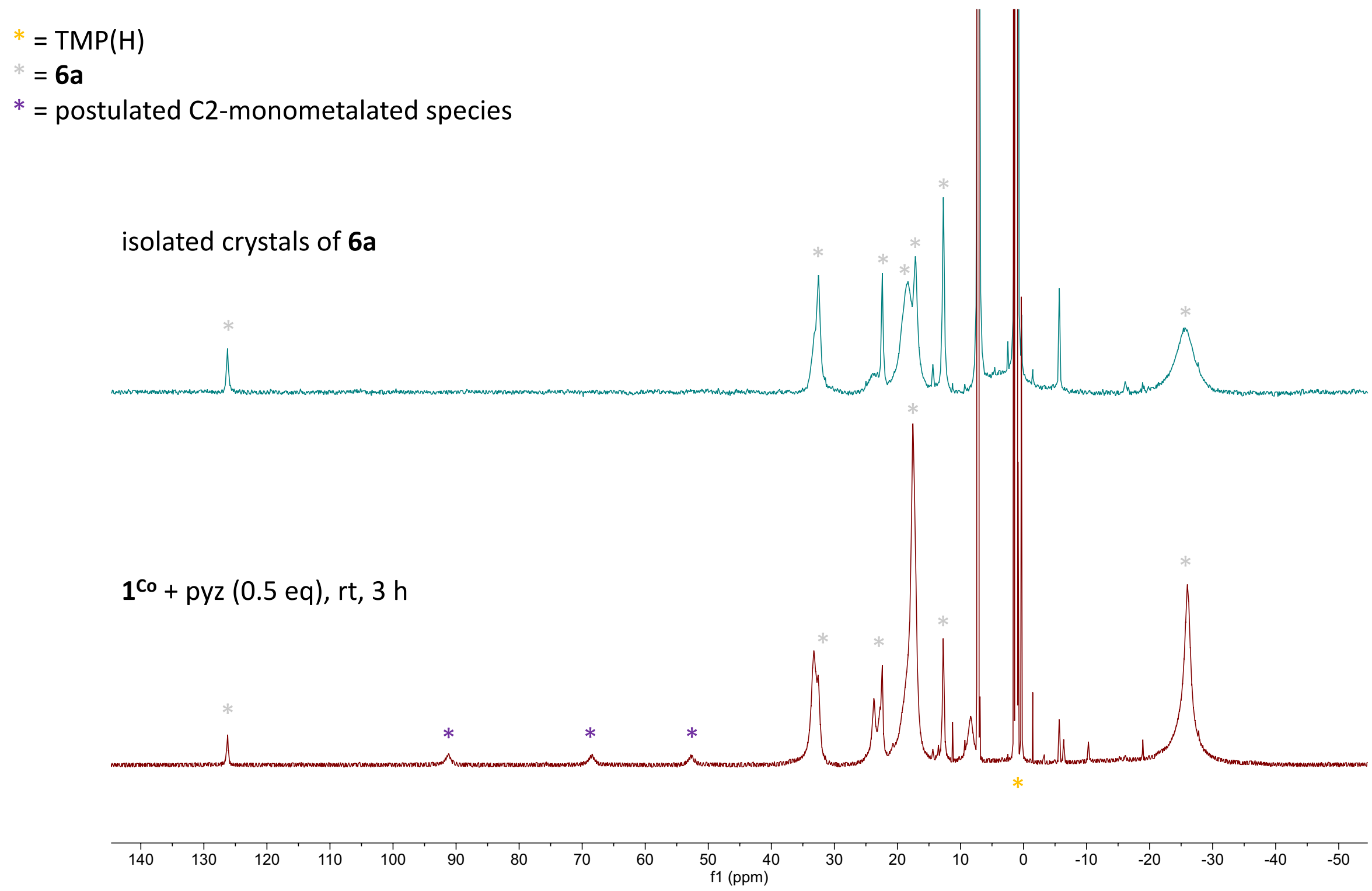

Figure S45. ^{1}H NMR spectra of isolated crystals of **6a** and of the reaction of **1^{Co}** and pyrazine (0.5 eq) after 3 h at rt in C_6D_6 in the 140 to –50 ppm region.

[(py)Co(TMP)$_2$] (2a) + Fe(TMP)$_2$ (1^{Fe}) (1.0 eq) in C_6D_6

[(py)Co(TMP)$_2$] (**2a**) (20.0 mg, 47.8 µmol) and Fe(TMP)$_2$ (**1^{Fe}**) (16.1 mg, 47.8 µmol) were dissolved in C_6D_6 (0.5 mL) in a J. Young NMR tube. A ^{1}H NMR spectrum was recorded to show no reaction took place after 5 hours at room temperature. Subsequently the solution was heated at 70 °C for 3 hours. *In situ* NMR monitoring was carried out. After one hour of heating, formation of the metalated product **3a** was detected along with unreacted **1^{Fe}** and other new unidentified paramagnetic resonances. After another two hours of heating, all signals persisted and decomposition was visible on the wall of the NMR tube.

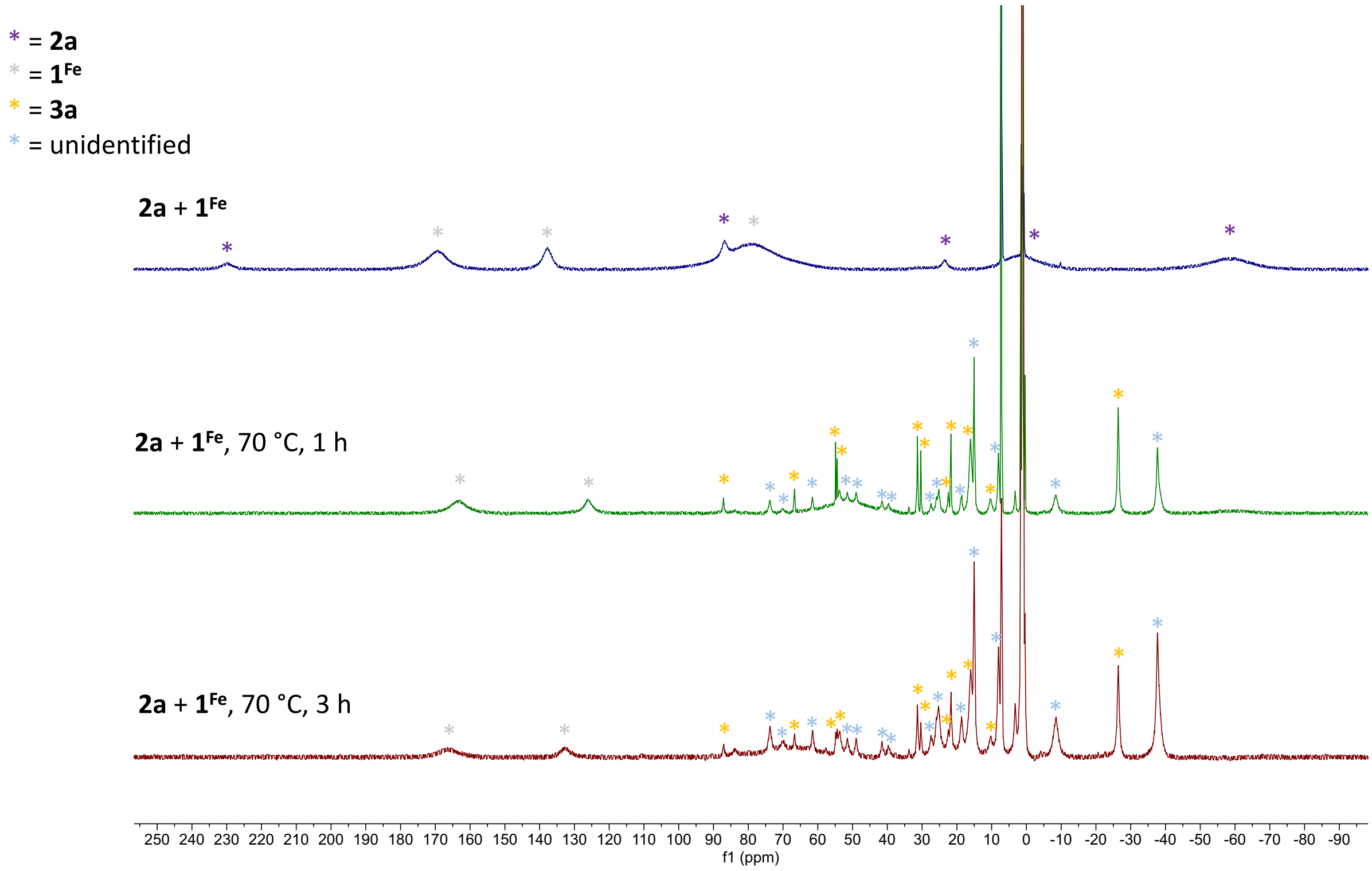

Figure S46. ^{1}H NMR spectra of the reaction of **2a** and **1^{Fe}** (1.0 eq) in C_6D_6 in the 250 to –90 ppm region at each stage of the *in situ* reaction

[(py)Fe(TMP)$_2$] (2^{Fe}) + Co(TMP)$_2$ (1^{Co}) (1.0 eq) in C_6D_6

[(py)Fe(TMP)$_2$] (**2^{Fe}**) (20.0 mg, 48.1 µmol) and Co(TMP)$_2$ (**1^{Co}**) (16.2 mg, 48.1 µmol) were dissolved in C_6D_6 (0.5 mL) in a J. Young NMR tube. A ^{1}H NMR spectrum was recorded to show the migration of pyridine from Fe to Co to yield **2a** and **1^{Fe}**. Subsequently the solution was heated at 70 °C for 3 hours. A ^{1}H NMR spectrum was then recorded to show the formation of the metalated product **3a** along with unreacted **1^{Fe}** and other new unidentified paramagnetic resonances, similar to Figure S46.

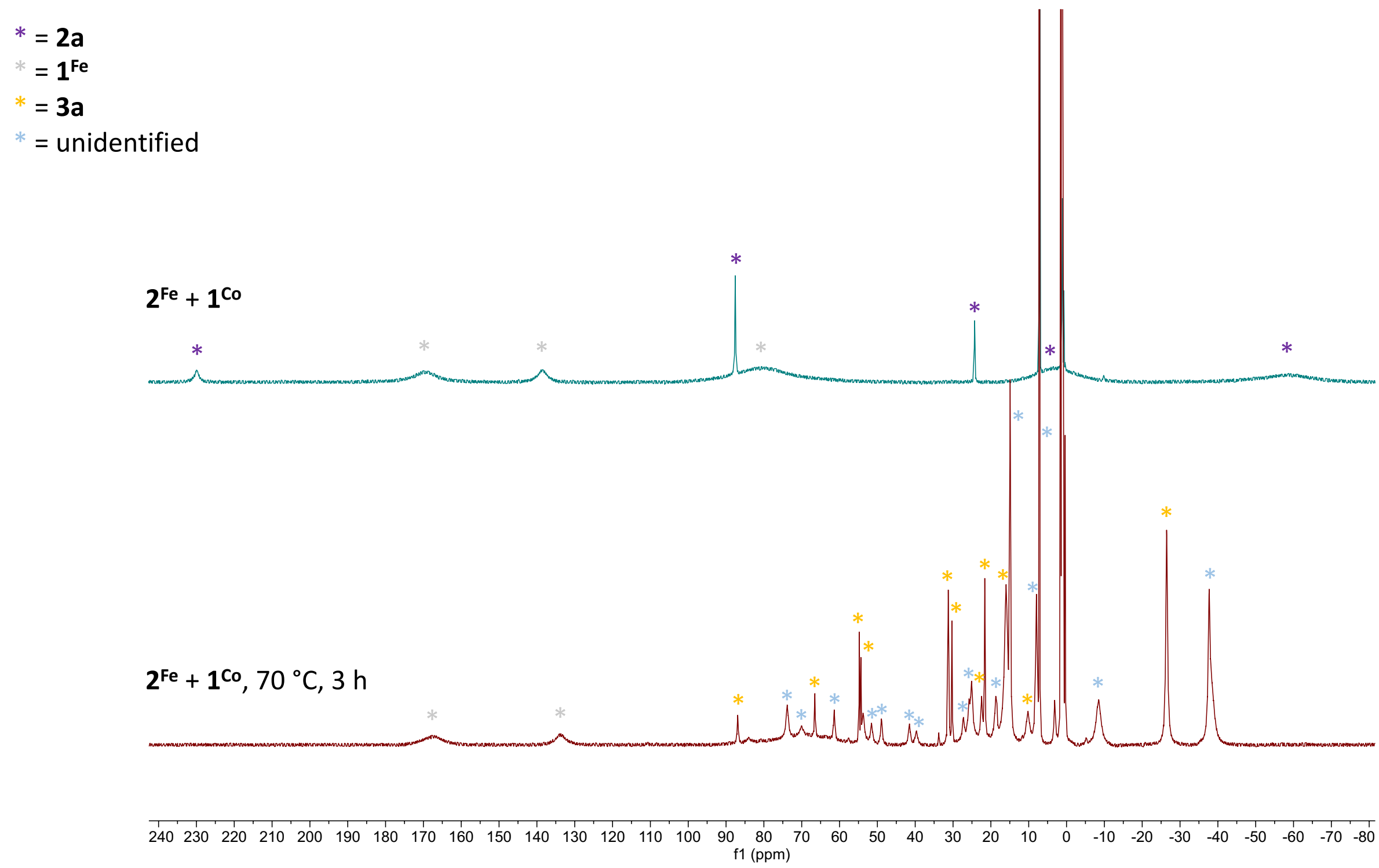

Figure S47. ^{1}H NMR spectra of the reaction of **2^{Fe}** and **1^{Co}** (1.0 eq) in C_6D_6 in the 240 to –80 ppm region at each stage of the *in situ* reaction

[(2-Pyridinyl)Co_2(TMP)$_3$] (3a) + CyBr (5.0 eq) in benzene

[(2-Pyridinyl)Co_2(TMP)$_3$] (**3a**) (61.7mg, 0.1 mmol) was dissolved in benzene (8 mL) in an ampoule to which cyclohexyl bromide (61.6 µL, 0.5 mmol) was subsequently added. The reaction mixture was heated at 70 °C for 16 hours. The reaction was quenched with saturated NH_4Cl aqueous solution. Saturated $NaHCO_3$ aqueous solution was added until the mixture turned basic. Hexamethylbenzene (3.2 mg, 20 µmol, 20 mol%) was added. The mixture was washed with brine and extracted with Et_2O. The combined organic layers were dried over anhydrous $MgSO_4$ and concentrated *in vacuo*. ^{1}H NMR spectrum of the crude showed a 83% yield of the cross-coupled product 2-cyclohexylpyridine against 20 mol% hexamethylbenzene as internal standard.[7]

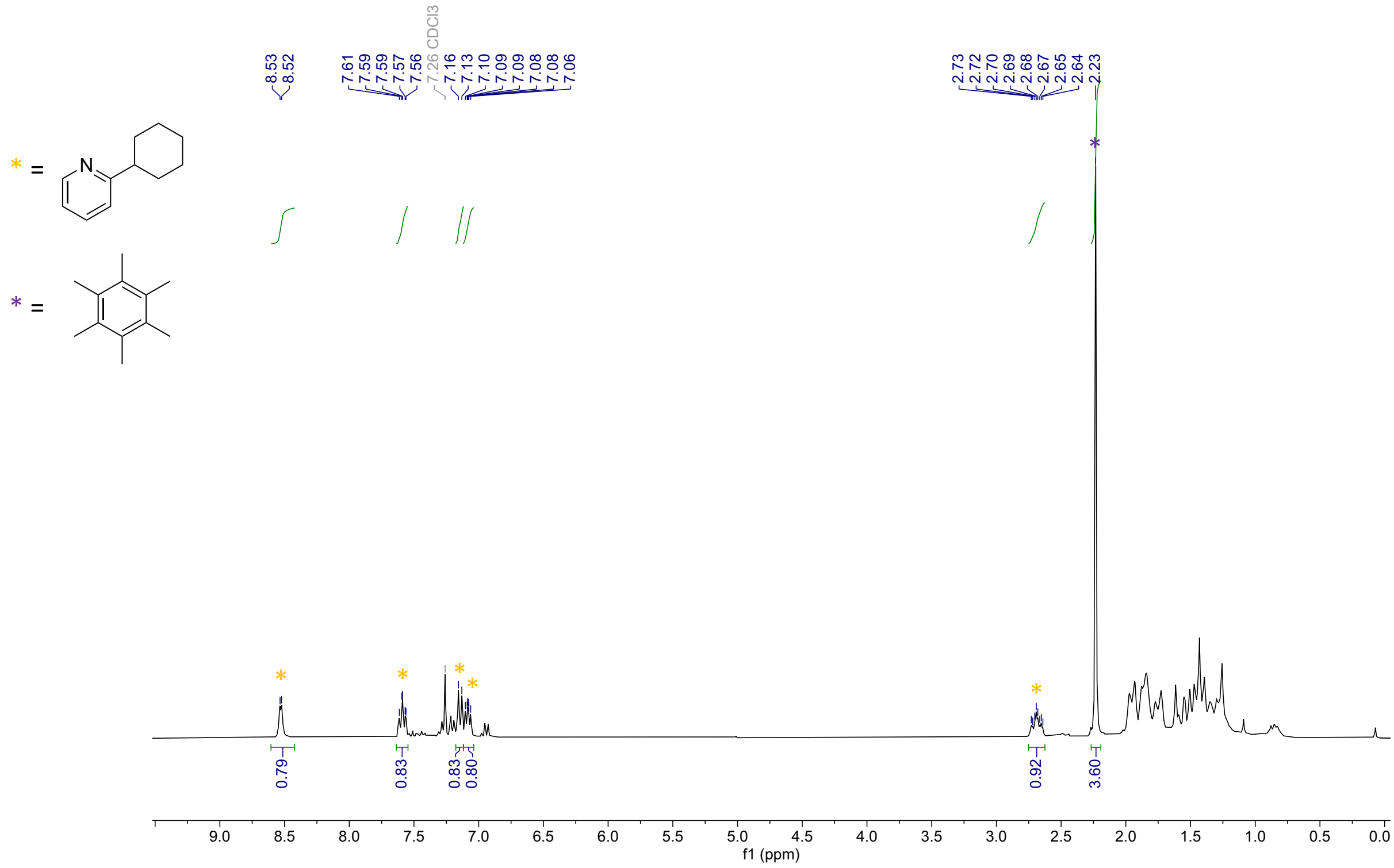

Figure S48. ^{1}H NMR spectrum of the reaction crude of [(2-Pyridinyl)Co_2(TMP)$_3$] (**3a**) + CyBr (5.0 eq) after aqueous workup in $CDCl_3$.

Pyridine + CyBr (3.0 eq) + $Co(TMP)_2$ (1^{Co}) (20 mol%) in benzene

Pyridine (16.1 µL, 0.2 mmol) and cyclohexyl bromide (73.9 µL, 0.6 mmol) were dissolved in benzene (8 mL) in an ampoule to which $Co(TMP)_2$ ($\mathbf{1}^{Co}$) (13.6 mg, 40 µmol, 20 mol%) was subsequently added. The reaction mixture was heated at 70 °C for 16 hours. The reaction was quenched with saturated NH_4Cl aqueous solution. Saturated $NaHCO_3$ aqueous solution was added until the mixture turned basic. Hexamethylbenzene (3.2 mg, 20 µmol, 10 mol%) was added. The mixture was washed with brine and extracted with Et_2O. The combined organic layers were dried over anhydrous $MgSO_4$ and concentrated *in vacuo*. ^1H NMR spectrum of the crude showed a 7% yield of the cross-coupled product 2-cyclohexylpyridine against 10 mol% hexamethylbenzene as internal standard.[7]

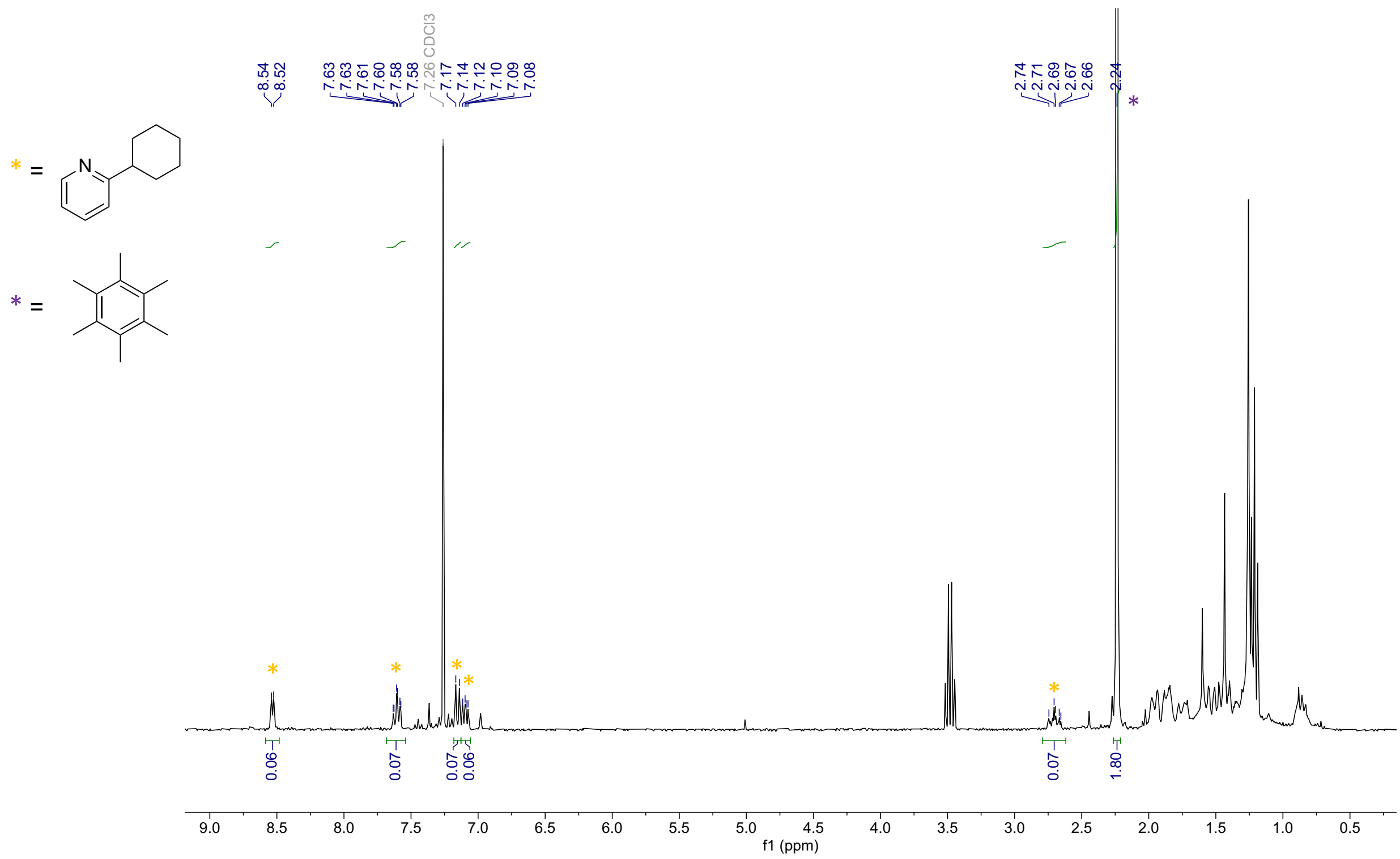

Figure S49. ^1H NMR spectrum of the reaction crude of pyridine + CyBr (3.0 eq) + $Co(TMP)_2$ ($\mathbf{1}^{Co}$) (20 mol%) after aqueous workup in $CDCl_3$.

[(2-Pyridinyl)Co_2(TMP)$_3$] (3a) + styrene (5.0 eq) in C_6D_6

[(2-Pyridinyl)Co_2(TMP)$_3$] (**3a**) (10.0 mg, 16.2 μmol) was dissolved in C_6D_6 (0.5 mL) in a J. Young NMR tube to which styrene (9.3 μL, 81.1 μmol) was subsequently added. A ^{1}H NMR spectrum was recorded to show no reaction took place at room temperature.

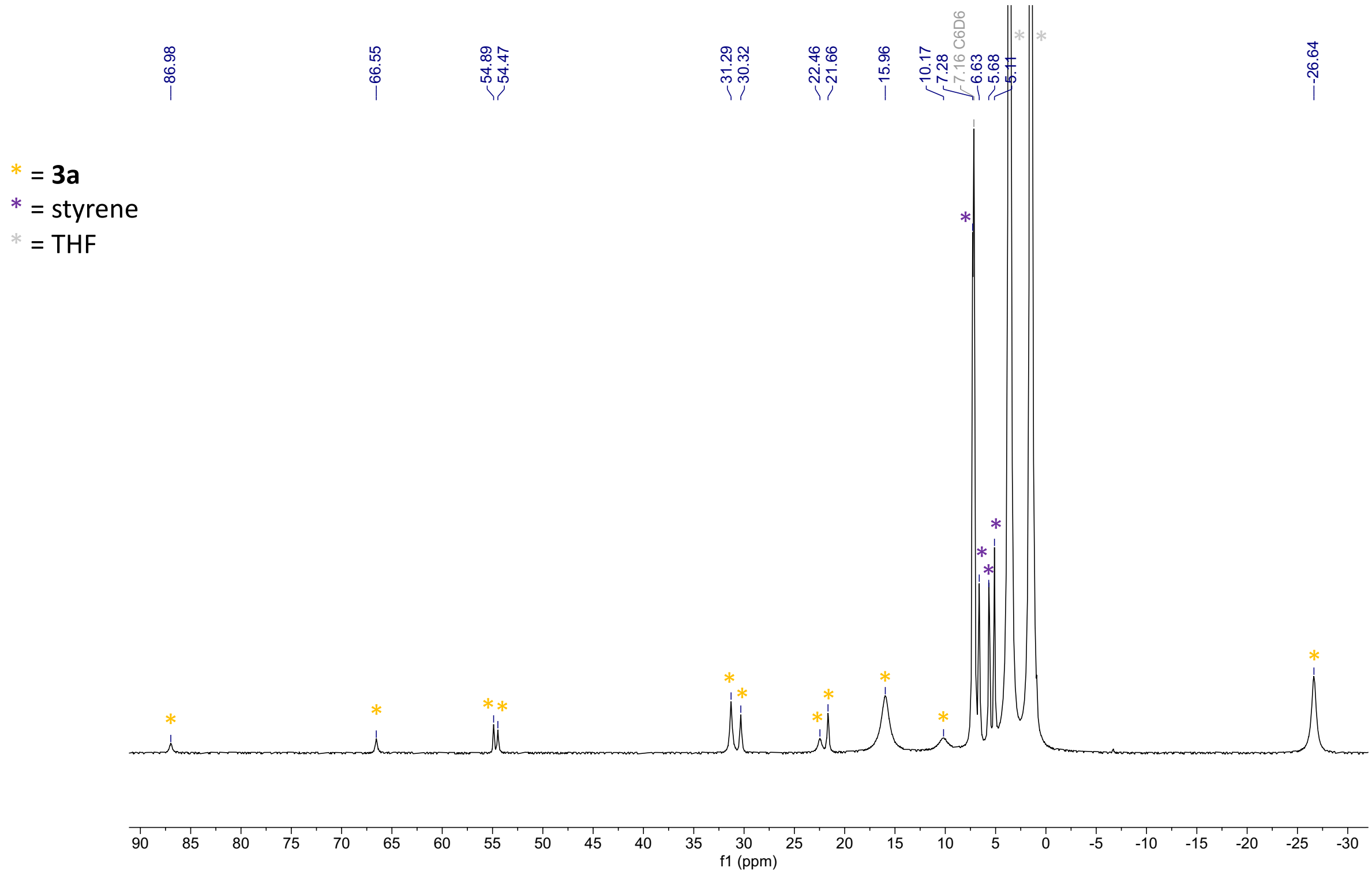

Figure S50. ^{1}H NMR spectrum of the reaction of **3a** and styrene (5.0 eq) at rt in C_6D_6 in the 90 to –30 ppm region.

Subsequently the solution was heated at 70 °C for 16 hours and decomposition was observed. The reaction mixture was exposed to air and filtered through celite with a syringe filter. A ^{1}H NMR spectrum of the filtrate was recorded to reveal the presence of unreacted styrene and TMP(H) and the absence of the addition product.

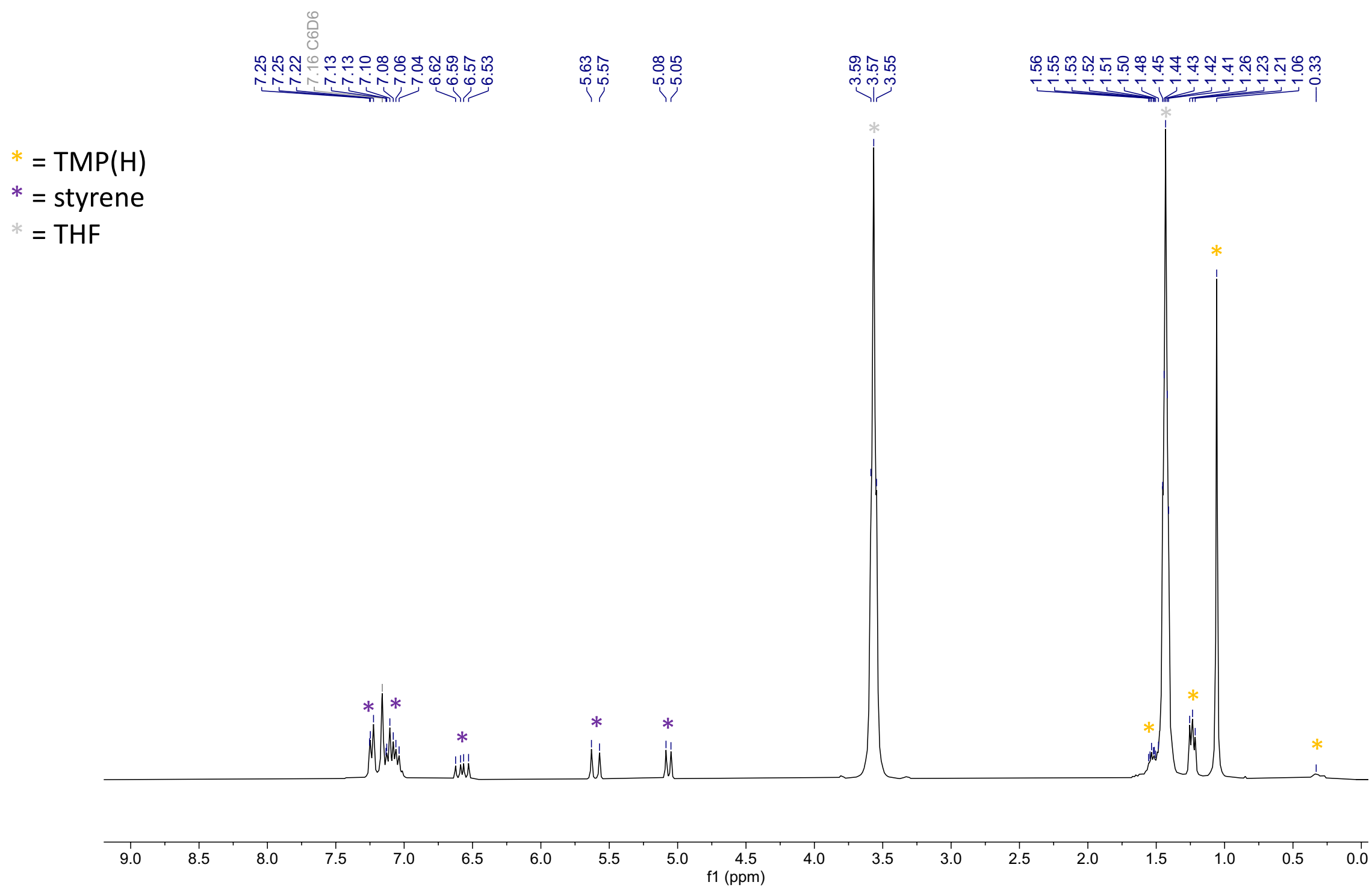

Figure S51. ^{1}H NMR spectrum of the reaction filtrate of **3a** and styrene (5.0 eq) in C_6D_6.

Solution Effective Magnetic Moment Calculations

Compound	Entry	Solvent	Mass of Sample (g)	Mass of Sample + Solvent (g)	Δf Frequency Shift (Hz)	Molar Mass of Sample (g/mol)	μ_{eff} Effective Magnetic Moment (μ_B)	μ_{eff} Average Effective Magnetic Moment (μ_B)
[(py)Fe(TMP)$_2$] (**2^{Fe}**)	1	C_6D_6	0.0049	0.4918	310.18	415.45	5.11	5.18
	2	C_6D_6	0.0065	0.4888	423.78	415.45	5.16	
	3	C_6D_6	0.01	0.4893	683.85	415.45	5.26	
[(py)Co(TMP)$_2$] (**2a**)	1	C_6D_6	0.0065	0.5092	260.96	418.54	4.17	4.06
	2	C_6D_6	0.0048	0.4716	178.28	418.54	3.87	
	3	C_6D_6	0.005	0.4395	227.68	418.54	4.13	
[(2-Methoxy-py)Co(TMP)$_2$] (**2f**)	1	C_6D_6	0.0077	0.511	303.23	448.56	4.28	4.28
	2	C_6D_6	0.0096	0.4211	376.48	448.56	3.87	
	3	C_6D_6	0.0084	0.5065	402.23	448.56	4.68	
[(2-Pyridinyl)Co$_2$(TMP)$_3$] (**3a**)	1	C_6D_6	0.0058	0.4788	120.44	616.71	3.58	3.75
	2	C_6D_6	0.0067	0.492	152.92	616.71	3.79	
	3	C_6D_6	0.0041	0.4678	102.55	616.71	3.88	
[(6-Phenyl-2-pyridinyl)Co$_2$(TMP)$_3$] (**3b**)	1	C_6D_6	0.0088	0.4856	180.95	692.81	3.79	3.56
	2	C_6D_6	0.0047	0.4947	84.37	692.81	3.61	
	3	C_6D_6	0.0056	0.4945	81.46	692.81	3.27	
[(2-$CH_2C_5H_4N$)Co$_2$(TMP)$_3$] (**3c**)	1	C_6D_6	0.0058	0.503	190.88	630.74	4.62	4.68
	2	C_6D_6	0.0063	0.4853	226.72	630.74	4.74	
	3	C_6D_6	0.0087	0.5131	291.4	630.74	4.69	
[(4-Methyl-2-pyridinyl)Co$_2$(TMP)$_3$] (**3d**)	1	C_6D_6	0.0048	0.4918	92.58	630.74	3.55	3.43
	2	C_6D_6	0.0056	0.505	90.67	630.74	3.31	
	3	C_6D_6	0.0044	0.5093	76.41	630.74	3.43	
[(6-Methoxy-2-pyridinyl)Co$_2$(TMP)$_3$] (**3f**)	1	C_6D_6	0.0142	0.6151	256.91	646.74	3.85	3.73
	2	C_6D_6	0.0069	0.5063	128.82	646.74	3.58	
	3	C_6D_6	0.0044	0.4884	94.51	646.74	3.77	
[(4-Methoxy-2-pyridinyl)Co$_2$(TMP)$_3$] (**3g**)	1	C_6D_6	0.0066	0.5025	130.56	646.74	3.67	3.89
	2	C_6D_6	0.005	0.4696	119.84	646.74	3.89	
	3	C_6D_6	0.0071	0.4745	190.44	646.74	4.12	
[(3-Methoxy-2-pyridinyl)Co$_2$(TMP)$_3$] (**3h**)	1	C_6D_6	0.006	0.47	174.56	646.74	4.27	3.82
	2	C_6D_6	0.0049	0.5009	89.23	646.74	3.53	
	3	C_6D_6	0.006	0.489	120.62	646.74	3.65	
[(6-Fluoro-2-pyridinyl)Co$_2$(TMP)$_3$] (**3i**)	1	C_6D_6	0.0055	0.4762	124.46	634.7	3.78	3.69
	2	C_6D_6	0.0061	0.4636	135.63	634.7	3.69	
	3	C_6D_6	0.0045	0.5001	87.33	634.7	3.60	
[(4,6-Difluoro-2-pyridinyl)Co$_2$(TMP)$_3$] (**3j**)	1	C_6D_6	0.0048	0.4888	89.96	652.69	3.55	3.72
	2	C_6D_6	0.0074	0.4892	174.55	652.69	3.95	
	3	C_6D_6	0.0057	0.5074	110.09	652.69	3.66	
	1	C_6D_6	0.0067	0.4863	166.57	634.7	3.98	3.98

[(3-Fluoro-2-pyridinyl)Co_2(TMP)$_3$] (**3k**)	2	C_6D_6	0.006	0.499	143.45	634.7	3.96	
	3	C_6D_6	0.0059	0.5243	136.4	634.7	3.99	
[(8-Caffeinyl)Co_2(TMP)$_3$] (**3l**)	1	C_6D_6	0.0077	0.4869	236.11	731.8	4.73	4.68
	2	C_6D_6	0.0086	0.5163	230.41	731.8	4.56	
	3	C_6D_6	0.0082	0.494	251.75	731.8	4.76	
[{PhN=(Ph)C}Co_2(TMP)$_3$] (**3m**)	1	C_6D_6	0.0113	0.5808	199.77	718.85	3.91	3.81
	2	C_6D_6	0.0108	0.5401	190.97	718.85	3.78	
	3	C_6D_6	0.006	0.4879	113.99	718.85	3.74	
[(MeO)Co_2(TMP)$_3$] (**4**)	1	C_6D_6	0.0087	0.4849	226.24	569.65	3.84	3.94
	2	C_6D_6	0.0052	0.3684	196.62	569.65	4.04	
	3	C_6D_6	0.0066	0.5244	167.27	569.65	3.95	
[{(Ph)(TMP)C=N}Co_2(TMP)$_3$] (**5**)	1	C_6D_6	0.0045	0.6045	72.42	781.99	4.00	4.07
	2	C_6D_6	0.0043	0.4734	94.78	781.99	4.13	
	3	C_6D_6	0.005	0.4234	120.1	781.99	4.08	
[(2,5-$C_4H_4N_2$-pyz)Co_4(TMP)$_6$] (**6a**)	1	d_8-THF	0.0054	0.5121	89.21	1155.31	4.47	4.29
	2	d_8-THF	0.0073	0.5406	104.66	1155.31	4.29	
	3	d_8-THF	0.0052	0.5252	69.22	1155.31	4.10	
[(4-$C_4H_3N_2$-pym)Co_2(TMP)$_3$] (**7b**)	1	C_6D_6	0.0056	0.4879	113.24	617.7	3.57	3.74
	2	C_6D_6	0.0051	0.5272	105.56	617.7	3.75	
	3	C_6D_6	0.0048	0.4802	119.1	617.7	3.91	
[(3-$C_4H_3N_2$-pdz)Co_2(TMP)$_3$] (**7c**)	1	C_6D_6	0.0041	0.5321	85.3	617.7	3.78	3.61
	2	C_6D_6	0.0033	0.4377	68.28	617.7	3.44	
	3	C_6D_6	0.0049	0.4556	107.9	617.7	3.60	
[(2-Quinoxalinyl)Co_2(TMP)$_3$] (**7d**)	1	C_6D_6	0.0077	0.5003	149.02	667.76	3.68	3.66
	2	C_6D_6	0.0158	0.5182	259.79	667.76	3.44	
	3	C_6D_6	0.0042	0.4877	92.22	667.76	3.87	
[(4-Quinazolinyl)Co_2(TMP)$_3$] (**7e**)	1	C_6D_6	0.0089	0.4763	168.31	667.76	3.55	3.74
	2	C_6D_6	0.007	0.5114	154.12	667.76	3.95	
	3	C_6D_6	0.0061	0.4559	132.78	667.76	3.72	

X-Ray Crystallographic Figures and Data

X-Ray Figures

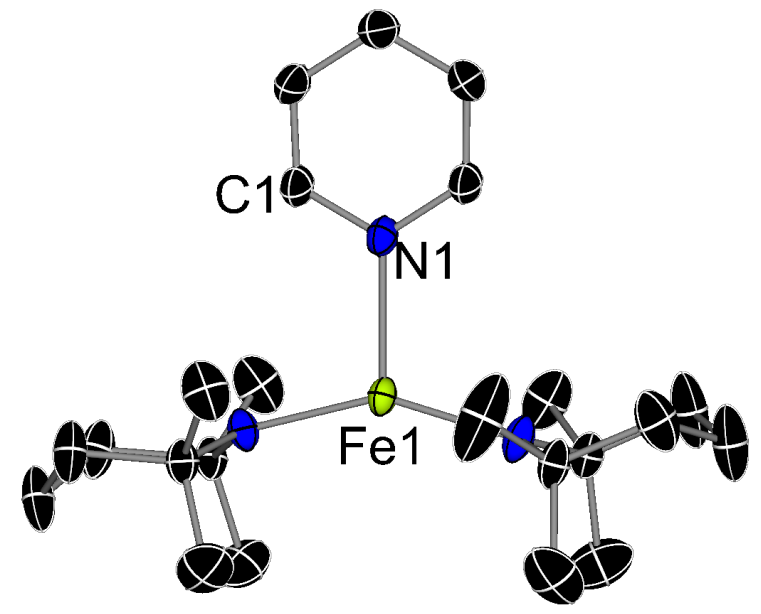

Figure S52. Molecular structure of [(py)Fe(TMP)$_2$] (**2^{Fe}**). Hydrogen atoms and disorder present in TMP ligands omitted for clarity. Ellipsoids displayed at 50% probability level. Due to the severity of the twinning and the amount of restraints necessary to refine the model (incomplete disorder of TMP), the structure is reported for connectivity purposes only.

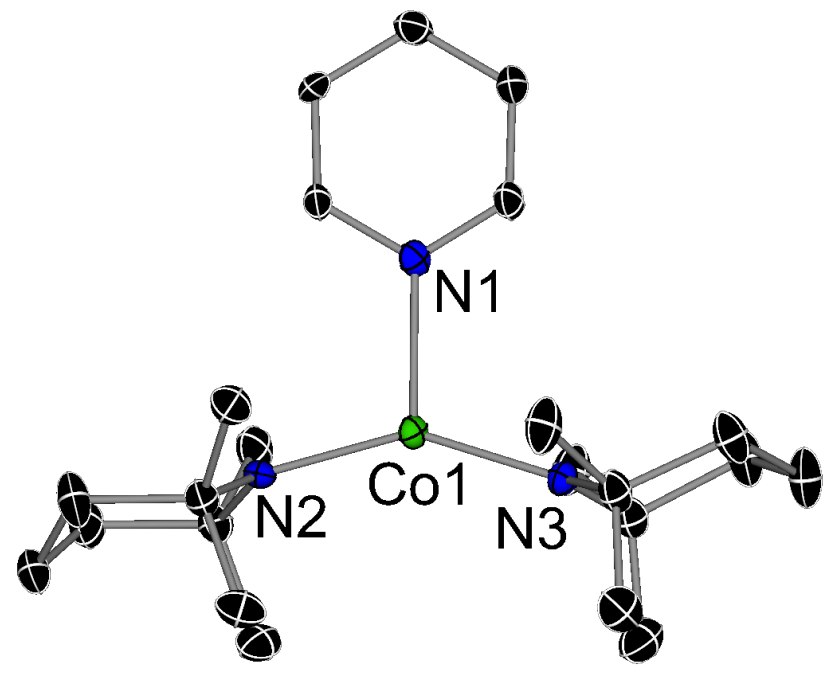

Figure S53. Molecular structure of [(py)Co(TMP)$_2$] (**2a**). Hydrogen atoms omitted for clarity. Thermal ellipsoids displayed at 50% probability level.

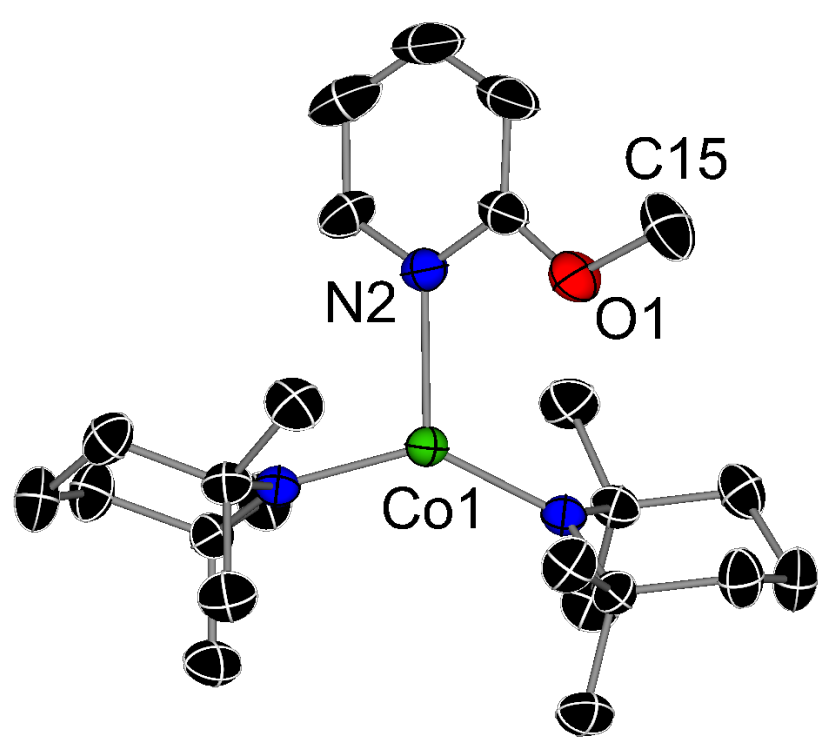

Figure S54. Molecular structure of [(2-methoxy-py)Co(TMP)$_2$] (**2f**). Hydrogen atoms omitted for clarity. Thermal ellipsoids displayed at 50% probability level.

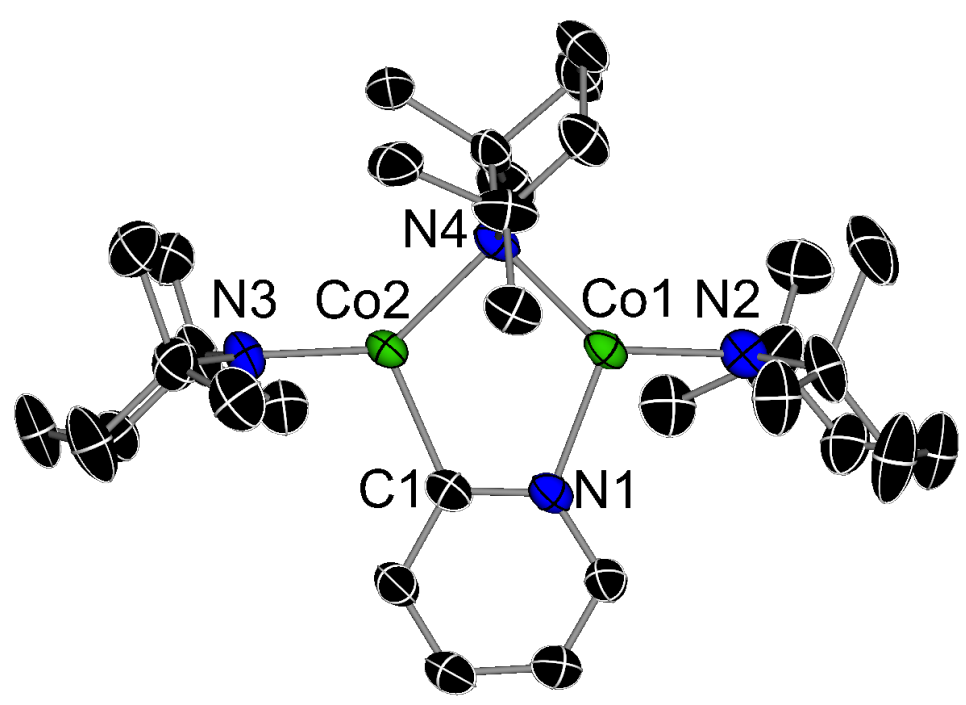

Figure S55. Molecular structure of [(2-pyridinyl)Co$_2$(TMP)$_3$] (**3a**). Hydrogen atoms omitted for clarity. Thermal ellipsoids displayed at 50% probability level.

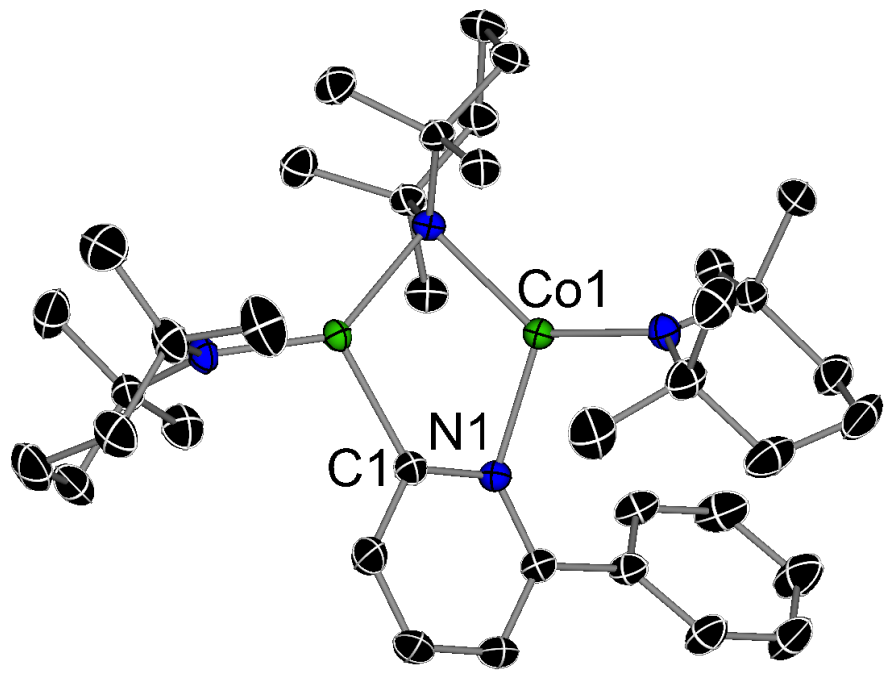

Figure S56. Molecular structure of [(6-phenyl-2-pyridinyl)$Co_2(TMP)_3$] (**3b**). Hydrogen atoms omitted for clarity. Thermal ellipsoids displayed at 50% probability level.

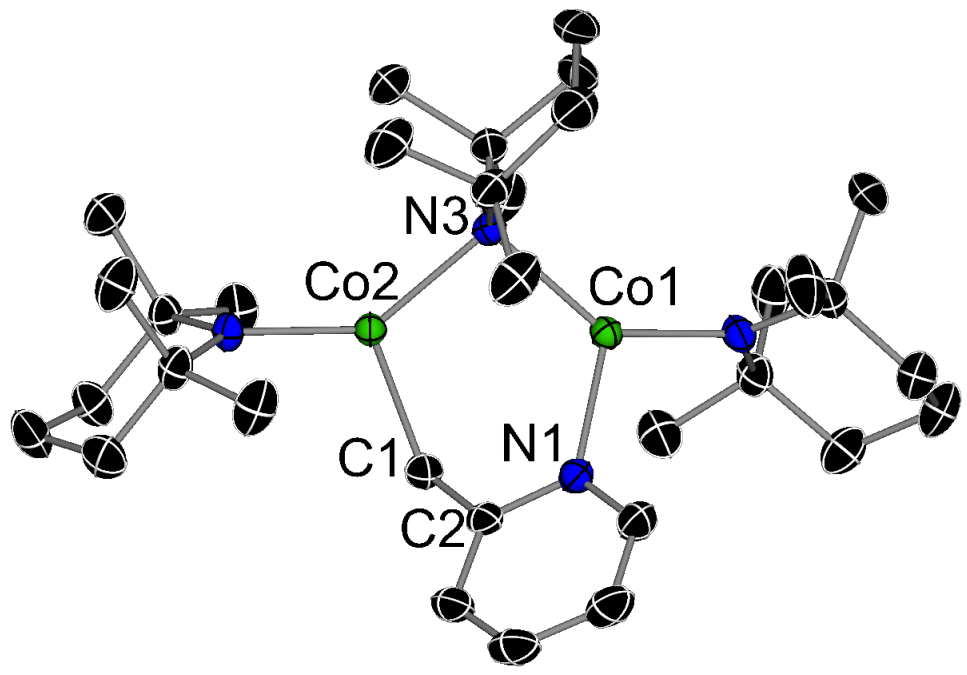

Figure S57. Molecular structure of [(2-$CH_2C_5H_4N$)$Co_2(TMP)_3$] (**3c**). Hydrogen atoms omitted for clarity. Thermal ellipsoids displayed at 50% probability level.

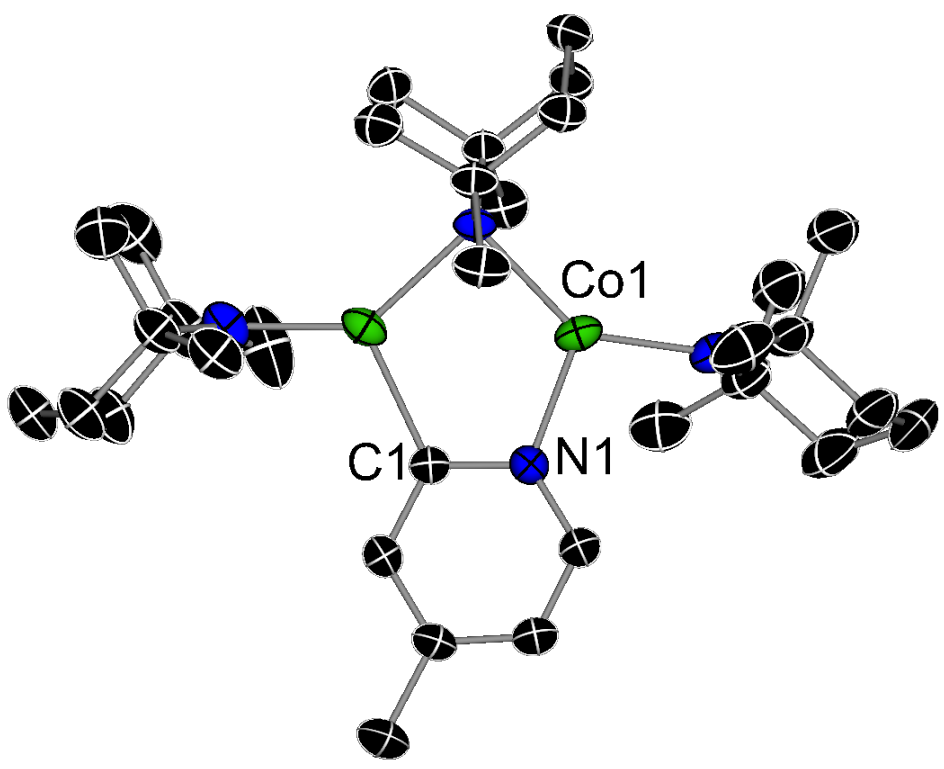

Figure S58. Molecular structure of [(4-methyl-2-pyridinyl)$Co_2(TMP)_3$] (**3d**). Hydrogen atoms omitted for clarity. Thermal ellipsoids displayed at 50% probability level.

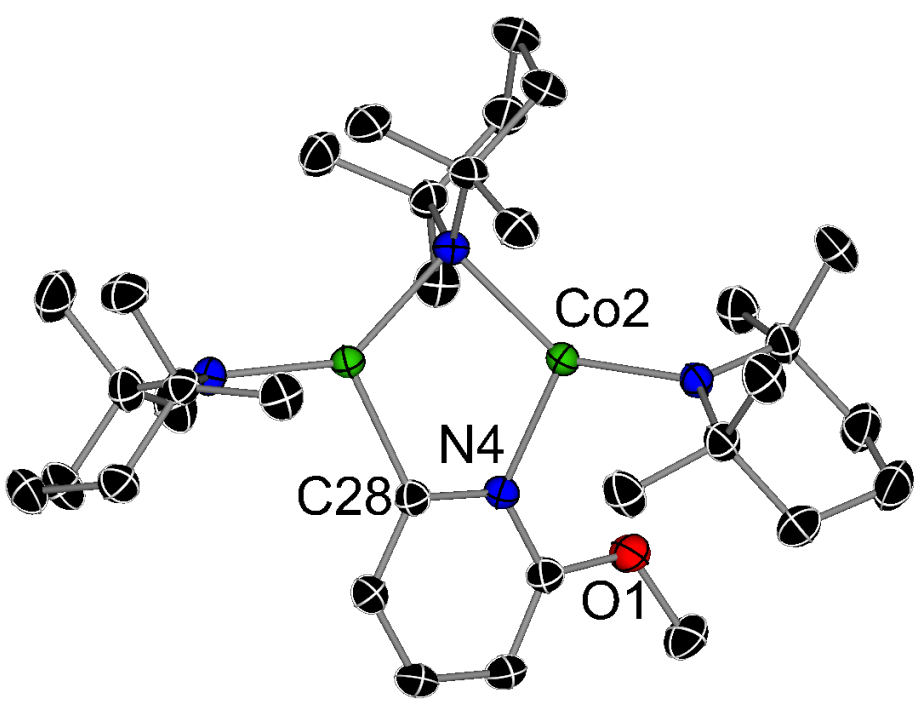

Figure S59. Molecular structure of [(6-methoxy-2-pyridinyl)$Co_2(TMP)_3$] (**3f**). Hydrogen atoms omitted for clarity. Thermal ellipsoids displayed at 50% probability level.

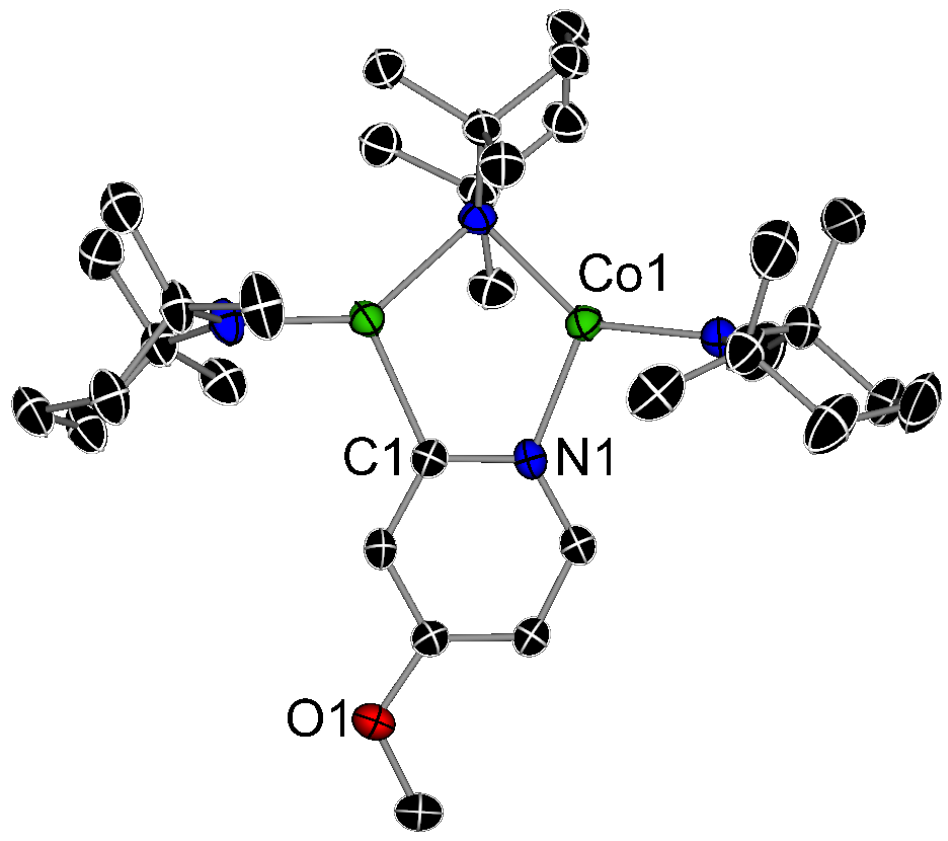

Figure S60. Molecular structure of [(4-methoxy-2-pyridinyl)$Co_2(TMP)_3$] (**3g**). Hydrogen atoms omitted for clarity. Thermal ellipsoids displayed at 50% probability level.

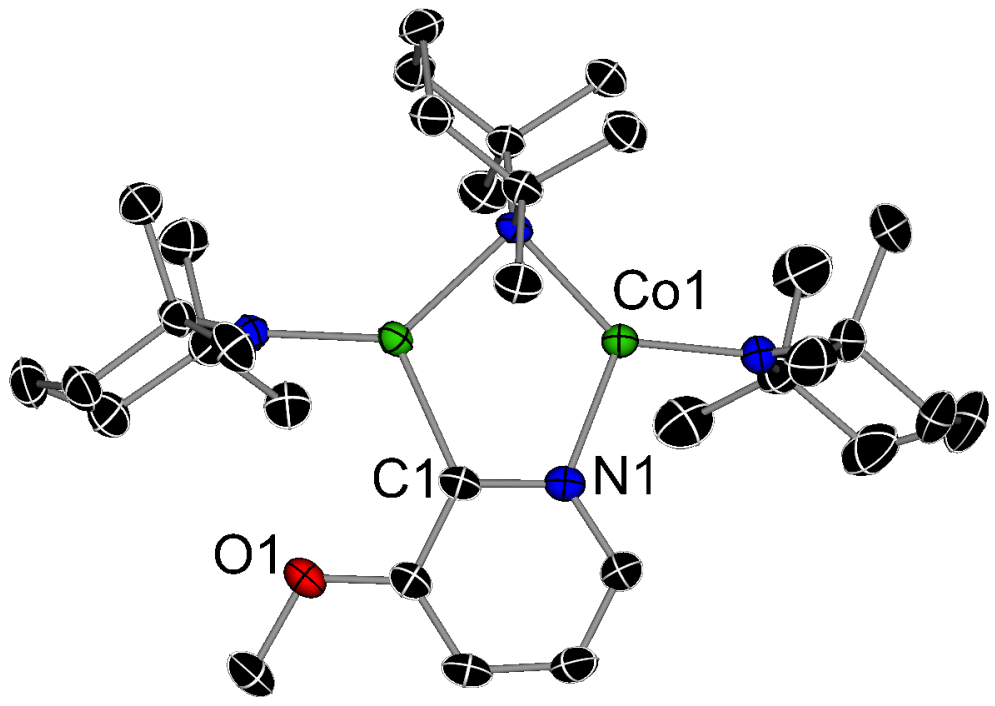

Figure S61. Molecular structure of [(3-methoxy-2-pyridinyl)$Co_2(TMP)_3$] (**3h**). Hydrogen atoms omitted for clarity. Thermal ellipsoids displayed at 50% probability level.

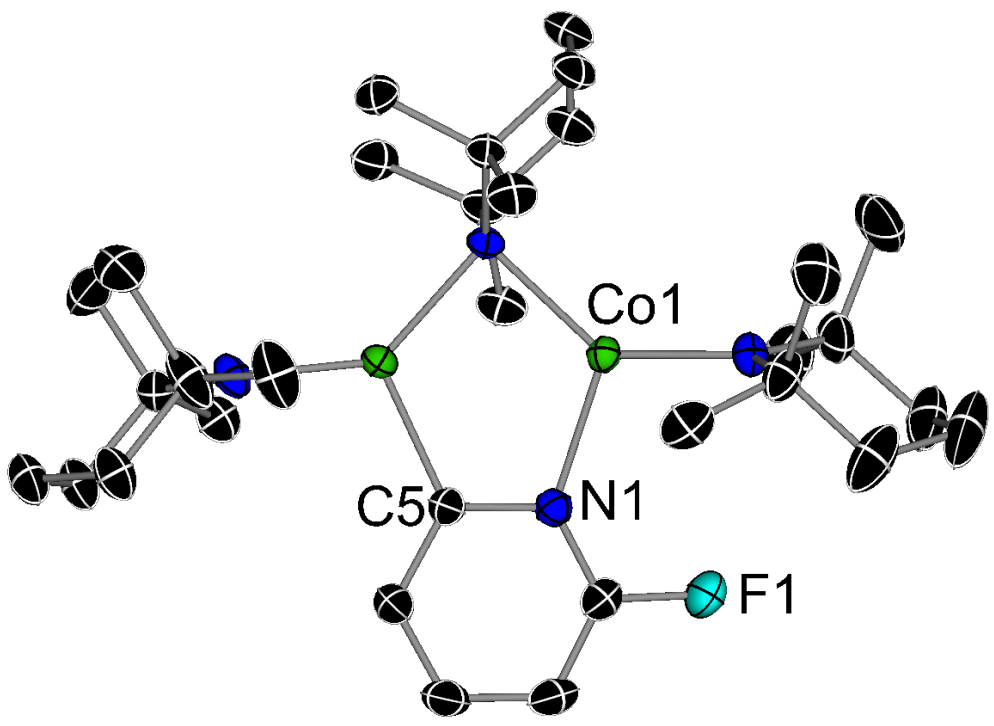

Figure S62. Molecular structure of [(6-fluoro-2-pyridinyl)$Co_2(TMP)_3$] (**3i**). Hydrogen atoms omitted for clarity. Thermal ellipsoids displayed at 50% probability level.

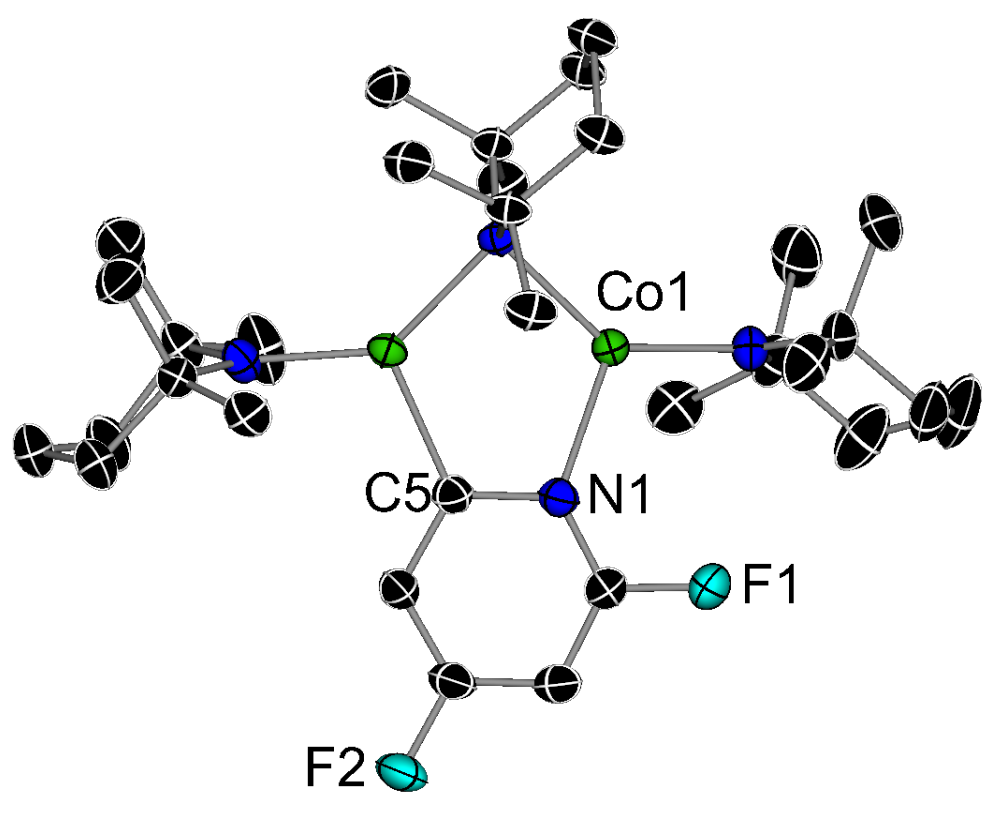

Figure S63. Molecular structure of [(4,6-difluoro-2-pyridinyl)$Co_2(TMP)_3$] (**3j**). Hydrogen atoms omitted for clarity. Thermal ellipsoids displayed at 50% probability level.

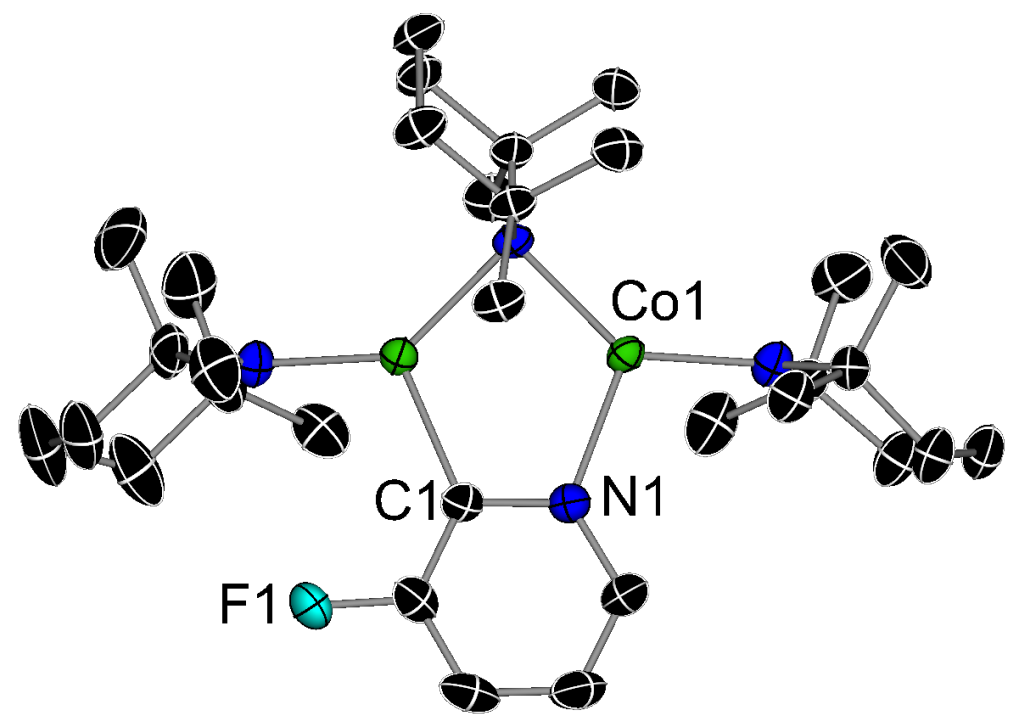

Figure S64. Molecular structure of [(3-fluoro-2-pyridinyl)$Co_2(TMP)_3$] (**3k**). Hydrogen atoms omitted for clarity. Thermal ellipsoids displayed at 50% probability level.

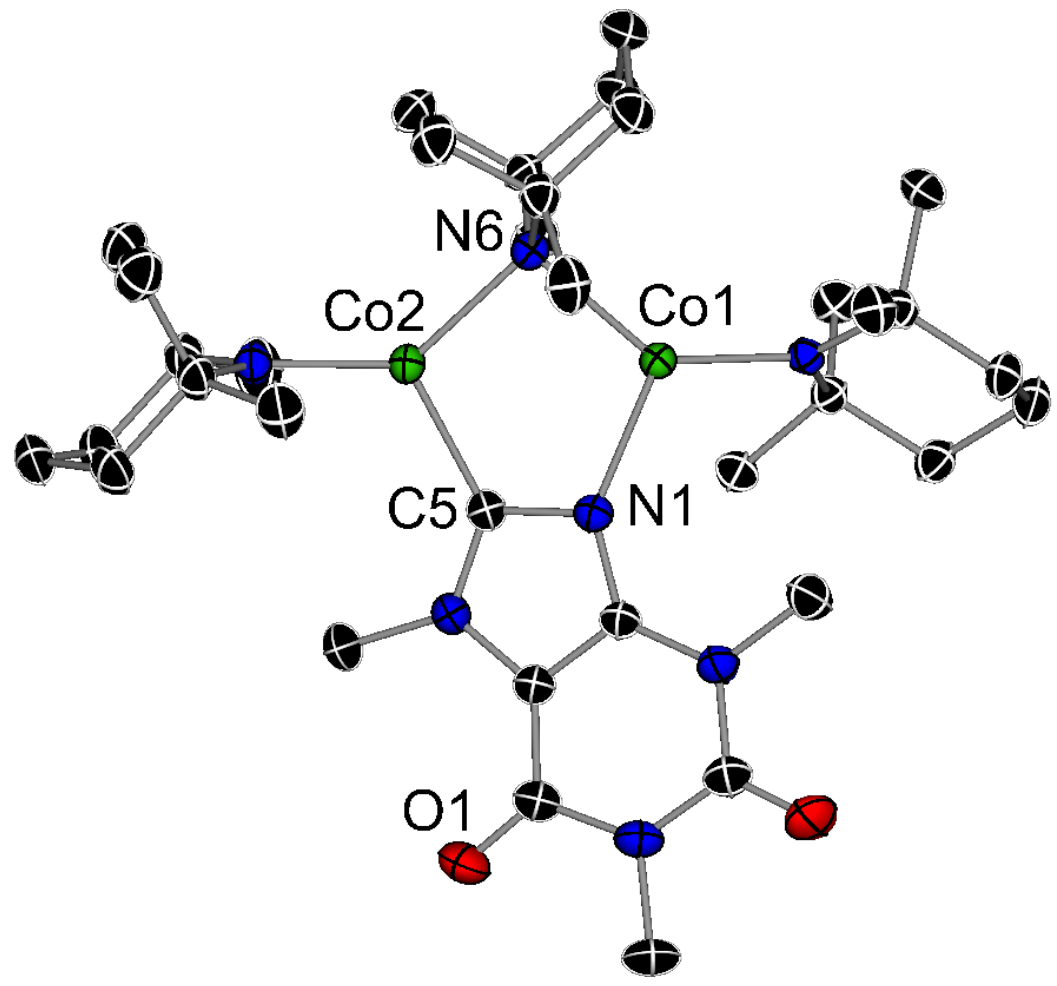

Figure S65. Molecular structure of [(8-caffeinyl)$Co_2(TMP)_3$] (**3l**). Hydrogen atoms omitted for clarity. Thermal ellipsoids displayed at 50% probability level.

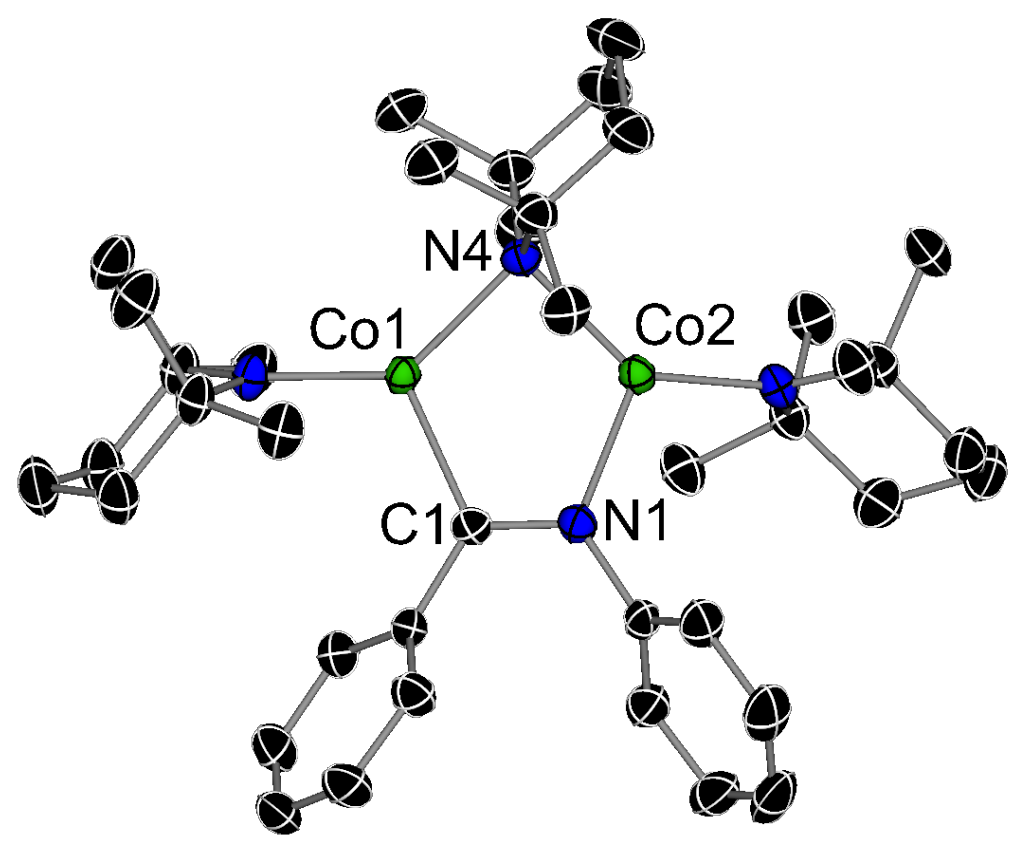

Figure S66. Molecular structure of [{PhN=(Ph)C}$Co_2(TMP)_3$] (**3m**). Hydrogen atoms omitted for clarity. Thermal ellipsoids displayed at 50% probability level.

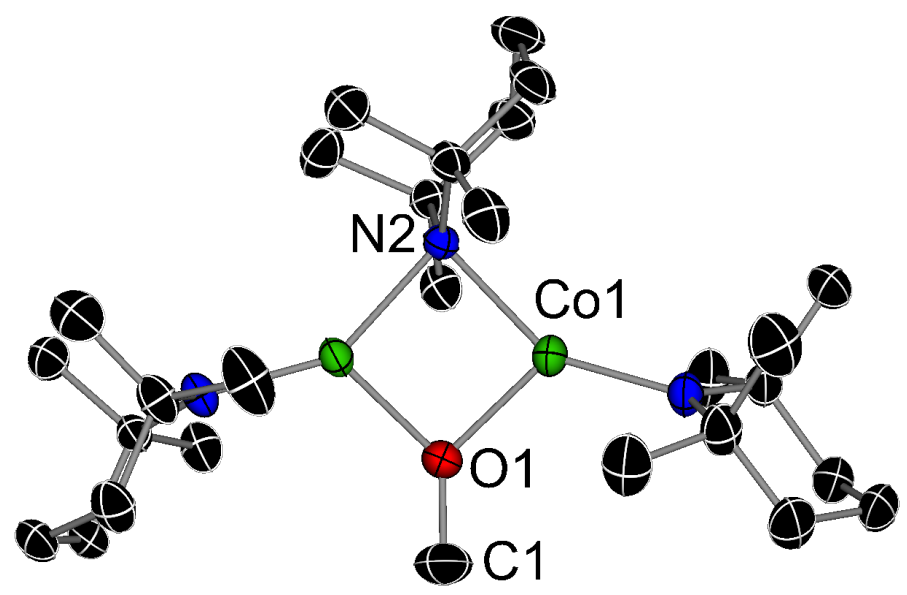

Figure S67. Molecular structure of [(MeO)Co_2(TMP)$_3$] (**4**). Hydrogen atoms omitted for clarity. Thermal ellipsoids displayed at 50% probability level.

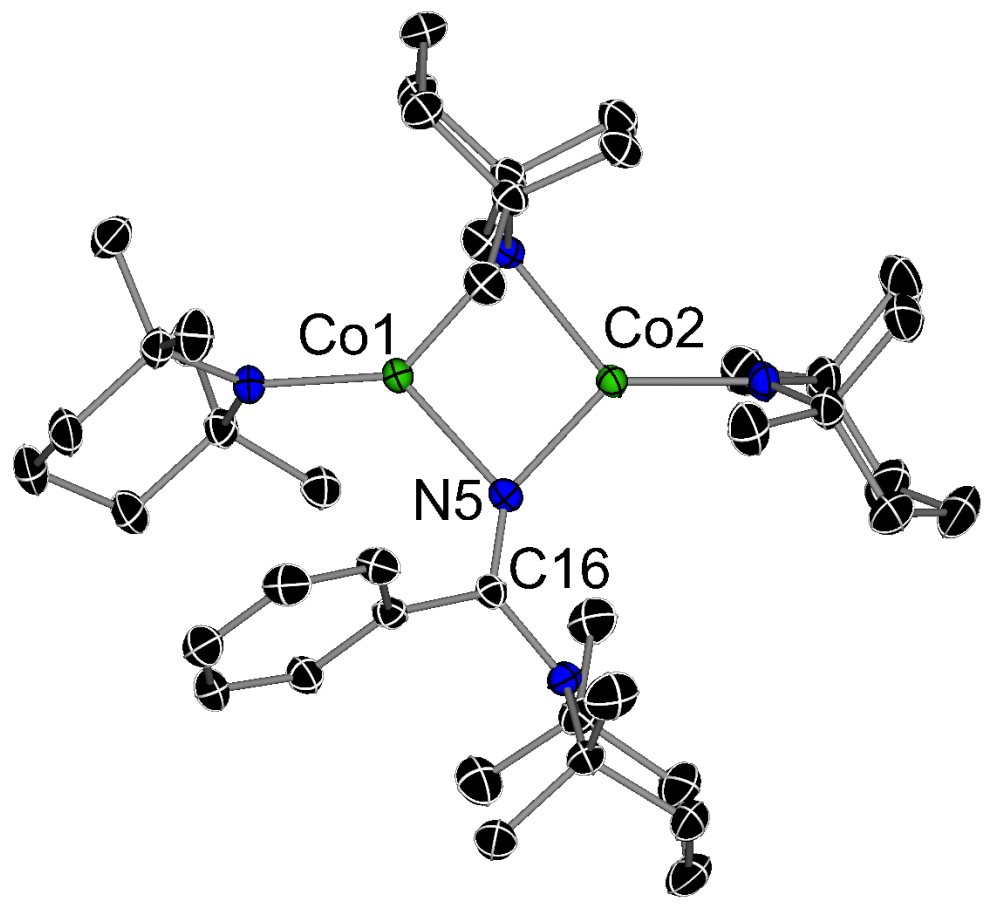

Figure S68. Molecular structure of [{(Ph)(TMP)C=N}Co_2(TMP)$_3$] (**5**). Hydrogen atoms omitted for clarity. Thermal ellipsoids displayed at 50% probability level.

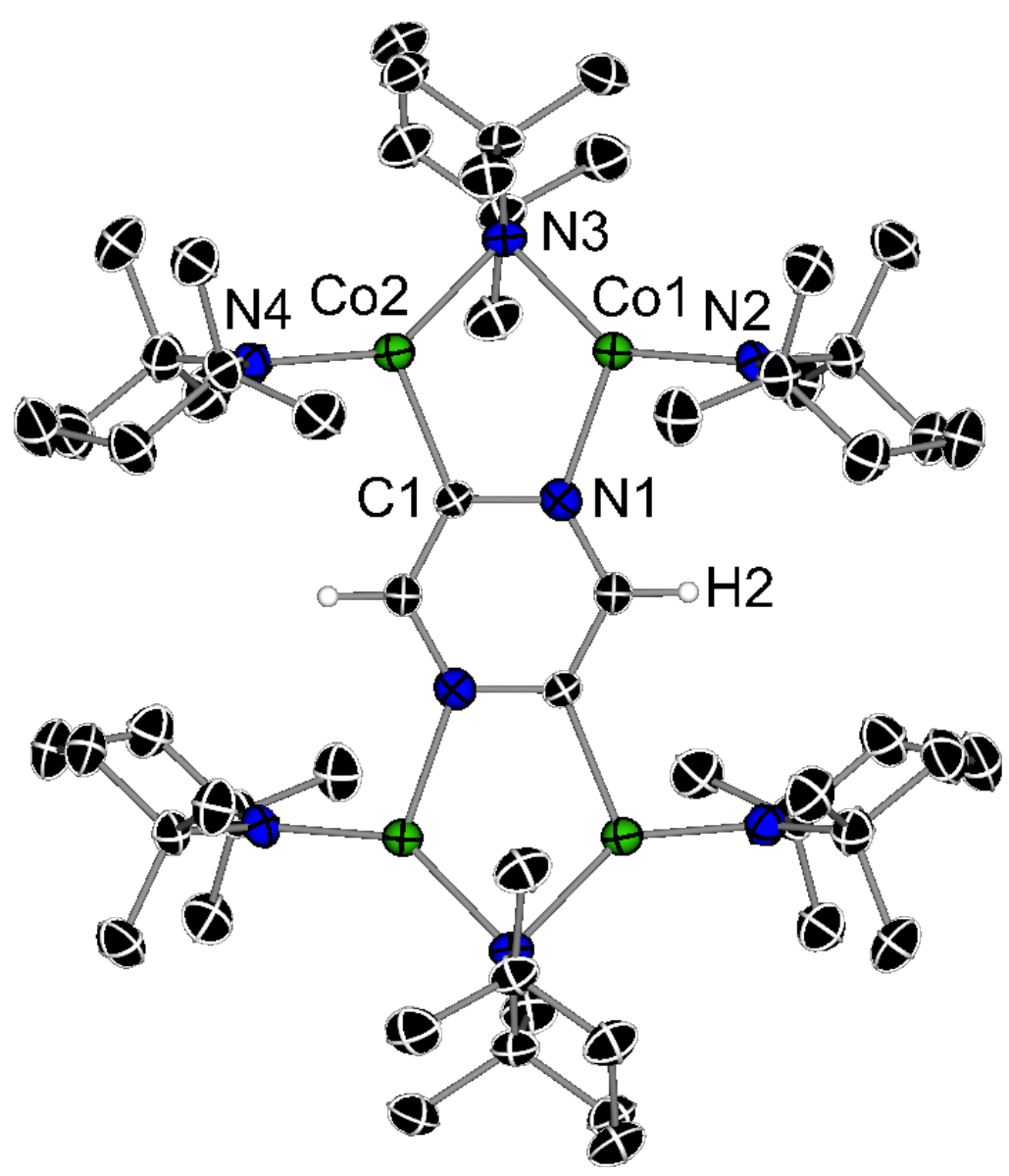

Figure S69. Molecular structure of [(2,5-$C_4H_2N_2$-pyz)Co_4(TMP)$_6$] (**6a**). Some hydrogen atoms omitted for clarity. Thermal ellipsoids displayed at 50% probability level.

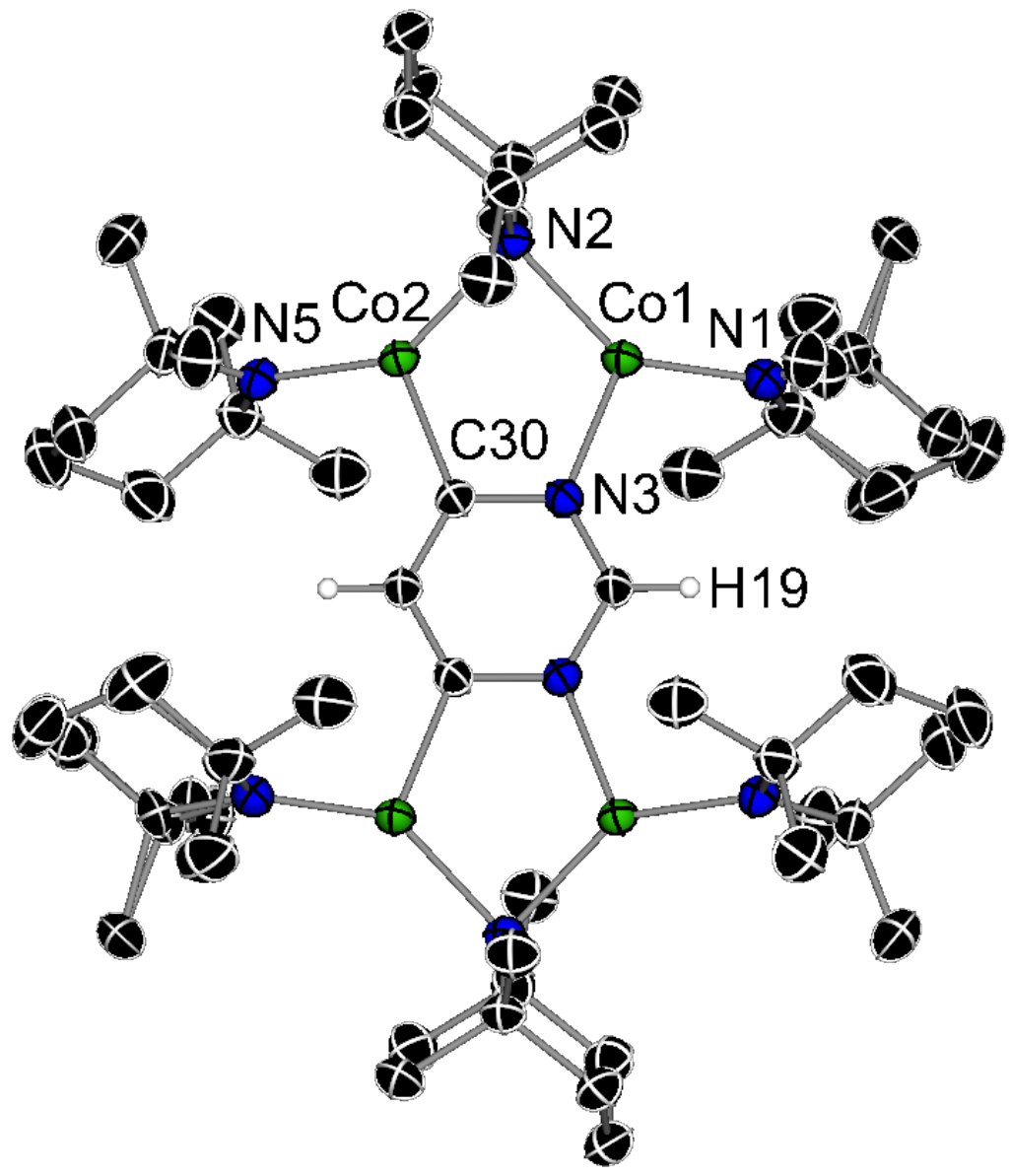

Figure S70. Molecular structure of [(4,6-$C_4H_2N_2$-pym)Co_4(TMP)$_6$] (**6b**). Some hydrogen atoms omitted for clarity. Thermal ellipsoids displayed at 50% probability level.

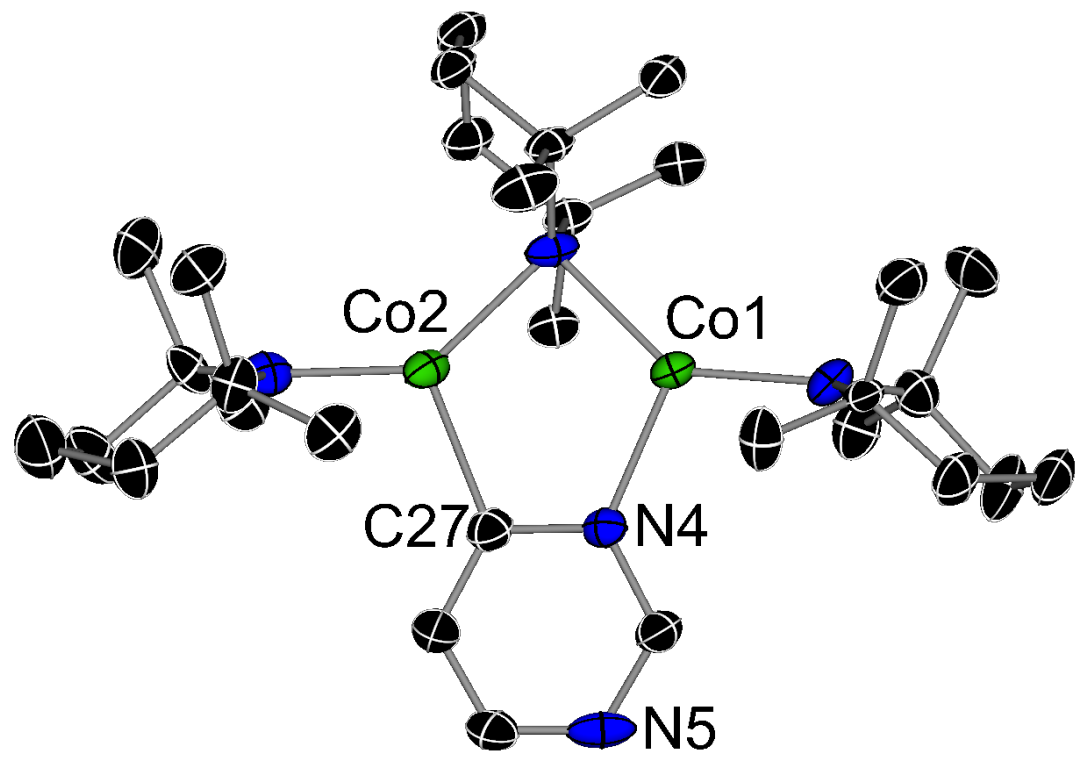

Figure S71. Molecular structure of [(4-$C_4H_3N_2$-pym)Co_2(TMP)$_3$] (**7b**). Hydrogen atoms omitted for clarity. Thermal ellipsoids displayed at 50% probability level.

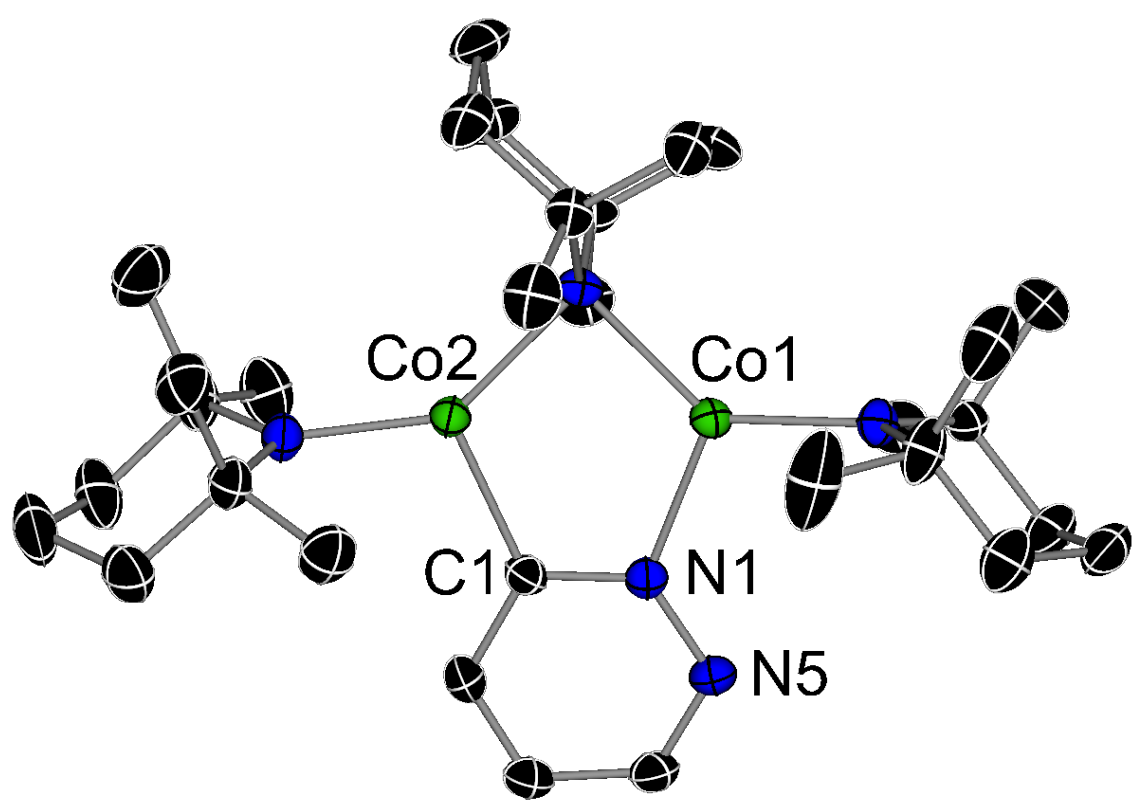

Figure S72. Molecular structure of [(3-$C_4H_3N_2$-pdz)Co_2(TMP)$_3$] (**7c**). Hydrogen atoms omitted for clarity. Thermal ellipsoids displayed at 50% probability level.

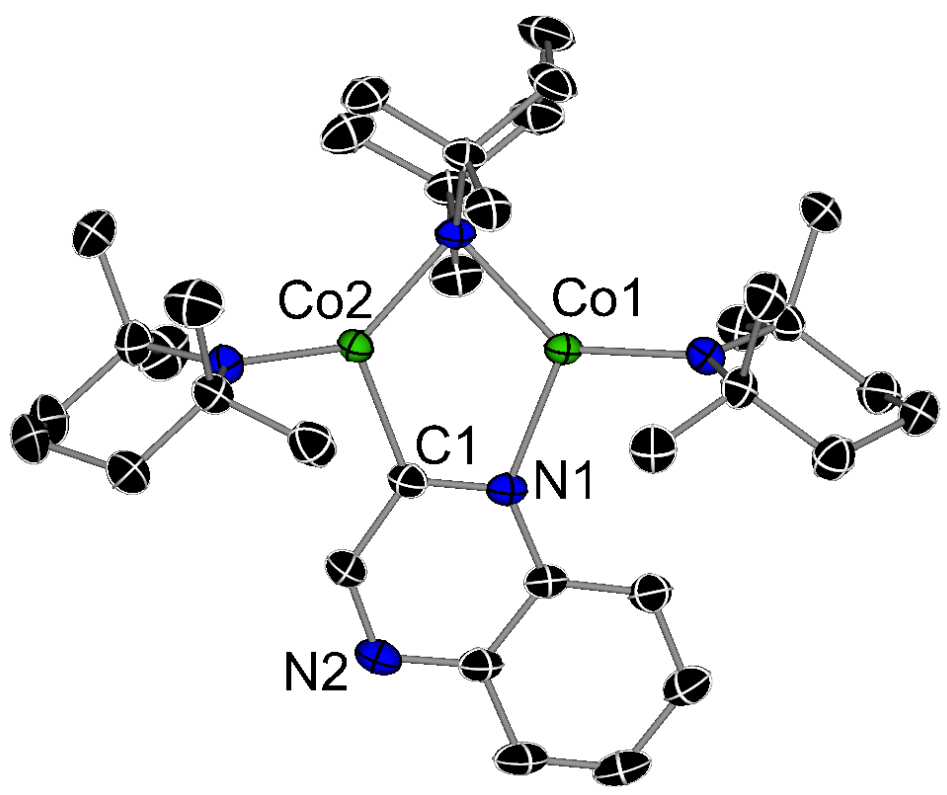

Figure S73. Molecular structure of [(2-quinoxalinyl)$Co_2(TMP)_3$] (**7d**). Hydrogen atoms omitted for clarity. Thermal ellipsoids displayed at 50% probability level.

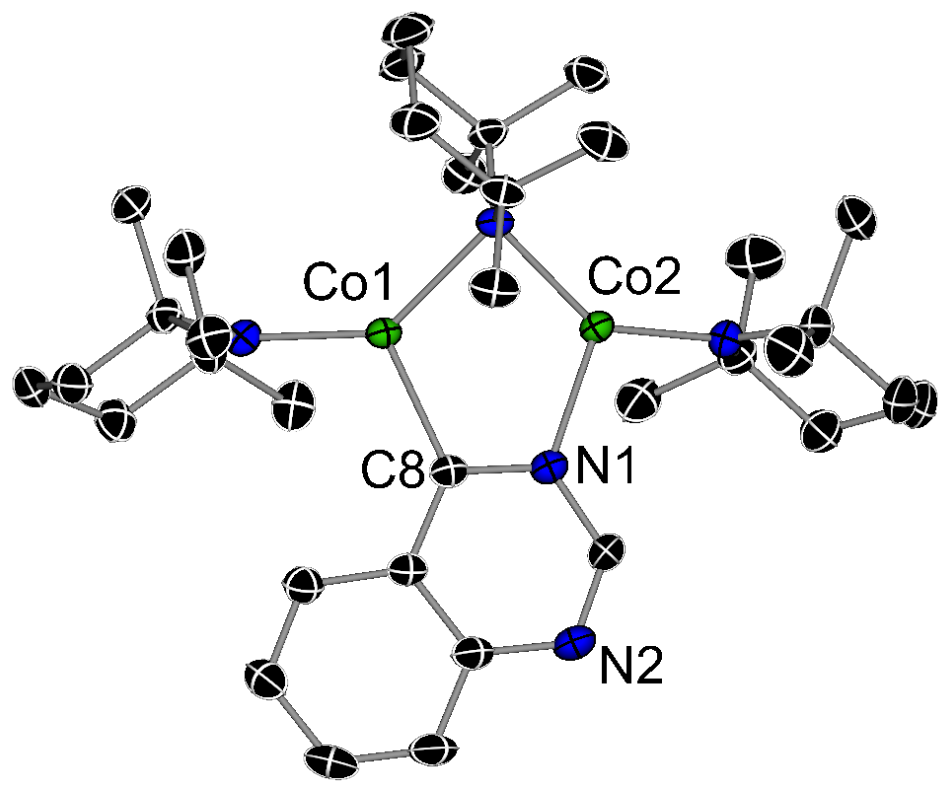

Figure S74. Molecular structure of [(4-quinazolinyl)$Co_2(TMP)_3$] (**7e**). Hydrogen atoms omitted for clarity. Thermal ellipsoids displayed at 50% probability level.

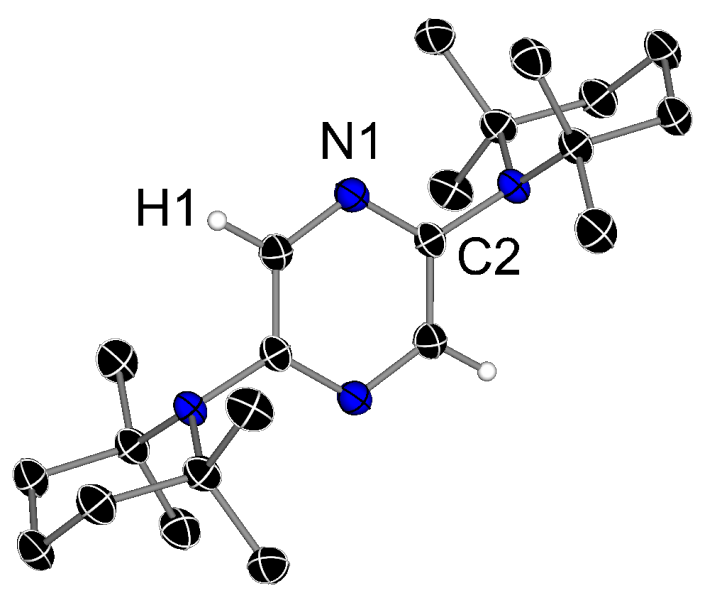

Figure S75. Molecular structure of 2,5-bis(2,2,6,6-tetramethylpiperidin-1-yl)pyrazine (**8c**). Some hydrogen atoms omitted for clarity. Thermal ellipsoids displayed at 50% probability level.

Table of Bond Parameters in 3a-d, 3f-m, 6a-b, 7b-e

Complex	Co···Co/Å	Co–N_{het}/Å	Co–C_{het}/Å	Average Co–N_{TMP} (bridging)/Å	Average Co–N_{TMP} (terminal)/Å
**3a**	2.8830(5)	2.026(2)	2.033(3)	2.0585	1.879
**3b**	2.7349(2)	2.0345(10)	2.0321(13)	2.06275	1.88165
**3c**	3.1177(4)	2.0653(8)	2.0898(10)	2.0799	1.886
**3d**	2.9237(2)	1.946(2)	2.112(3)	2.05645	1.89075
**3f**	2.87232(16)	2.0238(7)	2.0308(9)	2.0655	1.8822
**3g**	2.9282(3)	1.975(4)	2.062(5)	2.0585	1.8873
**3h**	2.9001(3)	2.0101(12)	2.0472(15)	2.065	1.88115
**3i**	2.8322(2)	2.0500(9)	2.0220(11)	2.0497	1.87855
**3j**	2.87492(18)	2.0530(8)	2.0293(10)	2.052	1.87745
**3k**	2.8716(3)	2.0204(13)	2.0349(14)	2.0581	1.87705
**3l**	3.0908(7)	2.0753(9)	2.0452(11)	2.07205	1.8931
**3m**	2.8997(2)	2.0303(10)	2.0522(11)	2.058	1.88355
**6a**	2.7778(3)	2.0083(13)	2.0145(14)	2.05155	1.87955
**6b**	2.8142(3)	2.0076(13)	2.0096(12)	2.05055	1.87735
**7b**	2.9034(6)	2.015(10)	2.044(13)	2.0555	1.879
**7c**	2.9078(5)	2.024(3)	2.024(4)	2.0515	1.886
**7d**	2.7735(3)	2.0425(11)	2.0019(14)	2.0514	1.8818
**7e**	2.8625(2)	2.0063(12)	2.0416(13)	2.05945	1.87925

X-Ray Tables

The crystal structures of compounds **2^{Fe}**, **2a**, **2f**, **3a**, **3b**, **3c**, **3d**, **3f**, **3g**, **3h**, **3i**, **3j**, **3k**, **3l**, **3m**, **4**, **5**, **6a**, **6b**, **7b**, **7c**, **7d**, **7e**, and **8c** have been deposited into the Cambridge Crystallographic Data Centre (CCDC) and have been assigned the following numbers: **2517148**, **2517149**, **2517150**, **2517151**, **2517152**, **2517153**, **2517154**, **2517155**, **2517156**, **2517157**, **2517158**, **2517159**, **2517160**, **2517161**, **2517162**, **2517163**, **2517164**, **2517165**, **2517166**, **2517167**, **2517168**, **2517169**, **2517170**, and **2517171**. Selected crystallographic and refinement parameters are presented in **Tables S1-8** below. In all cases samples immersed in inert oil were mounted at ambient conditions and transferred into the cold stream of nitrogen.

The structures of all compounds were measured at the University of Bern, Switzerland. All measurements were performed on a either a *RIGAKU Synergy S* or *Oxford Diffraction SuperNova* area-detector diffractometer using mirror optics monochromated Cu *K*α radiation (λ = 1.54184 Å) or Mo *K*α radiation (λ = 0.71073 Å), respectively. Data reduction was performed using the *CrysAlisPro* program.[8] The structure was solved by direct methods using *SHELXT*,[9] which revealed the positions of all non-hydrogen atoms of the title compound. Refinement of the structure was carried out on F^2 using full-matrix least-squares procedures. All calculations were performed using the *SHELXL-2014/7* program[10] in Olex2.[11]

Table S1. Crystal data and structure refinement parameters for compounds $\mathbf{2^{Fe}}$, **2a** and **2f**.

**Compound**	$\mathbf{2^{Fe}}$	**2a**	**2f**
CCDC Number	2517148	2517149	2517150
Identification code	24EH225_NK2-141	23EH306_NK1-140-1	23EH286_NK1-162-1
Empirical formula	$C_{23}H_{41}FeN_3$	$C_{23}H_{41}CoN_3$	$C_{24}H_{43}CoN_3O$
Formula weight	415.44	418.52	448.54
Temperature/K	173.00(10)	100.01(10)	173.00(10)
Crystal system	monoclinic	orthorhombic	monoclinic
Space group	$P2_1/c$	$Pca2_1$	C2/c
a/Å	9.8944(3)	12.66752(19)	16.6724(4)
b/Å	18.4285(5)	9.89617(17)	11.2918(3)
c/Å	12.7471(3)	18.2503(3)	13.3851(3)
α/°	90	90	90
β/°	91.472(3)	90	92.692(2)
γ/°	90	90	90
Volume/Å^3	2323.55(11)	2287.85(6)	2517.11(10)
Z	4	4	4
ρ_{calc}g/cm^3	1.188	1.215	1.184
μ/mm^{-1}	0.661	0.762	0.700
F(000)	904.0	908.0	972.0
Crystal size/mm	0.345 × 0.279 × 0.157	0.132 × 0.118 × 0.045	0.386 × 0.089 × 0.083
Radiation/Å	Mo Kα (λ = 0.71073)	Mo Kα (λ = 0.71073)	Mo Kα (λ = 0.71073)
2Θ range for data collection/°	3.886 to 61.04	4.464 to 61.016	4.358 to 61.014
Index ranges	-14 ≤ h ≤ 14, -26 ≤ k ≤ 26, -18 ≤ l ≤ 18	-18 ≤ h ≤ 18, -14 ≤ k ≤ 14, -26 ≤ l ≤ 26	-23 ≤ h ≤ 23, -16 ≤ k ≤ 16, -19 ≤ l ≤ 19
Reflections collected	13488	66381	37416
Independent reflections	13488 [R_{int} = ?, R_{sigma} = 0.0203]	6970 [R_{int} = 0.0615, R_{sigma} = 0.0361]	3839 [R_{int} = 0.0338, R_{sigma} = 0.0197]
Data/restraints/parameters	13488/126/332	6970/282/431	3839/0/173
Goodness-of-fit on F^2	1.038	1.188	1.055
Final R indexes [I>=2σ (I)]	R_1 = 0.0573, wR_2 = 0.1539	R_1 = 0.0650, wR_2 = 0.1570	R_1 = 0.0278, wR_2 = 0.0719
Final R indexes [all data]	R_1 = 0.0723, wR_2 = 0.1630	R_1 = 0.0727, wR_2 = 0.1599	R_1 = 0.0384, wR_2 = 0.0753
Largest diff. peak/hole / e Å^{-3}	1.64/-0.41	1.18/-1.34	0.33/-0.16
Flack parameter	-	0.49(5)	-

Table S2. Crystal data and structure refinement parameters for compounds **3a**, **3b** and **3c**.

**Compound**	**3a**	**3b**	**3c**
CCDC Number	2517151	2517152	2517153
Identification code	23EH272_NK1-163	24EH291_NK2-179	24EH018_NK2-62
Empirical formula	$C_{32}H_{58}Co_2N_4$	$C_{38}H_{62}Co_2N_4$	$C_{33}H_{60}Co_2N_4$
Formula weight	616.68	692.77	630.71
Temperature/K	173.01(10)	173.00(10)	173.00(10)
Crystal system	monoclinic	monoclinic	triclinic
Space group	C2/c	$P2_1/c$	P-1
a/Å	36.4310(5)	22.5942(5)	8.16977(8)
b/Å	7.88475(10)	10.82472(15)	14.06177(15)
c/Å	22.3694(3)	15.6402(3)	15.15832(17)
α/°	90	90	78.5294(9)
β/°	91.2373(13)	107.715(2)	82.1757(9)
γ/°	90	90	80.9410(9)
Volume/$Å^3$	6424.10(15)	3643.82(13)	1675.43(3)
Z	8	4	2
ρ_{calc}g/cm^3	1.275	1.263	1.250
μ/mm^{-1}	1.059	0.941	1.016
F(000)	2656.0	1488.0	680.0
Crystal size/mm	0.286 × 0.13 × 0.031	0.245 × 0.204 × 0.044	0.365 × 0.063 × 0.051
Radiation/Å	Mo Kα (λ = 0.71073)	Mo Kα (λ = 0.71073)	Mo Kα (λ = 0.71073)
2Θ range for data collection/°	4.234 to 61.016	4.212 to 61.012	4.414 to 61.016
Index ranges	-51 ≤ h ≤ 52, -11 ≤ k ≤ 11, -31 ≤ l ≤ 31	-31 ≤ h ≤ 32, -15 ≤ k ≤ 15, -22 ≤ l ≤ 22	-11 ≤ h ≤ 11, -20 ≤ k ≤ 20, -21 ≤ l ≤ 21
Reflections collected	94094	71792	73047
Independent reflections	9799 [R_{int} = 0.0406, R_{sigma} = 0.0234]	11133 [R_{int} = 0.0367, R_{sigma} = 0.0293]	10183 [R_{int} = 0.0213, R_{sigma} = 0.0139]
Data/restraints/parameters	9799/0/478	11133/0/409	10183/0/364
Goodness-of-fit on F^2	1.131	1.021	1.040
Final R indexes [I>=2σ (I)]	R_1 = 0.0545, wR_2 = 0.1340	R_1 = 0.0336, wR_2 = 0.0737	R_1 = 0.0240, wR_2 = 0.0639
Final R indexes [all data]	R_1 = 0.0672, wR_2 = 0.1394	R_1 = 0.0478, wR_2 = 0.0780	R_1 = 0.0281, wR_2 = 0.0653
Largest diff. peak/hole / e Å^{-3}	0.57/-0.47	0.37/-0.22	0.32/-0.18
Flack parameter	-	-	-

Table S3. Crystal data and structure refinement parameters for compounds **3d**, **3f** and **3g**.

**Compound**	**3d**	**3f**	**3g**
CCDC Number	2517154	2517155	2517156
Identification code	24EH206_NK2-129	23EH292_absorption	24EH295_NK2-177-2
Empirical formula	$C_{33}H_{60}Co_2N_4$	$C_{33}H_{60}Co_2N_4O$	$C_{33}H_{60}Co_2N_4O$
Formula weight	630.71	646.71	646.71
Temperature/K	173.00(10)	173.01(10)	173.00(10)
Crystal system	monoclinic	triclinic	monoclinic
Space group	C2/c	P-1	C2/c
a/Å	41.6566(4)	8.33080(10)	37.5272(5)
b/Å	7.97260(10)	15.1713(2)	8.00800(10)
c/Å	22.6434(3)	16.3532(2)	22.4314(3)
α/°	90	117.5600(10)	90
β/°	104.2510(10)	93.3010(10)	91.7010(10)
γ/°	90	92.2250(10)	90
Volume/Å^3	7288.71(15)	1824.28(4)	6738.07(15)
Z	8	2	8
ρ_{calc}g/cm^3	1.150	1.177	1.275
μ/mm^{-1}	0.935	0.937	1.015
F(000)	2720.0	696.0	2784.0
Crystal size/mm	0.218 × 0.15 × 0.05	0.565 × 0.371 × 0.316	0.344 × 0.162 × 0.115
Radiation/Å	Mo Kα (λ = 0.71073)	Mo Kα (λ = 0.71073)	Mo Kα (λ = 0.71073)
2Θ range for data collection/°	4.036 to 61.016	4.912 to 61.01	4.178 to 61.016
Index ranges	-59 ≤ h ≤ 59, -11 ≤ k ≤ 11, -32 ≤ l ≤ 31	-11 ≤ h ≤ 11, -21 ≤ k ≤ 21, -23 ≤ l ≤ 23	-53 ≤ h ≤ 53, -11 ≤ k ≤ 11, -32 ≤ l ≤ 32
Reflections collected	96977	111129	90742
Independent reflections	11147 [R_{int} = 0.0202, R_{sigma} = 0.0124]	11134 [R_{int} = 0.0285, R_{sigma} = 0.0141]	10288 [R_{int} = 0.0289, R_{sigma} = 0.0180]
Data/restraints/parameters	11147/57/430	11134/0/374	10288/136/478
Goodness-of-fit on F^2	1.070	1.065	1.039
Final R indexes [I>=2σ (I)]	R_1 = 0.0300, wR_2 = 0.0802	R_1 = 0.0226, wR_2 = 0.0628	R_1 = 0.0333, wR_2 = 0.0847
Final R indexes [all data]	R_1 = 0.0380, wR_2 = 0.0837	R_1 = 0.0258, wR_2 = 0.0640	R_1 = 0.0467, wR_2 = 0.0905
Largest diff. peak/hole / e Å^{-3}	0.34/-0.41	0.33/-0.20	0.68/-0.26
Flack parameter	-	-	-

Table S4. Crystal data and structure refinement parameters for compounds **3h**, **3i** and **3j**.

**Compound**	**3h**	**3i**	**3j**
CCDC Number	2517157	2517158	2517159
Identification code	24EH308_NK2-45	23EH297_NK2-30	23EH335_NK2-39
Empirical formula	$C_{33}H_{60}Co_2N_4O$	$C_{32}H_{57}Co_2FN_4$	$C_{32}H_{56}Co_2F_2N_4$
Formula weight	646.71	634.67	652.66
Temperature/K	173.00(10)	173.01(10)	173.00(10)
Crystal system	monoclinic	monoclinic	monoclinic
Space group	$P2_1/c$	C2/c	C2/c
a/Å	7.97123(13)	36.2391(4)	35.9355(3)
b/Å	26.6753(5)	7.95977(9)	7.95695(6)
c/Å	15.6947(3)	22.5564(2)	22.97717(17)
α/°	90	90	90
β/°	94.8090(16)	91.7334(10)	91.6313(6)
γ/°	90	90	90
Volume/Å^3	3325.49(10)	6503.54(13)	6567.36(9)
Z	4	8	8
ρ_{calc}g/cm^3	1.292	1.296	1.320
μ/mm^{-1}	1.028	1.052	1.048
F(000)	1392.0	2720.0	2784.0
Crystal size/mm	0.111 × 0.094 × 0.015	0.371 × 0.31 × 0.048	0.315 × 0.14 × 0.059
Radiation/Å	Mo Kα (λ = 0.71073)	Mo Kα (λ = 0.71073)	Mo Kα (λ = 0.71073)
2Θ range for data collection/°	4.014 to 61.016	4.198 to 61.016	4.156 to 61.016
Index ranges	-11 ≤ h ≤ 11, -38 ≤ k ≤ 38, -22 ≤ l ≤ 22	-51 ≤ h ≤ 51, -11 ≤ k ≤ 11, -32 ≤ l ≤ 32	-51 ≤ h ≤ 51, -11 ≤ k ≤ 11, -32 ≤ l ≤ 32
Reflections collected	102603	94318	192322
Independent reflections	10159 [R_{int} = 0.0471, R_{sigma} = 0.0294]	9939 [R_{int} = 0.0309, R_{sigma} = 0.0173]	10048 [R_{int} = 0.0277, R_{sigma} = 0.0098]
Data/restraints/parameters	10159/0/374	9939/6/391	10048/74/422
Goodness-of-fit on F^2	1.010	1.047	1.034
Final R indexes [I>=2σ (I)]	R_1 = 0.0343, wR_2 = 0.0706	R_1 = 0.0259, wR_2 = 0.0648	R_1 = 0.0238, wR_2 = 0.0644
Final R indexes [all data]	R_1 = 0.0523, wR_2 = 0.0761	R_1 = 0.0319, wR_2 = 0.0669	R_1 = 0.0268, wR_2 = 0.0658
Largest diff. peak/hole / e Å^{-3}	0.41/-0.38	0.32/-0.23	0.33/-0.22
Flack parameter	-	-	-

Table S5. Crystal data and structure refinement parameters for compounds **3k**, **3l** and **3m**.

**Compound**	**3k**	**3l**	**3m**
CCDC Number	2517160	2517161	2517162
Identification code	23EH346_NK1-163	24EH130_NK2-93	24EH020_NK2-41
Empirical formula	$C_{32}H_{57}Co_2FN_4$	$C_{35}H_{63}Co_2N_7O_2$	$C_{40}H_{64}Co_2N_4$
Formula weight	634.67	731.78	718.81
Temperature/K	173.00(10)	213.00(10)	173.00(10)
Crystal system	monoclinic	monoclinic	triclinic
Space group	C2/c	$P2_1/c$	P-1
a/Å	36.5443(5)	11.74520(15)	12.16004(12)
b/Å	7.94637(11)	39.1987(5)	12.66722(15)
c/Å	22.5535(3)	8.09048(11)	14.33445(16)
α/°	90	90	78.6597(10)
β/°	91.6029(12)	100.4193(12)	74.6306(9)
γ/°	90	90	64.5319(11)
Volume/Å^3	6546.85(15)	3663.41(8)	1913.45(4)
Z	8	4	2
ρ_{calc}g/cm^3	1.288	1.327	1.248
μ/mm^{-1}	1.045	0.946	0.899
F(000)	2720.0	1568.0	772.0
Crystal size/mm	0.35 × 0.11 × 0.052	0.679 × 0.088 × 0.08	0.159 × 0.116 × 0.082
Radiation/Å	Mo Kα (λ = 0.71073)	Mo Kα (λ = 0.71073)	Mo Kα (λ = 0.71073)
2Θ range for data collection/°	4.192 to 61.016	4.092 to 61.014	4.302 to 61.016
Index ranges	-52 ≤ h ≤ 52, -11 ≤ k ≤ 11, -32 ≤ l ≤ 32	-16 ≤ h ≤ 16, -55 ≤ k ≤ 55, -11 ≤ l ≤ 11	-17 ≤ h ≤ 17, -18 ≤ k ≤ 18, -20 ≤ l ≤ 20
Reflections collected	96512	112335	59922
Independent reflections	10002 [R_{int} = 0.0414, R_{sigma} = 0.0246]	11176 [R_{int} = 0.0223, R_{sigma} = 0.0115]	11422 [R_{int} = 0.0343, R_{sigma} = 0.0244]
Data/restraints/parameters	10002/30/404	11176/0/430	11422/0/428
Goodness-of-fit on F^2	1.028	1.030	1.041
Final R indexes [I>=2σ (I)]	R_1 = 0.0352, wR_2 = 0.0734	R_1 = 0.0259, wR_2 = 0.0691	R_1 = 0.0312, wR_2 = 0.0814
Final R indexes [all data]	R_1 = 0.0501, wR_2 = 0.0783	R_1 = 0.0301, wR_2 = 0.0710	R_1 = 0.0414, wR_2 = 0.0850
Largest diff. peak/hole / e Å^{-3}	0.31/-0.17	0.38/-0.23	0.41/-0.21
Flack parameter	-	-	-

Table S6. Crystal data and structure refinement parameters for compounds **4**, **5** and **6a**.

**Compound**	**4**	**5**	**6a**
CCDC Number	2517163	2517164	2517165
Identification code	24EH098_NK2-69	23EH058_NK1-63	23EH214_NK1-130
Empirical formula	$C_{28}H_{57}N_3OCo_2$	$C_{43}H_{77}N_5Co_2$	$C_{58}H_{110}Co_4N_8$
Formula weight	569.651	781.95	1155.25
Temperature/K	173.00(10)	173.00(10)	173.00(10)
Crystal system	triclinic	triclinic	monoclinic
Space group	P-1	P-1	$P2_1/n$
a/Å	7.96209(10)	8.29710(10)	12.8351(2)
b/Å	14.21182(18)	11.46010(10)	22.3875(3)
c/Å	14.75136(19)	23.2613(2)	12.9797(2)
α/°	69.3496(12)	78.5760(10)	90
β/°	88.4931(11)	84.9450(10)	113.580(2)
γ/°	83.6444(10)	73.4030(10)	90
Volume/Å^3	1552.21(4)	2076.50(4)	3418.23(11)
Z	2	2	2
ρ_{calc}g/cm^3	1.219	1.251	1.122
μ/mm^{-1}	1.093	0.834	0.991
F(000)	616	848.0	1244.0
Crystal size/mm	0.321 × 0.105 × 0.036	0.324 × 0.113 × 0.059	0.183 × 0.121 × 0.071
Radiation/Å	Mo Kα (λ = 0.71073)	Mo Kα (λ = 0.71073)	Mo Kα (λ = 0.71073)
2Θ range for data collection/°	4.96 to 61.02	6.986 to 66.282	4.188 to 61.014
Index ranges	-11 ≤ h ≤ 11, -20 ≤ k ≤ 20, -21 ≤ l ≤ 21	-12 ≤ h ≤ 12, -17 ≤ k ≤ 17, -35 ≤ l ≤ 35	-18 ≤ h ≤ 18, -31 ≤ k ≤ 31, -18 ≤ l ≤ 18
Reflections collected	92560	172393	102914
Independent reflections	9446 [R_{int} = 0.0303, R_{sigma} = 0.0136]	15832 [R_{int} = 0.0335, R_{sigma} = 0.0175]	10426 [R_{int} = 0.0404, R_{sigma} = 0.0255]
Data/restraints/parameters	9446/0/381	15832/0/467	10426/0/328
Goodness-of-fit on F^2	1.033	1.048	1.044
Final R indexes [I>=2σ (I)]	R_1 = 0.0245, wR_2 = 0.0669	R_1 = 0.0268, wR_2 = 0.0717	R_1 = 0.0363, wR_2 = 0.0950
Final R indexes [all data]	R_1 = 0.0287, wR_2 = 0.0687	R_1 = 0.0332, wR_2 = 0.0741	R_1 = 0.0508, wR_2 = 0.0999
Largest diff. peak/hole / e Å^{-3}	0.38/-0.21	0.54/-0.23	0.57/-0.33
Flack parameter	-	-	-

Table S7. Crystal data and structure refinement parameters for compounds **6b**, **7b** and **7c**.

**Compound**	**6b**	**7b**	**7c**
CCDC Number	2517166	2517167	2517168
Identification code	23EH296_NK2-19	23EH197_NK1-128	24EH226_NK1-133
Empirical formula	$C_{58}H_{110}Co_4N_8$	$C_{31}H_{57}Co_2N_5$	$C_{31}H_{57}Co_2N_5$
Formula weight	1155.25	617.67	617.67
Temperature/K	173.01(10)	100.01(10)	173.00(10)
Crystal system	monoclinic	monoclinic	triclinic
Space group	I2/a	P2/n	P-1
a/Å	29.4792(7)	21.2016(4)	7.89416(10)
b/Å	8.25670(15)	7.86280(10)	14.6022(4)
c/Å	29.0255(7)	21.3272(4)	14.9200(4)
α/°	90	90	87.544(2)
β/°	118.493(3)	117.035(2)	79.9164(19)
γ/°	90	90	74.6577(19)
Volume/Å^3	6209.1(3)	3166.83(11)	1632.93(7)
Z	4	4	2
ρ_{calc}g/cm^3	1.236	1.296	1.256
μ/mm^{-1}	1.091	1.075	1.042
F(000)	2488.0	1328.0	664.0
Crystal size/mm	0.783 × 0.066 × 0.028	0.223 × 0.197 × 0.05	0.185 × 0.056 × 0.028
Radiation/Å	Mo Kα (λ = 0.71073)	Mo Kα (λ = 0.71073)	Mo Kα (λ = 0.71073)
2Θ range for data collection/°	5.178 to 61.016	4.288 to 61.012	4 to 67.304
Index ranges	-42 ≤ h ≤ 42, -11 ≤ k ≤ 11, -41 ≤ l ≤ 41	-30 ≤ h ≤ 30, -11 ≤ k ≤ 11, -30 ≤ l ≤ 30	-12 ≤ h ≤ 12, -22 ≤ k ≤ 22, -22 ≤ l ≤ 22
Reflections collected	100467	89540	18862
Independent reflections	9477 [R_{int} = 0.0399, R_{sigma} = 0.0214]	9666 [R_{int} = 0.0484, R_{sigma} = 0.0265]	18862 [R_{int} = ?, R_{sigma} = 0.0563]
Data/restraints/parameters	9477/129/374	9666/177/604	18862/0/356
Goodness-of-fit on F^2	1.023	1.095	1.068
Final R indexes [I>=2σ (I)]	R_1 = 0.0354, wR_2 = 0.0864	R_1 = 0.0621, wR_2 = 0.1414	R_1 = 0.0613, wR_2 = 0.1520
Final R indexes [all data]	R_1 = 0.0492, wR_2 = 0.0918	R_1 = 0.0726, wR_2 = 0.1450	R_1 = 0.1009, wR_2 = 0.1663
Largest diff. peak/hole / e Å^{-3}	0.35/-0.19	0.62/-0.79	1.10/-0.46
Flack parameter	-	-	-

Table S8. Crystal data and structure refinement parameters for compounds **7d**, **7e** and **8c**.

**Compound**	**7d**	**7e**	**8c**
CCDC Number	2517169	2517170	2517171
Identification code	25EH050_NK2-155	23EH210_absorption	25EH155_NK3-66
Empirical formula	$C_{35}H_{59}Co_2N_5$	$C_{35}H_{59}N_5Co_2$	$C_{22}H_{38}N_4$
Formula weight	667.73	667.73	358.56
Temperature/K	173.00(10)	173.15	173.00(10)
Crystal system	triclinic	triclinic	triclinic
Space group	P-1	P-1	P-1
a/Å	11.32288(17)	11.3856(2)	6.6090(4)
b/Å	11.80685(17)	11.8235(2)	7.7504(3)
c/Å	14.3368(2)	14.3516(2)	11.6924(7)
α/°	96.0300(12)	96.4600(10)	72.008(5)
β/°	105.8020(14)	105.7640(10)	85.515(4)
γ/°	108.2066(13)	108.601(2)	65.876(5)
Volume/Å^3	1713.57(5)	1719.66(5)	519.08(5)
Z	2	2	1
ρ_{calc}g/cm^3	1.294	1.290	1.147
μ/mm^{-1}	0.999	0.995	0.519
F(000)	716.0	716.0	198.0
Crystal size/mm	0.12 × 0.101 × 0.03	0.152 × 0.124 × 0.089	0.445 × 0.243 × 0.196
Radiation/Å	Mo Kα (λ = 0.71073)	Mo Kα (λ = 0.71073)	Cu Kα (λ = 1.54184)
2Θ range for data collection/°	3.712 to 61.016	4.148 to 61.014	7.962 to 148.994
Index ranges	-16 ≤ h ≤ 16, -16 ≤ k ≤ 16, -20 ≤ l ≤ 20	-16 ≤ h ≤ 16, -16 ≤ k ≤ 16, -20 ≤ l ≤ 20	-8 ≤ h ≤ 8, -9 ≤ k ≤ 9, -14 ≤ l ≤ 14
Reflections collected	87984	49152	18331
Independent reflections	10450 [R_{int} = 0.0333, R_{sigma} = 0.0224]	10503 [R_{int} = 0.0312, R_{sigma} = 0.0310]	2085 [R_{int} = 0.0434, R_{sigma} = 0.0368]
Data/restraints/parameters	10450/0/391	10503/0/615	2085/0/194
Goodness-of-fit on F^2	1.032	1.032	1.135
Final R indexes [I>=2σ (I)]	R_1 = 0.0322, wR_2 = 0.0829	R_1 = 0.0318, wR_2 = 0.0700	R_1 = 0.0482, wR_2 = 0.1307
Final R indexes [all data]	R_1 = 0.0437, wR_2 = 0.0870	R_1 = 0.0468, wR_2 = 0.0745	R_1 = 0.0507, wR_2 = 0.1322
Largest diff. peak/hole / e Å^{-3}	0.47/-0.26	0.37/-0.25	0.32/-0.25
Flack parameter	-	-	-

Computational Methods

The computational mechanistic study for the C–H metalation of N-heteroarenes, pyridine and pyrazine with $Co(TMP)_2$ ($\mathbf{1}^{\mathbf{Co}}$) and $Fe(TMP)_2$ (TMP = 2,2,6,6-tetramethylpiperidide) were carried out using density functional theory (DFT) calculations with the dispersion-corrected hybrid exchange-correlation functional ωB97X-D,[12] as implemented in the Gaussian16 software.[13]

Cobalt and iron metals were described with the Lanl2dz effective core potential and its associated double-zeta basis set, along with a *f*-polarization function (exponents = 2.780 and 2.462, respectively),[14] while C and H atoms were modelled with the double-zeta 6-31g(d, p) basis sets, and 6-31+g(d, p) for the more electronegative N atoms. Geometry optimizations were performed in vacuum without imposing any symmetry constraints. In all the calculations, according to experimental magnetic data presented in this work, Co and Fe metals were modelled as high-spin in the +2 oxidation state. For the dinuclear complexes, both ferro- and antiferromagnetically coupled spin states were considered through broken-symmetry DFT calculations. The nature of the stationary points was confirmed by vibrational frequency analysis, with energy minima displaying only real frequencies while transition states exhibited one imaginary frequency corresponding to the desired reaction coordinate. Additionally, the optimized transition states were relaxed in both directions along the reaction coordinate to confirm that they connect the expected energy minima. The effect of the solvent used in experiments (benzene, $\varepsilon = 2.2706$) was accounted for through single-point calculations with the SMD continuum solvation model.[15]

Non-covalent interaction and QTAIM analyses were carried out using the Critic2 software.[16–18] For further details of the application of these analyses on similar systems, we refer the reader to our previous work.[1] The 3D electron density difference plot was generated and inspected with GaussView 6.0 software.

All the DFT data underlying this work, including the cartesian coordinates and energies of all the modelled structures, is openly accessible via the following ioChem-BD online dataset:
https://iochem-bd.bsc.es/browse/review-collection/100/485245/153cfbf05c1b6d3ebfa73f1e

Transition-state prediction of experimentally observed selectivity

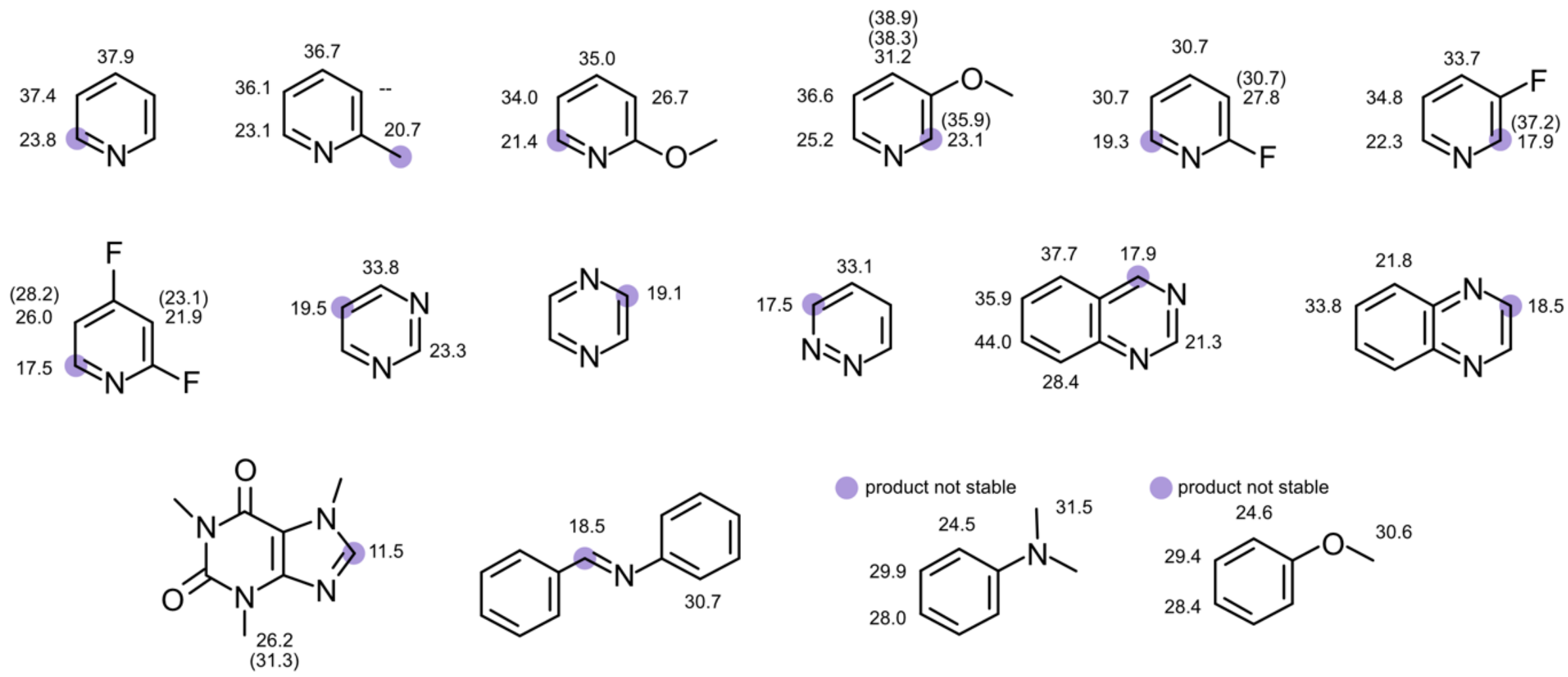

Figure S76. Gibbs energies (in kcal mol^{-1}) of the transition states computed under experimental conditions (benzene, rt), referenced to the lowest-energy intermediate preceding each transition state. For substrates containing more than one sp^2 nitrogen, non-sp^2 nitrogen atoms, as well as F and O atoms, all possible coordination modes were considered when determining the lowest-energy intermediate. The Gibbs energy barrier corresponding to the experimentally observed C–H activation site is highlighted with a purple circle.

Speciation study of di-nuclear Co and Fe complexes

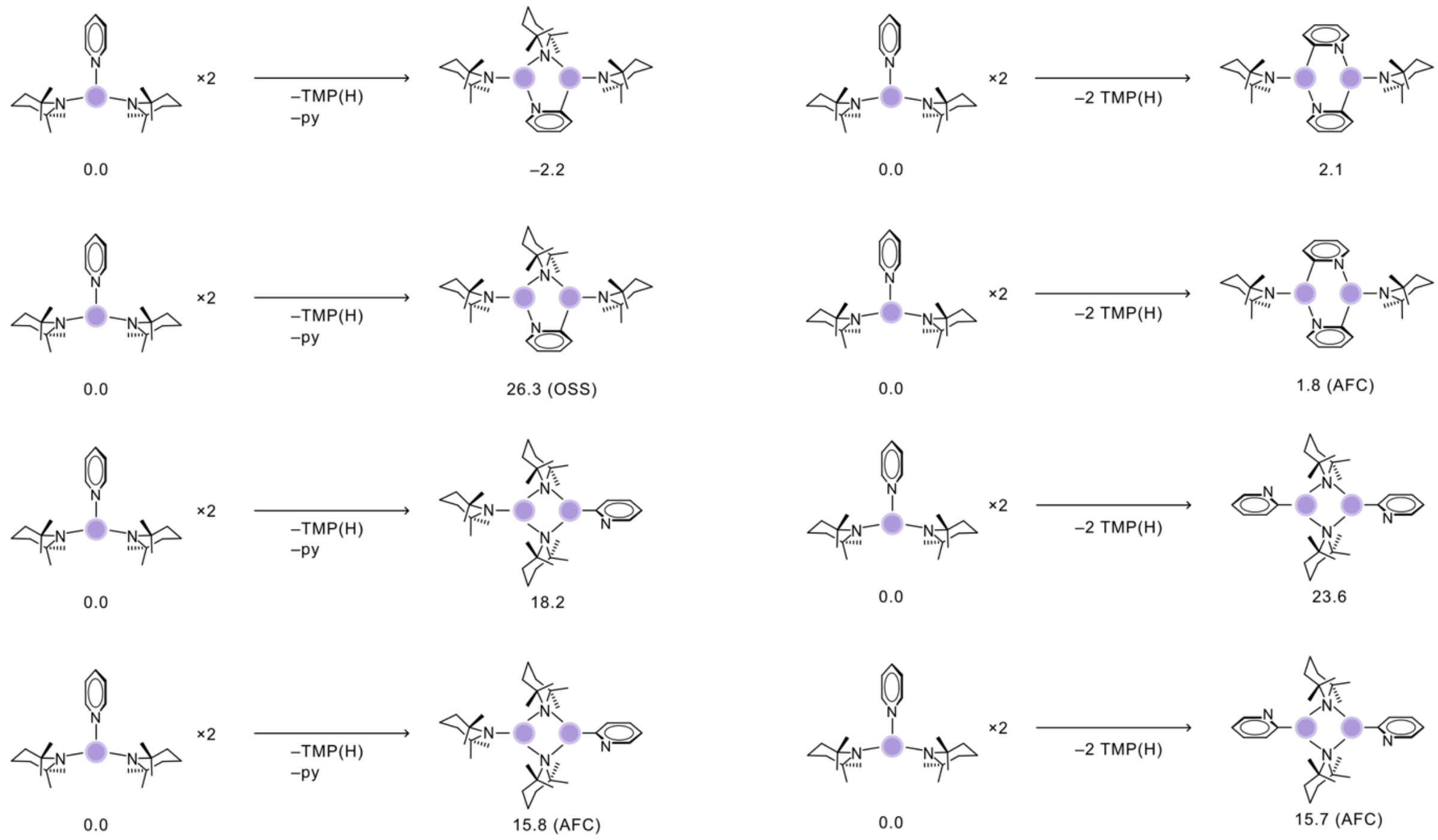

Figure S77. Speciation study under experimental conditions (benzene, rt). Gibbs energies are reported in kcal mol^{-1} and are referenced to the most stable reactants for the reaction of **2**$^{\mathbf{Co}}$ with pyridine. AFC denotes antiferromagnetically coupled, and OSS denotes open-shell singlet.

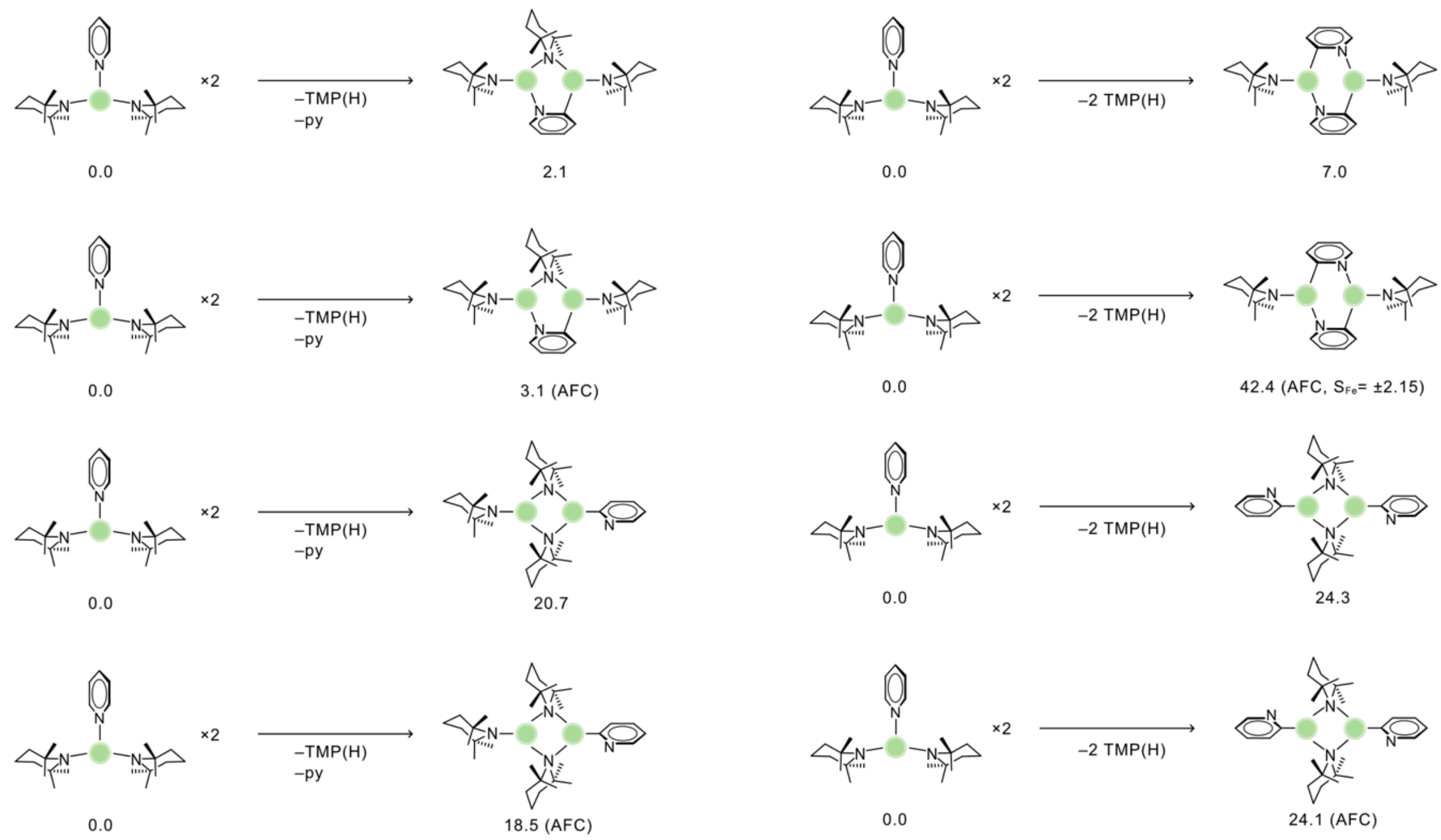

Figure S78. Speciation study under experimental conditions (benzene, rt). Gibbs energies are reported in kcal mol^{-1} and are referenced to the most stable reactants for the reaction of **2**$^{\mathbf{Fe}}$ with pyridine. AFC denotes antiferromagnetically coupled, and OSS denotes open-shell singlet.

C–H metalation of pyridine with $Fe(TMP)_2$

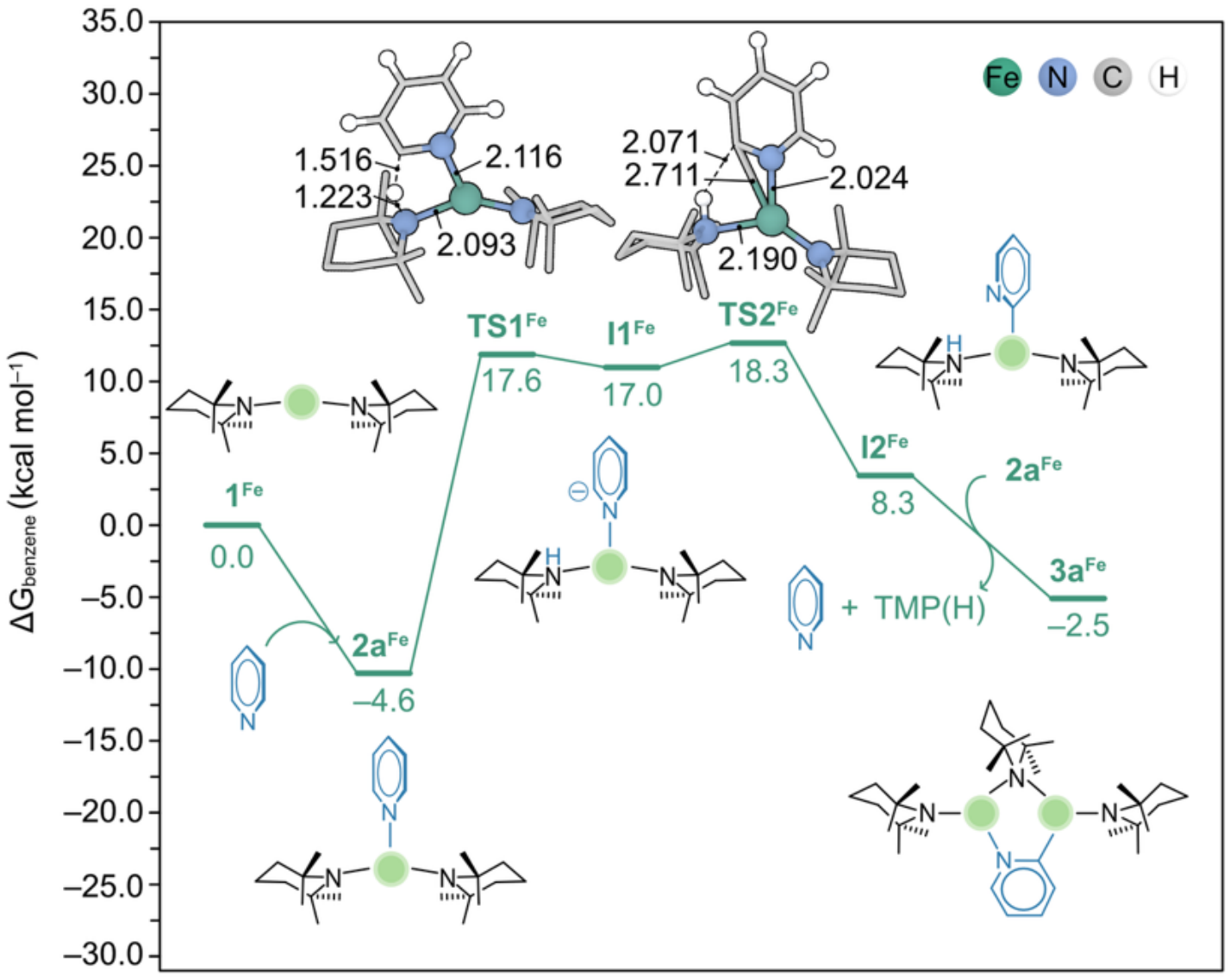

Figure S79. Gibbs energy profile for the C–H metalation of pyridine by $Fe(TMP)_2$ at 298 K and 1 atm. Optimized structures of $\mathbf{TS1^{Fe}}$ and $\mathbf{TS2^{Fe}}$, with relevant bond lengths (Å), are shown as insets; some H atoms are omitted for clarity.

Calculated C–H acidity of free and $Co(TMP)_2$-coordinated pyridine

The pK_a values for uncoordinated pyridine and the $Co(TMP)_2$-bound complex (**2a**) were computed and are summarized in Table S9. Coordination to $Co(TMP)_2$ leads to a uniform decrease in pK_a values across all C–H positions, while preserving the intrinsic acidity trend (*ortho* > *meta* > *para*), with the *ortho* position remaining the least acidic in both free pyridine and **2a**.

This overall increase in acidity is attributed to a decrease in electron density on the pyridine ring upon coordination of the nitrogen lone pair to the cobalt centre, consistent with the donor–acceptor interactions identified by NBO analysis (Figure 3d). Notably, this coordination perturbs the ring electronically in a largely uniform manner, in contrast to substituent effects at specific carbon positions (e.g., 2-methoxypyridine).

While the absolute pK_a values reflect the thermodynamics of proton dissociation, site selectivity in this C–H cobaltation is governed by kinetic factors. This is supported by the computed transition state barriers (Figure S76), which show a clear preference for activation at the *ortho* position ($\Delta G^{\ddagger}$ = 23.8 kcal mol^{-1}) compared to the *meta* (37.4 kcal mol^{-1}) and *para* (27.9 kcal mol^{-1}) positions.

Table S9. Computed pK_a values for *ortho*-, *meta*-, and *para*-C–H positions of pyridine in the free and $Co(TMP)_2$-coordinated forms (**2a**) in DMSO. Experimental values for free pyridine are provided for comparison.

**Molecule**	**pK_a values at different positions**		
	ortho-	*meta-*	*para-*
Pyridine (*experiment*)	43.5	41.0	40.3
Pyridine (*DFT*)	41.9	40.7	40.3
(pyridine)$Co(TMP)_2$, **2a** (*DFT*)	30.1	27.3	26.8

The pK_a values were derived from computed Gibbs energies for proton dissociation reactions (RH → R^- + H^+), where RH corresponds to pyridine or **2a** and R^- denotes the conjugate bases. The absolute Gibbs energy of H^+ in DMSO was estimated by fitting the computed pK_a of the *para* position of pyridine to the experimental value (pK_a = 40.3) using $\Delta G = 2.303RT\cdot pK_a$. From the calculated RH → R^- energy of 340.5 kcal mol^{-1}, the absolute Gibbs energy of H^+ in DMSO was determined to be 283.8 kcal mol^{-1}. This reference value was subsequently used to determine pK_a values for all other positions.

Geometry optimisations were carried out at the ωB97X-D/6-31+G(d,p)/LANL2DZ+f level of theory, and energies were refined through single-point calculations including SMD implicit solvation (ε = 2.2706) with a triple-ζ basis set (6-311+G(d,p)/LANL2TZ+f). Standard-state corrections were applied to all Gibbs energies, and pK_a values were obtained using $\Delta G = 2.303RT \cdot pK_a$.

References

(1) Logallo, A.; Maddock, L. C. H.; Mu, M.; Gravogl, L.; Jin, N.; Peñas-Defrutos, M. N.; Meyer, K.; García-Melchor, M.; Hevia, E. Unlocking the Metalation Applications of TMP-Powered Fe and Co(II) Bis(Amides): Synthesis, Structure and Mechanistic Insights. *Angew. Chem., Int. Ed.* **2024**, *63* (24), e202402907.

(2) Pangborn, A. B.; Giardello, M. A.; Grubbs, R. H.; Rosen, R. K.; Timmers, F. J. Safe and Convenient Procedure for Solvent Purification. *Organometallics* **1996**, *15* (5), 1518–1520.

(3) Evans, D. F. 400. The Determination of the Paramagnetic Susceptibility of Substances in Solution by Nuclear Magnetic Resonance. *J. Chem. Soc.* **1959**, *81* (0), 2003–2005.

(4) Schubert, E. M. Utilizing the Evans Method with a Superconducting NMR Spectrometer in the Undergraduate Laboratory. *J. Chem. Educ.* **1992**, *69* (1), 62.

(5) Piguet, C. Paramagnetic Susceptibility by NMR: The "Solvent Correction" Removed for Large Paramagnetic Molecules. *J. Chem. Educ.* **1997**, *74* (7), 815–816.

(6) Slesarchuk, N.; Ma, E.; Miranda-Pizarro, J.; Heikkinen, S.; Schollmeyer, D.; Nieger, M.; Vasko, P.; Repo, T. On the Mechanism of sp^2 C–H Borylation Using *Ortho-N*-Substituted Pyridinium Cations. *Dalton Trans.* **2024**, *53* (22), 9590–9595.

(7) Keaveney, S. T.; Kundu, G.; Schoenebeck, F. Modular Functionalization of Arenes in a Triply Selective Sequence: Rapid C(sp^2) and C(sp^3) Coupling of C–Br, C–OTf, and C–Cl Bonds Enabled by a Single Palladium(I) Dimer. *Angew. Chem., Int. Ed.* **2018**, *57* (38), 12573–12577.

(8) *CrysAlis PRO,* Oxford Diffraction Ltd, Yarnton, England, **2009**.

(9) Sheldrick, G. M. SHELXT - Integrated Space-Group and Crystal-Structure Determination. *Acta Crystallogr. Sect. A* **2015**, *71* (1), 3–8.

(10) Sheldrick, G. M. Crystal Structure Refinement with SHELXL. *Acta Crystallogr. Sect. C* **2015**, *71* (1), 3–8.

(11) Dolomanov, O. V.; Bourhis, L. J.; Gildea, R. J.; Howard, J. A. K.; Puschmann, H. OLEX2: A Complete Structure Solution, Refinement and Analysis Program. *J. Appl. Cryst.* **2009**, *42* (2), 339–341.

(12) Chai, J. Da; Head-Gordon, M. Long-Range Corrected Hybrid Density Functionals with Damped Atom–Atom Dispersion Corrections. *Phys. Chem. Chem. Phys.* **2008**, *10* (44), 6615–6620.

(13) Frisch, M. J.; Trucks, G. W.; Schlegel, H. B.; Scuseria, G. E.; Robb, M. A.; Cheeseman, J. R.; Scalmani, G.; Barone, V.; Petersson, G. A.; Nakatsuji, H.; Li, X.; Caricato, M.; Marenich, A. V.; Bloino, J.; Janesko, B. G.; Gomperts, R.; Mennucci, B.; Hratchian, H. P.; Ortiz, J. V.; Izmaylov, A. F.; Sonnenberg, J. L.; Williams-Young, D.; Ding, F.; Lipparini, F.; Egidi, F.; Goings, J.; Pen, B.; Petrone, A.; Henderson, T.; Ranasinghe, D.; Zakrzewski, V. G.; Gao, J.; Rega, N.; Zheng, G.; Liang, W.; Hada, M.; Ehara, M.; Toyota, K.; Fukuda, R.; Hasegawa, J.; Ishida, M.; Nakajima, T.; Honda, Y.; Kitao, O.; Nakai, H.; Vreven, T.; Throssell, K.; Montgomery Jr. J. A.; Peralta, J. E.; Ogliaro, F.; Bearpark, M. J.; Heyd, J. J.; Brothers, E. N.; Kudin, K. N.; Staroverov, V. N.; Keith, T. A.; Kobayashi, R.; Normand, J.; Raghavachari, K.; Rendell, A. P.; Burant, J. C.; Iyengar, S. S.; Tomasi, J.; Cossi, M.; Millam, J. M.; Klene, M.; Adamo, C.; Cammi, R.; Ochterski, J. W.; Martin, R. L.; Morokuma, K.; Farkas, O.; Foresman, J. B. and Fox, D. J. Gaussian 16, Revision C.01. Gaussian, Inc., Wallingford CT, **2016**.

(14) Ehlers, A. W.; Böhme, M.; Dapprich, S.; Gobbi, A.; Höllwarth, A.; Jonas, V.; Köhler, K. F.; Stegmann, R.; Veldkamp, A.; Frenking, G. A Set of f-Polarization Functions for Pseudo-Potential Basis Sets of the Transition Metals Sc–Cu, Y–Ag and La–Au. *Chem. Phys. Lett.* **1993**, *208* (1–2), 111–114.

(15) Marenich, A. V.; Cramer, C. J.; Truhlar, D. G. Universal Solvation Model Based on Solute Electron Density and on a Continuum Model of the Solvent Defined by the Bulk Dielectric Constant and Atomic Surface Tensions. *J. Phys. Chem. B,* **2009**, *113* (18), 6378–6396.

(16) Otero-De-La-Roza, A.; Johnson, E. R.; Luaña, V. Critic2: A Program for Real-Space Analysis of Quantum Chemical Interactions in Solids. *Comput. Phys. Commun.* **2014**, *185* (3), 1007–1018.

(17) Otero-de-la-Roza, A.; Blanco, M. A.; Pendás, A. M.; Luaña, V. Critic: A New Program for the Topological Analysis of Solid-State Electron Densities. *Comput. Phys. Commun.* **2009**, *180* (1), 157–166.

(18) Contreras-García, J.; Johnson, E. R.; Keinan, S.; Chaudret, R.; Piquemal, J. P.; Beratan, D. N.; Yang, W. NCIPLOT: A Program for Plotting Noncovalent Interaction Regions. *J. Chem. Theory Comput.* **2011**, *7* (3), 625–632.
